# An objective quantitative diagnosis of depression using a local-to-global multimodal fusion graph neural network

**DOI:** 10.1016/j.patter.2024.101081

**Published:** 2024-11-04

**Authors:** Shuyu Liu, Jingjing Zhou, Xuequan Zhu, Ya Zhang, Xinzhu Zhou, Shaoting Zhang, Zhi Yang, Ziji Wang, Ruoxi Wang, Yizhe Yuan, Xin Fang, Xiongying Chen, DIRECT Consortium, Yanfeng Wang, Ling Zhang, Gang Wang, Cheng Jin

**Affiliations:** 1 Medical Robot Research Institute, School of Biomedical Engineering, Shanghai Jiao Tong University, Shanghai 200240, China; 2 Beijing Key Laboratory of Mental Disorders, National Clinical Research Center for Mental Disorders & National Center for Mental Disorders, Beijing Anding Hospital & Advanced Innovation Center for Human Brain Protection, Capital Medical University, Beijing 100088, China; 3 Shanghai Artificial Intelligence Laboratory, Shanghai 200232, China; 4 CAS Key Laboratory of Behavioral Science, Institute of Psychology, Beijing 100101, China; 5 Department of Psychology, University of Chinese Academy of Sciences, Beijing 100101, China; 6 School of Electronic Information and Electronical Engineering, Shanghai Jiao Tong University, Shanghai 200240, China; 7 Department of Cognitive Science, Swarthmore College, Philadelphia, PA 19081, USA; 8 Stanford University School of Medicine, Ground Floor, 875 Blake Wilbur Drive, Stanford, CA 94305-5847, USA

**Keywords:** major depressive disorder, multimodal fusion, graph neural network, brain connectivity analysis, neuroimaging biomarkers

## Abstract

This study developed an artificial intelligence (AI) system using a local-global multimodal fusion graph neural network (LGMF-GNN) to address the challenge of diagnosing major depressive disorder (MDD), a complex disease influenced by social, psychological, and biological factors. Utilizing functional MRI, structural MRI, and electronic health records, the system offers an objective diagnostic method by integrating individual brain regions and population data. Tested across cohorts from China, Japan, and Russia with 1,182 healthy controls and 1,260 MDD patients from 24 institutions, it achieved a classification accuracy of 78.75%, an area under the receiver operating characteristic curve (AUROC) of 80.64%, and correctly identified MDD subtypes. The system further discovered distinct brain connectivity patterns in MDD, including reduced functional connectivity between the left gyrus rectus and right cerebellar lobule VIIB, and increased connectivity between the left Rolandic operculum and right hippocampus. Anatomically, MDD is associated with thickness changes of the gray and white matter interface, indicating potential neuropathological conditions or brain injuries.

## Highlights

• Local-global network architecture offers a comprehensive view on depression diagnosis
• Integrating functional, structural, and demographic data improves depression diagnosis
• Interpretable AI identifies potential digital biomarkers for depression diagnosis
• Achieves superior performance on depression diagnosis in a multi-center scenario

## The bigger picture

Major depressive disorder (MDD) presents a multifaceted challenge to global mental health, given its intricate etiology involving social, psychological, and biological determinants. There is a lack of definitive diagnostic markers, and doctors therefore rely heavily on subjective methods to diagnose individuals. Interest in artificial intelligence (AI)-powered diagnostic methods is increasing because of their potential to offer a more comprehensive and objective evaluation. Current AI methods, however, often neglect the complex interplay of functional, structural, and demographic factors that characterize MDD. They seldom integrate insights from both in-depth brain region analysis and broad population-level associations. Moreover, there is a lack of strategies that can interpret AI models and identify diagnostic markers. There is, therefore, an urgent need to improve network architectures, develop effective multimodal fusion strategies, and enhance model interpretability.

## Teaser

This study introduces an artificial intelligence (AI) system using local-global multimodal fusion graph neural networks for depression diagnosis. The system integrates functional and structural neuroimaging with health records, demonstrating state-of-the-art performance in diagnosing depression and its subtypes. Built and validated on large-scale, multi-center cohorts with 2,442 participants, the system reveals abnormal brain regions and connectivity patterns, along with digital structural features in depression patients. This advance contributes to the pursuit of objective diagnostic markers and better clinical diagnosis.

## Introduction

Major depressive disorder (MDD) is one of the most common psychiatric disorders, inflicting severe symptoms that may significantly impact a person’s physical and mental well-being, social functioning, and quality of life.^1^ This disorder epitomizes the quintessential features of depressive disorder, characterized by persistent and profound sadness, loss of interest or pleasure, and other cognitive and physical disruptions. These symptoms, persisting for a minimum of 2 weeks, as defined by standard diagnostic criteria, not only underscore the distinct characteristics of MDD but also highlight its significance as a focal point in clinical research and intervention. The number of MDD patients worldwide has increased by approximately 18% in the past decade, and currently, an estimated 185 million people suffer from the disease.^2^ Unfortunately, this growing epidemic often remains shrouded or invisible, with sufferers facing stigmatization and exclusion. They frequently endure their plight in isolation rather than seeking assistance. Without proper treatment, the disease can get worse and last longer. In severe cases, it could lead to self-harm or suicide.^3^^,^^4^ Therefore, the early and precise diagnosis of MDD is vital in preventing severe outcomes and minimizing financial and emotional burdens.

The diagnosis of MDD follows a structured clinical evaluation process, primarily guided by the *Diagnostic and Statistical Manual of Mental Disorders, Fifth Edition* (*DSM*-5)^5^ and *International Classification of Diseases, 11th Revision* (ICD-11)^6^ criteria. This process typically involves a clinical interview to assess mental health history and symptomatology; the use of diagnostic scales such as the Hamilton Depression Scale (HAM-D),^7^^,^^8^ the Beck Depression Inventory (BDI),^9^ and Patient Health Questionnaire-9^10^ to quantify symptom severity; the exclusion of other potential causes for depressive symptoms; and the evaluation of symptom duration and frequency. This approach, however, depends inherently on the subjective judgment of clinicians and self-reported measures from depression scales. As a result, it is fraught with challenges, including low detection rates, high misdiagnosis risk, and unsatisfactory levels of accuracy.^11^

Recent advances in neuroimaging research have elucidated the intricate structural and functional alterations in some brain regions associated with MDD. Structural studies have converged to implicate the prefrontal cortex and anterior cingulate in the pathology of MDD, with evidence of genetic variants, neuroinflammatory markers, reduced neurogenesis, and gray matter volume (GMV) alterations in these regions.^6^^,^^12^ Additionally, hippocampal atrophy and thinner cortical gray matter have been observed in the orbitofrontal cortex, anterior and posterior cingulate, and insula in MDD patients, suggesting potential structural disconnectivity.^13^^,^^14^ Despite these findings, the effect sizes for structural brain differences are generally small, and their predictive value at the individual level remains limited.^12^ Functionally, MDD is characterized by altered connectivity within the salience network, frontoparietal network, and default mode network.^6^^,^^15^ Notably, the salience network, which includes the amygdala and anterior cingulate cortex, tends to show increased activity in response to emotional stimuli, suggesting a hyperresponsive emotional processing system.^16^^,^^17^^,^^18^ Concurrently, the default mode network, implicated in self-referential thoughts and rumination, often exhibits altered connectivity, which may contribute to the cognitive and affective symptoms of MDD.^15^^,^^19^ Additionally, the frontoparietal network, critical for several higher-order cognitive processes, is frequently found to be hypoactive, potentially underlying the executive dysfunction observed in depression.^20^^,^^21^

Despite these findings, the underlying pathophysiology of depression remains largely elusive, and there are currently no internationally recognized effective molecular or imaging biomarkers, hampering research in both its diagnosis and treatment. In this context, it is appealing to establish an objective and quantitative system for the automatic diagnosis of MDD and further guide the uncovering and understanding of pathological mechanisms and markers of depression.

Existing research underscores the critical role that neural circuits play in both causing and characterizing brain disorders.^22^^,^^23^ As a commonly used non-invasive imaging technique, MRI serves as an effective tool for examining brain structure and functionality. Resting-state functional MRI (rs-fMRI) and structural MRI (sMRI) are two commonly used modalities for investigating brain function and anatomy, respectively. rs-fMRI measures brain activity by detecting changes associated with blood flow, revealing disrupted functional brain activities in psychiatric disorders. At the same time, sMRI is adept at revealing finer anatomical information due to its high spatial resolution,^24^ providing an effective tool for assessing anatomical alterations in the brain. Additionally, clinical information such as gender, age, and education can provide valuable information for the diagnosis of depression at the demographic level. The fusion of these multimodal data is expected to provide more comprehensive information for the diagnosis of MDD.

A majority of current MDD diagnosis methods based on MRI data adhere to the following pipeline: (1) feature engineering, including feature selection using either pre-trained weights, statistical analysis,^25^ or prior knowledge, and feature reduction via clustering-based or decomposition-based techniques; (2) disease diagnosis based on the selected features, using classifiers such as support vector machine,^26^^,^^27^^,^^28^ Gaussian process classifier,^29^ decision tree,^30^^,^^31^ Naive Bayes,^25^ and emerging deep learning models.^32^^,^^33^ Nevertheless, such traditional approaches have some intrinsic shortcomings. On the one hand, coarse and empirical feature extraction may lead to suboptimal performance in subsequent stages of classification. On the other hand, these methods failed to adequately capture the topological information of the brain network, which is essential for understanding functional connectivity and signal transmission across various brain regions.

Graph neural networks (GNNs) represent data as graphs composed of nodes and edges and iteratively update the representations of nodes by exchanging information with their neighbors through pairwise message passing. Recent studies have successfully employed GNN in investigating mental disorders, including Alzheimer disease (AD),^34^^,^^35^^,^^36^ autism spectrum disorder (ASD),^37^^,^^38^ and MDD.^39^^,^^40^ The correspondence between graph structure and brain anatomy, alongside the similarity between the message-passing mechanisms and the physiological functions of the brain, highlights the potential of GNN to more effectively retrieve the underlying information of the brain from MRI images.

Generally, GNN-based methods for mental disease diagnosis can be divided into two categories: regional GNN methods and subject GNN methods. Regional GNN methods recognize regions of interest (ROIs) in the brain as nodes and links between different ROIs as edges, thus turning disease diagnosis into a graph-level classification task.^41^^,^^42^^,^^43^ These approaches offer a fine-grained analysis of brain regions, as defined by the ROIs, and their interactions. For instance, specific brain regions associated with emotional regulation, such as the amygdala and prefrontal cortex, are often treated as nodes. The construction of edges based on neural connections or functional correlations between these regions allows for a detailed analysis of local brain circuitry. This fine-grained approach is highly effective in capturing disease-related local brain regions and biomarkers, enabling precise identification of neural abnormalities related to the etiology of depression. However, it may overlook the inter-individual relationships and variations that could influence the manifestation of the disorder. In contrast, the subject GNN methods incorporate the population aspect of mental disorders, thus turning disease diagnosis into a node-level classification task. In these methods, each node corresponds to an individual and each edge encodes the relationship between two subject nodes evaluated by imaging data (e.g., MRI, computed tomography) or demographic data (e.g., age, gender, acquisition site, educational attainment).^39^^,^^40^ Here, the integration of diverse demographic and imaging data provides a broader perspective. By considering the relationships between individuals, these methods can identify population-level patterns and trends that might be associated with the occurrence and development of depression. However, the graph modeling method used in this approach may limit interpretability and pose challenges in specific biomarker extraction.

In the realm of clinical application, the two methods exhibit distinct focal points. Regional GNN methods are tailored to an individual-centric perspective, diagnosing diseases by conducting a granular examination of the signal characteristics and functional connectivity features within discrete brain regions. Subject GNN methods adopt a population-based perspective, aiming to achieve diagnostic objectives through the integration and comparative analysis of the features of the study subject with those of similar individuals within the larger population. In essence, regional GNN and subject GNN can be likened to symptomatic diagnosis and epidemiological diagnosis, respectively, mirroring the two fundamental paradigms of medical diagnostics. The combination of regional and subject GNN methods offers the potential to leverage the strengths of both methods for a better understanding of the etiology and quantitative diagnosis of depression.

From a mechanistic standpoint, the differences between regional and subject GNN methods reflect varying levels of granularity in understanding depression. Regional GNN methods offer insights into the local, intracranial dynamics, providing a window into the direct physiological correlates of depression. Subject GNN methods, however, shed light on the interplay between individual differences and environmental factors, illuminating the complex web of influences that contribute to the manifestation of depression.

In the context of advancements in machine learning, regional GNN and subject GNN methods drive the development and innovation of algorithms in distinct and complementary directions. Regional GNN methods push the boundaries of graph-based learning by requiring models that can handle intricate and variable graph structures. Subject GNN methods challenge the field by demanding algorithms with the capacity to incorporate varying data types and capture the nuanced relationships within a population graph.

In summary, the current GNN-based approaches for mental disease diagnosis, namely regional GNN and subject GNN, offer complementary perspectives in understanding and diagnosing depression, indicating the potential for integrating Regional GNN and Subject GNN into a local-global network structure. This fusion is expected to enhance our understanding of the etiology of depression, improve diagnostic accuracy, and advance machine learning techniques in mental health diagnostics.

Regarding graph structure, recent research has revealed that the topological information within fMRI-based graphs is crucial to the performance of a GNN on MDD classification.^44^ However, in many studies, the graph structure is initialized in a rudimentary manner. The construction of local graphs based on MRI data typically begins with the selection of a brain atlas that defines a set of ROIs as nodes. Then, the fMRI blood-oxygen-level-dependent (BOLD) signal series of each ROI is extracted for generating edges. Finally, the edge weights are calculated between each node pair using metrics such as Pearson correlation and partial correlation. Despite its prevalence in the existing literature,^38^^,^^43^^,^^45^ this method features two flaws. First, the simple linear correlation ignores the temporal dynamics of fMRI signals, which play a vital role in reflecting brain activity and connectivity. Second, the constructed graph remains static during model training and fails to be optimized according to the target task. Given these limitations, the performance of GNNs is often less than optimal. Therefore, it is necessary to find a method that allows the graph structure defined by the adjacency matrix and the network parameters to train jointly, facilitating the dynamic adaptation of graph structure and active learning.

To synthesize the merits of the regional (local) and subject (global) GNN methods mentioned above while mitigating their limitations, and to construct a flexible and reasonable graph structure with features in multimodalities, a local-to-global multimodal fusion GNN (LGMF-GNN) was proposed for the objective quantitative diagnosis of MDD. Specifically, a local ROI GNN is utilized to generate graph-level embedding from the time series signal of various brain ROIs described by rs-fMRI. Then, a global subject GNN further fuses local functional connectivity features described by the above local embedding, anatomical features from sMRI, and demographic features from non-imaging data into a unified space. A final diagnosis that leverages the local-global and multimodal comprehensive information is then generated. By transitioning from regional brain graphs to subject graphs, this framework facilitates the extraction of individualized fine-grained features and the integration of multimodal data at the population level, culminating in a synergistic improvement and a progressive methodology for intelligent diagnosis.

This study involves large-scale, multi-center datasets collected from 24 institutions in 3 nations. Model development and internal validation were conducted using the Japanese Strategic Research Program for the Promotion of Brain Science (SRPBS) and REST-meta-MDD datasets, which include a total of 2,027 individuals, achieving a 10-fold cross-validation (CV) area under the receiver operating characteristic curve (AUROC) of 80.64% ± 5.74% and a leave-one-subject-out cross-validation (LOSO CV) AUROC of 73.71% ± 4.12%. External independent testing was performed using the Anding and OpenNeuro datasets, achieving AUROCs of 72.91% and 70.30%, respectively. In comparison with the state-of-the-art (SOTA) methods like BrainGNN on the identical dataset, the proposed model showed improvements of at least 5.46% in accuracy and 7.60% in AUROC. Furthermore, through in-depth interpretation research, we identified a collection of potential biomarkers that describe relational features in MDD patients, such as functional alterations in brain regions like the prefrontal cortex and hippocampus and structural alterations described by GMV and white matter volume. Our study paves the way for an objective quantitative diagnosis of MDD. All the data and code have been made accessible to the public.

## Results

### Data acquisition

This study employed four datasets comprising MDD patients and healthy controls (HCs), as outlined in Table 1. The SRPBS dataset (229 MDDs vs. 228 HCs) and the REST-meta-MDD dataset (814 MDDs vs. 756 HCs) were used for training and internal validation, while the Anding dataset (196 MDDs vs. 177 HCs) and the OpenNeuro dataset (21 MDDs vs. 21 HCs) were used for external independent testing. Collectively, these datasets incorporate data from 2,242 participants, each providing a complete set of information across three modalities: fMRI, sMRI, and demographic characteristics.

**Table 1:** Demographic and clinical characteristics of included multi-site participants

Dataset and variable	Depressive disorder	Healthy control
Anding		
sample size, N	196	177
age, y, mean ± SD	29.22 ± 8.40	27.63 ± 6.96
female sex, N (%)	148 (75.5)	116 (65.5)
episode status		
first episode, N (%)	93 (47.4)	–
recurrent, N (%)	96 (49.0)	–
unknown, N (%)	7 (3.6)	–
medication status		
drug naive, N (%)	169 (86.2)	–
treated, N (%)	16 (8.2)	–
unknown, N (%)	11 (5.6)	–
duration of illness, months, mean ± SD	5.00 ± 5.21	–
education, y, mean ± SD	15.69 ± 2.62	16.48 ± 2.75
SRPBS		
sample size, N	229	228
age, y, mean ± SD	43.05 ± 13.49	44.07 ± 14.89
female sex, N (%)	104 (45.4)	142 (62.3)
BDI-II, mean ± SD	27.43 ± 14.14	–
REST-meta-MDD		
sample size, N	814	756
age, y, mean ± SD	34.45 ± 11.61	34.64 ± 13.18
female sex, N (%)	519 (63.6)	446 (59.0)
episode status		
first episode, N (%)	404 (49.6)	–
recurrent, N (%)	208 (25.6)	–
unknown, N (%)	202 (24.8)	–
medication status		
drug naive, N (%)	300 (36.9)	–
treated, N (%)	221 (27.1)	–
unknown, N (%)	293 (29.3)	–
duration of illness, months, mean ± SD	39.44 ± 61.40	–
HAM-D, mean ± SD	21.17 ± 6.54	–
education, y, mean ± SD	11.95 ± 3.38	13.56 ± 3.42
OpenNeuro		
sample size, N	21	21
age, y, mean ± SD	32.04 ± 9.38	33.81 ± 8.49
female sex, N (%)	17 (81.0)	15 (71.4)
BDI-R, mean ± SD	25.11 ± 10.09	4.5 ± 4.64

HAM-D, Hamilton Depression Scale; BDI-II, Beck Depression Inventory-Second Edition; BDI-R, Beck Depression Inventory-Russian versions.

### Hierarchical graph structure for MDD diagnosis from both individual and population perspectives

**Figure 1:**
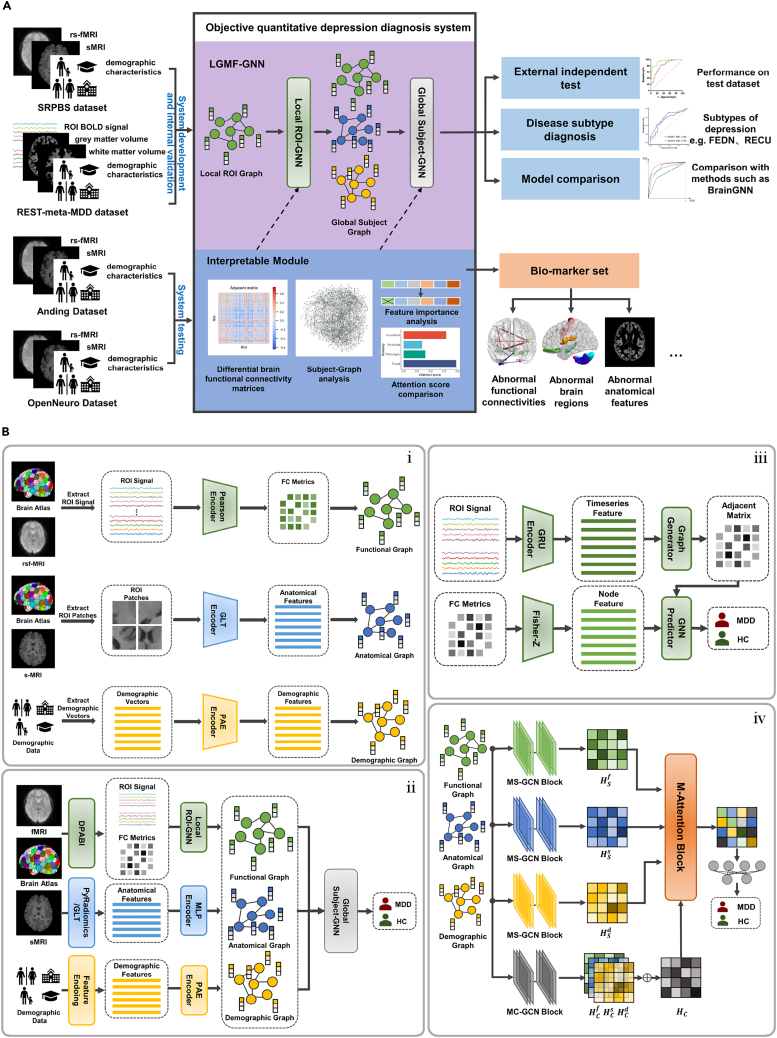
Objective quantitative depression diagnosis system (A) Workflow of the diagnosis system. (B) Framework of LGMF-GNN. (i) Feature extraction and graph initialization. Features were extracted from the data of the three modalities and further used to initialize the functional graph, structural graph, and demographic graph. (ii) The hierarchical LGMF-GNN structure. Local ROI GNN generates the embedding for each subject based on the ROI graph and ROI BOLD signals of each individual. Global subject GNN aggregates multiple information from the subject graphs of different modalities to obtain the final prediction result. (iii) Detailed network structure of local ROI GNN. (iv) Detailed network structure of global subject GNN.

Previous studies have often been limited to a single perspective when making the diagnosis, which results in suboptimal diagnostic performance and inadequate explanations of the underlying mechanisms. The LGMF-GNN was proposed for accurate and interpretable MDD diagnosis. LGMF-GNN generates the diagnosis by integrating information from both individual and population levels, as well as multiple data modalities that reflect brain structural and functional status (illustrated in Figure 1.). Specifically, our method employs two complementary sub-models: the ROI GNN and the subject GNN. The ROI GNN captures local brain information by representing the brain as a region of interest (ROI) graph, where brain regions serve as nodes and their functional interactions serve as edges. This sub-model focuses on the localized individual brain region-level perspective. Conversely, the subject GNN analyzes the embedding information among subjects from the global perspective by operating on the subject graph. In this graph, subjects and their relationships are represented as nodes and edges, respectively, and the edge weights are calculated based on the similarity of functional, anatomical, and demographic information. This sub-model adopts the population-level perspective. By integrating these sub-models and diverse data modalities, the LGMF-GNN achieves robust and precise MDD diagnoses.

In our proposed local-global model, the initial step is constructing ROI graphs for learning high-quality subject brain embeddings. A learnable adjacency matrix is derived by analyzing the ROI BOLD time series (see Figure 1B and the section “local ROI GNN” in the methods section for details). The node attributes are defined as the corresponding columns of the functional connectivity matrix obtained from rs-fMRI images. To generate an ROI graph representation, a graph convolutional network (GCN) with the attention mechanism is implemented in the local ROI GNN to aggregate information across ROIs with emphasis. As shown in Figure 1B, a regional embedding is generated by the gated recurrent unit (GRU) encoder based on the BOLD time series. Then, the graph generator takes the embedding as input and outputs a learnable adjacent matrix for each subject. Subsequently, the GNN predictor applies the attention mechanism to the learned graph structure and node features to acquire the local graph embeddings and local classification results. The local embeddings are then employed as the initial functional node features of the global subject GNN. To extract features from T1 MRI images and demographic data, we designed the global-local transformer (GLT) encoder and pairwise association encoder (PAE) to encode the raw anatomical and demographic data of each individual into one-dimensional feature vectors (see Figure 1B and the section “feature extraction” in the methods section). In the subject GNN, features from functional, anatomical, and demographic data are modeled as node attributes of the three subject graphs (illustrated in Figure 1B). In the population view, the modality-specific (MS)-GCN block is designed to generate modal-specific representations, while the modality-common (MC)-GCN block is designed to distill a modal-common representation. The multimodal (M)-Attention block refines these representations to produce the final representation, which contains the most important and expressive information from all three modalities. Finally, a multi-layer perceptron (MLP) serves as a classifier to produce the final global prediction. An enriched elucidation of the model structure can be obtained from the methods section.

### Performance on the SRPBS dataset

We evaluated the prediction performance of the proposed system as an MDD vs. HC binary classification task using five metrics: accuracy (ACC), the area under the receiver operating characteristic curve (AUROC), precision, recall, and F1-score. We evaluated the diagnostic capabilities of the local and global networks separately by examining the single-perspective diagnosis result of the two models on the SRPBS dataset. This dataset contains 229 MDDs and 228 HCs from six sites (Table 1). For the local ROI GNN, we partitioned the brain into ROIs using both automated anatomical labeling (AAL) and Craddock200 (CC200) brain atlases and conducted separate experiments for each. For the global subject GNN, we compared the 10-fold CV results obtained both with and without the inclusion of structural features derived from the T1 sMRI images. The independent performances of the two models are detailed in Table 2–1. When local ROI GNN and global subject GNN operate independently, the model’s performance, as indicated by ACC and AUROC, exhibits a variation of less than 2%. We have also conducted an ablation study to assess the role of the ROI GNN module, and the experimental results are shown in Table S13.

**Figure 2:**
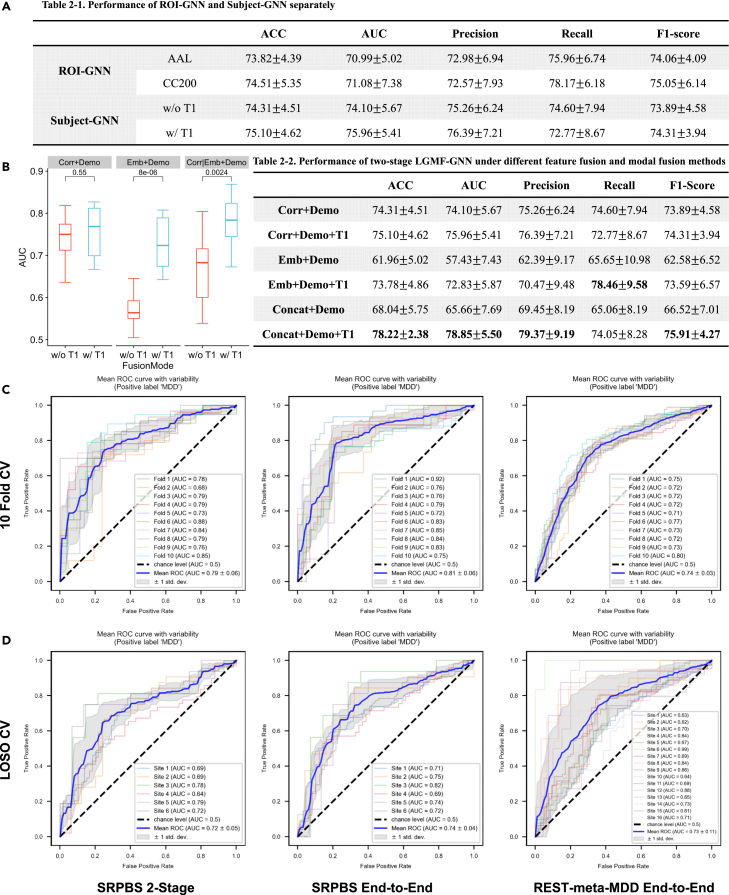
Performance on the SRPBS and REST-meta-MDD dataset (A) Performance of ROI GNN and Subject GNN separately. (B) Boxplot of AUROC of two-stage trained LGMF-GNN under different feature fusion and multimodal fusion methods. Three different groups represent different compositions of the functional state feature, and different colors represent different multimodal fusion methods. The t test shows that the addition of T1 modality significantly improves the AUROC of the model. (C) 10--Fold CV ROCs of two-stage and end-to-end trained LGMF-GNN. (D) LOSO CV ROCs of two-stage and end-to-end trained LGMF-GNN.

Furthermore, we conducted a 10-fold CV on local and global networks in a two-stage fashion. Specifically, we incorporated the embedding obtained by the local ROI GNN into the global model for the construction of the functional subject graph, thus linking the local network with the global network as the two-stage trained LGMF-GNN. To optimize the mode of local-global feature fusion and multimodal fusion, six experiments were designed, differing in the inclusion of structural modality (T1 sMRI) and the composition of functional-state node features. Three such composition modes were introduced: correlation, embedding, and concatenation. In the correlation (Corr in Table 2-2 in Figure 2B) mode, the flattened functional connectivity matrixes were set as node features of the functional graph. In the embedding (Emb in Table 2-2) mode, the local ROI GNN embeddings were set as node features of the functional graph. In the concatenation (Concat in Table 2-2) mode, the concatenation of the flattened functional connectivity matrixes and local ROI GNN embeddings was set as node features. For each functional feature configuration, we examined the performance of the model with and without the inclusion of structural modality, resulting in six experiments: Corr+Demo, Corr+Demo+T1, Emb+Demo, Emb+Demo+T1, Concat+Demo, and Concat+Demo+T1. The results of the experiments are shown in Table 2-2. It can be seen that the concatenation feature fusion method and the inclusion of T1 modality features significantly improved the classification performance (t test *p* = 0.55, <0.001, <0.01 in the three groups).

Subsequently, we jointly trained local and global networks in an end-to-end manner, speculating that this approach may enhance the embeddings generated by the local network to better fit the downstream global network. The obtained AUROCs are depicted in Figure 2C. The proposed model achieved an AUROC of 78.85% ± 5.50% when trained in two-stage mode and an AUROC of 80.64% ± 5.74% when trained in end-to-end mode (for more detailed results, refer to Table S1). The M-Attention block of the subject GNN is used to apply attention mechanisms between three MS embeddings and one MC embedding to allocate different levels of attention to different modalities. The attention scores of different modalities learned during the fusion process can reflect their importance in the diagnosis of depression. The attention scores of the three MS embeddings were functional (0.3148), demographic (0.1598), and structural (0.1367), in descending order. Notably, the MC embedding that contains information about all three data modalities achieves the highest attention score (0.3887). We also conducted ablation experiments to explore the role of different data modalities, and the experimental results are shown in Table S14.

### Performance on the larger and more complex REST-meta-MDD dataset

The REST-meta-MDD dataset is more intricate than the SRPBS dataset, which contains 814 MDDs at different disease stages as well as 756 HCs (Table 1). The dataset also contains patients with different MDD subtypes, including first-episode MDD and recurrent MDD, and some were scanned while the patient was on antidepressants. Such a dataset would more closely mirror real-world clinical scenarios while presenting increased analytical challenges. Additionally, data collection across 16 distinct sites introduces pronounced “site effects.”^46^ These effects arise from differences in the MRI equipment or scanning procedures used at different imaging sites, leading to variations in imaging results or data. Site effects can obscure features of interest in neuroimaging and decrease statistical power, undermining the credibility and generalizability of the system. Following prior experiments, we conducted a 10-fold CV on the REST-meta-MDD dataset in the end-to-end training mode using all three modalities. The performance of the proposed system is shown in Figure 2C and Table S2.

To further investigate the impact of site effects on model training and generalization performance, we conducted 10-fold CV on the single-site data of the 20th cohort (S20) in the REST-meta-MDD dataset and the 6-site data of the SRPBS dataset using the two-stage trained LGMF-GNN, without specifically addressing site effects. The results of these evaluations are presented in Table S3. The analysis revealed a marked decline in system performance in multi-site scenarios compared to single-site scenarios, with ACC dropping by 22.90% and AUROC by 27.08%, This decline underscores the substantial impact of site effects on the proposed model, detrimentally affecting its performance. We conducted a thorough evaluation and discussion on the site effects in the “The evaluation of site-effect” section of supplemental experimental procedures, where we proposed solutions from the perspectives of data processing, system design, and training strategies. Although our proposed system cannot achieve similar performance in multi-site scenarios as in single-site scenarios, the five strategies (detailed in “methods for suppressing site effects and data enhancement and ablation study” in the supplemental experimental procedures) presented proved effective in suppressing site effects and mitigating performance loss. In the six-site scenario, these strategies facilitated a 9.73% reduction in performance loss, elevating the accuracy to 78.75% from 69.02%, as documented in Table S16.

### Performance across MDD subtypes and under medication influence

Beyond the fundamental classification between MDDs and HCs, we further tested the capabilities of the proposed system by distinguishing between HCs and more specific MDD subtypes. Specifically, we divided the MDD patients in the REST-meta-MDD dataset into a “first-episode drug-naive” (FEDN) subset and a “recurrent” (RECU) subset. We then assessed the ability of the system to distinguish between HCs and these two MDD subtypes. The results indicated that the system accurately differentiated FEDN patients from HCs with a classification accuracy of 75.13% (AUROC: 76.73%), and RECU patients were distinguished from HCs with an accuracy of 74.05% (AUROC: 72.28%). When differentiating between FEDN and RECU patients, the classification accuracy was 76.92% (AUROC: 68.89%). For more detailed results, refer to Tables S4–S7. These results suggest that the model exhibits a slightly lower diagnostic accuracy for the RECU MDD subgroup compared to the FEDN MDD subgroup when distinguishing from HCs, but it still maintains its robustness.

We also investigated the impact of medication on the model’s diagnostic performance. The analysis was feasible only for the REST-meta-MDD dataset as it provides detailed medication information. We compared the model’s AUROC for drug-naive (*n* = 300) and treated (*n* = 221) MDD patients against HCs. The AUROC for the drug-naive group was marginally larger than that for the treated group, indicating a slightly better diagnostic performance for the former. However, the difference was minimal, with a difference of only 0.02, which underscores the robustness of the proposed model against medication status. Visual representations in Figures 4A–4C provide further insight into the output of the model for both groups. The density plots indicate that medication status impacts the distribution of predicted disease probabilities, with the treated group showing higher density in the lower probability range, suggesting symptom mitigation, whereas the drug-naive group exhibited a higher density in the higher probability range, reflecting more pronounced symptoms.

In summary, the proposed model demonstrates robust diagnostic capabilities across different MDD subtypes, with only slight variations in accuracy observed. Medication status appears to have a nuanced effect on the output of the model, which aligns with clinical expectations, further validating the ability of the model to capture intrinsic disease patterns.

### Generalizability on the external independent Anding and OpenNeuro datasets

We evaluated the generalization performance of the proposed system on the Anding and OpenNeuro datasets. The Anding dataset is an external independent dataset containing 196 MDDs and 177 HCs from Anding Hospital, China (Table 1). The participants in this dataset belong to the same population as the training set, namely the Asian population. All the participants had refrained from any medication treatment for 2 weeks before data collection. The dataset includes both FEDN and RECU MDD cases, which could effectively test the generalization ability of the system. The OpenNeuro dataset is an external independent dataset that includes 21 patients with depressive disorder and 21 HC participants (Table 1). Diverging from the previous three datasets derived from the Asian population, this dataset contains individuals from the European population, who exhibit relatively less severe depressive symptoms. The utilization of the OpenNeuro dataset as an external independent test set allows for a rigorous assessment of the system’s generalization performance under more challenging conditions.

For the external independent test procedure, the system was trained on the consolidated SRPBS and REST-meta-MDD datasets and tested on the Anding and OpenNeuro datasets, respectively. Finally, the system achieved an ACC of 69.97%, an AUROC of 72.91%, and an F1-score of 71.57% on the Anding dataset. On the OpenNeuro dataset, the system attained an ACC of 69.05%, an AUROC of 70.30%, and an F1-score of 71.11%. The external independent test results on the Anding dataset show that while the ACC and AUROC of the comparison models dropped to less than 60%, LGMF-GNN still achieved an ACC of 69.97% and an AUROC of 72.91% (for more detailed results, refer to Tables S8 and S9). These results demonstrate a good generalization performance of the proposed system.

### Comparison experiments and cross-site generalization capabilities

To evaluate the generalization ability of LGMF-GNN, we conducted LOSO CV on the SRPBS and REST-meta-MDD datasets, as exhibited in Figure 2D. The ROCs indicate that although the performance decreased when the model was trained and tested on cross-site data, the system still achieved an overall good performance, with an LOSO CV AUROC of 73.33% ± 5.70% and 75.57% ± 9.45% on the SRPBS dataset and the REST-meta-MDD dataset, respectively. To further validate the proposed system, LGMF-GNN is compared to SOTA GNN models for brain disease diagnosis. Specifically, we assessed the performance of LGMF-GNN against traditional GCN,^47^ graph isomorphism network (GIN),^48^ and graph attention network (GAT)^49^ and popular brain GNNs, namely BrainGNN,^38^ edge-variational graph convolutional network (EV-GCN),^50^ local-to-global graph neural network (LG-GNN),^51^ contrastive graph pooling (ContrastPool),^52^ phenotypic edge relational graph attention network (pRGAT),^53^ multi-scale adaptive multi-channel fusion deep graph convolutional network (MAMF-GCN),^39^ specificity-aware federated graph learning (SFGL),^54^ causality-inspired graph neural network (CI-GNN),^55^ and interpretable graph neural networks for connectome-based brain disorder analysis (IBGNN).^56^ Among these, BrainGNN, SFGL, CI-GNN, and IBGNN are local-view methods based on graph-level classification of ROI graphs; EV-GNN, pRGAT, and MAMF-GCN are global-view methods based on node-level classification on population graphs; and LG-GNN and ContrastPool are local-to-global methods. Comparing our proposed method with these approaches will demonstrate its advantages. A comprehensive introduction to each method used for comparison, including the specifics of their implementation and the chosen hyperparameter configurations, is detailed in the comparison experiments section of the supplemental experimental procedures and Tables S10.1–S10.12.

**Figure 3:**
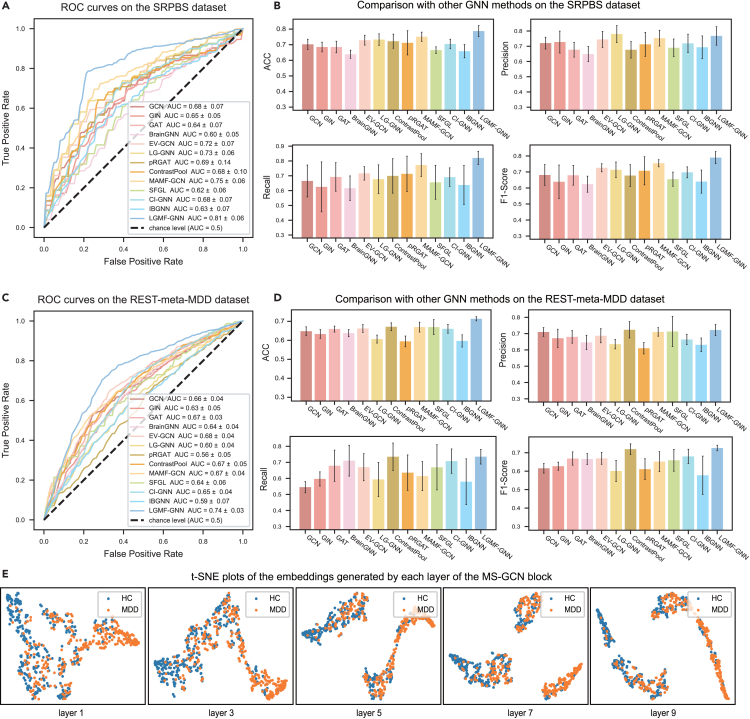
Comparison with other GNN methods (A) ROCs comparison of different models on the SRPBS dataset. (B) Histogram comparing the results of the proposed model and the SOTA models across various evaluation metrics in the SRPBS dataset. The histogram displays the mean performance metrics for each model, with error bars denoting the standard error of the mean. (C) ROCs comparison of different models on the REST-meta-MDD dataset. (D) Histogram comparing the results of the proposed model and the SOTA models across various evaluation metrics on the REST-meta-MDD dataset. The histogram displays the mean performance metrics for each model, with error bars denoting the standard error of the mean. (E) t-SNE plots of the embeddings generated by each layer of the MS-GCN block.

The results of 10-fold CV are shown in Figures 3A–3D, in which our system, LGMF-GNN, exhibited optimal performance for MDD diagnosis. The system achieved an increase in ACC of at least 3.65% and an increase in AUROC of 5.39% in the SRPBS dataset. Moreover, it generated at least a 4.20% increase in ACC and a 5.18% rise in AUROC on the REST-meta-MDD dataset, surpassing the results of previously mentioned models. In contrast, other methods were significantly affected by site effects and failed to produce accurate classifications, with ACC hovering at approximately 70% (for more detailed results, refer to Tables S11 and S12). Except for MAMF-GCN, all other tested methods exhibited an AUROC of less than 75%.

The t-distributed stochastic neighbor embedding (t-SNE) plots in Figure 3E visually depict the embedding outputs at each layer of MS-GCN. It is evident that as the number of layers increases, the embeddings of the same class become more clustered, while the distinction between two clusters of different classes becomes more pronounced. This observation suggests that Snowball GCN effectively mitigates the issue of oversmoothing, which often arises when the number of layers in GNN increases. We believe this is one of the key factors contributing to the superior performance of the proposed model compared to other GNN networks.

### System calibration and its association with self-test depression scale score

**Figure 4:**
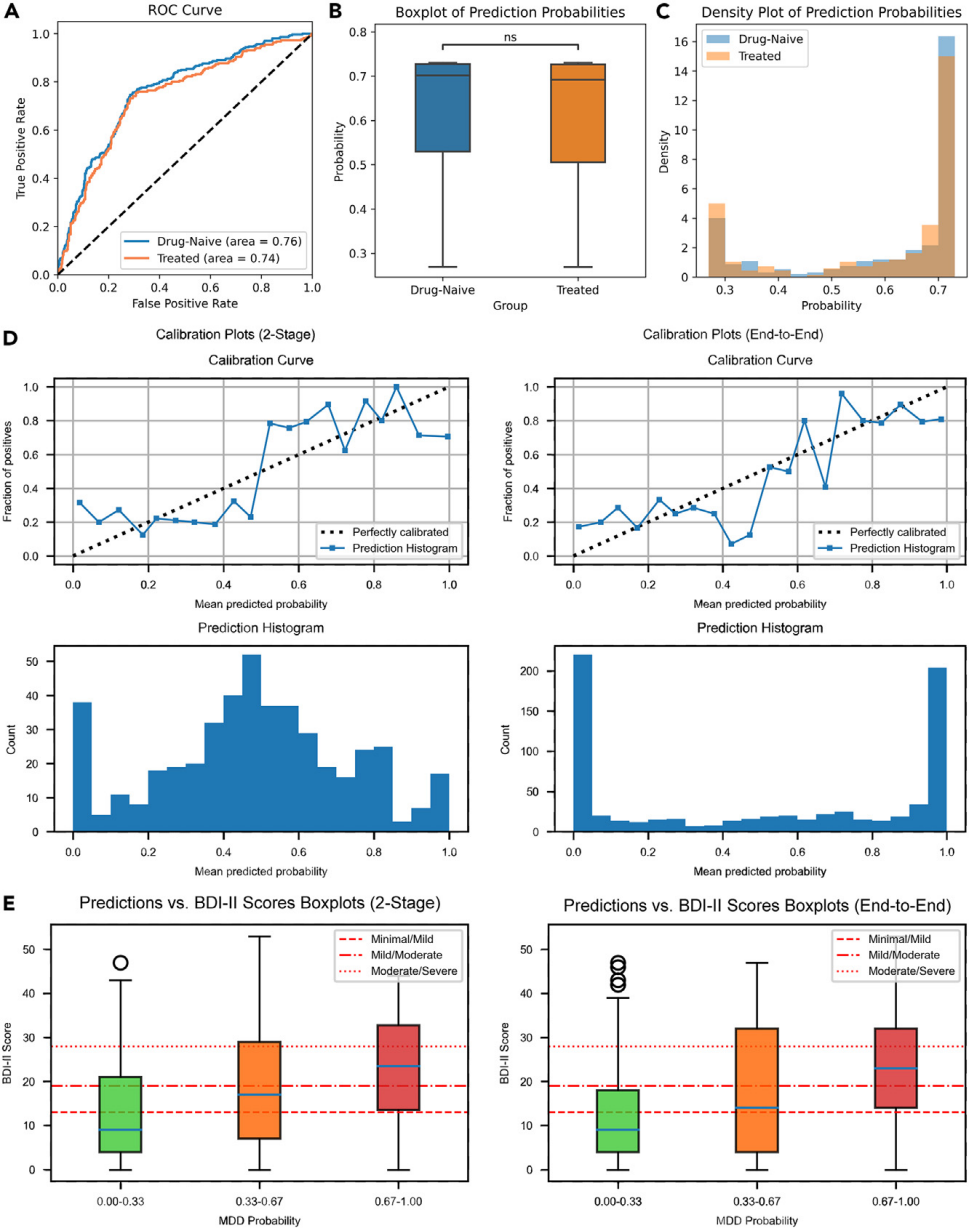
System calibration and association with self-test score of depression scale (A) The ROC of drug-naive MDD vs. HC and treated MDD vs. HC on the REST-meta-MDD dataset. (B) Boxplot of prediction probabilities output by LGMF-GNN for drug-naive and treated patients. The “ns” notation in the plot signifies that the p-value derived from the t-test is greater than or equal to 0.05, indicating no statistically significant difference of model prediction probabilities between the two patient groups. (C) Density plots of prediction probabilities output by LGMF-GNN for drug-naive and treated patients. (D) Calibration curve of two-stage and end-to-end trained LGMF-GNN. (E) Boxplot of system prediction probability and participants’ Beck Depression Inventory (BDI) self-rating scale scores.

In addition to generalization ability, we also assessed the calibration of the proposed system. The proposed LGMF-GNN exhibited good calibration because of its close agreement with the observed probabilities of MDD, as evaluated in the calibration curve (Figure 4D). A close examination of both the calibration curve and prediction results histogram revealed a marked distinction between the model trained under the two-stage mode and the end-to-end mode. The latter tends to provide a more decisive diagnostic outcome, as most predicted probabilities fall within the 0–0.1 and 0.9–1.0 range of bins. Furthermore, the two ends of the calibration curve of the end-to-end trained model are closer to the optimal calibration line, indicating that the disease risk is not significantly underestimated or overestimated. To further explore the relationship between the prediction results of the system and the severity of depression, we constructed boxplots between model prediction probability of illness, and participants’ self-assessed BDI score (Figure 4E). According to the BDI-II criteria, a self-rating score of 0–13 is diagnosed as minimal, 14–19 as mild, 20–28 as moderate, and 29–63 as severe depression. Notably, the median self-test scores of patients in different predicted probability buckets fall in different partitions. This observation suggests that the system not only accomplishes the binary classification task between HCs and MDDs but it also possesses the capability to estimate the severity of depression.

### System interpretation

This paper proposes a system that can assist physicians not only in diagnosing MDD but also offer references and guidance for exploring the underlying mechanisms and biomarkers of MDD. Diverging from the majority of methods that build a fixed graph structure based solely on the correlation between BOLD signals to focus only on either the individual or the population perspectives,^38^^,^^44^ our proposed system incorporates learnability for the brain functional connectivity matrix, which can be optimized through local and global tasks. This feature provides a new entry point for interpreting the learning outcomes of the network—that is to say, we believe that the functional connectivity matrix learned by the network, through both the HC group and the MDD patients, can reflect the differences in brain function and structure between these two groups.

**Figure 5:**
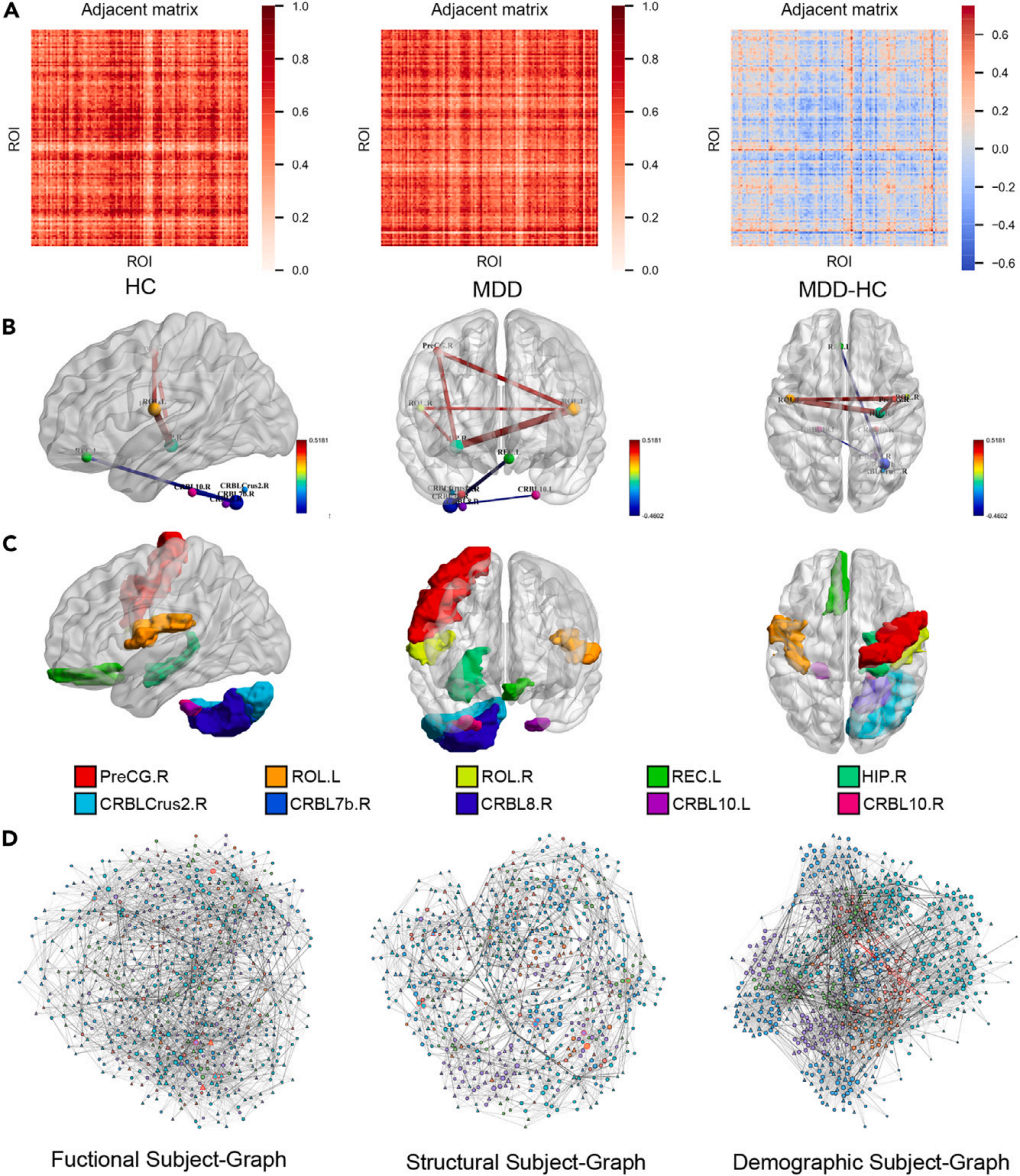
Abnormal functional connectivity in MDD patients obtained from the learnable functional connectivity matrix (A) The average functional connectivity matrix of the MDD and HC groups learned by LGMF-GNN and the differential functional connectivity matrix (MDD-HC). (B) The top five enhanced functional connections and the top five weakened functional connections shown in BrainNet Viewer. (C) The top 10 abnormal brain regions shown in BrainNet Viewer. (D) Subject graphs of functional, anatomical, and demographic modalities. “Δ” represents MDD, “○” represents HC, the different colors of nodes represent different sites, and the thickness of edges represents the strength of correlation between individuals.

To measure this difference, for the functional modality, the average functional connectivity matrix of HCs obtained from network learning was subtracted from the average functional connectivity matrix of MDD patients to obtain the heatmap shown in Figure 5A, which we refer to as the differential functional connectivity matrix. The positive values in the matrix represent the enhanced functional connections of MDD patients compared with HCs, while the negative values imply diminished functional connections. The absolute value indicates the magnitude of the difference. We identified the top five enhanced and diminished functional connections based on their magnitudes. Figures 5B and 5C present the differential functional connections filtered using the system, along with their corresponding ROIs. The top five enhanced functional connections include left Rolandic operculum-right hippocampus, right precentral gyrus-left hippocampus, right precentral gyrus-right hippocampus, right Rolandic operculum-right hippocampus, and left Rolandic operculum-right Rolandic operculum. The top five diminished functional connections are left gyrus rectus-right cerebellum lobule VIIb, right cerebellum lobule VIIb-right cerebellum lobule VIII, right cerebellum lobule VIIb-left cerebellum lobule X, right cerebellum lobule VIIb-right cerebellum lobule X, and right cerebellum crus II-right cerebellum lobule VIIb.

To gain insight into the brain regions that contribute the most to MDD diagnosis, we investigated the node degrees acquired from the absolute differential functional connectivity matrix. Based on statistical analysis from this matrix, we selected the top 10 brain regions with the largest degree to include as many potential markers as possible (Figure 5C). The selected brain regions are the right cerebellum lobule VIIb, right hippocampus, left Rolandic operculum, left gyrus rectus, left cerebellum lobule X, right precentral gyrus, right gyrus rectus, left cerebellum lobule X, right cerebellum lobule VIII, right Rolandic operculum, and right cerebellum crus II, which include five cerebellar and five cerebral regions. Notably, these regions correspond to those involved in the differential functional connectivity analysis, which adds weight to our findings. We provide a detailed discussion of the functional and structural alterations that have been identified in these brain regions in MDD patients in Table S17.

For the sMRI analysis, we extracted 1,209 dimensional radiomic feature vectors from the GMV and white matter volume images, which encompass 93 radiomic characters. To determine the factors that contribute most to the diagnosis of MDD, we experimented by assessing the impact of specific radiomic characteristics on model performance. Specifically, we masked the 93 radiomic characters one by one during the test stage and identified the top five significant characters (e.g., GLCM_MaximumProbability, FirstOrder_Uniformity) that led to the greatest decrease in model performance. We asserted that these selected characters have the most significant impact on model performance and thus are highly effective in depression diagnosis. Detailed descriptions of these five characters and their implications for depression diagnosis are provided in Table S18.

The results obtained through the interpretation of our system demonstrate consistency with previous research findings, while also presenting statistically significant and reproducible discoveries that contribute additional knowledge to the field. Prior studies have indicated that depression pathogenesis entails dysfunctional neural circuits that regulate emotions, self-reflection, reward processing, and cognitive control. Abnormal functional connectivity in regions such as the precentral gyrus^20^ and the hippocampus^57^^,^^58^^,^^59^ have been found. However, structural magnetic resonance studies have revealed that during the acute phase of first-episode depression, a reduction in hippocampal, insular, prefrontal cortical, and orbitofrontal volume is common.^60^ Notably, half of the abnormal brain regions in MDD patients specified by the model interpretations in this paper are located in the cerebellum. Historically underexplored beyond motor functions, the cerebellum is now recognized for its significant role in emotional and cognitive management. Liu et al.^61^ found a correlation between abnormal cerebellar functional connectivity and depression in adults. Their research revealed that MDD patients exhibited markedly reduced cerebellar functional connectivity relative to both the default mode network (mainly consisting of the ventral medial prefrontal cortex and posterior cingulate/supraoccipital gyrus) and the executive control network (primarily involving the superior frontal cortex and orbitofrontal cortex). Xu et al.^62^ found that emotional memory and the severity of depressive symptoms are associated with structural changes in both the posterior and anterior gray matter regions in the cerebellum in MDD sufferers.

## Discussion

In this study, we approached the depression diagnosis task from an individual-to-population perspective and facilitated an objective and quantitative diagnosis by effectively integrating multiple modalities, including functional, structural, and demographic information through a local-global multimodal fusion network. The interaction between individual brain regions and population graphs offers abundant referential information for disease diagnosis, improving the system classification performance and contributing to learning task-driven brain functional connectivity matrix. This, in turn, improves the interpretability of the system and provides innovative ways of analyzing disease mechanisms. Moreover, the GNN provides a powerful tool for high-quality fusion of multimodal and heterogeneous data, which offers multi-dimensional information for disease diagnosis. Data from various modalities offer different advantages, each contributing to a more holistic understanding of the disease.

We conducted our experiments on large multi-center datasets involving 2,442 participants from 24 sites. The results of 10-fold CV demonstrated that the system could effectively distinguish healthy individuals from MDD patients, achieving an ACC of 78.85% ± 5.50% and an AUROC of 80.64% ± 5.74% on the SRPBS dataset. Meanwhile, on the REST-meta-MDD dataset, it recorded an ACC of 71.34% ± 1.50% and an AUROC of 73.67% ± 2.67%. In the MDD subtype classification task, our system achieved similar performance in the HC vs. FEDN and HC vs. RECU classification tasks, with an ACC of 75.13% ± 3.16% and 74.05% ± 4.34%, respectively. This indicates the robustness of the proposed system. Furthermore, the system maintained a stable performance even in the challenging task of classifying FEDN and RECU subtypes, with an ACC of 76.92% ± 8.94%, demonstrating both expertise in diagnosing depression as a whole and a significant capability to classify subtypes within, which caters to actual clinical needs. The correlation between the predictive outcomes of the system and the patients’ BDI self-assessment scores has shown that the diagnostic outputs of the system can accurately indicate the severity of depressive symptoms. The results of the LOSO CV demonstrated that the system effectively overcame the problems caused by site effects and exhibited strong generalization ability. The comparative experiments of LGMF-GNN with SOTA methods on various datasets also confirm this point.

The application of GNNs in diagnosing psychiatric disorders such as MDD and in pinpointing affected brain regions has undergone a surge in recent times. However, several challenges persist, including oversmoothing issues, a singular perspective, and limited interpretability.^63^^,^^64^^,^^65^^,^^66^^,^^67^ Our approach aligns with and expands upon the current insights into MDD diagnostic models, emphasizing the advantages of both local and global perspective-based GNN depression diagnostic models. By developing a local-global network architecture, enhanced by sophisticated network modules and optimized training strategies, the proposed method not only synthesizes the benefits of existing methodologies but it also builds upon them. It offers a comprehensive global perspective through the population graph, while simultaneously preserving the detailed information of individual brain regions for the diagnosis of MDD. Comparative experimental results have demonstrated that the LGMF-GNN proposed in this paper achieves superior performance compared to current advanced local, global, and local-global GNN methods. Moreover, in line with the contemporary shift toward multimodal integration in artificial intelligence (AI)-based depression diagnostic approaches,^68^^,^^69^^,^^70^ the multimodal fusion strategy introduced in this study adeptly addresses the complexity of MDD by integrating various data sources. This approach significantly reduces the constraints inherent in single-modal data analysis found in current diagnostic models. Consequently, it offers a more nuanced and comprehensive insight into the diverse manifestations of the disease, enhancing our understanding of its heterogeneity and complexity.

Finally, through interpretation studies, we proposed a set of biomarkers, including abnormal brain functional connections and anatomical structures that can provide valuable insights for the MDD diagnosis from both functional and anatomical perspectives. These works make up for the deficiency of interpretability of MDD diagnostic models, providing alternative methods and perspectives for the interpretation of these models.

In conclusion, interpretable, end-to-end learning with a hierarchical graph structure can promote objective and quantitative diagnosis of MDD and has the potential to identify its underlying mechanism.

### System generalization ability

Generalizing across multiple sites poses a significant challenge for a neuroimaging-based classifier due to different MRI scanners, acquisition parameters, and participant instructions. This knotty problem of site effects was also present in our study: three public multi-site datasets and one private dataset were used, involving 24 sites, 1,182 patients with MDD, and 1,260 HCs. The performance of the system in single-site and multi-site scenarios shown in Table S3 indicates that the site effects have a significant negative impact on model training and testing. Therefore, to mitigate this interference and improve the generalization performance of our system, we utilized the ComBat method to harmonize the data across sites (detailed in the “methods for suppressing site effects and data enhancement and ablation study” in the supplemental experimental procedures) during the data preprocessing stage. Additionally, our system design incorporates site information into demographic characteristics by learning and suppressing site differences through a demographic graph. Finally, the adversarial training method and domain migration loss were designed to encourage LGMF-GNN to disregard the interference factors introduced by site effects and focus on the features that are the most relevant to classification. The ablation results of the loss functions are shown in Table S15. Although the LOSO CV performance of the model is lower than that of a 10-fold CV when dealing with data from unfamiliar sites, it still demonstrates good classification performance and generalizability. The experimental results indicate that the methods employed in suppressing site effects are effective and outline a meaningful direction (Table S16). These approaches may serve as a reference for future multi-site research.

### Multimodal fusion

Taking advantage of the high modeling flexibility of GNN, the proposed system successfully achieved a high-quality fusion of the three data modalities: functional, structural, and demographic. As shown in Figure 2B, the addition of the T1 modality provided essential anatomical insights, considerably enhancing the diagnostic accuracy for MDD. Concerning the features of functional modality, we experimented with three fusion methods, and our findings suggest that concatenating correlation features and local network embeddings resulted in the most optimal performance. This is because the concatenation form not only retains the original shallow features but it also incorporates more abstract high-order features extracted by the local ROI GNN network.

We visualized the subject graph constructed based on the three modalities in Figure 5D. In this visualization, “Δ” represents MDD, “○” represents HC, while the various node colors correspond to distinct imaging sites. Additionally, the thickness of the edges represents the strength of inter-individual correlations. Both the HC group and the MDD patient group have nodes with significantly larger degrees (marked in red border) in the functional and structural graphs, representing typical individuals of each group. The sMRI images of the patients corresponding to the eight MDD nodes with the highest degree in the structural subject graph are presented in Figure S7, providing an anatomical characterization of the “typical MDD patients.” In Figure 5D, it can be seen from the graph that edges with larger weights often exist between individuals within the same category, helping to summarize the general characteristics related to a single category. In the demographic graph, nodes within the same site are more densely connected, promoting effective communication of information. In contrast, nodes from different sites are sparsely connected by edges among only a few individuals in each site. These edges (marked in red) build a pathway that facilitates site harmonization.

In conclusion, the graphs of the three modalities perform distinct functions within the network, each providing unique and vital information. The M-Attention block of the subject GNN learns the attention score of each modality during the fusion process. Based on the attention scores, the importance of the three modalities for depression diagnosis in descending order was functional (0.3148), demographic (0.1598), and structural (0.1367). It is worth noting that consistent with our system design, the MC embedding that includes information from all three modalities obtains the highest attention score (0.3887), underscoring its pivotal role.

### Hierarchical graph learning

Based on the hierarchical network, the interaction between local and global perspectives enhances the diagnosis performance of LGMF-GNN. In Figures 2C and 2D, we compared the ROC curve of the 10-fold CV of the system obtained through two-stage training and end-to-end training. The system obtained through end-to-end training achieved a higher AUROC, supporting the effect of the hierarchical structure. Furthermore, upon comparison of the calibration curve and the histogram, we recognized that the end-to-end trained model tends to give a firmer diagnosis, and the predicted probability is better aligned with the prevalence rate. This also demonstrates the collaborative optimization of local and global networks.

The advantages of hierarchical network structure can be summarized into three primary aspects. First, the fine-grained brain region information provided by the local network and the global population information provided by the global network can be collaboratively optimized in the network training stage, providing more comprehensive information for disease diagnosis. Second, the addition of the global network allows for more flexibility in the fusion of multimodal information, which is achieved by combining the three subject graphs of different modalities. Third, the local network enables the learnability of the functional connectivity matrix, thereby improving the interpretability of the system. Meanwhile, the local network also provides better feature vectors for nodes in the global graph. These enhancements hold promise for broader applications beyond MDD diagnosis, potentially extending to the study of other brain diseases or neurological conditions, such as ASD and AD.

### Findings about the underlying mechanisms of depression obtained through interpretation

Through the interpretation of the system, from the perspective of functionality, we discovered abnormal brain functional connections and functional alterations of brain regions in MDD patients. From the perspective of structure, we identified anatomical structural alterations in brain gray matter and white matter as denoted by imaging radiomics characters. In Table S17, we present a detailed literature review that showcases the findings of existing studies on the brain regions identified through interpretable analyses in this paper.

Among the discovered abnormalities, functional abnormalities in the hippocampus, precentral gyrus, and the Rolandic operculum are confirmed by more existing research results. In depression analysis, the hippocampus has always been a key focus. Several studies have shown significant functional and structural abnormalities in patients with depression compared to HCs. Functionally, patients with MDD had increased functional connectivity between the right anterior hippocampus and lingual gyrus, while functional connectivity between the right posterior hippocampus and the right inferior frontal gyrus was diminished.^71^ Moreover, reduced functional connectivity was observed between the right hippocampus and the bilateral medial superior frontal gyrus,^72^ accompanied by a decrease in the node strength of the right hippocampus cornu ammonis 3/4, suggesting weakened brain-wide connectivity.^73^ There are also studies showing that the MDD group exhibited significantly weaker connectivity of the right hippocampal subregional networks with the temporal cortex (extending to the insula) and basal ganglia but showed increased connectivity of the right subiculum to the bilateral lingual gyrus.^74^ Additionally, with the aging of patients with depression, the functional connectivity between the right anterior hippocampus and the left postcentral gyrus tends to increase.^75^ Structurally, hippocampal volume reduction is evident even in the early stages of MDD,^76^^,^^77^^,^^78^ affecting both first-episode patients^79^ and adolescents.^80^ This reduction is also observed in patients with depression and bipolar disorder, affecting both GMV and white matter volume in the right hippocampus.^81^

The changes in the precentral gyrus in depression have also received attention. Compared to HCs, patients with depression have a higher regional cerebral blood flow and greater activation in the right precentral gyrus.^82^^,^^83^ Non-anxious depression patients show reduced functional connectivity between the right precentral gyrus and the right centromedial/laterobasal nucleus.^84^ In the somatic depression group, the regional homogeneity (ReHo) and amplitude of low-frequency fluctuations (ALFF) in the bilateral precentral gyrus are significantly lower than the pure depression group.^85^

The performance of the Rolandic operculum in depression is also noteworthy. In FEDN patients with depression, the functional connectivity of the left Rolandic operculum is enhanced.^86^ In patients with late-onset RECU depression, the ReHO of the left Rolandic operculum gyrus is higher.^87^ Alterations of ALFF in the right Rolandic operculum have also been identified.^88^^,^^89^ In addition, structural alterations in the right Rolandic operculum have been observed to correlate with depressive symptoms in patients suffering from post-stroke depression.^90^

Some of our findings, however, such as the functional alterations in the cerebellum and abnormal functional connectivity between cerebral and cerebellar regions, represent fresh contributions from this study with a large sample size. The role of the cerebellum in depression is increasingly recognized in research. For instance, in terms of function, connectivity studies have revealed diminished cerebral and cerebellar coupling in lobules VI and VIIA/B with prefrontal, posterior parietal, and limbic regions in MDD patients.^91^ Compared with HCs, patients with MDD exhibit significantly increased ReHo values in the right cerebellum crus II.^92^ Structurally, studies have indicated increased gray matter density (GMD) in the right cerebellum VIII of MDD patients, alongside reduced gray GMVs in the right and left cerebellum VIII and X, which correlate with depression severity as measured by BDI scores.^62^ Despite these advances, the exploration of functional connectivity between cerebral and cerebellar regions in depression remains underdeveloped. Our interpretable results underscore the imperative for more in-depth investigation in this direction.

This study’s findings delineate both functional and anatomical abnormalities associated with the cerebellum and cerebral regions, offering valuable contributions to the advancing field of depression research. Our interpretations fortify current research on MDD-related alterations in regions such as the prefrontal cortex and stimulate inquiry into underexplored areas, particularly the role of the cerebellum in the etiology and progression of depression.

### Clinical application and advantages

The LGMF-GNN model introduced in this paper is an end-to-end approach that automates the construction of ROI graphs and subject graphs, enabling an objective and quantitative diagnosis of depression. This innovation holds significant promise for its application in clinical settings. Hospitals can utilize medical records and imaging data from previous patients and healthy individuals to establish ROI graphs and subject graphs, thereby creating a comprehensive graph case library. Upon the arrival of a new patient or research subject, the hospital only needs to collect their MRI scans and basic demographic information, such as gender, age, and education level. The LGMF-GNN can then automatically construct the ROI graph and integrate the new subject into the population graph as a new node. During the model inference phase, the local ROI GNN module performs fine-grained feature extraction and fusion from an individual perspective. Meanwhile, the global subject GNN module facilitates automated message passing and comparison between the new individual and the case nodes in the hospital’s local case library within the latent space. This results in node-level classification, providing an objective and quantitative diagnosis of depression and outputting the probability of illness for the newly included subject. Additionally, the model can identify the most similar past cases from the case library based on the edge weights of the subject graph, offering interpretability.

Clinical practitioners can continue to use their customary diagnostic procedures, but they can now complement their decision-making process with the quantitative probability predictions provided by the LGMF-GNN, as well as the most similar cases selected from the case library. This integration enhances the objectivity and quantification of clinical diagnosis. It is noteworthy that the diagnostic framework based on LGMF-GNN is scalable and shareable. The population graph constructed via LGMF-GNN serves as an anonymized, lightweight case library. In this library, patient data such as fMRI, sMRI, and demographic information are stored, updated, and maintained as nodes with highly abstracted feature vectors. Leveraging federated learning, multiple hospitals across different regions can share and integrate their population graphs, thereby establishing a larger-scale case library. This deep feature-based sharing significantly reduces the risk of patient privacy breaches. With the collaboration of more hospitals and the accumulation of data over time, the population graph will continue to grow and strengthen, offering more precise diagnoses and more reliable insights into the underlying mechanism of MDD.

The lifetime prevalence rate of depression in the population is roughly 1 in 5.^17^^,^^93^^,^^94^^,^^95^ Currently, studies are focusing on training and validating depression diagnostic models on unbalanced datasets and trying to reduce the interference of unbalanced data distribution on model training and generalization.^25^^,^^96^ To assess the performance of LGMF-GNN on unbalanced datasets, we constructed a validation set with an MDD:HC ratio of 1:4 using the bootstrap method. LGMF-GNN was then validated using this set. The experimental setup and results are presented in Table S24 of the supplemental information. Despite the performance degradation in scenarios with unbalanced class distribution, LGMF-GNN still achieved performance comparable to that of the current methods.

### Improving and future work

The local-global GNN architecture, along with the multimodal fusion strategy and the multi-site harmonization techniques, has demonstrated its effectiveness in enhancing diagnostic performance. The conceptual framework we have established is inherently versatile. The strategy of leveraging GNNs for local-global information integration and multimodal data fusion is a promising approach that warrants exploration in other diagnostic domains.

We identify three main limitations of LGMF-GNN and propose potential solutions for future work. First, an important distinction of our study from previous works is the local-global hierarchical graph learning strategy. However, this hierarchical structure also brings new limitations and challenges. In the subject graph, we include all individuals in one graph. With large datasets, the process requires excessive computing and storage resources, posing greater challenges to model training and generalization ability. Employing graph sampling methods like GraphSAGE^97^ and FastGCN^98^ could mitigate these resource demands and enhance the scalability and adaptability of the model to new data. Second, overfitting and site effect mitigation also remain concerns in multi-site data analysis. Although we have already proposed and utilized several methods, such as ComBat, adversarial training, and domain migration loss, to suppress site effects in this paper, the generalization of our system is still affected as the number of data sites increases. To address this issue, more neuroimaging data harmonization and domain migration methods need to be developed. Third, the network failed to provide sufficient interpretability of structural modality features extracted from T1 MRI, and this study did not delve deeply into the changes in the fine and exact anatomical structure of MDD patients compared with HCs. Further refinement of T1 MRI structural feature extraction methods could yield richer anatomical insights for MDD diagnosis.

We are currently collaborating with Beijing Anding Hospital and several other medical centers to clean and process the data of 9 million clinical records from more than 1 million individuals in the Beijing-Tianjin-Hebei mental health big data platform. We are contributing to the development of the China Brain Plan 2030 data platform for mental health disorders. These efforts are expected to facilitate a more comprehensive validation and long-term progress of the proposed system. We will also proactively release our large-scale multimodal datasets, pushing AI-assisted quantitative MDD diagnosis and treatment to a deeper level.

## Experimental procedures

### Data acquisition and preprocessing

#### Dataset Introduction

##### Anding dataset

The dataset acquired from Anding Hospital, China contains the raw fMRI and sMRI scanning results and clinical records of 196 MDD patients and 177 HCs (Table 1). Data collection was conducted using standardized scanners and parameters. The MDD patients include both those with their first episode of depression and those with recurrent depression. The enrollment process for this dataset was carefully controlled. Only MDD patients who had previously been medicated but had refrained from taking medication for at least 2 weeks before data collection were included. Functional images were acquired using the gradient-echo echo-planar imaging sequence, and 200 volumes were collected, with a total scan time of 6 min 40 s. The scan parameters of the functional images were as follows: repetition time (TR) = 2,000 ms; echo time (TE) = 30 ms; number of slices = 33, interlaced axial scanning; slice thickness/gap = 3.5/0.7 mm; flip angle (FA) = 90°; matrix = 64 × 64; field of view (FOV) = 200 × 200 mm^2^; and voxel size = 3.13 × 3.13 × 4.2 mm^3^. T1 structural images were acquired using the magnetization-prepared rapid acquisition gradient echo sequence, and the scan parameters were as follows: TR = 2,530 ms; TE = 1.85 ms; number of sagittal slices = 192; slice thickness = 1 mm; FA = 15°; matrix = 256 × 256; FOV = 256 × 256 mm^2^; and voxel size = 1 × 1 × 1 mm^3^. The diagnoses were established by senior psychiatrists based on the *DSM-IV* criteria for MDD, utilizing the Mini-International Neuropsychiatric Interview (MINI) diagnostic interview as the assessment standard.

##### SRPBS dataset

The SRPBS dataset^99^ is a dataset collected and released by the SRPBS project. Neuroimaging data and demographic information from 457 MDD patients and HC participants were collected at six sites. Each participant underwent a single rs-fMRI session, a structural MRI session, and an optional field map session. Six scanners from three manufacturers (Siemens, Philips, and GE) were used to produce these neuroimaging data. A coherent protocol was designed and used for rs-fMRI. Detailed imaging parameters used at each site for rs-fMRI and T1 structural MRI are summarized in Table S19. Diagnosis of MDD was conducted site specifically. At sites 2, 3, 4, and 5, affiliated with Hiroshima University, diagnoses were made by expert clinicians based on the *DSM-IV-Text Revision* or *DSM-5* criteria, with confirmation via the MINI at the time of study participation. At Kyoto University’s site 6, MDD diagnoses were determined using the Structured Clinical Interview for *DSM* (SCID). Site 8, which is associated with the University of Tokyo, diagnosed psychiatric disorders using *DSM-IV* criteria. HCs were screened for psychiatric disorders using the MINI.

##### REST-meta-MDD dataset

In the REST-meta-MDD project,^19^^,^^100^ 16 research groups from 16 hospitals in China agreed to share data from MDD patients and matched HCs from studies approved by local institutional review boards. The project contributed 1,570 subjects in total—814 MDDs and 756 HCs. The participating groups first preprocessed fMRI images with a standardized protocol at local hospitals and then shared the final fMRI indices and brain matters segmented from T1 sMRI along with demographic (age, sex, and education) and clinical information (e.g., FEDN/RECU, medication usage, illness severity). Detailed imaging parameters used at each site for rs-fMRI and T1 structural MRI are summarized in Table S20. The diagnostic labels for the dataset were derived from the study cohorts as provided by the contributing hospitals.

##### OpenNeuro dataset

The OpenNeuro dataset^101^ involved 21 patients who suffer from DDs and 21 HCs from a single center in Russia, providing raw fMRI and sMRI images as well as demographic information for each subject. The fMRI study was carried out in the International Tomography Center, Novosibirsk, using a 3-T Ingenia scanner (Philips). Functional imaging scans were acquired using the following parameters: TR = 2,500 ms; TE = 35 ms; voxel size 2 × 2 × 5 mm^3^; and fat suppression mode. The reference anatomical image was obtained by the T1w three-dimensional (3D) turbo field echo method with a voxel size of 1 × 1 × 1 mm^3^. The instruction for participants was to lie still with eyes closed for 6 min. The diagnostic criteria for depression are based on the *ICD-10*,^102^ which encompasses mild depression (F32.0), moderate depression (F32.1), and dysthymia (F34.1). Notably, the severity of all three depressive disorders in this dataset is lower than that of MDD (F33.0). The final diagnosis was made at the multidisciplinary clinic Pretor and the International Institute of Psychology and Psychotherapy. None of the patients received antidepressants. Additionally, both groups were equivalent in terms of gender, mean age, and intelligence level (as measured by Raven’s Coloured Progressive Matrices).

Finally, a total of 2,442 participants (1,260 depressive disorder patients vs. 1,182 HCs) from 24 sites (6 sites from SRPBS, 16 sites from REST-meta-MDD, 1 site from Anding, and 1 site from OpenNeuro) were included in our analysis. This study was approved by the institutional review boards at the respective institutions and was conducted in accordance with the ethical standards of the Helsinki Declaration. Informed consent was waived for this retrospective study, as no protected health information was used. Detailed demographic and clinical characteristics of the study population are shown in Table 1. The category and site distribution of the SRPBS and REST-meta-MDD datasets are detailed in Tables S21 and S22.

#### Data Preprocessing

##### Rs-fMRI preprocessing

Each rs-fMRI scan was pre-processed using Data Processing & Analysis for (Resting-State) Brain Imaging (DPABI).^103^ We first discarded the first five time points and corrected all volume slices for different signal acquisition times. Then, the time series of images for each subject were realigned. After realignment, individual structural images were co-registered to the mean functional image. To remove the nuisance signals, the Friston 24-parameter model and global signal regression^104^ were referenced to regress out head motion effects from the realigned data. The DARTEL tool was used to transform the functional data from the individual native space to the MNI space. Further, spatial smoothing and temporal filtering were performed to reduce noises.

##### T1w MRI preprocessing

For the SRPBS, Anding and OpenNeuro datasets with raw whole brain T1 sMRI provided, the skull-stripped T1w MRI can be obtained after the rs-fMRI preprocessing with the DPABI tool. The T1 image was then affine registered to the Montreal Neurological Institute (MNI) atlas by FSL’s Functional Magnetic Resonance Imaging of the Brain’s Linear Image Registration Tool tool,^105^ which has a voxel size of 1 × 1 × 1 mm^3^. To remove the black boundaries, the dimension of the 3D brain volume is center cropped into a size of 140 × 170 × 140 mm^3^. Additionally, GMV and white matter volume images were obtained for the three datasets through voxel-based morphometry (VBM) using the SPM tools in the DPABI toolbox. VBM provides an automated quantitative analysis of the distribution of gray and white matter to detect differences in brain tissue concentration for each voxel (e.g., GMD). To include voxel-wise volume changes, the GMD is then modulated by multiplication with the Jacobian determinant (JD). The JD is derived from the non-linear deformation field needed to transform each subject brain to a given template brain. The modulated GMD is then multiplied with the voxel volumes and is interpreted as GMV.^106^ GMV and white matter volume in SPM can provide insight into the changes and differences in brain anatomy and can be used to identify biomarkers of brain diseases.

##### Data exclusion and quality control

Stringent data exclusion and quality control measures were implemented to ensure the integrity of the datasets used in this study. Criteria for exclusion included age, image quality, head motion, spatial correlation, site-specific characteristics, and duplicate entries. The specific criteria and the number of subjects excluded based on these criteria for each dataset are detailed in the “data cleaning and quality control” section of the supplemental experimental procedures and Figures S1–S4). These measures were crucial for minimizing biases and ensuring the high quality of the analyzed data.

#### Feature Extraction

##### rs-fMRI feature extraction

Previous research has confirmed the correlation between blood flow levels in the brain and neural activity in the brain.^107^ Therefore, the BOLD signal series reflects the changes in the activity levels of each ROI over time. It is one of the important indicators for studying brain function. We chose the widely used revised AAL^108^ and CC200^109^ brain atlases for ROI definition. The AAL brain atlas is a commonly used brain atlas that divides the human brain into 116 regions, including 90 regions in the brain and 26 regions in the cerebellum, and assigns a label to each region to help researchers identify and distinguish different brain regions. The CC200 brain atlas is a more complex brain atlas that divides the human brain into 200 regions. Both brain atlases are derived by analyzing and processing a large amount of brain imaging data and using MRI technology to measure the connectivity and organization of each region and have been used to study neurological and psychiatric disorders such as AD, Parkinson disease, and ASDs.

Based on the ROI defined by the AAL and CC200 brain atlases, the average BOLD signal within each ROI was extracted using the DPABI tool. Since the rs-fMRI data in the dataset were collected based on different scanners and protocols, the length of the extracted ROI BOLD signals was different. To align the data for subsequent extraction of temporal features, the extracted ROI BOLD signals were truncated using a sliding window with a certain window width and step size to obtain a time series of equal length. The Fisher Z-transform has been applied to standardize the time series of each ROI. We measured the functional connectivity between brain regions by the Pearson correlation of ROI BOLD signals to form an initial functional connectivity matrix.

##### T1-sMRI feature extraction

For the SRPBS dataset, we used a global-local transformer to extract anatomical features from the preprocessed image patches since it provided the whole raw T1-sMRI images. The optimization task is designed as brain age prediction, which correlates with anatomical structure and avoids information leakage for MDD diagnosis. The feature map $m_{GLT}\in R^{n\times d}$ before the fully connected layer is extracted as each subject’s anatomical feature vector, where n is the number of ROIs and d is the length of the feature vector for each ROI. However, the REST-meta-MDD dataset does not provide raw T1-sMRI data, but only contains structural image data of segmented gray matter, white matter, and cerebrospinal fluid volume images. Previous neuroimaging studies have shown that the GMV and white matter volume are correlated with depression.^110^ Therefore, the radiomics features were extracted from the whole four datasets using PyRadiomics.^111^ We extracted a 1,209-dimensional radiomics feature vector from each subject’s GMV and white matter volume images respectively, yielding a 2,418-dimensional anatomical feature vector for each subject. Every 1,209-dimensional radiomics feature consists of 93 radiomics metrics extracted from the original images and 12 derived images (e.g., Laplacian of Gaussian filtering and wavelet transform). For details on feature extractor settings, see Figure S5.

##### Controlling for nuisance variables

Site effects^112^ are important confounding factors that cannot be ignored in MRI-based imaging studies. The datasets used in this study are collected from multiple institutions using different scanners and protocols. The SRPBS dataset was collected from six different sites using different protocols, and the REST-meta-MDD dataset involves 16 cohorts from different hospitals, leading to series site effects. The nuisance variables caused by the heterogeneous scanner manufacturers, different acquisition protocols, and instructions to participants are difficult to remove even with a unified image preprocessing pipeline. To reduce the effects of unwanted nuisance variables, we applied the ComBat harmonization,^113^ a multivariate mixed linear regression model, on rs-fMRI-derived connectivity measures (see the “methods for suppressing site effects and data enhancement and ablation study” section in the supplemental experimental procedures for specific implementation details). According to Figure S6, this method can successfully regress out site effects while avoiding over-correction on important biological variance.

### Model architecture and training

An overview of the proposed method is illustrated in Figure 1. The model mainly consists of a local ROI GNN and a global subject GNN. Each subject’s ROI signals and initial functional connectivity matrix were fed into the ROI GNN to generate an embedding and a refined functional connection matrix. Then, subject graphs with different modalities were constructed based on the brain embedding generated by ROI GNN, with different atlases for each subject, T1 features, and the non-imaging data (e.g., age, sex, data acquisition site, years of education), respectively. The global subject GNN is performed on this graph for node classification to obtain the disease state prediction for each subject. The specific structure of the network and the method of graph construction are described in detail in the following sections.

#### Local ROI GNN

##### Network structure

As shown in Figure 1B, the local ROI GNN mainly consists of three components: (1) a GRU regional time series encoder, (2) a graph generator based on regional time series embedding, and (3) a GNN predictor to generate the graph level embedding and the local prediction.

##### GRU regional time series encoder

BOLD signal has been widely used in brain disease diagnosis and research. However, traditional BOLD signal encoding methods such as Pearson correlation or partial correlation failed to capture the temporal order. As a variant of the widely used time series signal encoder LSTM (long short-term memory), bidirectional GRU (bi-GRU) has a simpler structure and is able to obtain complete time series information by calculating and combining forward and backward signals. Therefore, we chose bi-GRU as the time series encoder to achieve lightweight frameworks and fit the low temporal resolution of the BOLD signal. Specifically, for an input BOLD time series $X\in R^{n\times t}$ of a subject, where n is the number of ROI and t is the length of the time series, the GRU encoder generates a regional embedding for each ROI, $h_{e}=Encoder\left(x\right),h_{e}\in R^{n\times d}$, where d is the output size of the GRU encoder.

##### Graph generator

The graph generator is designed to generate a flexible graph structure based on the encoded time series feature $h_{e}$. Since the graph structure is expressed by the adjacent matrix A, a learnable graph structure can be generated as $A=softmax\left(h_{e}h_{e}^{T}\right)\cdot n$. We enhanced the edge weight with the number of ROIs (nodes) to avoid too-small variance of the edge weights and ensure the sparsity of the graph. The learnable graph structure A can be regularized by the downstream prediction task.

##### GNN predictor

For the graph predictor, we adopted a three-layer GCN model, and the concatenation aggregation with attention mechanism was employed to transform and propagate node features and structure information on the constructed graph. The node feature $F_{p}$ of node p was initialized as a vector of Pearson correlation coefficients to all ROIs. Specifically, the k-th GCN layer is defined referring to GCN proposed by Kipf and Welling^114^ as:

(Equation 1) $$\begin{array}{c} H_{l}^{k}=\sigma \left(GCN\left(H_{l}^{k-1},A\right)\right)=\sigma \left(D^{-\frac{1}{2}}AD^{-\frac{1}{2}}H_{l}^{k-1}W^{k}\right) \end{array}$$

where D is the diagonal matrix, A is the adjacent matrix derived from the graph generator, and $W^{t}$ is a trainable weight matrix of the k-th layer, which is a two-layer MLP in our implementation. $H_{l}^{0}=F$. The final embedding of the whole graph is calculated through concatenating node embedding weighted by the attention score.

(Equation 2) $$\begin{array}{c} H_{\mathrm{lG}}=concat\left(a_{ROI}H_{l}^{k}\right) \end{array}$$

(Equation 3) $$\begin{array}{c} a_{ROI}=Attention\left(m\right)=softmax\left(\Sigma_{j=0}^{n}m_{ij}\right)\cdot n \end{array}$$

Finally, another MLP layer is employed for class prediction $\hat{y}$

(Equation 4) $$\begin{array}{c} \hat{y}=MLP\left(BatchNorm1D\left(H_{\mathrm{lG}}\right)\right)\text{.} \end{array}$$

#### Global subject GNN

##### Graph construction

In subject graphs, an individual is modeled as a node in the graph, and the edges between nodes reflect the connections between individuals. Three types of subject graphs are constructed based on medical imaging and non-imaging data, as shown in Figure 1B. The first type is the functional subject graph, denoted as $G_{f}=\left(V_{f},E_{f},W_{f}\right)$. Similarities between graph embeddings generated by local GNN of two subjects are extracted to generate this k-nearest graph, which models the correlation of functional brain activity among different individuals. The second type is the anatomical subject graph, denoted as $G_{s}=\left(V_{s},E_{s},W_{s}\right)$. This is a k-nearest graph based on the similarities of the anatomical structure of the brain reflected by T1 sMRI features. The third type is the demographic subject graph, denoted as $G_{d}=\left(V_{d},E_{d},W_{d}\right)$. In this graph, the relationship of subjects is evaluated by non-image discrete data such as age and sex.

For $G_{f}$, the calculated functional feature embedding $H_{G}$ is first taken as the feature of the corresponding node. Then, the edge weight $W_{f}$ based on the cosine similarity of node features is calculated, and K pairs of nodes with top K edge weights are selected to construct a k-nearest neighbors (KNN) graph. $G_{s}$ is constructed in a similar way, with the feature vector extracted from T1w MRI as the node feature. For the $G_{d}$, inspired by EV-GCN,^50^ a PAE is used to determine the weights between subjects $v_{si}$ and $v_{sj}$ based on additional information provided by non-imaging data vector η. $W_{d}\left(i,j\right)$ is defined as $W_{d}\left(i,j\right)=\frac{\cos\left(MLP\left(\eta_{i}\right),MLP\left(\eta_{j}\right)\right)+1}{2}$, where cos denotes the cosine similarity between two input vectors.

##### Network structure

The global subject GNN is designed to be a multimodal fusion network with three MS-GCN blocks to extract the unique feature of each data modality, an MC-GCN block to fuse common information shared by all data modalities and a multimodal attention block (M-Attention) to achieve efficient information integration.

##### Snowball GCN

Li et al.^115^ have clarified that GCN is essentially a type of Laplacian smoothing, which computes the new features of a vertex as the weighted average of itself and its neighbors. A k-layer GCN block will aggregate information from k-hop neighbors. However, stacking multiple layers of GCN will cause the oversmoothing problem, in which case, the features of vertices from different clusters are mixed and indistinguishable. Due to these limitations, most GCNs are no deeper than four layers,^116^ which makes it hard to aggregate information comprehensively on the subject graph with more than 2,000 nodes. To mitigate the impact of this problem, the present study used the snowball GCN block proposed by Zhao et al.^117^ as the basic GCN block. The main idea of this block is connecting multi-scale feature information in the hidden layer with a densely connected graph network to obtain richer representations of each node. The structure of snowball GCN is as follows:

(Equation 5) $$\begin{array}{c} H_{g}^{0}=X\text{,} \end{array}$$

(Equation 6) $$\begin{array}{c} H_{l+1}=\mathrm{Tanh}\left(L\left[H_{g}^{0},H_{g}^{1},\ldots ,H_{g}^{l}\right]W_{l}\right),l=0,1,2,\ldots ,n-1 \end{array}\text{,}$$

(Equation 7) $$\begin{array}{c} C=\mathrm{Tanh}\left(\left[H_{g}^{0},H_{g}^{1},\ldots ,H_{g}^{n}\right]W_{n}\right)\text{,} \end{array}$$

(Equation 8) $$\begin{array}{c} H_{gG}=normalize\left(L^{p}CW_{c}\right) \end{array}$$

where n is the number of snowball GCN layers, $W_{l},W_{n},W_{c}$ are trainable matrixes, $H_{g}^{0},H_{g}^{1},\ldots ,H_{g}^{l}$ are extracted features, $H_{gG}$ is the global subject-graph embedding of one specific modality, and p∈{0,1}. When $p=0,L^{p}=I$; when $p=1,L^{p}={L=D}^{-\frac{1}{2}}AD^{-\frac{1}{2}}$, which means that we project C back onto the Fourier basis. This is necessary when graph structure encodes a great deal of information.

##### MS-GCN block

The MS-GCN is applied to extract MS embeddings, which is defined as follows:

(Equation 9) $$\begin{array}{c} H_{s}^{f}=SnowballGCN\left(X^{f},A^{f}\right)=SnowballGCN\left(H_{\mathrm{lG}},A^{f}\right) \end{array}\text{,}$$

(Equation 10) $$\begin{array}{c} H_{s}^{s}=SnowballGCN\left(X^{s},A^{s}\right)\text{,} \end{array}$$

(Equation 11) $$\begin{array}{c} H_{s}^{d}=SnowballGCN\left(X^{d},A^{p}\right)\text{,} \end{array}$$

where $X^{f},X^{s},X^{d}$ are node features for rs-fMRI, sMRI, and demographic modalities, respectively, and $H_{s}^{f},H_{s}^{s},H_{s}^{d}$ are the MS representations. The weights of the three snowball GCN networks are independent of one another, making it possible to extract unique features more effectively.

##### MC-GCN block

Although each modality and brain atlas has a specific data structure and semantic information, it is impossible to completely decouple the different types of data. For the same task, data from different modalities tend to contain some common information. Extracting this shared information not only helps to summarize high-quality features for solving the final problem but it also avoids redundancy in multimodal information fusion. To achieve this target, we add MC-GCN to the model, which shares the weight matrix between different modes when performing snowball GCN.

(Equation 12) $$H_{c}^{f}=SnowballGCN\left(X^{f},A^{f}\right)=SnowballGCN\left(H_{\mathrm{lG}},A^{f}\right)=normalize\left(L^{fp}C^{f}W_{cs}\right)\text{,}$$

(Equation 13) $$\begin{array}{c} H_{c}^{s}=SnowballGCN\left(X^{s},A^{s}\right)=normalize\left(L^{sp}C^{s}W_{cs}\right)\text{,} \end{array}$$

(Equation 14) $$\begin{array}{c} H_{c}^{d}=SnowballGCN\left(X^{d},A^{d}\right)=normalize\left(L^{dp}C^{d}W_{cs}\right)\text{,} \end{array}$$

where $H_{c}^{f},H_{c}^{s},H_{c}^{d}$ are the MC representations for rs-fMRI, sMRI and demographic modalities, respectively, and $W_{cs}$ is the shared trainable matrix. By sharing weights in this way, MC features can be filtered out. In this experiment, we selected three modalities and two brain atlases for the experiment, and the resulting common embeddings are denoted as $H_{c}^{fAAL},H_{c}^{fCC200},H_{c}^{s},and\;H_{c}^{d}$. The final common embedding is obtained by the weighted sum of the four $H_{c}={\alpha H}_{c}^{fAAL}+\beta H_{c}^{fCC200}+\gamma H_{c}^{s}+\epsilon H_{c}^{d}$, where α,β,γ, and ϵ are hyperparameters measuring the importance of each MC embedding. In the implementation, we set α,β,γ, and ϵ to be equal and sum to 1 to pay equal attention to all modalities.

##### M-attention block

The contribution of different modes of information to the final diagnosis varies with the target disease. To apply more attention to the effective modes and make the less important modes only play an auxiliary role, we use the attention mechanism on four specific embeddings, $H_{s}^{fAAL},H_{s}^{fCC200},H_{s}^{s},{and\;H}_{s}^{d}\text{,}$ and one common embedding, $H_{c}$:

(Equation 15) $$\begin{array}{c} a_{fAAL},a_{fCC200},a_{s},a_{p},a_{c}=Attention\left(H_{s}^{fAAL},H_{s}^{fCC200},H_{s}^{s},H_{s}^{d},H_{c}\right) \end{array}$$

(Equation 16) $$\begin{array}{c} e_{i}=\mathrm{Tanh}\left(W_{ai}H_{S}^{i}+b_{i}\right) \end{array}$$

(Equation 17) $$a_{i}=\frac{\exp\left(e_{i}\right)}{\exp\left(e_{fAAL}\right)+\exp\left(e_{fCC200}\right)+\exp\left(e_{s}\right)+\exp\left(e_{d}\right)+\exp\left(e_{c}\right)},\;i\;\in \{f_{AAL},f_{CC200},s,d,c\}$$

After obtaining the attention score, the final embedding can be calculated by combining the representation and the weight as follows:

(Equation 18) $$\begin{array}{c} H=a_{fAAL}H_{s}^{fAAL}+a_{fCC200}H_{s}^{fCC200}+a_{s}H_{s}^{s}+a_{p}H_{s}^{d}+a_{c}H_{C} \end{array}$$

#### Objective function

The overall objective function of LGMF-GNN contains three kinds of loss terms as follows:

(Equation 19) $$\begin{array}{c} L=L_{cls}+L_{specific}+L_{common}+L_{domain} \end{array}$$

##### Classification loss

We chose cross-entropy loss as the classification loss function. It should be noted that the proposed model can make predictions in both local and global stages, so the classification loss is a weighted sum of the two parts:

(Equation 20) $$\begin{array}{c} L_{cls}=L_{cls_{global}}+\lambda L_{cls_{local}} \end{array}\text{,}$$

where λ is the weight hyperparameter of the local classification loss. In our implementation, we set λ to 0.2 to ensure that the global classification loss plays a dominant role in the overall optimization process.

##### Modality independence loss

As mentioned above in the network structure section, we hope to extract features that are independent and shared between modalities separately. However, our extractor is based on the same structure. To facilitate the separation of MS and MC embeddings, we impose the modality-independence constraint on them. Specifically, the Hilbert-Schmidt independence criterion^118^ is used to measure the dissimilarity of the two sets of embedding distributions:

(Equation 21) $$\begin{array}{c} HSIC\left(H_{s},H_{c}\right)={\left(m-1\right)}^{-2}tr\left(K_{c}RK_{s}R\right) \end{array}\text{,}$$

(Equation 22) $$\begin{array}{c} K\left(H^{i},H^{j}\right)=<\phi \left(H^{i}\right),\phi \left(H^{j}\right)> \end{array}\text{,}$$

(Equation 23) $$\begin{array}{c} R=I-\frac{1}{m}\;ee^{T} \end{array}\text{,}$$

where I is the unit matrix, e is a column vector with all values of 1, ϕ is a kernel function that maps the input to reproducing kernel Hilbert space, and <> denotes the inner product of the inputs. In this way, we obtain the loss function:

(Equation 24) $$L_{specific}=HSIC\left(H_{s}^{fAAL},H_{c}\right)+HSIC\left(H_{s}^{fCC200},H_{c}\right)+HSIC\left(H_{s}^{s},H_{c}\right)+HSIC\left(H_{s}^{d},H_{c}\right)\text{.}$$

##### Modality similarity loss

To extract the modality-shared features, we designed the MC-GCN block, which is a snowball GCN with shared weights. Apart from the model structure design, the modality similarity constraint is proposed to further encourage the similarity of MC embeddings across modalities. Specifically, the $L_{2}$-norm is calculated between normalized embeddings of different modalities:

(Equation 25) $$\begin{array}{c} N_{c}=H_{c}\cdot H_{c}^{T} \end{array}$$

(Equation 26) $$\begin{array}{c} L_{common}={\left\|N_{c}^{f}-N_{c}^{p}\right\|}^{2}+{\left\|N_{c}^{f}-N_{c}^{s}\right\|}^{2}+{\left\|N_{c}^{d}-N_{c}^{s}\right\|}^{2} \end{array}$$

##### Domain migration loss

To alleviate site effects during the training stage, the domain adversarial training strategy was used, and a domain migration loss was designed to reduce the difference between the generated embeddings in the source and target domains. The adversarial training is achieved with a site classifier and a gradient inversion layer, and domain migration loss is designed based on the central moment discrepancy (CMD) distance. Thus, the total domain loss is defined as:

$$L_{domain}=L_{site}+L_{CMD}$$

where $L_{site}$ is the cross-entropy loss between the site prediction result of the site classifier and the real site label. $L_{CMD}$ is the CMD distance between the source and target domain. More detailed information can be found in the “domain adversarial training” and “domain migration loss” sections of the supplemental experimental procedures.

#### Implementation details

The proposed LGMF-GNN was implemented in Python 3.7 and PyTorch 1.12.1 on an NVIDIA TITAN RTX GPU. The Adam optimizer was used with a learning rate of 1e−2 and halved every 100 epochs. We allowed the model to run for at most 800 epochs for all experiments. Based on the experimental results and analysis presented in Table S23 and “the impact of brain atlases on model performance**”** section of the supplemental experimental procedures, we ultimately used the AAL brain atlas for brain ROI definition, model training, and interpretable research. The parameters α,β,γ, and ϵ, which govern the modality weights in the final common embedding $H_{c}$, were assigned equal values to sum to 1, ensuring balanced consideration across all modalities. The weight hyperparameter λ of the local classification loss was fixed at 0.2 to prioritize the global classification loss in the overall optimization. Grid searches were performed to determine the hyperparameter k, representing the number of neighbors in the KNN population graph construction, and n, indicating the number of layers in the snowball GCN. The optimal values for these parameters were found to be k=10 and n=9. A comprehensive discussion and detailed results concerning hyperparameter selection are provided in Figures S8 and S9 and the “hyperparameter selection” section of the supplemental experimental procedures.

### Methods for interpretable research on the model

#### Interpretation of the functional modality

Interpretability experiments for the functional modality were conducted on the combined dataset from the SRPBS and REST-meta-MDD studies, which provided a sufficient sample size to ensure the statistical power of our findings. The local ROI GNN is designed to construct a learnable adjacency matrix by capturing the deep and dynamic temporal feature correlations of the ROI BOLD signals. This matrix was optimized for both local and global tasks. Consequently, the trained LGMF-GNN can predict an adjacency matrix based on the input fMRI ROI signals. This predicted matrix not only encompasses deeper temporal features but it also integrates demographic and anatomical constraints, surpassing traditional matrices that rely solely on Pearson correlation coefficients. By analyzing these learned adjacency matrices, or functional connectivity patterns, from the HC and MDD groups, we can elucidate the underlying mechanisms of the functional states of MDD.

During model training, we conducted 10-fold CV experiments on the SRPBS and REST-meta-MDD datasets, which means each sample in the dataset was added to the training set nine times. We used the model obtained from each fold to perform inference on the training set corresponding to that fold and saved the functional connectivity matrix obtained by local ROI GNN during inference. In this way, for each sample in the dataset, we obtained nine learned functional connectivity matrices. These functional connectivity matrices are subsequently averaged to produce a single functional connectivity matrix for each sample. Then, we utilized the diagnostic labels indicating whether each sample was diagnosed with MDD to split the dataset into two groups: HC and MDD. We separately averaged the learned functional connectivity matrices for all samples within each group. This resulted in the average functional connectivity matrices for the HC and MDD groups, as depicted in Figure 5A. The differential functional connectivity matrix (MDD-HC) is obtained by subtracting these two matrices.

The interpretability of the variation of functional connectivity between HC and MDD was based on the differential functional connectivity matrix. Specifically, we extracted the row and column coordinates corresponding to the five maximum and minimum values in the matrix. The functional connections between brain regions indexed by these coordinates represent the enhanced and weakened FCs in MDD compared to HC. The interpretability of the most contributive brain regions was based on the differential functional connectivity matrix after taking absolute values. By summing the rows of this matrix, we obtained the degree of each node, with a higher degree indicating a greater difference in functional connections associated with that brain region between the MDD and HC groups. Thus, we selected the 10 nodes with the highest degrees as the most diagnostically informative brain regions.

#### Interpretation of the anatomical modality

For anatomical modality, the analysis was conducted on the SRPBS dataset. Following the steps outlined in the data processing section, we obtained a 1,209-dimensional feature vector as input for the anatomical modality, encompassing 93 radiomic characters that characterize the anatomical properties of the brain tissue. To identify the key radiomic characters contributing to the diagnosis of MDD, we employed a feature-masking strategy to assess the impact of a specific radiomic character on model performance. Specifically, using the model trained with 10-fold CV for inference, we masked, or set to zero, the elements corresponding to the vector position of each radiomic feature during the inference process. This yielded the average performance across the 10-fold CVs after masking. The performance of the model was measured by the AUROC. By comparing the average inference AUROC of 10-fold CV results obtained from LGMF-GNN with unmasked inputs to those with masked inputs, we evaluated the performance loss of each radiomic character. We identified the top five radiomic characters with the greatest performance loss as the most contributive to diagnosis.

## Resource availability

### Lead contact

Cheng Jin is the lead contact for this study and can be reached at chengjin520@sjtu.edu.cn.

### Materials availability

This study did not generate new unique reagents.

### Data and code availability

• All of the code associated with this study is publicly available online at https://github.com/ATP-BME/LGMF-GNN and has been archived at a Figshare repository^119^ at https://doi.org/10.6084/m9.figshare.27107440.
• The SRPBS dataset is available at https://doi.org/10.7303/syn22317081.
• The REST-meta-MDD dataset is available at https://doi.org/10.57760/sciencedb.o00115.00013.
• Lists of members and their affiliations for the DIRECT Consortium appear in the supplemental information as Table S25.
• The OpenNeuro dataset is available at https://openneuro.org/datasets/ds002748.

## Acknowledgments

This study was funded by the Sci-Tech Innovation 2030 - Major Project of Brain Science and Brain-inspired Intelligence Technology (grant no. 2021ZD0200600), the 10.13039/501100012166National Key Research and Development Program of China (grant no. 2022YFB4702702) and the Shanghai Committee of Science and Technology, China (grant no. 21DZ1100301). We would also like to acknowledge the data curators and providers who meticulously organized and maintained the dataset. Their dedication in ensuring the accessibility and quality of the data has been paramount to our research.

## Author contributions

C.J., G.W., L.Z., and Y.W. contributed to the conception of the study and offered method guidance. S.L. designed the algorithm, developed the structure, performed the experiment, and wrote the first and final draft of this manuscript. J.Z., X. Zhu, and X. Zhou performed the data preprocessing and offered clinical guidance. Y.Z. and S.Z. helped perform the analysis with constructive discussions. Z.Y. and X.C. contributed help in data preprocessing. Z.W. contributed significantly to manuscript preparation. R.W., Y.Y., and X.F. provided valuable suggestions for the experiments. All the authors reviewed and approved the final manuscript.

## Declaration of interests

The authors declare no competing interests.

## Supplemental information

Document S1. Tables S1–S25, Figures S1–S9, and supplemental experimental procedures

Document S2. Article plus supplemental information
