# Supplementary material for: An objective quantitative diagnosis of depression using a local-to-global multimodal fusion graph neural network
Source: Patterns (N Y). 2024 Nov 4;5(12):101081. doi: 10.1016/j.patter.2024.101081 (PMC11701859; doi:10.1016/j.patter.2024.101081)
Supplement: Document S1. Tables S1–S25, Figures S1–S9, and supplemental experimental procedures [file mmc1.pdf]

Patterns, Volume *5*

Supplemental information

An objective quantitative diagnosis of depression using a local-to-global multimodal fusion graph neural network

Shuyu Liu, Jingjing Zhou, Xuequan Zhu, Ya Zhang, Xinzhu Zhou, Shaoting Zhang, Zhi Yang, Ziji Wang, Ruoxi Wang, Yizhe Yuan, Xin Fang, Xiongying Chen, DIRECT Consortium, Yanfeng Wang, Ling Zhang, Gang Wang, and Cheng Jin

Supplemental Items

Table S1. Cross validation result of LGMF-GNN on SRPBS dataset

		ACC	AUC	Precision	Recall	F1-Score
10-Fold CV	2-Stage	78.22±2.38	78.85±5.50	79.37±9.19	74.05±8.28	75.91±4.27
	End-to-End	78.75±4.55	80.64±5.74	76.80±7.93	82.00±5.79	78.96±4.88
LOSO CV	2-Stage	71.43±5.68	71.72±5.17	72.08±8.12	73.00±5.67	72.30±5.59
	End-to-End	72.64±4.77	73.71±4.12	72.45±5.72	74.35±6.49	73.33±5.70

Table S2. Cross validation result of LGMF-GNN on REST-meta-MDD dataset

	ACC	AUC	Precision	Recall	F1-score
10-Fold CV	71.34±1.50	73.67±2.67	72.20±4.61	73.52±6.26	72.56±2.06
LOSO CV	74.53±9.70	73.46±10.89	74.71±9.98	77.21±11.90	75.57±9.45

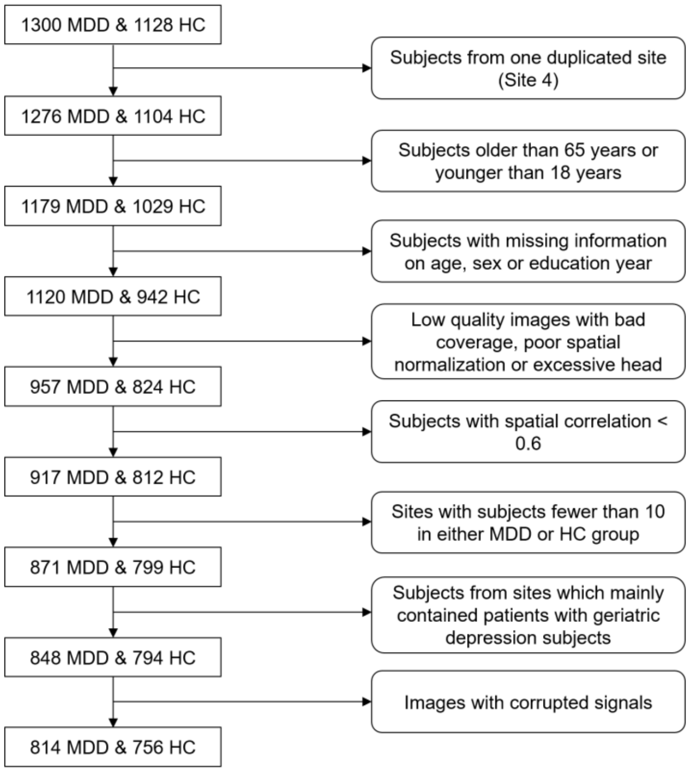

Figure S1. The flowchart of data selection on REST-meta-MDD dataset

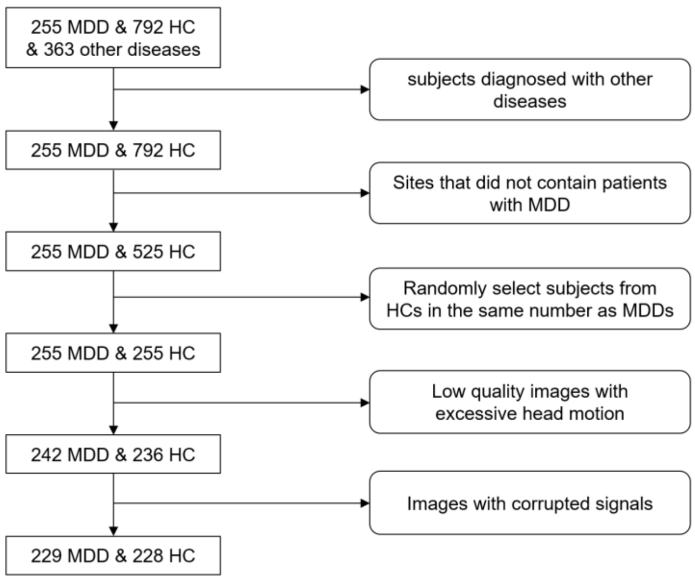

Figure S2. The flowchart of data selection on SRPBS dataset

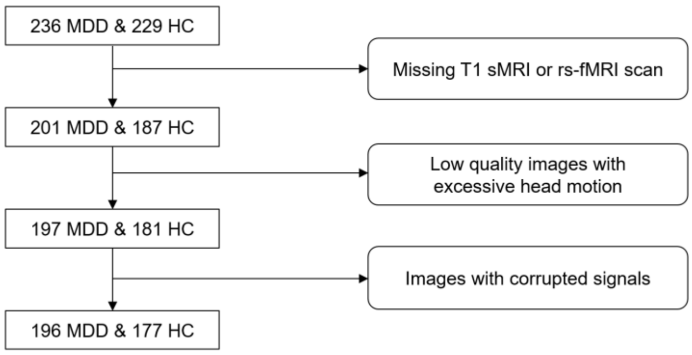

Figure S3. The flowchart of data selection on Anding dataset

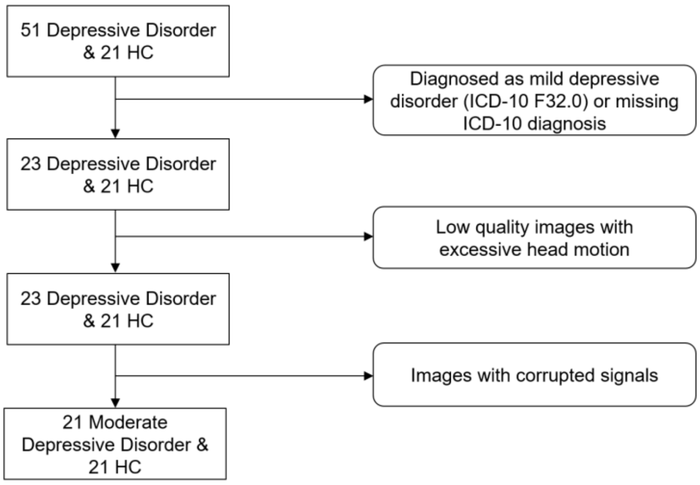

Figure S4. The flowchart of data selection on Openneuro dataset

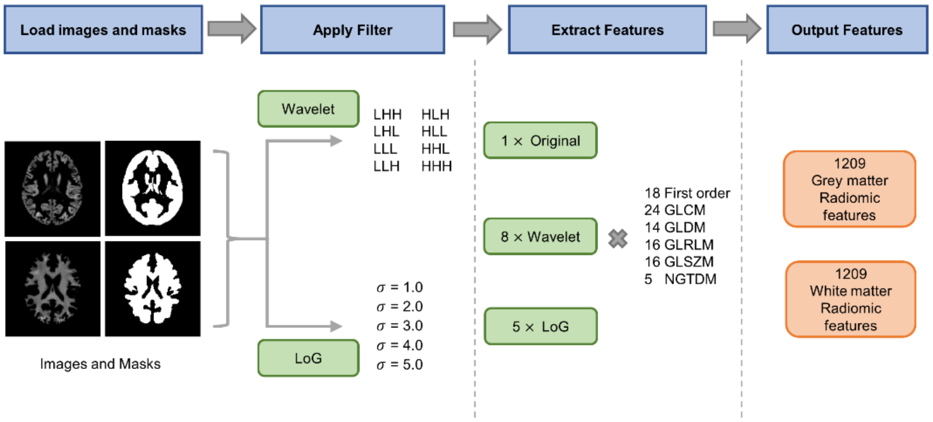

Figure S5. Overview of the feature extraction process using PyRadiomics. Grey matter or white matter volumes and segmentation masks are loaded into PyRadiomics first. Next, Laplacian of Gaussian filters (Sigma=2.0,3.0,4.0,5.0) and Wavelet filters were applied to the original image, resulting in 12 derived images. Subsequently, radiomic features were extracted from the 13 images, forming two 1209-dimensional feature vectors to represent the anatomical features in grey and white matter.

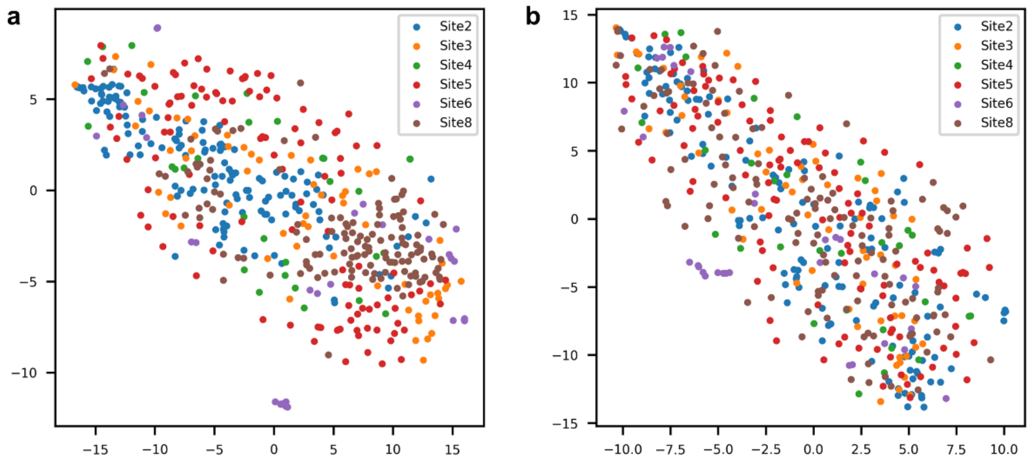

Figure S6. t-SNE plots of SRPBS dataset before and after Combat harmonization

Table S3. Comparison of 10-fold CV results on single-site and multi-site scenarios

	ACC	AUC	Precision	Recall	F1-Score
S20 (1-site)	91.92±4.67	93.41±4.56	89.32±4.56	92.47±7.84	90.62±4.54
SRPBS (6-site)	69.02±3.57	66.33±4.76	72.88±4.55	62.93±2.84	65.94±4.53

Table S4. Category distribution of the FEDN vs HC subset of REST-meta-MDD dataset by site

Site	FEDN	HC	Total
8	28	28	56
9	22	22	44
14	61	32	93
20	97	126	223
23	11	11	22
Total	219	219	438

Table S5. Category distribution of the RECU vs HC subset of REST-meta-MDD dataset by site

Site	RECU	HC	Total
7	11	11	22
9	26	26	52
11	18	17	35
19	12	12	24
20	46	58	104
21	76	65	141
Total	189	189	378

Table S6. Category distribution of the FEDN vs RECU subset of REST-meta-MDD dataset by site

Site	FEDN	RECU	Total
9	22	26	48
20	97	46	143
Total	119	72	191

Table S7. Subtype classification on the REST-meta-MDD dataset

	ACC	AUC	Precision	Recall	F1-Score
FEDN vs. HC	75.13±3.16	76.73±4.57	76.65±8.80	73.12±6.50	74.31±4.08
RECU vs. HC	74.05±4.34	72.28±7.49	73.24±8.44	71.91±6.36	71.98±3.45
FEDN vs. RECU	76.92±8.94	68.89±15.63	78.98±14.81	85.95±11.68	81.35±10.20

Table S8. Comparison with other GNN methods on the Anding dataset

	ACC	AUC	Precision	Recall	F1-Score
**GCN**	54.69	57.75	67.53	26.53	38.10
**GIN**	52.54	53.65	57.60	36.74	44.86
**GAT**	56.84	56.93	64.00	40.82	49.84
**BrainGNN**	55.23	48.89	54.33	**92.86**	68.55
**EV-GCN**	57.11	57.74	60.98	51.02	55.56
**LG-GNN**	57.91	59.92	60.54	57.14	58.79
**ContrastPool**	54.17	54.49	53.06	85.25	65.41
**pRGAT**	54.42	54.14	56.25	59.69	57.92
**MAMF-GCN**	56.84	54.88	59.89	54.08	56.84
**SFGL**	51.21	48.34	53.27	58.16	55.61
**CI-GNN**	57.50	58.84	58.79	59.69	59.24
**IBGNN**	52.01	48.64	52.51	90.82	66.54
**LGMF-GNN**	**69.97**	**72.91**	**71.21**	71.94	**71.57**

Table S9. Comparison with other GNN methods on the Openneuro dataset

	ACC	AUC	Precision	Recall	F1-Score
**GCN**	59.52	55.33	59.09	61.91	60.47
**GIN**	61.91	51.47	66.67	47.62	55.56
**GAT**	66.67	60.32	**68.42**	61.91	65.00
**BrainGNN**	57.14	48.75	63.64	33.33	43.75
**EV-GCN**	66.67	65.08	65.22	71.43	68.18
**LG-GNN**	**69.05**	69.84	68.18	71.43	69.77
**ContrastPool**	65.00	62.66	65.22	71.43	68.18
**pRGAT**	54.76	54.76	60.00	28.57	38.71
**MAMF-GCN**	66.67	64.17	**68.42**	61.91	65.00
**SFGL**	54.76	46.49	54.17	61.91	57.78
**CI-GNN**	71.67	61.68	70.00	66.67	68.29
**IBGNN**	57.14	55.67	55.17	76.19	64.00
**LGMF-GNN**	**69.05**	**70.30**	66.67	**76.19**	**71.11**

Table S10.1. Hyperparameter setting of GCN

Hyperparameter name	Value	Description
num_layer	3	Number of GNN message passing layers
emb_dim	64	Dimensionality of hidden units in GNNs
lr	1e-4	Learning rate of optimizer
batch_size	32	Input batch size for training
drop_ratio	0.6	Dropout ratio
stepsize	50	Scheduler step size
gamma	0.6	Scheduler shrinking rate

Table S10.2. Hyperparameter setting of GIN

Hyperparameter name	Value	Description
num_layer	3	Number of GNN message passing layers
emb_dim	64	Dimensionality of hidden units in GNNs
lr	1e-4	Learning rate of optimizer
batch_size	32	Input batch size for training
drop_ratio	0.6	Dropout ratio
stepsize	50	Scheduler step size
gamma	0.6	Scheduler shrinking rate

Table S10.3. Hyperparameter setting of GAT

Hyperparameter name	Value	Description
num_layer	5	Number of GNN message passing layers
emb_dim	32	Dimensionality of hidden units in GNNs
lr	1e-3	Learning rate of optimizer
batch_size	16	Input batch size for training
drop_ratio	0.5	Dropout ratio
stepsize	50	Scheduler step size
gamma	0.6	Scheduler shrinking rate

Table S10.4. Hyperparameter setting of BrainGNN

Hyperparameter name	Value	Description
num_layer	2	Number of GNN layers
indim	200	Dimensionality of node feature
lr	1e-2	Learning rate of optimizer
batch_size	64	Input batch size for training
ratio	0.5	Pooling ratio
stepsize	20	Scheduler step size
gamma	0.6	Scheduler shrinking rate
lamb0	1	Classification loss weight
lamb1	0	S1 unit regularization
lamb2	0	S2 unit regularization
lamb3	0.1	S1 entropy regularization
lamb4	0.1	S2 entropy regularization
lamb5	0.1	S1 consistence regularization

Table S10.5. Hyperparameter setting of EV-GNN

Hyperparameter name	Value	Description
hgc	16	Hidden units of gconv layer
lg	4	Number of gconv layers
lr	1e-2	Learning rate of optimizer
edropout	0.3	Edge dropout rate

Table S10.6. Hyperparameter setting of LG-GNN

Hyperparameter name	Value	Description
hgc	64	Hidden units of gconv layer
lg	4	Number of gconv layers
lr	1e-2	Learning rate of optimizer
edropout	0.3	Edge dropout rate
topk_ratio	0.5	The ratio of ROIs differ significantly from those of the unselected nodes to be selected
dropout	0.6	Ratio of dropout

Table S10.7. Hyperparameter setting of ContrastPool

Hyperparameter name	Value	Description
batch_size	20	Input batch size for training
Init_lr	1e-2	The initial learning rate
lr_reduce_factor	0.5	Scheduler shrinking rate
lr_schedule_patience	25	Scheduler step size
min_lr	1e-6	Minimum learning rate
Lamda1	1	The weight factor of the entropy loss of the assignment matrix
Lamda2	1e-3	The weight factor of the entropy loss to the adjacency matrix of the contrast graph
dropout	0	The ratio of dropout
L	2	Number of GNN layers
pooling ratio	0.5	The ratio of nodes in the output graph of each ContrastPool layer

Table S10.8. Hyperparameter setting of pRGAT

Hyperparameter name	Value	Description
num_features	2000	Number of features to keep for the feature selection step
connectivity	correlation	Type of connectivity used for network construction
lr	1e-4	Learning rate of optimizer
hid_c	12	Size of hidden layer

Table S10.9. Hyperparameter setting of MAMF-GCN

Hyperparameter name	Value	Description
pheno_edge_threshold	3.1	Graph sparsification edge threshold of the phenotypic grpah
hgc	16	Hidden units of gconv layer
lg	4	Number of gconv layers
lr	0.01	Learning rate of optimizer
node_feature_dim	900	Dimension of node feature
edropout	0.2	Edge dropout rate
dropout	0.2	Ratio of dropout
snowball_layer_num	6	Num of snowball layer
alpha	5e-5	Weight of modal consistency loss
Beta	1e-6	Weight of modal disparsity loss
K	9	Number of the top k neighbors in the k-nearest graph
Stepsize	100	Scheduler step size
gamma	0.6	Scheduler shrinking rate

Table S10.10. Hyperparameter setting of SFGL

Hyperparameter name	Value	Description
minibatch_size	32	Input batch size
window_size	30	The length of the sliding window
window_stride	2	The stride of the sliding window
lr	1e-3	Learning rate of optimizer
reg_lambda	1e-5	Value of lambda
gamma	0.8	Balancing coefficient of the shared branch and the subject-specific branch
num_epochs	8	Local epochs of federal learning
num_iters	20	Communication rounds of federal learning
num_heads	4	The head number of Transformer
num_layers	2	The number of GIN layer
hidden_dim	64	Output dimension of Transfomer
ph	16	Hidden dimension of personalized branch
sparsity	30	Degree of sparsity
dropout	0.5	Ratio of dropout

Table S10.11. Hyperparameter setting of CI-GNN

Hyperparameter name	Value	Description
batch_size	16	Input batch size for training
latent_dim	[128, 128, 128]	Classifier hidden dims
dropout	0.5	Ratio of final layer dropout
lr	1e-3	Learning rate of optimizer
mlp_hidden	[128,128]	Mlp hidden dims
GVAE_hidden_dim	64	Hidden dims of graph VAE
Nalpha	56	Dimension of causal factor alpha
Nbeta	8	Dimension of non-causal factor beta

Table S10.12. Hyperparameter setting of IBGNN

Hyperparameter name	Value	Description
Batch_size	16	Input batch size for training
n_GNN_layers	2	Number of IBGConv layers
n_MLP_layers	1	Number of MLP layers
lr	1e-3	Learning rate of optimizer
initial_epochs	100	Number of epochs for initial training
explainer_epochs	100	Number of epochs to train the explainer
tuning_epochs	100	Number of epochs to fine-tune the whole model

Table S11. Comparison with other GNN methods on the SRPBS dataset

	ACC	AUC	Precision	Recall	F1-Score
**GCN**	70.25±4.44	67.65±7.00	72.06±5.29	66.49±14.76	68.11±8.78
**GIN**	68.51±4.22	65.14±5.05	72.79±9.85	62.51±23.09	63.86±14.32
**GAT**	68.52±4.83	63.90±6.82	67.72±6.29	69.19±12.52	67.94±8.03
**BrainGNN**	63.88±3.55	59.49±5.48	64.87±6.81	61.59±11.36	62.37±6.89
**EV-GCN**	72.87±4.11	71.58±7.46	74.49±6.84	71.62±5.88	72.61±3.29
**LG-GNN**	73.29±5.08	73.04±6.28	**78.03±7.65**	67.69±13.30	71.32±6.73
**ContrastPool**	72.25±6.17	68.30±10.39	67.71±7.42	69.90±15.93	67.76±9.30
**pRGAT**	71.20±10.65	68.95±13.89	71.28±10.82	71.28±16.39	70.73±12.07
**MAMF-GCN**	75.10±4.03	75.25±5.99	75.33±7.11	77.21±10.55	75.43±3.07
**SFGL**	66.52±2.90	62.49±6.30	69.00±7.95	65.57±15.71	65.50±6.07
**CI-GNN**	70.48±4.13	68.34±7.34	71.88±8.27	69.07±8.58	69.78±4.68
**IBGNN**	65.84±5.84	62.82±6.98	69.35±10.22	63.77±18.06	63.98±9.86
**LGMF-GNN**	**78.75±4.55**	**80.64±5.74**	76.80±7.93	**82.00±5.79**	**78.96±4.88**

Table S12. Comparison with other GNN methods on the REST-meta-MDD dataset

	ACC	AUC	Precision	Recall	F1-Score
**GCN**	64.78±3.21	65.73±4.31	71.00±3.68	54.53±4.76	61.55±3.53
**GIN**	63.31±3.15	63.45±4.63	67.14±7.66	59.64±6.07	62.67±2.99
**GAT**	65.92±2.15	66.95±2.97	68.00±5.32	67.87±13.37	66.83±4.95
**BrainGNN**	63.76±2.59	64.24±3.75	64.59±6.00	71.05±13.16	66.58±4.07
**EV-GCN**	66.24±2.80	68.49±4.41	68.70±5.91	66.98±11.58	66.93±4.39
**LG-GNN**	60.64±2.86	59.86±3.52	63.52±3.99	59.33±14.54	60.08±7.85
**ContrastPool**	67.14±2.80	66.80±5.44	**72.37±6.86**	73.49±11.69	71.91±3.99
**pRGAT**	59.43±3.88	56.36±4.94	61.03±4.81	63.57±15.14	61.08±7.78
**MAMF-GCN**	66.94±3.58	66.76±3.52	71.05±3.59	61.43±12.39	65.18±7.43
**SFGL**	66.95±5.40	64.09±5.98	71.30±12.70	66.90±19.29	65.91±8.14
**CI-GNN**	66.05±3.14	64.91±3.52	66.39±4.28	70.77±10.41	68.00±5.30
**IBGNN**	59.68±4.40	58.90±6.53	63.13±5.73	57.93±19.56	57.78±14.25
**LGMF-GNN**	**71.34±1.50**	**73.67±2.67**	72.20±4.61	**73.52±6.26**	**72.56±2.06**

Table S13. Ablation study performance of local ROI-GNN module

	ACC	AUC	Precision	Recall	F1-Score
LGMF-GNN	78.75±4.55	80.64±5.74	76.80±7.93	82.00±5.79	78.96±4.88
-local ROI-GNN	75.10±4.62	75.96±5.41	76.39±7.21	72.77±8.67	74.31±3.94

Table S14. Ablation study performance of different data modalities

	ACC	AUC	Precision	Recall	F1-Score
LGMF-GNN	78.75±4.55	80.64±5.74	76.80±7.93	82.00±5.79	78.96±4.88
-Demographic	68.66±7.74	73.81±5.74	68.42±12.41	76.20±14.95	69.89±7.91
-Anatomical	73.72±5.05	74.11±7.14	73.83±8.57	73.64±5.89	73.31±4.79

Table S15. Ablation study performance of loss functions

	ACC	AUC	Precision	Recall	F1-Score
LGMF-GNN	78.75±4.55	80.64±5.74	76.80±7.93	82.00±5.79	78.96±4.88
-$L_{specific}$	76.85±4.78	70.08±6.55	76.31±5.68	76.64±5.93	76.40±5.34
-L_{common}	76.89±3.53	75.90±7.29	77.40±7.30	74.86±8.49	75.70±5.78

Table S16. Effect of site effects suppression methods on model generalization ability

	ACC	AUC	Precision	Recall	F1-Score
LGMF-GNN	69.02±3.57	66.33±4.76	72.88±4.55	62.93±2.84	65.94±4.53
+ Combat	72.67±4.51	71.91±3.32	74.23±4.01	67.84±2.46	70.58±3.74
+ Mixup	76.22±4.53	75.76±5.21	74.03±3.87	76.92±3.05	75.04±3.24
+ DropEdge	76.89±3.98	75.94±4.54	74.93±5.67	78.18±6.03	75.98±5.23
+Adversarial	78.22±2.38	78.85±5.50	79.37±9.19	74.05±8.28	75.91±4.27
+End-to-End	78.75±4.55	80.64±5.74	76.80±7.93	82.00±5.79	78.96±4.88

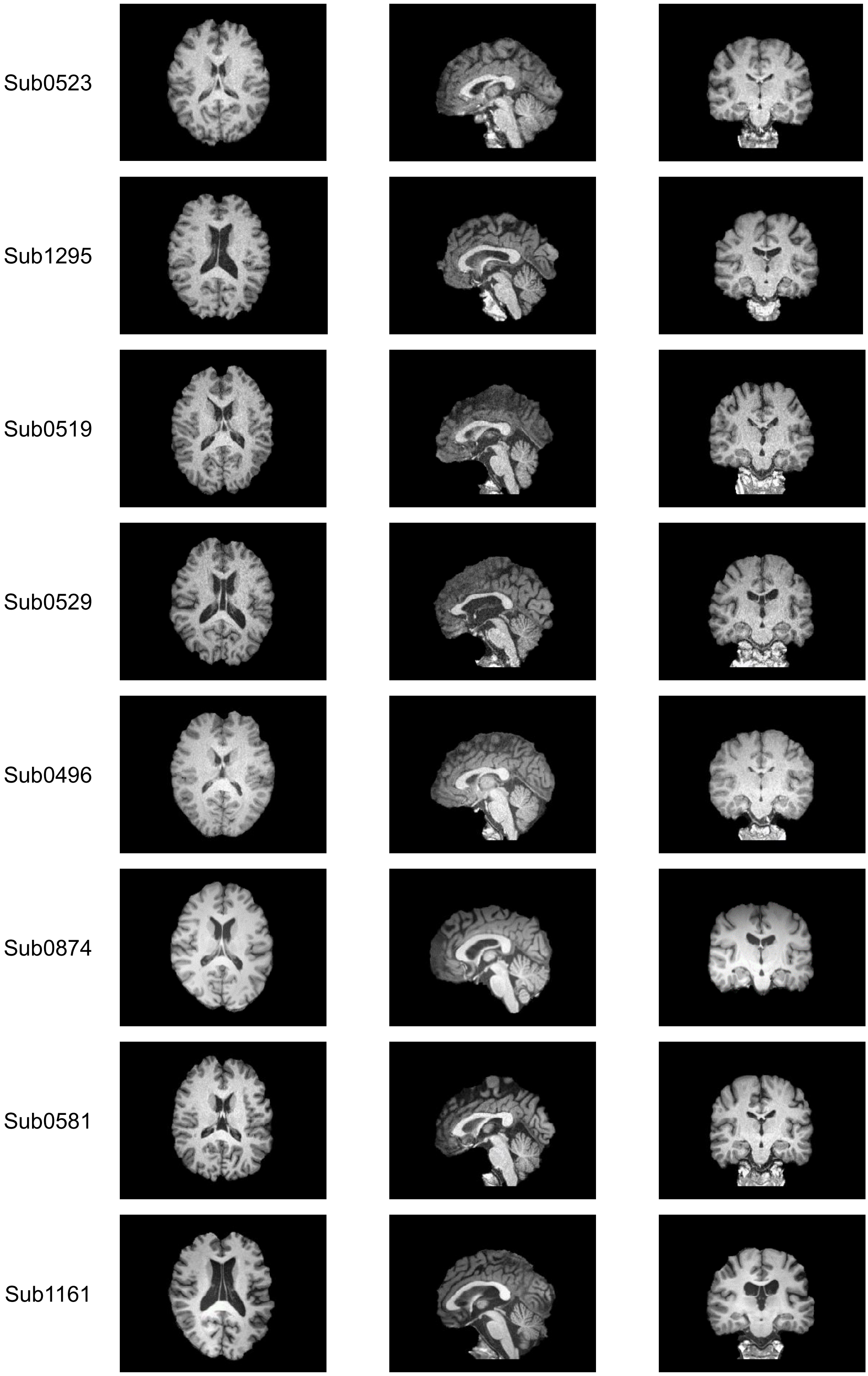

Figure S7. Eight cases corresponding to the MDD nodes with the highest degree in the structural subject graph.

Table S17. Abnormal brain regions and existing research findings

Name in AAL atlas	Brain region name	Existing findings related to functional alteration	Existing findings related to structural alteration
CRBL7b.R, CRBL10.L, CRBL10.R, CRBL8.R	Right Cerebellum lobules VIIB；Left Cerebellum lobules X; Right Cerebellum lobules X; Right Cerebellum lobules VIII；	connectivity analyses have shown reduced cerebro-cerebellar coupling of lobules VI and VIIA/B with prefrontal, posterior parietal, and limbic regions in patients with MDD[18]	The GM density in the right Cerebellum VIII was increased in the MDD patients ($p < 0.05$), and the relative GM densities in the right Cerebellum VIII were significantly correlated with the BDI scores in the MDD patients[19].<br>The GM volumes in right Cerebellum VIII, left Cerebellum X and right Cerebellum X were significantly smaller in the patients with MDD than in the HC subjects ($p < 0.05$), and the GM volumes in right Cerebellum VIII and left Cerebellum X were significantly correlated with the BDI scores in the MDD patients ($p < 0.05$)[19].
HIP.R	Right Hippocampus	In comparison with HCs, patients with BD and MDD had an increased FC between the right anterior hippocampus and lingual gyrus and a decreased FC between the right posterior hippocampus and right IFG ($p<0.00005$)[20]；<br>MDD patients demonstrated decreased right hippocampus–bilateral medial superior frontal gyrus FC relative to HC ($p<0.001$)[21]；<br>Compared to HC, MDD patients showed decreased node strength of the right	MDD patients have smaller hippocampus volumes even in the earlier phase of their illness ($p<0.01$)[25-27];<br>The cumulative analysis revealed hippocampus volume loss in patients with first-episode depression relative to controls in both the left ($p=0.0321$) and right ($p=0.0173$) hippocampus, and the average volume reduction was −4.0% in the left and −4.5% in the right hippocampus[28].

		hippocampus cornu ammonis 3/4 (p<0.001), indicating decreased connectivity to the rest of the brain[22]；<br>The functional connectivity between the right anterior hippocampus and left postcentral gyrus increased with aging in MDD patients compared with HCs (p<0.05)[23]；<br>The MDD group exhibited significantly weaker connectivity of the right hippocampal subregional networks with the temporal cortex (extending to the insula) and basal ganglia but showed increased connectivity of the right subiculum to the bilateral lingual gyrus (p<0.05)[24]	Adolescents with MDD had reduced hippocampal volume compared with healthy adolescents (p=0.006) [29]；<br>Both the MDD and BD patients showed decreased gray matter volume in the right hippocampus compared with the HCs (p<0.001)[30]；<br>Hippocampal white matter was significantly smaller in MDDs than in HCs (p=0.01)[31].
ROL.L	Left Rolandic operculum	The fronto-limbic system has altered in the first-episode and drug-naïve MDD patients， and increased FC of the left rolandic operculum was found (p<0.05)[32].<br>Compared with old HCs, the late-onset recurrent depression patients had higher ReHo in the left rolandic operculum gyrus/left superior temporal gyrus (p<0.05) [33].	-
REC.L	Left gyrus rectus	Compared with HCs, the connectivity between the left rectus gyrus and the left orbital part of the superior frontal gyrus is enhanced in patients with treatment-naive depression (p=0.0054)[34]	For structural MRI, the greatest difference between healthy individuals and those with depression could be observed in a voxel in the left gyrus rectus[35]
PreCG.R	Right Precentral gyrus	Compared with HCs, MDD patients had a higher regional cerebral blood flow in the right Precental gyrus (PreCG) (p<0.001)[36]；<br>The non-anxious depression patients showed decreased FC	-

		between the right precentral gyrus and the right centromedial /laterobasal compared to the HC group ($p<0.001$) [37]; There was a significant reduction in ReHo and ALFF in the bilateral precentral gyrus in the somatic MDD group as compared to the pure depression group ($p<0.01$)[38]; HCs had greater activation compared to MDD+PTSD patients in the right precentral gyrus ($p<0.001$)[39].	
ROL.R	Right Rolandic operculum	Compared with HCs, patients with MDD had significantly higher ALFF in the left cerebellum and significantly lower ALFF in the right rolandic opercular cortex ($p<0.001$)[40]; Increased ALFF in the contralesional rolandic operculum was found in the slow-5 frequency band, and this post-stroke depression-specific alteration could predict depression severity [41].	Lesions in the right Rolandic operculum are associated with self-rating affective and apathetic depressive symptoms for post-stroke patients[42].
CRBLCrus2.R	Right Cerebellum Crus II	Compared with HCs, patients with MDD showed significantly increased ReHo values in the right cerebellum Crus2 ($p<0.001$). Additionally, partial correlation analysis showed that the right cerebellum crus2 and right angular gyrus were positively associated with working memory ($r=0.277$, $p=0.038$)[43].	-

Table S18. Key indicators of sMRI imaging radiomics for the diagnosis of depression

**Name**	**Description**	**Implication for Depression Diagnosis**
GLCM_MaximumProbability	The maximum probability of co-occurring pixels within a specified distance and angle, suggesting the homogeneity in the image texture	Alterations in the microstructural organization of gray and white matter; Disruptions in neural connectivity and organization
FirstOrder_Uniformity	The similarity of the intensity distribution in a given region, indicating the uniformity of an image	Disruptions in the typical distribution of grey and white matter intensities; Irregularities in tissue composition which indicate neurostructural changes associated with depression
FirstOrder_Range	The difference between the maximum and minimum voxel intensity values of an image	Variations in tissue density; The magnitude and range of anatomical changes related to depression
GLRLM_RunLengthNonUniformity	The deviation of the run lengths from the average run length value, suggesting the heterogeneity in the texture	Disruptions in the regularity of structural patterns of grey and white matter; Changes in tissue composition, connectivity, or other microstructural features related to depression
GLSZM_GrayLevelNonUniformityNormalized	The degree of variability of discrete voxel intensity values in an image, indicating the heterogeneity of voxel intensities	Irregularities in tissue structure and composition of grey and white matter; Grey and white matter morphological abnormalities associated with depression

Table S19. Imaging parameters used for SRPBS dataset

rs-fMRI						
Protocol #	2	3	4	5	6	8
Site	Hiroshima University Hospital	Hiroshima University Hospital	Hiroshima Kajikawa Hospital	Center of Innovation in Hiroshima University	Kyoto university	University of Tokyo
MRI Scanner	GE Signa HDxt	GE Signa HDxt	SIEMENS Spectra	SIEMENS MAGNETOM Verio.Dot	SIEMENS TimTrio	GE Discovery MR750w
Magnitic field strength	3T	3T	3T	3T	3T	3T
Number of channels per coil	8HR-BRAIN	8HR-BRAIN	head-12ch	head-12ch	32-channel phased array head coil	Head 24
TR (s)	2	2	2.7	2.5	2,500	2.5
TE (ms)	27	27	31	30	30	30
Flip angel (deg)	90	90	90	80	80	80
Phse encoding	PA	AP	AP	AP	PA	PA
Matrix	64 x 64	64 x 64	64 x 64	64 x 64	64 x 64	64 x 64
Field of view (mm)	256	256	192	212	212 x 212	212
In-plane resolution (mm)	4.0 x 4.0	4.0 x 4.0	3.0 x 3.0	3.3 x 3.3	3.3125 X 3.3125	3.3
Slice thickness (mm)	4	4	3	3.2	3.2	3.2

Slice gap (mm)	0	0	0	0.8	0.8	0.8
Number of slices	32	32	38	40	40	40
Slice acquisition order	Ascending (Interleaved)	Ascending (Interleaved)	Ascending	Ascending	Ascending	Ascending
Number of volumes	143 + 7 (dummy)	143 + 7 (dummy)	107 + 5 (dummy)	240 + 4 (dummy)	240 + 4 (dummy)	240 + 4 (dummy)
Total scan time	4 min. 46s. + 14s (dummy)	4 min. 46s. + 14s (dummy)	4 min. 49s. + 14s (dummy)	10 min + 10s (dummy)	10 min + 10s (dummy)	10 min + 10s (dummy)
Eye closed/fixate	Fixate	Fixate	Fixate	Fixate	Fixate	Fixate
**Structural (T1w)**						
Protocol #	2	3	4	5	6	8
Site	Hiroshima University Hospital	Hiroshima University Hospital	Hiroshima Kajikawa Hospital	Center of Innovation in Hiroshima University	Kyoto university	University of Tokyo
Scanner	GE Signa HDxt	GE Signa HDxt	SIEMENS Spectra	SIEMENS MAGNETOM Verio.Dot	SIEMENS TimTrio	GE Discovery MR750w
Voxel size (mm^3)	1 x 1 x 1	1 x 1 x 1	1 x 1 x 1	1 x 1 x 1	0.9375 x 0.9375 x 1.0	1 x 1 x 1.2
TR (ms)	6812	6812	1900	2300	2000	7.7
TE (ms)	1896	1896	2.38	2.98	3.4	3.1
TI (ms)	450	450	900	900	990	400
Flip angel (deg)	20	20	10	9	8	11
FOV	256	256	256	256	225 x 240	240
Matrix	256 x 256	256 x 256	256 x 256	256 x 256	240 x 256	256 x 256

Table S20. Imaging parameters used for REST-meta-MDD dataset

Cohort	Site	MRI Scanner	Magnitic field strength	Receive (coil channel)	TR (s)	TE (ms)	Flip angel (deg)	Field of view (mm)	Voxel size	Slice thickness (mm)	Number of slices	Number of volumes
1	National Clinical Research Center for Mental Disorders (Peking University Sixth Hospital) & Key Laboratory of Mental Health, Ministry of Health (Peking University)	Siemens Tim Trio 3T	3T	32	2000	30	90	210×210	3.28×3.28 ×4.80	4.0/0.8	30	210
2	Department of Clinical Psychology, Suzhou Suzhou Psychiatric Hospital, The Affiliated Guangji Hospital of Soochow University	Philips Achieva 3T	3T	8	2000	30	90	240×240	1.67×1.67 ×4.00	4.0/0	37	200
7	Sir Run Run Shaw Hospital, Zhejiang University School of Medicine	GE discovery MR750	3T	8	2000	30	90	220×220	2.29×2.29 ×3.20	3.2/0	37	184
8	Department of Psychiatry, First Affiliated Hospital, China Medical University	GE Signa 3T	3T	8	2000	30	90	240×240	3.75×3.75 ×3.00	3.0/0	35	200

9	The First Affiliated Hospital of Jinan University	GE Discovery MR750 3.0T	3T	8	2000	25	90	240×240	3.75×3.75 ×4.00	3.0/1.0	35	200
10	First Hospital of Shanxi Medical University	Siemens Tim Trio 3T	3T	32	2000	30	90	240×240	3.75×3.75 ×4.52	3.0/1.52	32	212
11	Department of Psychiatry, The First Affiliated Hospital of Chongqing Medical University	GE Signa 3T	3T	8	2000	30	90	240×240	3.75×3.75 ×5.00	5	33	200
13	The First Affiliated Hospital of Xi'an Jiaotong University, Xi'an Central Hospital	GE Excite 1.5T	1.5T	16	2500	35	90	256×256	4.00×4.00 ×4.00	4/0	36	150
14	The Second Xiangya Hospital of Central South University	Siemens Tim Trio 3T	3T	32	2500	25	90	240×240	3.75×3.75 ×3.50	3.5/0	39	200
15	Department of Psychosomatics and Psychiatry, Zhongda Hospital, School of Medicine, Southeast University	Siemens Verio 3.0T MRI	3T	12	2000	25	90	240×240	3.75×3.75 ×4.00	4/0	36	240
17	Department of Psychiatry, The First Affiliated Hospital of Chongqing Medical	GE Signa 3T	3T	8	2000	40	90	240×240	3.75×3.75 ×4.00	4.0/0	33	240

	University											
19	Anhui Medical University	GE Signa 3T	3T	8	2000	22.5	30	220×220	3.44×3.44 ×4.60	4.0/0.6	33	240
20	Faculty of Psychology, Southwest University	Siemens Tim Trio 3T	3T	12	2000	30	90	220×220	3.44×3.44 ×4.00	3.0/1.0	32	242
21	Beijing Anding Hospital, Capital Medical University	Siemens Tim Trio 3T	3T	32	2000	30	90	200×200	3.12×3.12 ×4.20	3.5/0.7	33	240
22	The Institute of Mental Health, Second Xiangya Hospital of Central South University	Philips Gyroscan Achieva 3.0T	3T	32	2000	30	90	240×240	1.67×1.67 ×4.00	4.0/0	36	250
23	Mental Health Center, West China Hospital, Sichuan University	Philips Achieva 3.0T TX	3T	8	2000	30	90	240×240	3.75×3.75 ×4.00	4.0/0	38	240

Table S21. Category distribution of the included SRPBS dataset participants by site

Site	MDD	HC	Total
2	49	49	98
3	16	14	30
4	32	25	57
5	65	65	130
6	16	15	31
8	51	60	111
Total	229	228	457

Table S22. Category distribution of the included REST-meta-MDD dataset participants by site

Site	MDD	HC	Total
1	73	73	146
2	16	14	30
7	35	37	72
8	39	48	87
9	48	48	96
10	45	26	71
11	20	17	37
13	20	16	36
14	61	32	93
15	30	37	67
17	41	41	80
19	18	31	49
20	249	228	477
21	79	65	144
22	18	20	38
23	22	23	45
Total	814	756	1570

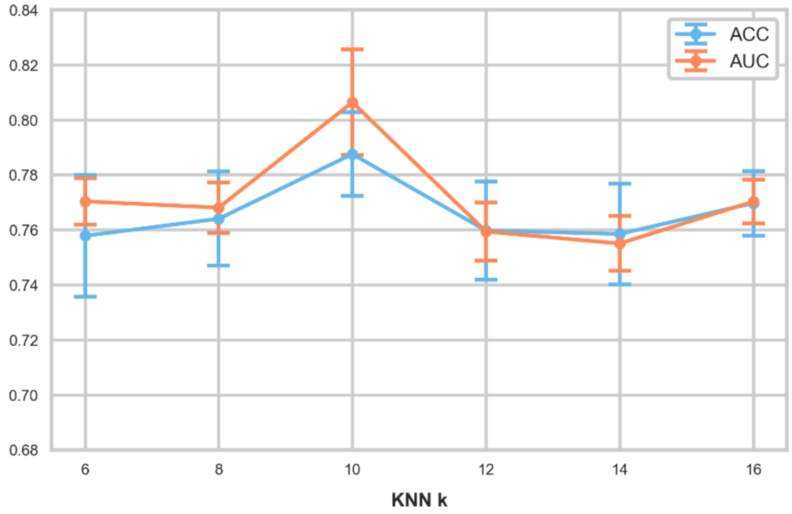

Figure S8. Experimental results for the optimal number of nearest neighbors during KNN graph construction.

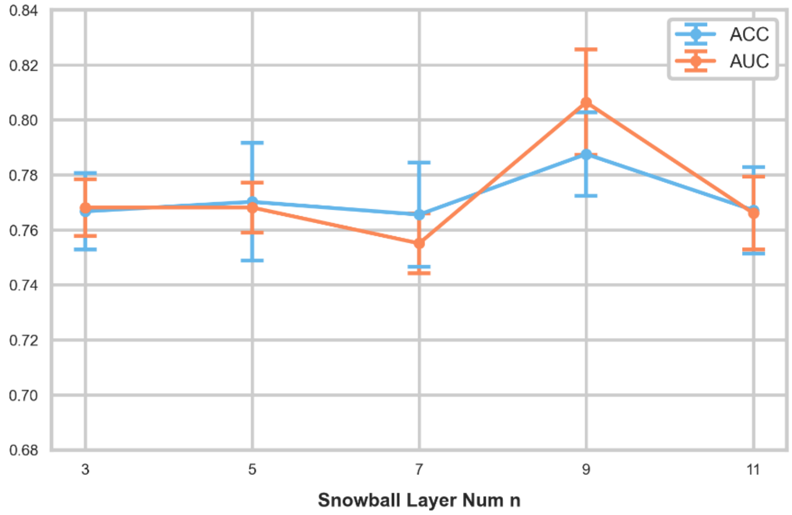

Figure S9. Experimental results for the optimal number of Snowball Layer.

Table S23. The impact of brain atlases on model performance.

	ACC	AUC	Precision	Recall	F1-Score
2atlas	74.57±4.16	74.52±5.53	75.48±9.76	72.04±3.36	73.42±5.45
AAL	**78.75±4.55**	**80.64±5.74**	**76.80±7.93**	**82.00±5.79**	**78.96±4.88**
CC200	76.63±4.97	76.72±8.18	76.44±9.67	75.46±10.77	75.49±8.36

Table S24. Performance of LGMF-GNN on the unbalanced SRPBS validation dataset.

	ACC	AUC	Precision	Recall	F1-Score
LGMF-GNN	86.50±8.08	82.97±11.19	68.40±20.31	87.50±16.77	73.50±12.76

Table S25. The full list of DIRECT consortium members

Chao-Gan Yan[1,2,3,4,5,6], Xiao Chen[1,2,3,4], Li-Ping Cao[9], Wei Chen[11], Yu-Qi Cheng[12], YiRu Fang[13], Qi-Yong Gong[14,15], Wen-Bin Guo[16], Li Kuang[17], Bao-Juan Li[18], Tao Li[20,21], Yan-Song Liu[22], Zhe-Ning Liu[16], Jian-Ping Lu[23], Qing-Hua Luo[17], Hua-Qing Meng[17], Dai-Hui Peng[13], Jiang Qiu[24], Yue-Di Shen[25], Tian-Mei Si[26], Yan-Qing Tang[27], Chuan-Yue Wang[28], Fei Wang[27,29], Hua-Ning Wang[18], Kai Wang[30], Xiang Wang[16], Ying Wang[31], Xiao-Ping Wu[32], Chun-Ming Xie[33], Guang-Rong Xie[16], Peng Xie[34,35,36], Xiu-Feng Xu[12], Hong Yang[37], Jian Yang[35], Shu-Qiao Yao[16], Yong-Qiang Yu[38], Yong-Gui Yuan[39], Ke-Rang Zhang[40], Wei Zhang[41], Zhi-Jun Zhang[33], Jun-Juan Zhu[42], Xi-Nian Zuo[43,44], Jing-Ping Zhao[16], Yu-Feng Zang[45,46]

[1]CAS Key Laboratory of Behavioral Science, Institute of Psychology, Chinese Academy of Sciences, Beijing 100101, China
[2]International Big-Data Center for Depression Research, Chinese Academy of Sciences, Beijing 100101, China
[3]Magnetic Resonance Imaging Research Center, Institute of Psychology, Chinese Academy of Sciences, Beijing 100101, China
[4]Department of Psychology, University of Chinese Academy of Sciences, Beijing 100049, China
[5]Sino-Danish College, University of Chinese Academy of Sciences, Beijing 101408, China
[6]Sino-Danish Center for Education and Research, Graduate University of Chinese Academy of Sciences, Beijing 101408, China
[9]Affiliated Brain Hospital of Guangzhou Medical University, Guangzhou 510370, China
[11]Department of Psychiatry, Sir Run Run Shaw Hospital, Zhejiang University School of Medicine, Hangzhou 310020, Zhejiang, China
[12]Department of Psychiatry, First Affiliated Hospital of Kunming Medical University, Kunming, Yunnan 650032, China
[13]Shanghai Mental Health Center, Shanghai Jiao Tong University School of Medicine, Shanghai 200030, China
[14]Huaxi MR Research Center (HMRRC), Department of Radiology, West China Hospital of Sichuan University, Chengdu, Sichuan 610044, China
[15]Research Unit of Psychoradiology, Chinese Academy of Medical Sciences, Chengdu, Sichuan 610052, China
[16]Department of Psychiatry, and National Clinical Research Center for Mental Disorders, The Second Xiangya Hospital of Central South University, Changsha 410011, Hunan, China
[17]Department of Psychiatry, The First Affiliated Hospital of Chongqing Medical University, Chongqing 400042, China
[18]Xijing Hospital of Air Force Military Medical University, Xi’an, Shaanxi 710032, China
[20]Affiliated Mental Health Center & Hangzhou Seventh People’s Hospital, Zhejiang University School
of Medicine, Hangzhou, Zhejiang 310063, China
[21]Mental Health Center and Psychiatric Laboratory, West China Hospital of Sichuan University, Chengdu, Sichuan 610044, China
[22]Department of Clinical Psychology, Suzhou Psychiatric Hospital, The Affiliated Guangji Hospital of Soochow University, Suzhou, Jiangsu 215003, China
[23]Shenzhen Kangning Hospital, Shenzhen, Guangzhou 518020, China
[24]Faculty of Psychology, Southwest University, Chongqing 400715, China
[25]Department of Diagnostics, Affiliated Hospital, Hangzhou Normal University Medical School, Hangzhou, Zhejiang 311121, China
[26]National Clinical Research Center for Mental Disorders (Peking University Sixth Hospital) & Key Laboratory of Mental Health, Ministry of Health (Peking University), Beijing 100191, China
[27]Department of Psychiatry, First Affiliated Hospital, China Medical University, Shenyang, Liaoning 110122, China
[28]Beijing Anding Hospital, Capital Medical University, Beijing 100120, China
[29]Early Intervention Unit, Department of Psychiatry, Affiliated Nanjing Brain Hospital, Nanjing Medical University, Nanjing 210024, China
[30]Department of Neurology, The First Affiliated Hospital of Anhui Medical University, Hefei,

Anhui 230022, China
[31]The First Affiliated Hospital of Jinan University, Guangzhou, Guangdong 250024, China
[32]Xi'an Central Hospital, Xi'an, Shaanxi 710004, China
[33]Department of Neurology, Affiliated ZhongDa Hospital of Southeast University, Nanjing, Jiangsu 210009, China
[34]Institute of Neuroscience, Chongqing Medical University, Chongqing 400016, China
[35]Chongqing Key Laboratory of Neurobiology, Chongqing 400000, China
[36]Department of Neurology, The First Affiliated Hospital of Chongqing Medical University, Chongqing 400042, China
[37]Department of Radiology, The First Affiliated Hospital, College of Medicine, Zhejiang University, Hangzhou, Zhejiang 310058, China
[38]The First Affiliated Hospital of Anhui Medical University, Hefei, Anhui 230032, China
[39]Department of Psychosomatics and Psychiatry, Zhongda Hospital, School of Medicine, Southeast University, Nanjing, Jiangsu 210009, China
[40]First Hospital of Shanxi Medical University, Taiyuan, Shanxi 030001, China
[41]West China Hospital of Sichuan University, Chengdu, Sichuan 610044, China
[42]Department of Psychiatry, Shanghai Jiao Tong University School of Medicine, Shanghai 200025, China
[43]Developmental Population Neuroscience Research Center, IDG/McGovern Institute for Brain Research, Beijing Normal University, Beijing 100091, China
[44]National Basic Science Data Center, Beijing 100038, China
[45]Center for Cognition and Brain Disorders, The Affiliated Hospital of Hangzhou Normal University, Hangzhou, Zhejiang 310018, China
[46]Zhejiang Key Laboratory for Research in Assessment of Cognitive Impairments, Hangzhou, Zhejiang 310000, China

Supplemental Experimental Procedures

Supplemental Experimental Procedures. Data cleaning and quality control

REST-meta-MDD:
As shown in **Figure. S1**. To control the data quality and avoid introduction of bias, subjects were selected from a total of 2428 subjects as follows: (1) subjects older than 65 years or younger than 18 years were excluded; (2) subjects with missing information on age, sex or education year were excluded; (3) low quality images with bad coverage (< 90% group mask), poor spatial normalization (visual inspection) or excessive head motion (mean framewise displacement (FD) > 0.2) were discarded; (4) to further exclude subjects with distortions that were not detected by visual inspection, we excluded subjects with spatial correlation < 0.6 (a threshold defined by mean - 2SD) between each subject's regional homogeneity (ReHo) map and the group mean ReHo map; (5) sites with subjects fewer than 10 in either MDD or HC group was removed; (6) sites mainly contained patients with geriatric depression subjects were excluded; (7) one duplicated site (detected after consortium data sharing) was discarded; (8) images with zero signals detected in targeted atlas were discarded.

SRPBS:
As shown in **Figure. S2**. To control the data quality and avoid introduction of bias, subjects were selected from a total of 1410 subjects as follows: (1) subjects diagnosed with other disease were excluded; (2) subjects from sites that did not contain MDD patients were excluded; (3) to make the data more balanced, subjects with the same number of MDDs were randomly selected from HCs; (4) low quality images with excessive head motion (mean framewise displacement (FD) > 0.4) were discarded; (5) images with zero signals detected in targeted atlas were discarded.

Anding:
As shown in **Figure. S3**. To control the data quality and avoid introduction of bias, subjects were selected from a total of 465 subjects as follows: (1) subjects missing T1 sMRI or rs-fMRI scan were excluded; (2) low quality images with excessive head motion (mean framewise displacement (FD) > 0.2) were discarded; (3) images with zero signals detected in targeted atlas were discarded.

Openneuro:
As shown in **Figure. S4**. To control the data quality and avoid introduction of bias, subjects were selected from a total of 72 subjects as follows: (1) subjects diagnosed as mild depressive disorder (ICD-10 F32.0) or missing ICD-10 diagnosis were excluded; (2) low quality images with excessive head motion (mean framewise displacement (FD) > 0.2) were discarded; (3) images with zero signals detected in targeted atlas were discarded.

Supplemental Experimental Procedures. The evaluation of site-effect

The datasets utilized in this study exhibit high heterogeneity. Specifically, the SRPBS dataset was collected from six different sites with varying acquisition protocols, and the participants in the REST-meta-MDD dataset included in this study involves 16 cohorts from different hospitals. To make reasonable and adequate use of these experimental data, multicenter correction is a necessary and important step. Multicenter correction involves harmonizing fMRI-derived data from multiple acquisition sites, study cohorts or devices. The purpose of this correction is to eliminate site effects that arise from differences in image scanners, scanning parameters, and image processing flows. Strong site effects can impact the reliability, reproducibility, and generalization ability of the data, which, in turn, can affect the interpretation of the results.

To evaluate the extent of site effects, we initially conducted experiments from two perspectives: 1) The data distribution, specifically the brain functional connectivity matrix calculated based on the BOLD signal, and 2) The interference with the model's generalization ability.

1) The data distribution For the brain functional connectivity matrix calculated from fMRI, the upper triangle of the functional connectivity matrix based on the AAL atlas was flattened to a one-dimensional vector and then normalized by the Z-Score standardization method in this paper. To visualize the site effects present in the functional connectivity matrix data, dimensional reduction using t-SNE is performed, followed by creating a scatter plot to illustrate the data distribution. **Figure S6** reveals that before correction, the data from each site has a tendency to aggregate and there are more outliers, indicating that the original functional connectivity data derived from fMRI has strong site effects. Directly inputting this data into the model would lead to confusion and confounding.

2) The interference with the generalization ability To examine the impact of site effects on model's performance, we conducted a pre-experiment using single-site data from site 20 of the REST-meta-MDD dataset and data from six sites of the SRPBS dataset. We employed the global Subject-GNN network for ten-fold cross-validation, and the results are shown in **Table S3**. It is evident that the model performs significantly better on the single-site dataset than on the multi-site dataset, with the cross-validation results for the multi-site dataset showing a decrease of nearly 20% in comparison to the single site. This indicates that site effects have a significant impact on both the training and generalization performance of the model. Therefore, the development of an effective multicenter correction method is essential to mitigate the influence of site effects.

Supplemental Experimental Procedures. System performance for MDD subtype classification

We conducted an experiment where we divided the MDD patients in the REST-meta-MDD dataset into two subsets: "first-episode drug-naïve" (FEDN) and "recurrent" (RECU).

The subset partition according to FEDN and RECU references the previous work[1] published in PNAS by the REST-meta-MDD dataset providers, aiming to explore the model's ability to distinguish different disease subtypes. The medication status was further considered for the first-episode group primarily because this group has detailed and reliable medication records in the dataset. Moreover, medication use may affect the physiological and psychological states of patients, thereby influencing diagnostic accuracy. Excluding the influence of medication for first-episode patients helps to more clearly identify the initial state and characteristics of depression. The medication status was not specifically considered for the recurrent group because these patients may have already received pharmacological treatment in previous episodes, and the dataset lacks detailed records of their medication use. Additionally, recurrence itself indicates the uniqueness and complexity of the disease. In this case, the research focus may shift more towards understanding the recurrence mechanisms and seeking more effective treatments.

For the FEDN vs HC subset and the RECU vs HC subset, we first filtered all FEDN and RECU subject samples from the REST-meta-MDD dataset. We then randomly sampled an equal number of HC samples from the sites where the FEDN and RECU subjects originated. In cases where the number of HC at a particular site was fewer than MDD, we supplemented HC samples from site 20, which had the largest data volume, to achieve a 1:1 ratio of HC to MDD in the subset. For the FEDN vs RECU subset, we only selected samples from the overlapping acquisition sites (i.e., site 9 and site 20) for the two subtypes. The category distribution across sites after filtering is shown in T**able S4-S6**. And the performance of the proposed system for MDD subtype classification is shown in **Table S7**.

Supplemental Experimental Procedures. Comparison experiments

To further validate the proposed system, LGMF-GNN is compared to state-of-the-art (SOTA) graph neural network models for brain disease diagnosis. Specifically, we assessed the performance of LGMF-GNN against traditional GCN[2], GIN[3], and GAT[4] networks and popular brain graph neural networks, namely BrainGNN[5], EV-GCN[6], LG-GNN[7], ContrastPool[8], pRGAT[9], MAMF-GCN[10], SFGL[11], CI-GNN[12] and IBGNN[13]. Among these, BrainGNN, SFGL, CI-GNN and IBGNN are local-view methods based on graph-level classification of ROI graphs, EV-GNN, pRGAT and MAMF-GCN are global-view methods based on node-level classification on population graphs, and LG-GNN and ContrastPool are local-to-global methods

Here, we briefly introduce the experimental design and the setting of hyperparameters of the comparison method.

All the comparison methods outlined below utilize the same loss functions and optimizers as described in the original paper. For methods such as GCN, GIN, and GAT, which are not specifically designed for brain graphs, we have provided a detailed description of how the graph and node features are constructed. For methods like BrainGNN, which are tailored for brain graphs, we have strived to maintain consistency with the original paper in terms of network architecture and hyperparameter selection in our implementation. In cases where the hyperparameters are not applicable due to changes in atlas or usage scenarios, such as different disease types, we have conducted random searches within the parameter space defined in the original paper to determine the optimal hyperparameter settings for the comparative experiments. The hyperparameter settings for each method are detailed in **Table S10**.

1) GCN

GCN is one of the earliest and most influential approaches in the GNN domain. They extend the concept of convolution from Euclidean data to non-Euclidean graph data. The authors motivate the choice of the convolutional architecture via a localized first-order approximation of spectral graph convolutions

In the implementation of GCN, we modeled depression diagnosis as a graph-level classification task, each subject was constructed as a graph, and the ROIs defined by AAL atlas were used as nodes. The node features were the row vectors corresponding to the functional connectivity matrix calculated by Fisher-Z transformation according to the Pearson correlation coefficient of ROI BOLD signal. The absolute value of the functional connectivity matrix was used as the graph adjacency matrix.

2) GIN

GIN aims to improve on the aggregation mechanism of GCNs by ensuring that the representation of a node is sufficiently influenced by its neighbors, even in the presence of isomorphic subgraphs. The key operation in GINs is a "set-aggregation" function that sums the features of a node and its neighbors, followed by a transformation that includes a learnable function of the node's own feature. This design ensures that GINs can distinguish between different nodes, even if they have the same number of neighbors and similar neighbor features, thus enhancing the expressive power of the model.

In our implementation of GIN, the graph and node features were constructed in the same way as GCN.

3) GAT

GAT uses an Attention mechanism that allows different weights to be assigned to different nodes and relies on pairs of neighboring nodes for training, rather than on a specific network structure, and can be used for inductive tasks.

In our implementation of GAT, the graph and node features were constructed in the same way as GCN.

4) BrainGNN

BrainGNN is a graph neural network (GNN) framework to analyze functional magnetic resonance images (fMRI) and discover neurological biomarkers. Considering the special property of brain graphs, the authors design novel ROI-aware graph convolutional (Ra-GConv)

layers that leverage the topological and functional information of fMRI. Motivated by the need for transparency in medical image analysis, BrainGNN contains ROI-selection pooling layers (R-pool) that highlight salient ROIs (nodes in the graph), so that it can infer which ROIs are important for prediction. Furthermore, regularization terms including unit loss, topK pooling (TPK) loss and group-level consistency (GLC) loss were proposed on pooling results to encourage reasonable ROI-selection and provide flexibility to encourage either fully individual-patterns or patterns that agree with group-level data.

In our implementation, brain ROI was defined according to the AAL atlas, and the methods of node feature extraction and dimension reduction and graph construction were consistent with the original paper.

5) EV-GCN

In this paper, the authors present a generalizable framework designed to automatically integrate imaging data with non-imaging data in populations for uncertainty-aware disease prediction. The framework's centerpiece is a learnable adaptive population graph equipped with variational edges. The authors mathematically demonstrate that this graph can be optimized in conjunction with graph convolutional neural networks. To quantify the predictive uncertainty associated with the graph topology, they introduce the novel concept of Monte-Carlo edge dropout. Across four different databases, experimental results indicate that the authors' method can consistently and significantly enhance diagnostic accuracy for Autism spectrum disorder, Alzheimer's disease, and ocular diseases.

In our implementation, brain ROI was defined according to the AAL atlas, and demographic information included age, sex, and site. The methods of node feature extraction and dimension reduction and graph construction were consistent with the original paper.

6) LG-GNN

The LG-GNN model is a local-to-global graph neural network structured to include a local ROI-GNN component, which is tasked with learning feature embeddings of local brain regions and pinpointing biomarkers. Subsequently, a global Subject-GNN is constructed to discern the relationships between subjects by utilizing the embeddings produced by the local ROI-GNN in conjunction with non-imaging data. The local ROI-GNN is equipped with a self-attention based pooling module that is designed to retain the most crucial embeddings for the classification task. On the other hand, the global Subject-GNN incorporates an adaptive weight aggregation block that generates multi-scale feature embeddings specific to each subject. The LG-GNN model has been rigorously tested using two public datasets for the classification of ASD and AD. The experimental findings revealed that this approach not only achieves state-of-the-art performance but also excels across a range of evaluation metrics.

In our implementation, brain ROI was defined according to the AAL atlas, and demographic information included age, sex, and site. The methods of node feature extraction and dimension reduction and graph construction were consistent with the original paper.

7) ContrastPool

In their paper, the authors propose a contrastive dual-attention block, which adaptively assigns a weight to each ROI of each subject, performs adaptive aggregation over subjects in each group, and makes contrast across different groups to obtain a contrast graph. Guided by the contrast graph, the authors introduce a differentiable graph pooling method called ContrastPool to generate brain network representations that are effective to the task of disease classification. They have applied their method to five resting-state fMRI brain network datasets across three diseases and have shown its superiority compared to state-of-the-art baselines. The case study conducted by the authors confirms that the patterns extracted by their method correspond with the domain knowledge found in neuroscience literature and reveal direct and intriguing insights. Their contributions highlight the potential of ContrastPool in enhancing the understanding of brain networks and neurodegenerative conditions.

In our implementation, brain ROI was defined according to the AAL atlas. The methods of node feature extraction and dimension reduction and graph construction were consistent with the original paper.

8) pRGAT

In an effort to enhance the valid information in ASD prediction, this paper has investigated various methods to construct the population graph, including Phenotype-Edge (P-Edge), fMRI-

Edge (F-Edge), and a combination of phenotype and fMRI-Edge (PF-Edge). Additionally, they have introduced Graph Attention Networks (GAT) to capture the correlation between subjects on the graph's node-features, which was previously overlooked by GCN-based methods. However, the original GAT architecture does not account for edge-features. To leverage the structural information encoded in edge-features, the researchers further introduced relation-aware attention through the Relational Graph Attention Network (RGAT), which is based on GAT. Using the three graph structures and RGAT, they proposed three ASD prediction models: RGAT with P-Edge (p-RGAT), RGAT with F-Edge (f-RGAT), and RGAT with PF-Edge (pf-RGAT). Experiment results have shown that the integration of relation-aware attention through RGAT, based on GAT, has enhanced the ASD prediction model's ability to learn more diverse information, thereby improving the model's generalization ability.

As a comparative method for the proposed model, we utilized the pf-RGAT model structure that exhibited the best performance in the original paper. This model integrates phenotype data and fMRI data to determine the edges between nodes in the population graph. Moreover, it employs the Relational Graph Attention Convolutional layer (RGATConv), which takes into account not only the correlation of node features but also the relevance of edge features, allowing RGAT to more comprehensively capture the complex relationships between individuals. In our implementation, brain ROI was defined according to the AAL atlas, and demographic information included age, sex, and site. The methods of node feature extraction and dimension reduction and graph construction were consistent with the original paper.

9) MAMF-GCN

In this paper, the researchers propose a multi-scale adaptive multi-channel fusion deep graph convolutional network with an attention mechanism (MAMF-GCN) to better integrate features of various modalities and atlases by exploiting multi-channel correlations. An encoder automatically merges one channel with non-imaging data to produce similarity weights between subjects using a similarity perception mechanism. Additional channels generate multi-scale imaging features of fMRI data after processing across different atlases. An adaptive convolution module fuses multi-modal information, applying a deep graph convolutional network (GCN) to extract information from more profound hidden layers. The researchers assessed the performance of the proposed method on the Autism Brain Imaging Data Exchange (ABIDE) dataset and the Major Depressive Disorder (MDD) dataset. Experimental results indicate that the proposed method surpasses many state-of-the-art methods in node classification performance.

In the original paper, the AAL and HO (Harvard-Oxford) atlases were employed to build the fMRI functional k-nearest neighbor (knn) population graph. To maintain alignment with our research, we utilized the AAL and CC200 atlases for constructing the fMRI functional knn population graph in our implementation. Demographic data, including age, sex, and site, were incorporated to create the phenotypic graph. The techniques for node feature extraction, dimensionality reduction, and graph construction were adopted in line with the methods described in the original paper.

10) SFGL

In this paper, the research team has proposed a specificity-aware federated graph learning (SFGL) framework for rs-fMRI analysis and automated brain disorder identification. This framework consists of a server and multiple clients or sites for federated model aggregation and prediction. At each client, the model is divided into a shared branch and a personalized branch. Parameters of the shared branch are sent to the server, while those of the personalized branch stay local, promoting knowledge sharing among sites while maintaining site specificity. In the shared branch, the team employs a spatio-temporal attention graph isomorphism network to learn dynamic fMRI representations. In the personalized branch, they integrate vectorized demographic information (including age, gender, and education years) and functional connectivity networks to retain site-specific characteristics. The representations generated by both branches are then fused for classification purposes. The experimental results on two fMRI datasets, totaling 1218 subjects, indicate that the SFGL framework outperforms several state-of-the-art approaches.

In our implementation, brain ROI was defined according to the AAL atlas, and demographic information included age, sex, and site. The methods of dynamic graph sequence construction and dynamic graph representation learning are consistent with the original paper.

11) CI-GNN

This study presents the CI-GNN, a novel Granger causality-inspired model that intrinsically identifies influential subgraphs within brain networks that are causally tied to diagnostic outcomes, such as differentiating between patients with major depressive disorder and healthy individuals. Unlike conventional approaches, CI-GNN does not necessitate additional interpretive models post-training. It employs a graph variational autoencoder framework to learn subgraph representations that capture both causal and non-causal elements, regulated by a conditional mutual information (CMI) constraint to ensure the fidelity of the causal relationships. Theoretical validation supports the CMI constraint's effectiveness in capturing causality. Empirical assessments against other GNNs and explainers on synthetic and real-world brain disease datasets demonstrate CI-GNN's superior performance and its ability to offer clinically relevant, concise, and reliable explanations.

In our implementation, brain ROI was defined according to the AAL atlas, and the methods of functional graph construction and two-stage training strategy were consistent with the original paper.

12) IBGNN

In this work, an interpretable framework has been proposed to analyze disorder-specific ROIs and key connections. This framework is composed of two main components: a brain-network-oriented backbone model designed for disease prediction, and a globally shared explanation generator that emphasizes disorder-specific biomarkers, including salient ROIs and significant connections. Experiments have been conducted using three real-world datasets of brain disorders, and the results have demonstrated that the framework not only achieves exceptional performance but also successfully identifies meaningful biomarkers.

In our implementation, brain ROI was defined according to the AAL atlas, and the methods of functional graph construction were consistent with the original paper. According to the training method of the original article, we first trained the backbone IBGNN model and then applied the learned explanation mask to fine-tune the whole model to obtain the IBGNN+ model.

Supplemental Experimental Procedures. Ablation study

The ablation study of local ROI-GNN module.

In our proposed LGMF-GNN model, the local ROI-GNN module is designed to capture the BOLD signal time-series features of multiple brain ROI and the functional connectivity patterns between them at a refined scale. It aggregates these features to form graph-level embedding for each individual, which are subsequently utilized as feature vectors for the functional graph nodes in the global Subject-GNN. In the ablation experiment concerning this module, we removed the local ROI-GNN from the LGMF-GNN and instead used functional connectivity as the node features for the subject-GNN functional subject graph. Specifically, we computed the Pearson correlation coefficients between the ROI BOLD signals for each individual to obtain a functional connectivity matrix, which was then flattened into a vector using its upper triangle for node features. The results of this ablation are presented in **Table S13**. It is evident that the absence of the local ROI-GNN module resulted in a marked degradation in model performance across all metrics. This indicates that the fine-grained functional connectivity features and BOLD signal time-series information provided by the local ROI-GNN are instrumental in the diagnostic process for depression. Additionally, it is noteworthy that the local ROI-GNN plays a pivotal role in enhancing the interpretability of the proposed model by offering a more granular and flexible understanding. Analyzing the functional connectivity matrix learned by the local ROI-GNN allows us to identify the brain regions and changes in functional connectivity strength that the model focuses on for diagnosis.

The ablation study of different modalities

In our manuscript, we introduce a method that leverages three distinct data modalities: functional, anatomical, and demographic. We achieve multi-modal fusion within the global GNN by integrating three subject graphs, each representing one of the modalities. For the ablation experiments, we omitted the anatomical and demographic modalities by removing their respective subject graphs from the global subject GNN, thus focusing on the fusion of the remaining two modalities. The results of these ablation studies are presented in **Table S14**. It can be seen that without demographic or anatomical modality, the performance of the model can be significantly reduced, which proves the significance of integrating multi-modal information in the diagnosis of depression and the effectiveness of LGMF-GNN multi-modal fusion method.

The ablation study of loss functions

In order to achieve efficient multi-modal fusion, we have designed Modality Independent Loss and Modality Common Loss in our paper:

- Modality Independent Loss ($L_{specific}$): This loss term is designed to ensure that the model can extract features that are specific to each modality. By enforcing independence between the modality-specific embeddings and the modality-common embeddings, the model is encouraged to learn unique characteristics of each modality. This is crucial for scenarios where distinct modalities provide complementary information.
- Modality Common Loss (L_{common}): This loss term is responsible for promoting the similarity between the modality-common embeddings across different modalities. By doing so, it helps the model to identify and extract features that are shared among the modalities. This is particularly important for tasks that require integrating information from multiple sources, as it allows the model to capture and leverage the commonalities that exist across different types of data.

To assess the impact of these loss functions on the performance of our LGMF-GNN model, we conducted ablation study and the result are shown in **Table S15**. It can be seen that the performance of the model decreases after the removal of $L_{specific}$ and L_{common}, which proves the help of the two multimodal fusion loss for model training.

Supplemental Experimental Procedures. Methods for suppressing site effects and data enhancement and ablation study

- **Combat harmonization**

The results above indicate that the functional connectivity matrix derived from fMRI images exhibits notable site effects. To mitigate the site effects during data preprocessing, we employed the Combat harmonization method to promote multi-site coordination. The Combat method is essentially a model based on multiple mixed linear regression, which was originally proposed to suppress the batch effect in genomic studies. Due to the effectiveness of this method in genomic studies, this method was later extended to the study of neuroimaging data to remove site-related bias from neuroimaging data acquired from multiple sites and their derived values[14]. Combat uses empirical Bayes to improve parameter estimation of biological effects and site images to avoid overcorrection of important biological variability during the correction process. In this paper, we set the site index or acquisition protocol index as the batch number, and the subject's sex, age, and disease status as covariates to preserve as much site-independent variance as possible during the correction process. To avoid information leakage, a machine learning form of Combat (https://github.com/sahahn/neuroCombat)[14] was used for harmonization. The Combat model underwent a 10-fold cross-validation process to obtain the coordinated data. The t-SNE plot of the functional connectivity matrix after harmonization is shown in Figure S2b, illustrating a more uniform data distribution across all sites, successful elimination of outliers, and effective reduction of site effects on the data.

- **Data Enhancement**

Site effects are primarily caused by the differences in data distribution among sites, which are due to the use of different instruments and parameters for data collection at each site. As a consequence, the training data fails to meet the assumption of an independent and homogeneous distribution necessary for network training and generalization. Furthermore, data-driven methods like graph neural networks necessitate a significant amount of training data to attain satisfactory generalization performance, thereby demanding effective data augmentation techniques to increase the size of the training data. In this paper, DropEdge[15] and a mixup method on graphs were used for data augmentation. DropEdge, which is commonly used in graph neural networks, perturbs the graph structure by randomly removing a portion of the edges in the adjacency matrix during the training process to achieve the effect of data enhancement, thereby alleviating the overfitting phenomenon and enhancing the model generalization performance. The mixup method proposed in this paper extends the Mixup[16] method commonly used in computer vision to graph neural networks, and perturbation of the graph is achieved by linear interpolation of node features and graph structure. Linear interpolation makes the distribution of training data more uniform, which helps suppress the site effects. From the perspective of graph theory, the enhanced data can be viewed as sampling from different graphons, thus achieving data augmentation and improved generalization ability of the model.

Specifically, the Mixup method used in this paper is implemented according to the following equation:

$$\lambda \sim Bern(a,b), y \in \{0,1\} \tag{1}$$

$$\tilde{X}[y] = \lambda X[y] + (1-\lambda)\bar{X}[y] \tag{2}$$

$$\tilde{A}[y] = \lambda X[y] + (1-\lambda)\bar{A}[y] \tag{3}$$

$$\tilde{Y}[y] = \lambda Y[y] + (1-\lambda)\bar{Y}[y] \tag{4}$$

Where $Bern(a,b)$ represents the Bernoulli distribution with parameters a,b. $X,A,Y,$ and $\tilde{X},\tilde{A},\tilde{Y}$ represents the node features, adjacent matrix and classification labels, respectively. $[\cdot]$ represents indexing according to the original label and $\bar{\cdot}$ indicates random permutation by row.

- **Domain adversarial training**

Based on the pre-experimental results, site effects have a substantial impact on both the training and generalization of the model. To reduce site-specific variation in the local embeddings produced by the local ROI-GNN, a domain classifier and a gradient inversion layer were added to the local ROI-GNN in this paper. Specifically, the domain classifier (a 2-layer MLP) identifies the origin site for each local whole-brain embedding. In addition, during the process of gradient backpropagation, a gradient inversion layer is used to reverse the gradients. Through the minimization of the domain classification loss, the domain classifier can be optimized, and the layers preceding it can be encouraged to generate embeddings that confuse the domain classifier. During the model training phase, the layers before the local ROI-GNN domain classifier and the domain classifier engage in an adversarial process and eventually

mitigate the site variance of the local whole-brain embeddings generated by the model.

- **Domain migration loss**

The training and generalization of the model rely on the assumption that the labeled training nodes are randomly sampled and that the test nodes and training nodes follow the independent and identical distribution (iid). This allows the patterns and information learned from the training set to be successfully applied to the test set. However, in a multi-site scenario, particularly when the training set and test set come from different sites, satisfying the assumption of iid becomes challenging. As a result, site effects can significantly hinder the model's ability to generalize. In such cases, the model is more likely to fit redundant rules from the training data, leading to a loss in generalization performance. Therefore, inspired by the work of Qi Zhu et al[17], this paper used a domain migration loss to migrate GNNs from partial site-biased data to complete graph data containing all sites. Specifically, the distribution bias of GNN is defined as follows: assume that the n node embeddings are $H = \{h_1, h_2, \dots, h_n\}$, from which M nodes are labeled $\{(x_i, y_i)\}$ and represented as $H_{train} = \{h_1, \dots, h_m\}$. Assuming that H and H_{train} originate from two different probability distributions p, q, the distribution bias in GNN can be quantified by the distance metric $d(H, H_{train})$. In the training phase, this paper randomly sampled the same number of embeddings from H_{train} and H_{test} and calculated the Central Moment Discrepancy (CMD) distance between them as the domain migration loss:

$$L_{CMD}(H_{train}, H_{test}) = \frac{1}{b-a} \|E(H_{train}) - E(H_{test})\| + \Sigma_{k=2}^{\infty} \frac{1}{|b-a|^k} \|c_k(H_{train}) - c_k(H_{test})\| \quad (5)$$

During the experimental phase, we systematically integrated these methods and evaluated their efficiency in enhancing the model’s generalization performance using 10-fold cross-validation on the SRPBS dataset. The results are summarized in **Table S16**, which demonstrates that the Combat harmonization, Mixup data augmentation, and adversarial training achieved a significant improvement in model generalization ability.

Supplemental Experimental Procedures. Hyperparameter Selection

To further investigate the impact of the number of neighbors (KNN k) for each node in constructing the K-nearest neighbor graph and the number of network layers for Snowball GCN (Snowball Layer Num) on the performance of the model, we explored the changes in model performance under different hyperparameter and model structure settings using grid search on the SRPBS dataset, as shown in **Figure S8** and **Figure S9**. It can be observed that the model's performance remains relatively stable with the variation of hyperparameters. The model achieves the best performance when the number of neighbors in the K-nearest graph is 10 and the number of network layers in Snowball GCN is 9. These two parameter settings can be considered as influencing the receptive field of LGMF-GNN. When the number of neighboring nodes is small or the network depth is shallow, the network aggregates and updates node representations without accessing enough information from neighboring nodes, leading to insufficient information in the node representations. In contrast, when the number of neighboring nodes is large or the network depth is deep, the network introduces excessive noise information into the node representations, resulting in a decrease in model performance. When the number of neighboring nodes and network layers are appropriately balanced and at a moderate level, the network can effectively aggregate and update information within the desired range, thus avoiding excessive noise and contributing to improved performance.

Supplemental Experimental Procedures. The impact of brain atlases on model performance

In this study we have tried 2 brain atlases, the AAL atlas and the CC200 atlas, to define the region of interest (ROI) in participants' brains. In order to investigate the impact of ROI partitioning on the diagnosis of depression, we examined the changes in the 10-fold cross-validation results of the end-to-end LGMF-GNN model on the SRPBS dataset when using different brain atlases. The experimental results showed that the model exhibited robust performance across different ROI partitioning methods (**Table S23**). Additionally, we found that the model performed better when the ROI partitioning was more generalized. This may be because overly fine-grained ROI partitioning introduces more noise, thereby increasing the risk of model confusion.

Supplemental Experimental Procedures. Model validation on unbalanced dataset

In practice, the incidence rate of depression in the population is roughly 1/5. To thoroughly investigate the model’s generalization capability on unbalanced datasets, we employed the Bootstrap method to sample the validation sets from the 10-fold cross-validation on the SRPBS dataset, creating validations set with a HC to MDD ratio of 4:1 to mimic the real-world distribution. For each fold, we performed 10 Bootstrap samplings, collected the model’s prediction results for performance metric calculation, and then averaged the Bootstrap results across the 10 folds. The outcomes of the Bootstrap experiments are detailed in **Table S24**.

We acknowledge that the experimental results on the imbalanced dataset do not meet the performance standards we had achieved on the balanced dataset. The performance metrics indicate that the model’s ability to accurately identify MDD cases is slightly compromised when the data distribution is skewed towards the HC group. Despite the performance degradation in scenarios with unbalanced class distribution, LGMF-GNN still achieved similar performance to current methods[44,45].

Supplemental References

1. Yan, C.G., Chen, X., Li, L., Castellanos, F.X., Bai, T.J., Bo, Q.J., Cao, J., Chen, G.M., Chen, N.X., Chen, W., et al. (2019). Reduced default mode network functional connectivity in patients with recurrent major depressive disorder. Proc Natl Acad Sci U S A *116*, 9078-9083. 10.1073/pnas.1900390116.
2. Kipf, T.N., and Welling, M. (2016). Semi-supervised classification with graph convolutional networks. arXiv preprint arXiv:1609.02907.
3. Xu, K., Hu, W., Leskovec, J., and Jegelka, S. (2019). How powerful are graph neural networks? International Conference on Learning Representations 2019. ICLR.
4. Veličković, P., Cucurull, G., Casanova, A., Romero, A., Lio, P., and Bengio, Y. (2018). Graph attention networks. International Conference on Learning Representations 2018. ICLR.
5. Li, X., Zhou, Y., Dvornek, N., Zhang, M., Gao, S., Zhuang, J., Scheinost, D., Staib, L.H., Ventola, P., and Duncan, J.S. (2021). BrainGNN: Interpretable Brain Graph Neural Network for fMRI Analysis. Medical Image Analysis *74*, 102233. 10.1016/j.media.2021.102233.
6. Huang, Y., and Chung, A.C.S. (2020). Edge-Variational Graph Convolutional Networks for Uncertainty-Aware Disease Prediction. held in Cham, 2020//. A.L. Martel, P. Abolmaesumi, D. Stoyanov, D. Mateus, M.A. Zuluaga, S.K. Zhou, D. Racoceanu, and L. Joskowicz, eds. (Springer International Publishing), pp. 562-572.
7. Zhang, H., Song, R., Wang, L., Zhang, L., Wang, D., Wang, C., and Zhang, W. (2022). Classification of Brain Disorders in rs-fMRI via Local-to-Global Graph Neural Networks. IEEE Transactions on Medical Imaging, 1-1. 10.1109/TMI.2022.3219260.
8. Xu, J., Bian, Q., Li, X., Zhang, A., Ke, Y., Qiao, M., Zhang, W., Sim, W.K.J., and Gulyás, B. (2024). Contrastive Graph Pooling for Explainable Classification of Brain Networks. IEEE Transactions on Medical Imaging, 1-1. 10.1109/TMI.2024.3392988.
9. Gu, X., Xie, L., Xia, Y., Cheng, Y., Liu, L., and Tang, L. (2023). Autism spectrum disorder diagnosis using the relational graph attention network. Biomedical Signal Processing and Control *85*, 105090. https://doi.org/10.1016/j.bspc.2023.105090.
10. Pan, J., Lin, H., Dong, Y., Wang, Y., and Ji, Y. (2022). MAMF-GCN: Multi-scale adaptive multi-channel fusion deep graph convolutional network for predicting mental disorder. Computers in Biology and Medicine *148*, 105823. https://doi.org/10.1016/j.compbiomed.2022.105823.
11. Zhang, J., Wang, Q., Wang, X., Qiao, L., and Liu, M. (2024). Preserving specificity in federated graph learning for fMRI-based neurological disorder identification. Neural Networks *169*, 584-596. https://doi.org/10.1016/j.neunet.2023.11.004.
12. Zheng, K., Yu, S., and Chen, B. (2024). CI-GNN: A Granger causality-inspired graph neural network for interpretable brain network-based psychiatric diagnosis. Neural Networks *172*, 106147. https://doi.org/10.1016/j.neunet.2024.106147.
13. Cui, H., Dai, W., Zhu, Y., Li, X., He, L., and Yang, C. (2022). Interpretable Graph Neural Networks for Connectome-Based Brain Disorder Analysis. held in Cham, 2022//. L. Wang, Q. Dou, P.T. Fletcher, S. Speidel, and S. Li, eds. (Springer Nature Switzerland), pp. 375-385.
14. Fortin, J.-P., Cullen, N., Sheline, Y.I., Taylor, W.D., Aselcioglu, I., Cook, P.A., Adams, P., Cooper, C., Fava, M., McGrath, P.J., et al. (2018). Harmonization of cortical thickness measurements across scanners and sites. NeuroImage *167*, 104-120. https://doi.org/10.1016/j.neuroimage.2017.11.024.
15. Rong, Y., Huang, W.-b., Xu, T., and Huang, J. (2019). DropEdge: Towards Deep Graph Convolutional Networks on Node Classification. held in New Orleans, Louisiana, United States, (OpenReview.net).
16. Zhang, H., Cisse, M., Dauphin, Y.N., and Lopez-Paz, D. (2017). mixup: Beyond Empirical Risk Minimization. arXiv:1710.09412. 10.48550/arXiv.1710.09412.
17. Zhu, Q., Ponomareva, N., Han, J., and Perozzi, B. (2021). Shift-Robust GNNs: Overcoming the Limitations of Localized Graph Training Data. arXiv:2108.01099. DOI:10.48550/arXiv.2108.01099.
18. Depping, M.S., Schmitgen, M.M., Kubera, K.M., and Wolf, R.C. (2018). Cerebellar Contributions to Major Depression. Frontiers in Psychiatry *9*. 10.3389/fpsyt.2018.00634.
19. Xu, L.-Y., Xu, F.-C., Liu, C., Ji, Y.-F., Wu, J.-M., Wang, Y., Wang, H.-B., and Yu, Y.-Q.

(2017). Relationship between cerebellar structure and emotional memory in depression. Brain and Behavior *7*, e00738. https://doi.org/10.1002/brb3.738.
20. Fateh, A.A., Long, Z., Duan, X., Cui, Q., Pang, Y., Farooq, M.U., Nan, X., Chen, Y., Sheng, W., Tang, Q., and Chen, H. (2019). Hippocampal functional connectivity-based discrimination between bipolar and major depressive disorders. Psychiatry Research: Neuroimaging *284*, 53-60. https://doi.org/10.1016/j.pscychresns.2019.01.004.
21. Fan, J., Gao, F., Wang, X., Liu, Q., Xia, J., Han, Y., Yi, J., Tan, C., and Zhu, X. (2023). Right amygdala-right precuneus connectivity is associated with childhood trauma in major depression patients and healthy controls. Soc Cogn Affect Neurosci *18*. 10.1093/scan/nsac064.
22. Jacob, Y., Morris, L.S., Verma, G., Rutter, S.B., Balchandani, P., and Murrough, J.W. (2022). Altered hippocampus and amygdala subregion connectome hierarchy in major depressive disorder. Translational Psychiatry *12*, 209. 10.1038/s41398-022-01976-0.
23. Hu, J., Liu, J., Liu, Y., Wu, X., Zhuang, K., Chen, Q., Yang, W., Xie, P., Qiu, J., and Wei, D. (2021). Dysfunction of the anterior and intermediate hippocampal functional network in major depressive disorders across the adult lifespan. Biological Psychology *165*, 108192. https://doi.org/10.1016/j.biopsycho.2021.108192.
24. Shengli, C., Yingli, Z., Zheng, G., Shiwei, L., Ziyun, X., Han, F., Yingwei, Q., and Gangqiang, H. (2022). An aberrant hippocampal subregional network, rather than structure, characterizes major depressive disorder. Journal of Affective Disorders *302*, 123-130. https://doi.org/10.1016/j.jad.2022.01.087.
25. Kaymak, S.U., Demir, B., Şentürk, S., Tatar, I., Aldur, M.M., and Uluğ, B. (2010). Hippocampus, glucocorticoids and neurocognitive functions in patients with first-episode major depressive disorders. European Archives of Psychiatry and Clinical Neuroscience *260*, 217-223. 10.1007/s00406-009-0045-x.
26. Cole, J., Toga, A.W., Hojatkashani, C., Thompson, P., Costafreda, S.G., Cleare, A.J., Williams, S.C., Bullmore, E.T., Scott, J.L., Mitterschiffthaler, M.T., et al. (2010). Subregional hippocampal deformations in major depressive disorder. J Affect Disord *126*, 272-277. 10.1016/j.jad.2010.03.004.
27. Jun, X., Jun, C., Yicheng, Z., Jingfeng, Z., Bo, Y., Liming, X., and Chengyuan, W. (2004). Volumetric MRI analysis of the amygdala and hippocampus in subjects with major depression. Journal of Huazhong University of Science and Technology [Medical Sciences] *24*, 500-502. 10.1007/BF02831120.
28. Cole, J., Costafreda, S.G., McGuffin, P., and Fu, C.H.Y. (2011). Hippocampal atrophy in first episode depression: A meta-analysis of magnetic resonance imaging studies. Journal of Affective Disorders *134*, 483-487. 10.1016/j.jad.2011.05.057.
29. MacMaster, F.P., and Kusumakar, V. (2004). Hippocampal volume in early onset depression. BMC Medicine *2*, 2. 10.1186/1741-7015-2-2.
30. Chen, L., Wang, Y., Niu, C., Zhong, S., Hu, H., Chen, P., Zhang, S., Chen, G., Deng, F., Lai, S., et al. (2018). Common and distinct abnormal frontal-limbic system structural and functional patterns in patients with major depression and bipolar disorder. NeuroImage: Clinical *20*, 42-50. https://doi.org/10.1016/j.nicl.2018.07.002.
31. Frodl, T., Reinhold, E., Koutsouleris, N., Reiser, M., and Meisenzahl, E.M. (2010). Interaction of childhood stress with hippocampus and prefrontal cortex volume reduction in major depression. Journal of Psychiatric Research *44*, 799-807. https://doi.org/10.1016/j.jpsychires.2010.01.006.
32. Liu, Y., Chen, Y., Liang, X., Li, D., Zheng, Y., Zhang, H., Cui, Y., Chen, J., Liu, J., and Qiu, S. (2020). Altered Resting-State Functional Connectivity of Multiple Networks and Disrupted Correlation With Executive Function in Major Depressive Disorder. Frontiers in Neurology *11*. 10.3389/fneur.2020.00272.
33. Sun, J.-f., Chen, L.-m., He, J.-k., Wang, Z., Guo, C.-l., Ma, Y., Luo, Y., Gao, D.-q., Hong, Y., Fang, J.-l., and Xu, F.-q. (2022). A Comparative Study of Regional Homogeneity of Resting-State fMRI Between the Early-Onset and Late-Onset Recurrent Depression in Adults. Frontiers in Psychology *13*. 10.3389/fpsyg.2022.849847.
34. Long, Z., Duan, X., Wang, Y., Liu, F., Zeng, L., Zhao, J.-p., and Chen, H. (2015). Disrupted structural connectivity network in treatment-naive depression. Progress in Neuro-Psychopharmacology and Biological Psychiatry *56*, 18-26. https://doi.org/10.1016/j.pnpbp.2014.07.007.
35. Winter, N.R., Leenings, R., Ernsting, J., Sarink, K., Fisch, L., Emden, D., Blanke, J., Goltermann, J., Opel, N., Barkhau, C., et al. (2022). Quantifying Deviations of Brain

Structure and Function in Major Depressive Disorder Across Neuroimaging Modalities. JAMA Psychiatry *79*, 879-888. 10.1001/jamapsychiatry.2022.1780.
36. Xiong, Y., Chen, R.-S., Wang, X.-Y., Li, X., Dai, L.-Q., and Yu, R.-Q. (2022). Cerebral blood flow in adolescents with drug-naive, first-episode major depressive disorder: An arterial spin labeling study based on voxel-level whole-brain analysis. Frontiers in Neuroscience *16*. 10.3389/fnins.2022.966087.
37. Qiao, J., Tao, S., Wang, X., Shi, J., Chen, Y., Tian, S., Yao, Z., and Lu, Q. (2020). Brain functional abnormalities in the amygdala subregions is associated with anxious depression. Journal of Affective Disorders *276*, 653-659. https://doi.org/10.1016/j.jad.2020.06.077.
38. Liu, P., Tu, H., Zhang, A., Yang, C., Liu, Z., Lei, L., Wu, P., Sun, N., and Zhang, K. (2021). Brain functional alterations in MDD patients with somatic symptoms: A resting-state fMRI study. Journal of Affective Disorders *295*, 788-796. https://doi.org/10.1016/j.jad.2021.08.143.
39. Keller, M., Mendoza-Quiñones, R., Cabrera Muñoz, A., Iglesias-Fuster, J., Virués, A.V., Zvyagintsev, M., Edgar, J.C., Zweerings, J., and Mathiak, K. (2022). Transdiagnostic alterations in neural emotion regulation circuits – neural substrates of cognitive reappraisal in patients with depression and post-traumatic stress disorder. BMC Psychiatry *22*, 173. 10.1186/s12888-022-03780-y.
40. Zhang, L., Wei, X., and Zhao, J. (2022). Amplitude of Low-Frequency Oscillations in First-Episode Drug-Naive Patients with Major Depressive Disorder: A Resting State Functional Magnetic Resonance Imaging Study. Neuropsychiatric Disease and Treatment *Volume 18*, 555-561. 10.2147/ndt.s348683.
41. Wu, X., Wang, L., Jiang, H., Fu, Y., Wang, T., Ma, Z., Wu, X., Wang, Y., Fan, F., Song, Y., and Lv, Y. (2023). Frequency-dependent and time-variant alterations of neural activity in post-stroke depression: A resting-state fMRI study. NeuroImage: Clinical *38*, 103445. https://doi.org/10.1016/j.nicl.2023.103445.
42. Sutoko, S., Atsumori, H., Obata, A., Funane, T., Kandori, A., Shimonaga, K., Hama, S., Yamawaki, S., and Tsuji, T. (2020). Lesions in the right Rolandic operculum are associated with self-rating affective and apathetic depressive symptoms for post-stroke patients. Scientific Reports *10*, 20264. 10.1038/s41598-020-77136-5.
43. Ni, S., Gao, S., Ling, C., Jiang, J., Wu, F., Peng, T., Sun, J., Zhang, N., and Xu, X. (2023). Altered brain regional homogeneity is associated with cognitive dysfunction in first-episode drug-naive major depressive disorder: A resting-state fMRI study. Journal of Affective Disorders *343*, 102-108. https://doi.org/10.1016/j.jad.2023.10.003.
44. Mousavian, M., Chen, J., and Greening, S. (2018). Feature Selection and Imbalanced Data Handling for Depression Detection. held in Cham, 2018//. S. Wang, V. Yamamoto, J. Su, Y. Yang, E. Jones, L. Iasemidis, and T. Mitchell, eds. (Springer International Publishing), pp. 349-358.
45. Mousavian, M., Chen, J., Traylor, Z., and Greening, S. (2021). Depression detection from sMRI and rs-fMRI images using machine learning. Journal of Intelligent Information Systems *57*, 395-418. 10.1007/s10844-021-00653-w.
