# Supplementary material for: An objective quantitative diagnosis of depression using a local-to-global multimodal fusion graph neural network
Source: Patterns (N Y). 2024 Nov 4;5(12):101081. doi: 10.1016/j.patter.2024.101081 (PMC11701859; doi:10.1016/j.patter.2024.101081)
Supplement: Document S2. Article plus supplemental information [file mmc2.pdf]

Article

Patterns

An objective quantitative diagnosis of depression using a local-to-global multimodal fusion graph neural network

Highlights

- Local-global network architecture offers a comprehensive view on depression diagnosis
- Integrating functional, structural, and demographic data improves depression diagnosis
- Interpretable AI identifies potential digital biomarkers for depression diagnosis
- Achieves superior performance on depression diagnosis in a multi-center scenario

Authors

Shuyu Liu, Jingjing Zhou,
Xuequan Zhu, ..., Ling Zhang,
Gang Wang, Cheng Jin

Correspondence

chengjin520@sjtu.edu.cn (C.J.),
gangwangdoc@ccmu.edu.cn (G.W.),
zhangling@ccmu.edu.cn (L.Z.),
wangyanfeng@sjtu.edu.cn (Y.W.)

In brief

This study introduces an artificial intelligence (AI) system using local-global multimodal fusion graph neural networks for depression diagnosis. The system integrates functional and structural neuroimaging with health records, demonstrating state-of-the-art performance in diagnosing depression and its subtypes. Built and validated on large-scale, multi-center cohorts with 2,442 participants, the system reveals abnormal brain regions and connectivity patterns, along with digital structural features in depression patients. This advance contributes to the pursuit of objective diagnostic markers and better clinical diagnosis.

Liu et al., 2024, Patterns *5*, 101081
December 13, 2024 © 2024 The Author(s). Published by Elsevier Inc.
https://doi.org/10.1016/j.patter.2024.101081

CellPress

Patterns

CellPress
OPEN ACCESS

Article

An objective quantitative diagnosis of depression using a local-to-global multimodal fusion graph neural network

Shuyu Liu,[1,10] Jingjing Zhou,[2,4,5,10] Xuequan Zhu,[2,10] Ya Zhang,[3,6,10] Xinzhu Zhou,[2] Shaoting Zhang,[3] Zhi Yang,[2] Ziji Wang,[7] Ruoxi Wang,[1] Yizhe Yuan,[1] Xin Fang,[1] Xiongying Chen,[2] DIRECT Consortium,[9] Yanfeng Wang,[3,6,*] Ling Zhang,[2,*] Gang Wang,[2,4,5,*] and Cheng Jin[1,2,3,8,11,*]

[1]Medical Robot Research Institute, School of Biomedical Engineering, Shanghai Jiao Tong University, Shanghai 200240, China
[2]Beijing Key Laboratory of Mental Disorders, National Clinical Research Center for Mental Disorders & National Center for Mental Disorders, Beijing Anding Hospital & Advanced Innovation Center for Human Brain Protection, Capital Medical University, Beijing 100088, China
[3]Shanghai Artificial Intelligence Laboratory, Shanghai 200232, China
[4]CAS Key Laboratory of Behavioral Science, Institute of Psychology, Beijing 100101, China
[5]Department of Psychology, University of Chinese Academy of Sciences, Beijing 100101, China
[6]School of Electronic Information and Electronical Engineering, Shanghai Jiao Tong University, Shanghai 200240, China
[7]Department of Cognitive Science, Swarthmore College, Philadelphia, PA 19081, USA
[8]Stanford University School of Medicine, Ground Floor, 875 Lake Wilbur Drive, Stanford, CA 94305-5847, USA
[9]Further details can be found in the supplemental information
[10]These authors contributed equally
[11]Lead contact
*Correspondence: wangyanfeng@sjtu.edu.cn (Y.W.), zhangling@ccmu.edu.cn (L.Z.), gangwangdoc@ccmu.edu.cn (G.W.), chengjin520@sjtu.edu.cn (C.J.)
https://doi.org/10.1016/j.patter.2024.101081

THE BIGGER PICTURE Major depressive disorder (MDD) presents a multifaceted challenge to global mental health, given its intricate etiology involving social, psychological, and biological determinants. There is a lack of definitive diagnostic markers, and doctors therefore rely heavily on subjective methods to diagnose individuals. Interest in artificial intelligence (AI)-powered diagnostic methods is increasing because of their potential to offer a more comprehensive and objective evaluation. Current AI methods, however, often neglect the complex interplay of functional, structural, and demographic factors that characterize MDD. They seldom integrate insights from both in-depth brain region analysis and broad population-level associations. Moreover, there is a lack of strategies that can interpret AI models and identify diagnostic markers. There is, therefore, an urgent need to improve network architectures, develop effective multimodal fusion strategies, and enhance model interpretability.

SUMMARY

This study developed an artificial intelligence (AI) system using a local-global multimodal fusion graph neural network (LGMF-GNN) to address the challenge of diagnosing major depressive disorder (MDD), a complex disease influenced by social, psychological, and biological factors. Utilizing functional MRI, structural MRI, and electronic health records, the system offers an objective diagnostic method by integrating individual brain regions and population data. Tested across cohorts from China, Japan, and Russia with 1,182 healthy controls and 1,260 MDD patients from 24 institutions, it achieved a classification accuracy of 78.75%, an area under the receiver operating characteristic curve (AUROC) of 80.64%, and correctly identified MDD subtypes. The system further discovered distinct brain connectivity patterns in MDD, including reduced functional connectivity between the left gyrus rectus and right cerebellar lobule VIIB, and increased connectivity between the left Rolandic operculum and right hippocampus. Anatomically, MDD is associated with thickness changes of the gray and white matter interface, indicating potential neuropathological conditions or brain injuries.

This is an open access article under the CC BY license (http://creativecommons.org/licenses/by/4.0/).

INTRODUCTION

Major depressive disorder (MDD) is one of the most common psychiatric disorders, inflicting severe symptoms that may significantly impact a person's physical and mental well-being, social functioning, and quality of life.[1] This disorder epitomizes the quintessential features of depressive disorder, characterized by persistent and profound sadness, loss of interest or pleasure, and other cognitive and physical disruptions. These symptoms, persisting for a minimum of 2 weeks, as defined by standard diagnostic criteria, not only underscore the distinct characteristics of MDD but also highlight its significance as a focal point in clinical research and intervention. The number of MDD patients worldwide has increased by approximately 18% in the past decade, and currently, an estimated 185 million people suffer from the disease.[2] Unfortunately, this growing epidemic often remains shrouded or invisible, with sufferers facing stigmatization and exclusion. They frequently endure their plight in isolation rather than seeking assistance. Without proper treatment, the disease can get worse and last longer. In severe cases, it could lead to self-harm or suicide.[3,4] Therefore, the early and precise diagnosis of MDD is vital in preventing severe outcomes and minimizing financial and emotional burdens.

The diagnosis of MDD follows a structured clinical evaluation process, primarily guided by the *Diagnostic and Statistical Manual of Mental Disorders, Fifth Edition* (*DSM*-5)[5] and *International Classification of Diseases, 11th Revision* (ICD-11)[6] criteria. This process typically involves a clinical interview to assess mental health history and symptomatology; the use of diagnostic scales such as the Hamilton Depression Scale (HAM-D),[7,8] the Beck Depression Inventory (BDI),[9] and Patient Health Questionnaire-9[10] to quantify symptom severity; the exclusion of other potential causes for depressive symptoms; and the evaluation of symptom duration and frequency. This approach, however, depends inherently on the subjective judgment of clinicians and self-reported measures from depression scales. As a result, it is fraught with challenges, including low detection rates, high misdiagnosis risk, and unsatisfactory levels of accuracy.[11]

Recent advances in neuroimaging research have elucidated the intricate structural and functional alterations in some brain regions associated with MDD. Structural studies have converged to implicate the prefrontal cortex and anterior cingulate in the pathology of MDD, with evidence of genetic variants, neuroinflammatory markers, reduced neurogenesis, and gray matter volume (GMV) alterations in these regions.[6,12] Additionally, hippocampal atrophy and thinner cortical gray matter have been observed in the orbitofrontal cortex, anterior and posterior cingulate, and insula in MDD patients, suggesting potential structural disconnectivity.[13,14] Despite these findings, the effect sizes for structural brain differences are generally small, and their predictive value at the individual level remains limited.[12] Functionally, MDD is characterized by altered connectivity within the salience network, frontoparietal network, and default mode network.[6,15] Notably, the salience network, which includes the amygdala and anterior cingulate cortex, tends to show increased activity in response to emotional stimuli, suggesting a hyperresponsive emotional processing system.[16–18] Concurrently, the default mode network, implicated in self-referential thoughts and rumination, often exhibits altered connectivity, which may contribute to the cognitive and affective symptoms of MDD.[15,19] Additionally, the frontoparietal network, critical for several higher-order cognitive processes, is frequently found to be hypoactive, potentially underlying the executive dysfunction observed in depression.[20,21]

Despite these findings, the underlying pathophysiology of depression remains largely elusive, and there are currently no internationally recognized effective molecular or imaging biomarkers, hampering research in both its diagnosis and treatment. In this context, it is appealing to establish an objective and quantitative system for the automatic diagnosis of MDD and further guide the uncovering and understanding of pathological mechanisms and markers of depression.

Existing research underscores the critical role that neural circuits play in both causing and characterizing brain disorders.[22,23] As a commonly used non-invasive imaging technique, MRI serves as an effective tool for examining brain structure and functionality. Resting-state functional MRI (rs-fMRI) and structural MRI (sMRI) are two commonly used modalities for investigating brain function and anatomy, respectively. rs-fMRI measures brain activity by detecting changes associated with blood flow, revealing disrupted functional brain activities in psychiatric disorders. At the same time, sMRI is adept at revealing finer anatomical information due to its high spatial resolution,[24] providing an effective tool for assessing anatomical alterations in the brain. Additionally, clinical information such as gender, age, and education can provide valuable information for the diagnosis of depression at the demographic level. The fusion of these multimodal data is expected to provide more comprehensive information for the diagnosis of MDD.

A majority of current MDD diagnosis methods based on MRI data adhere to the following pipeline: (1) feature engineering, including feature selection using either pre-trained weights, statistical analysis,[25] or prior knowledge, and feature reduction via clustering-based or decomposition-based techniques; (2) disease diagnosis based on the selected features, using classifiers such as support vector machine,[26–28] Gaussian process classifier,[29] decision tree,[30,31] Naive Bayes,[25] and emerging deep learning models.[32,33] Nevertheless, such traditional approaches have some intrinsic shortcomings. On the one hand, coarse and empirical feature extraction may lead to suboptimal performance in subsequent stages of classification. On the other hand, these methods failed to adequately capture the topological information of the brain network, which is essential for understanding functional connectivity and signal transmission across various brain regions.

Graph neural networks (GNNs) represent data as graphs composed of nodes and edges and iteratively update the representations of nodes by exchanging information with their neighbors through pairwise message passing. Recent studies have successfully employed GNN in investigating mental disorders, including Alzheimer disease (AD),[34–36] autism spectrum disorder (ASD),[37,38] and MDD.[39,40] The correspondence between graph structure and brain anatomy, alongside the similarity between the message-passing mechanisms and the physiological functions of the brain, highlights the potential of GNN to more effectively retrieve the underlying information of the brain from MRI images.

CellPress
OPEN ACCESS

Generally, GNN-based methods for mental disease diagnosis can be divided into two categories: regional GNN methods and subject GNN methods. Regional GNN methods recognize regions of interest (ROIs) in the brain as nodes and links between different ROIs as edges, thus turning disease diagnosis into a graph-level classification task.[41–43] These approaches offer a fine-grained analysis of brain regions, as defined by the ROIs, and their interactions. For instance, specific brain regions associated with emotional regulation, such as the amygdala and prefrontal cortex, are often treated as nodes. The construction of edges based on neural connections or functional correlations between these regions allows for a detailed analysis of local brain circuitry. This fine-grained approach is highly effective in capturing disease-related local brain regions and biomarkers, enabling precise identification of neural abnormalities related to the etiology of depression. However, it may overlook the inter-individual relationships and variations that could influence the manifestation of the disorder. In contrast, the subject GNN methods incorporate the population aspect of mental disorders, thus turning disease diagnosis into a node-level classification task. In these methods, each node corresponds to an individual and each edge encodes the relationship between two subject nodes evaluated by imaging data (e.g., MRI, computed tomography) or demographic data (e.g., age, gender, acquisition site, educational attainment).[39,40] Here, the integration of diverse demographic and imaging data provides a broader perspective. By considering the relationships between individuals, these methods can identify population-level patterns and trends that might be associated with the occurrence and development of depression. However, the graph modeling method used in this approach may limit interpretability and pose challenges in specific biomarker extraction.

In the realm of clinical application, the two methods exhibit distinct focal points. Regional GNN methods are tailored to an individual-centric perspective, diagnosing diseases by conducting a granular examination of the signal characteristics and functional connectivity features within discrete brain regions. Subject GNN methods adopt a population-based perspective, aiming to achieve diagnostic objectives through the integration and comparative analysis of the features of the study subject with those of similar individuals within the larger population. In essence, regional GNN and subject GNN can be likened to symptomatic diagnosis and epidemiological diagnosis, respectively, mirroring the two fundamental paradigms of medical diagnostics. The combination of regional and subject GNN methods offers the potential to leverage the strengths of both methods for a better understanding of the etiology and quantitative diagnosis of depression.

From a mechanistic standpoint, the differences between regional and subject GNN methods reflect varying levels of granularity in understanding depression. Regional GNN methods offer insights into the local, intracranial dynamics, providing a window into the direct physiological correlates of depression. Subject GNN methods, however, shed light on the interplay between individual differences and environmental factors, illuminating the complex web of influences that contribute to the manifestation of depression.

In the context of advancements in machine learning, regional GNN and subject GNN methods drive the development and innovation of algorithms in distinct and complementary directions. Regional GNN methods push the boundaries of graph-based learning by requiring models that can handle intricate and variable graph structures. Subject GNN methods challenge the field by demanding algorithms with the capacity to incorporate varying data types and capture the nuanced relationships within a population graph.

In summary, the current GNN-based approaches for mental disease diagnosis, namely regional GNN and subject GNN, offer complementary perspectives in understanding and diagnosing depression, indicating the potential for integrating Regional GNN and Subject GNN into a local-global network structure. This fusion is expected to enhance our understanding of the etiology of depression, improve diagnostic accuracy, and advance machine learning techniques in mental health diagnostics.

Regarding graph structure, recent research has revealed that the topological information within fMRI-based graphs is crucial to the performance of a GNN on MDD classification.[44] However, in many studies, the graph structure is initialized in a rudimentary manner. The construction of local graphs based on MRI data typically begins with the selection of a brain atlas that defines a set of ROIs as nodes. Then, the fMRI blood-oxygen-level-dependent (BOLD) signal series of each ROI is extracted for generating edges. Finally, the edge weights are calculated between each node pair using metrics such as Pearson correlation and partial correlation. Despite its prevalence in the existing literature,[38,43,45] this method features two flaws. First, the simple linear correlation ignores the temporal dynamics of fMRI signals, which play a vital role in reflecting brain activity and connectivity. Second, the constructed graph remains static during model training and fails to be optimized according to the target task. Given these limitations, the performance of GNNs is often less than optimal. Therefore, it is necessary to find a method that allows the graph structure defined by the adjacency matrix and the network parameters to train jointly, facilitating the dynamic adaptation of graph structure and active learning.

To synthesize the merits of the regional (local) and subject (global) GNN methods mentioned above while mitigating their limitations, and to construct a flexible and reasonable graph structure with features in multimodalities, a local-to-global multimodal fusion GNN (LGMF-GNN) was proposed for the objective quantitative diagnosis of MDD. Specifically, a local ROI GNN is utilized to generate graph-level embedding from the time series signal of various brain ROIs described by rs-fMRI. Then, a global subject GNN further fuses local functional connectivity features described by the above local embedding, anatomical features from sMRI, and demographic features from non-imaging data into a unified space. A final diagnosis that leverages the local-global and multimodal comprehensive information is then generated. By transitioning from regional brain graphs to subject graphs, this framework facilitates the extraction of individualized fine-grained features and the integration of multimodal data at the population level, culminating in a synergistic improvement and a progressive methodology for intelligent diagnosis.

This study involves large-scale, multi-center datasets collected from 24 institutions in 3 nations. Model development and internal validation were conducted using the Japanese Strategic Research Program for the Promotion of Brain Science (SRPBS) and REST-meta-MDD datasets, which include a total

Table 1. Demographic and clinical characteristics of included multi-site participants

Dataset and variable	Depressive disorder	Healthy control
Anding		
sample size, N	196	177
age, y, mean ± SD	29.22 ± 8.40	27.63 ± 6.96
female sex, N (%)	148 (75.5)	116 (65.5)
episode status		
first episode, N (%)	93 (47.4)	–
recurrent, N (%)	96 (49.0)	–
unknown, N (%)	7 (3.6)	–
medication status		
drug naive, N (%)	169 (86.2)	–
treated, N (%)	16 (8.2)	–
unknown, N (%)	11 (5.6)	–
duration of illness, months, mean ± SD	5.00 ± 5.21	–
education, y, mean ± SD	15.69 ± 2.62	16.48 ± 2.75
SRPBS		
sample size, N	229	228
age, y, mean ± SD	43.05 ± 13.49	44.07 ± 14.89
female sex, N (%)	104 (45.4)	142 (62.3)
BDI-II, mean ± SD	27.43 ± 14.14	–
REST-meta-MDD		
sample size, N	814	756
age, y, mean ± SD	34.45 ± 11.61	34.64 ± 13.18
female sex, N (%)	519 (63.6)	446 (59.0)
episode status		
first episode, N (%)	404 (49.6)	–
recurrent, N (%)	208 (25.6)	–
unknown, N (%)	202 (24.8)	–
medication status		
drug naive, N (%)	300 (36.9)	–
treated, N (%)	221 (27.1)	–
unknown, N (%)	293 (29.3)	–
duration of illness, months, mean ± SD	39.44 ± 61.40	–
HAM-D, mean ± SD	21.17 ± 6.54	–
education, y, mean ± SD	11.95 ± 3.38	13.56 ± 3.42
OpenNeuro		
sample size, N	21	21
age, y, mean ± SD	32.04 ± 9.38	33.81 ± 8.49
female sex, N (%)	17 (81.0)	15 (71.4)
BDI-R, mean ± SD	25.11 ± 10.09	4.5 ± 4.64

HAM-D, Hamilton Depression Scale; BDI-II, Beck Depression Inventory-Second Edition; BDI-R, Beck Depression Inventory-Russian versions.

of 2,027 individuals, achieving a 10-fold cross-validation (CV) area under the receiver operating characteristic curve (AUROC) of 80.64% ± 5.74% and a leave-one-subject-out cross-validation (LOSO CV) AUROC of 73.71% ± 4.12%. External independent testing was performed using the Anding and OpenNeuro datasets, achieving AUROCs of 72.91% and 70.30%, respectively. In comparison with the state-of-the-art (SOTA) methods like BrainGNN on the identical dataset, the proposed model showed improvements of at least 5.46% in accuracy and 7.60% in AUROC. Furthermore, through in-depth interpretation research, we identified a collection of potential biomarkers that describe relational features in MDD patients, such as functional alterations in brain regions like the prefrontal cortex and hippocampus and structural alterations described by GMV and white matter volume. Our study paves the way for an objective quantitative diagnosis of MDD. All the data and code have been made accessible to the public.

RESULTS

Data acquisition

This study employed four datasets comprising MDD patients and healthy controls (HCs), as outlined in Table 1. The SRPBS dataset (229 MDDs vs. 228 HCs) and the REST-meta-MDD dataset (814 MDDs vs. 756 HCs) were used for training and internal validation, while the Anding dataset (196 MDDs vs. 177 HCs) and the OpenNeuro dataset (21 MDDs vs. 21 HCs) were used for external independent testing. Collectively, these datasets incorporate data from 2,242 participants, each providing a complete set of information across three modalities: fMRI, sMRI, and demographic characteristics.

Hierarchical graph structure for MDD diagnosis from both individual and population perspectives

Previous studies have often been limited to a single perspective when making the diagnosis, which results in suboptimal diagnostic performance and inadequate explanations of the underlying mechanisms. The LGMF-GNN was proposed for accurate and interpretable MDD diagnosis. LGMF-GNN generates the diagnosis by integrating information from both individual and population levels, as well as multiple data modalities that reflect brain structural and functional status (illustrated in Figure 1.). Specifically, our method employs two complementary sub-models: the ROI GNN and the subject GNN. The ROI GNN captures local brain information by representing the brain as a region of interest (ROI) graph, where brain regions serve as nodes and their functional interactions serve as edges. This sub-model focuses on the localized individual brain region-level perspective. Conversely, the subject GNN analyzes the embedding information among subjects from the global perspective by operating on the subject graph. In this graph, subjects and their relationships are represented as nodes and edges, respectively, and the edge weights are calculated based on the similarity of functional, anatomical, and demographic information. This sub-model adopts the population-level perspective. By integrating these sub-models and diverse data modalities, the LGMF-GNN achieves robust and precise MDD diagnoses.

In our proposed local-global model, the initial step is constructing ROI graphs for learning high-quality subject brain embeddings. A learnable adjacency matrix is derived by analyzing the ROI BOLD time series (see Figure 1B and the section "local ROI GNN" in the methods section for details). The node attributes are defined as the corresponding columns of the functional connectivity matrix obtained from rs-fMRI images. To generate

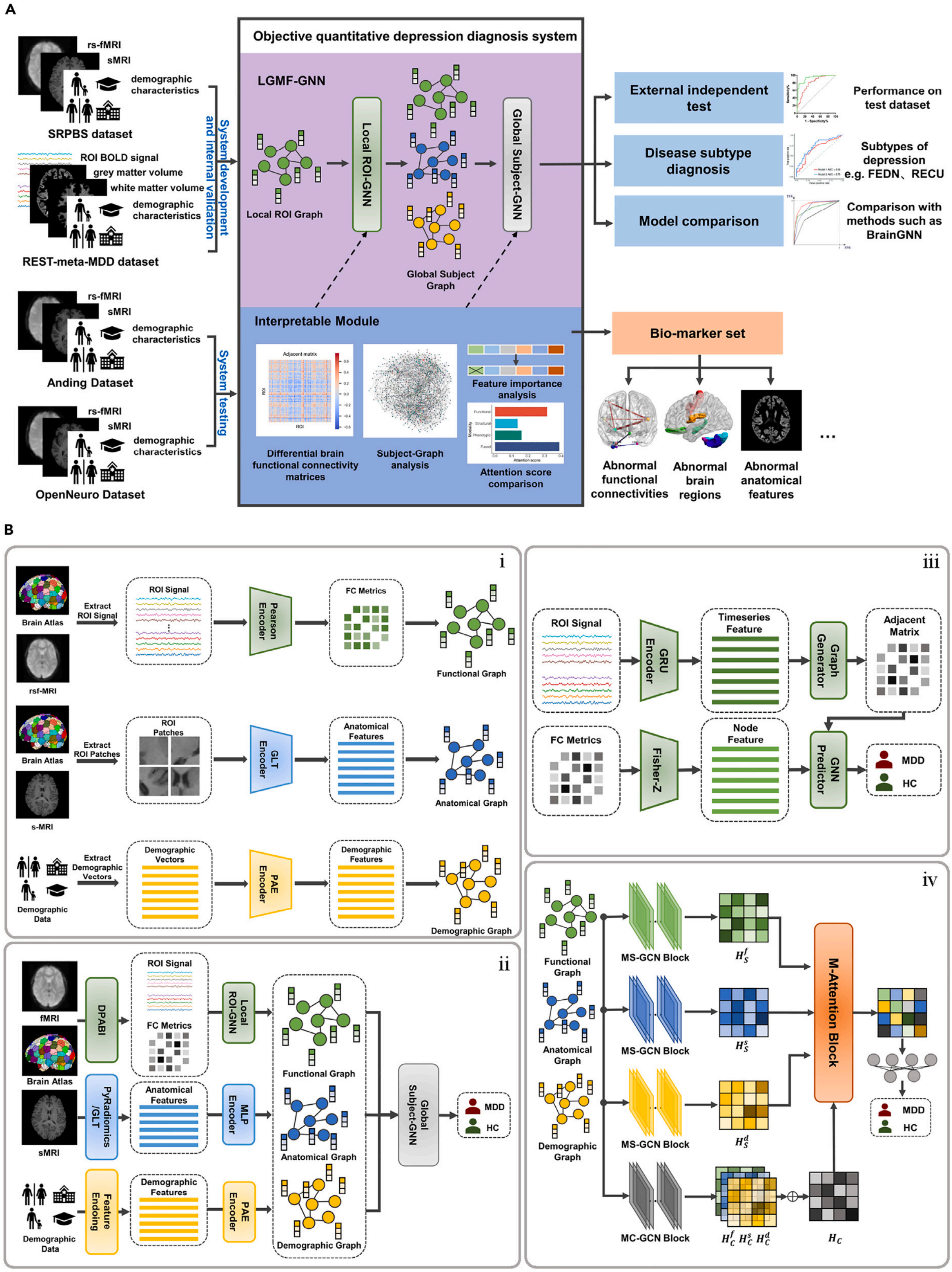

(legend on next page)

an ROI graph representation, a graph convolutional network (GCN) with the attention mechanism is implemented in the local ROI GNN to aggregate information across ROIs with emphasis. As shown in Figure 1B, a regional embedding is generated by the gated recurrent unit (GRU) encoder based on the BOLD time series. Then, the graph generator takes the embedding as input and outputs a learnable adjacent matrix for each subject. Subsequently, the GNN predictor applies the attention mechanism to the learned graph structure and node features to acquire the local graph embeddings and local classification results. The local embeddings are then employed as the initial functional node features of the global subject GNN. To extract features from T1 MRI images and demographic data, we designed the global-local transformer (GLT) encoder and pairwise association encoder (PAE) to encode the raw anatomical and demographic data of each individual into one-dimensional feature vectors (see Figure 1B and the section "feature extraction" in the methods section). In the subject GNN, features from functional, anatomical, and demographic data are modeled as node attributes of the three subject graphs (illustrated in Figure 1B). In the population view, the modality-specific (MS)-GCN block is designed to generate modal-specific representations, while the modality-common (MC)-GCN block is designed to distill a modal-common representation. The multimodal (M)-Attention block refines these representations to produce the final representation, which contains the most important and expressive information from all three modalities. Finally, a multi-layer perceptron (MLP) serves as a classifier to produce the final global prediction. An enriched elucidation of the model structure can be obtained from the methods section.

Performance on the SRPBS dataset

We evaluated the prediction performance of the proposed system as an MDD vs. HC binary classification task using five metrics: accuracy (ACC), the area under the receiver operating characteristic curve (AUROC), precision, recall, and F1-score. We evaluated the diagnostic capabilities of the local and global networks separately by examining the single-perspective diagnosis result of the two models on the SRPBS dataset. This dataset contains 229 MDDs and 228 HCs from six sites (Table 1). For the local ROI GNN, we partitioned the brain into ROIs using both automated anatomical labeling (AAL) and Craddock200 (CC200) brain atlases and conducted separate experiments for each. For the global subject GNN, we compared the 10-fold CV results obtained both with and without the inclusion of structural features derived from the T1 sMRI images. The independent performances of the two models are detailed in Table 2–1. When local ROI GNN and global subject GNN operate independently, the model's performance, as indicated by ACC and AUROC, exhibits a variation of less than 2%. We have also conducted an ablation study to assess the role of the ROI GNN module, and the experimental results are shown in Table S13.

Furthermore, we conducted a 10-fold CV on local and global networks in a two-stage fashion. Specifically, we incorporated the embedding obtained by the local ROI GNN into the global model for the construction of the functional subject graph, thus linking the local network with the global network as the two-stage trained LGMF-GNN. To optimize the mode of local-global feature fusion and multimodal fusion, six experiments were designed, differing in the inclusion of structural modality (T1 sMRI) and the composition of functional-state node features. Three such composition modes were introduced: correlation, embedding, and concatenation. In the correlation (Corr in Table 2-2 in Figure 2B) mode, the flattened functional connectivity matrixes were set as node features of the functional graph. In the embedding (Emb in Table 2-2) mode, the local ROI GNN embeddings were set as node features of the functional graph. In the concatenation (Concat in Table 2-2) mode, the concatenation of the flattened functional connectivity matrixes and local ROI GNN embeddings was set as node features. For each functional feature configuration, we examined the performance of the model with and without the inclusion of structural modality, resulting in six experiments: Corr+Demo, Corr+Demo+T1, Emb+Demo, Emb+Demo+T1, Concat+Demo, and Concat+Demo+T1. The results of the experiments are shown in Table 2-2. It can be seen that the concatenation feature fusion method and the inclusion of T1 modality features significantly improved the classification performance (t test p = 0.55, <0.001, <0.01 in the three groups).

Subsequently, we jointly trained local and global networks in an end-to-end manner, speculating that this approach may enhance the embeddings generated by the local network to better fit the downstream global network. The obtained AUROCs are depicted in Figure 2C. The proposed model achieved an AUROC of 78.85% ± 5.50% when trained in two-stage mode and an AUROC of 80.64% ± 5.74% when trained in end-to-end mode (for more detailed results, refer to Table S1). The M-Attention block of the subject GNN is used to apply attention mechanisms between three MS embeddings and one MC embedding to allocate different levels of attention to different modalities. The attention scores of different modalities learned during the fusion process can reflect their importance in the diagnosis of depression. The attention scores of the three MS embeddings were functional (0.3148), demographic (0.1598), and structural (0.1367), in descending order. Notably, the MC embedding that contains information about all three data modalities achieves the highest attention score (0.3887). We also conducted ablation experiments to explore the role of different data modalities, and the experimental results are shown in Table S14.

Performance on the larger and more complex REST-meta-MDD dataset

The REST-meta-MDD dataset is more intricate than the SRPBS dataset, which contains 814 MDDs at different disease stages as

Figure 1. Objective quantitative depression diagnosis system
(A) Workflow of the diagnosis system.
(B) Framework of LGMF-GNN. (i) Feature extraction and graph initialization. Features were extracted from the data of the three modalities and further used to initialize the functional graph, structural graph, and demographic graph. (ii) The hierarchical LGMF-GNN structure. Local ROI GNN generates the embedding for each subject based on the ROI graph and ROI BOLD signals of each individual. Global subject GNN aggregates multiple information from the subject graphs of different modalities to obtain the final prediction result. (iii) Detailed network structure of local ROI GNN. (iv) Detailed network structure of global subject GNN.

A

Table 2-1. Performance of ROI-GNN and Subject-GNN separately

		ACC	AUC	Precision	Recall	F1-score
ROI-GNN	AAL	73.82±4.39	70.99±5.02	72.98±6.94	75.96±6.74	74.06±4.09
	CC200	74.51±5.35	71.08±7.38	72.57±7.93	78.17±6.18	75.05±6.14
Subject-GNN	w/o T1	74.31±4.51	74.10±5.67	75.26±6.24	74.60±7.94	73.89±4.58
	w/ T1	75.10±4.62	75.96±5.41	76.39±7.21	72.77±8.67	74.31±3.94

B

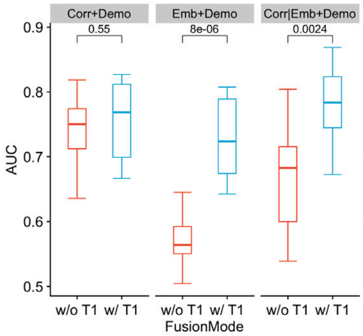

Table 2-2. Performance of two-stage LGMF-GNN under different feature fusion and modal fusion methods

	ACC	AUC	Precision	Recall	F1-Score
**Corr+Demo**	74.31±4.51	74.10±5.67	75.26±6.24	74.60±7.94	73.89±4.58
**Corr+Demo+T1**	75.10±4.62	75.96±5.41	76.39±7.21	72.77±8.67	74.31±3.94
**Emb+Demo**	61.96±5.02	57.43±7.43	62.39±9.17	65.65±10.98	62.58±6.52
**Emb+Demo+T1**	73.78±4.86	72.83±5.87	70.47±9.48	**78.46±9.58**	73.59±6.57
**Concat+Demo**	68.04±5.75	65.66±7.69	69.45±8.19	65.06±8.19	66.52±7.01
**Concat+Demo+T1**	**78.22±2.38**	**78.85±5.50**	**79.37±9.19**	74.05±8.28	**75.91±4.27**

C

D

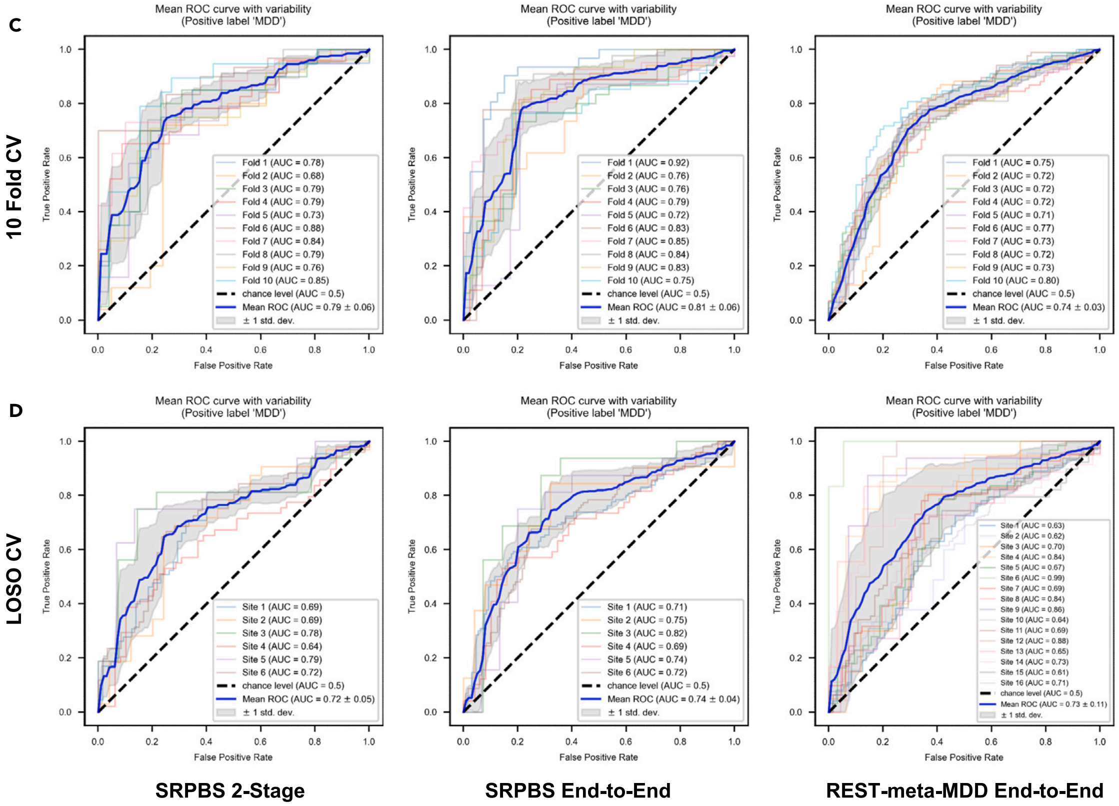

Figure 2. Performance on the SRPBS and REST-meta-MDD dataset

(A) Performance of ROI GNN and Subject GNN separately.

(B) Boxplot of AUROC of two-stage trained LGMF-GNN under different feature fusion and multimodal fusion methods. Three different groups represent different compositions of the functional state feature, and different colors represent different multimodal fusion methods. The t test shows that the addition of T1 modality significantly improves the AUROC of the model.

(C) 10–Fold CV ROCs of two-stage and end-to-end trained LGMF-GNN.

(D) LOSO CV ROCs of two-stage and end-to-end trained LGMF-GNN.

well as 756 HCs (Table 1). The dataset also contains patients with different MDD subtypes, including first-episode MDD and recurrent MDD, and some were scanned while the patient was on antidepressants. Such a dataset would more closely mirror real-world clinical scenarios while presenting increased analytical challenges. Additionally, data collection across 16 distinct sites

introduces pronounced "site effects."[46] These effects arise from differences in the MRI equipment or scanning procedures used at different imaging sites, leading to variations in imaging results or data. Site effects can obscure features of interest in neuroimaging and decrease statistical power, undermining the credibility and generalizability of the system. Following prior experiments, we conducted a 10-fold CV on the REST-meta-MDD dataset in the end-to-end training mode using all three modalities. The performance of the proposed system is shown in Figure 2C and Table S2.

To further investigate the impact of site effects on model training and generalization performance, we conducted 10-fold CV on the single-site data of the 20th cohort (S20) in the REST-meta-MDD dataset and the 6-site data of the SRPBS dataset using the two-stage trained LGMF-GNN, without specifically addressing site effects. The results of these evaluations are presented in Table S3. The analysis revealed a marked decline in system performance in multi-site scenarios compared to single-site scenarios, with ACC dropping by 22.90% and AUROC by 27.08%, This decline underscores the substantial impact of site effects on the proposed model, detrimentally affecting its performance. We conducted a thorough evaluation and discussion on the site effects in the "The evaluation of site-effect" section of supplemental experimental procedures, where we proposed solutions from the perspectives of data processing, system design, and training strategies. Although our proposed system cannot achieve similar performance in multi-site scenarios as in single-site scenarios, the five strategies (detailed in "methods for suppressing site effects and data enhancement and ablation study" in the supplemental experimental procedures) presented proved effective in suppressing site effects and mitigating performance loss. In the six-site scenario, these strategies facilitated a 9.73% reduction in performance loss, elevating the accuracy to 78.75% from 69.02%, as documented in Table S16.

Performance across MDD subtypes and under medication influence

Beyond the fundamental classification between MDDs and HCs, we further tested the capabilities of the proposed system by distinguishing between HCs and more specific MDD subtypes. Specifically, we divided the MDD patients in the REST-meta-MDD dataset into a "first-episode drug-naive" (FEDN) subset and a "recurrent" (RECU) subset. We then assessed the ability of the system to distinguish between HCs and these two MDD subtypes. The results indicated that the system accurately differentiated FEDN patients from HCs with a classification accuracy of 75.13% (AUROC: 76.73%), and RECU patients were distinguished from HCs with an accuracy of 74.05% (AUROC: 72.28%). When differentiating between FEDN and RECU patients, the classification accuracy was 76.92% (AUROC: 68.89%). For more detailed results, refer to Tables S4–S7. These results suggest that the model exhibits a slightly lower diagnostic accuracy for the RECU MDD subgroup compared to the FEDN MDD subgroup when distinguishing from HCs, but it still maintains its robustness.

We also investigated the impact of medication on the model's diagnostic performance. The analysis was feasible only for the REST-meta-MDD dataset as it provides detailed medication information. We compared the model's AUROC for drug-naive (n = 300) and treated (n = 221) MDD patients against HCs. The AUROC for the drug-naive group was marginally larger than that for the treated group, indicating a slightly better diagnostic performance for the former. However, the difference was minimal, with a difference of only 0.02, which underscores the robustness of the proposed model against medication status. Visual representations in Figures 4A–4C provide further insight into the output of the model for both groups. The density plots indicate that medication status impacts the distribution of predicted disease probabilities, with the treated group showing higher density in the lower probability range, suggesting symptom mitigation, whereas the drug-naive group exhibited a higher density in the higher probability range, reflecting more pronounced symptoms.

In summary, the proposed model demonstrates robust diagnostic capabilities across different MDD subtypes, with only slight variations in accuracy observed. Medication status appears to have a nuanced effect on the output of the model, which aligns with clinical expectations, further validating the ability of the model to capture intrinsic disease patterns.

Generalizability on the external independent Anding and OpenNeuro datasets

We evaluated the generalization performance of the proposed system on the Anding and OpenNeuro datasets. The Anding dataset is an external independent dataset containing 196 MDDs and 177 HCs from Anding Hospital, China (Table 1). The participants in this dataset belong to the same population as the training set, namely the Asian population. All the participants had refrained from any medication treatment for 2 weeks before data collection. The dataset includes both FEDN and RECU MDD cases, which could effectively test the generalization ability of the system. The OpenNeuro dataset is an external independent dataset that includes 21 patients with depressive disorder and 21 HC participants (Table 1). Diverging from the previous three datasets derived from the Asian population, this dataset contains individuals from the European population, who exhibit relatively less severe depressive symptoms. The utilization of the OpenNeuro dataset as an external independent test set allows for a rigorous assessment of the system's generalization performance under more challenging conditions.

For the external independent test procedure, the system was trained on the consolidated SRPBS and REST-meta-MDD datasets and tested on the Anding and OpenNeuro datasets, respectively. Finally, the system achieved an ACC of 69.97%, an AUROC of 72.91%, and an F1-score of 71.57% on the Anding dataset. On the OpenNeuro dataset, the system attained an ACC of 69.05%, an AUROC of 70.30%, and an F1-score of 71.11%. The external independent test results on the Anding dataset show that while the ACC and AUROC of the comparison models dropped to less than 60%, LGMF-GNN still achieved an ACC of 69.97% and an AUROC of 72.91% (for more detailed results, refer to Tables S8 and S9). These results demonstrate a good generalization performance of the proposed system.

Comparison experiments and cross-site generalization capabilities

To evaluate the generalization ability of LGMF-GNN, we conducted LOSO CV on the SRPBS and REST-meta-MDD datasets,

as exhibited in Figure 2D. The ROCs indicate that although the performance decreased when the model was trained and tested on cross-site data, the system still achieved an overall good performance, with an LOSO CV AUROC of 73.33% ± 5.70% and 75.57% ± 9.45% on the SRPBS dataset and the REST-meta-MDD dataset, respectively. To further validate the proposed system, LGMF-GNN is compared to SOTA GNN models for brain disease diagnosis. Specifically, we assessed the performance of LGMF-GNN against traditional GCN,[47] graph isomorphism network (GIN),[48] and graph attention network (GAT)[49] and popular brain GNNs, namely BrainGNN,[38] edge-variational graph convolutional network (EV-GCN),[50] local-to-global graph neural network (LG-GNN),[51] contrastive graph pooling (ContrastPool),[52] phenotypic edge relational graph attention network (pRGAT),[53] multi-scale adaptive multi-channel fusion deep graph convolutional network (MAMF-GCN),[39] specificity-aware federated graph learning (SFGL),[54] causality-inspired graph neural network (CI-GNN),[55] and interpretable graph neural networks for connectome-based brain disorder analysis (IBGNN).[56] Among these, BrainGNN, SFGL, CI-GNN, and IBGNN are local-view methods based on graph-level classification of ROI graphs; EV-GNN, pRGAT, and MAMF-GCN are global-view methods based on node-level classification on population graphs; and LG-GNN and ContrastPool are local-to-global methods. Comparing our proposed method with these approaches will demonstrate its advantages. A comprehensive introduction to each method used for comparison, including the specifics of their implementation and the chosen hyperparameter configurations, is detailed in the comparison experiments section of the supplemental experimental procedures and Tables S10.1–S10.12.

The results of 10-fold CV are shown in Figures 3A–3D, in which our system, LGMF-GNN, exhibited optimal performance for MDD diagnosis. The system achieved an increase in ACC of at least 3.65% and an increase in AUROC of 5.39% in the SRPBS dataset. Moreover, it generated at least a 4.20% increase in ACC and a 5.18% rise in AUROC on the REST-meta-MDD dataset, surpassing the results of previously mentioned models. In contrast, other methods were significantly affected by site effects and failed to produce accurate classifications, with ACC hovering at approximately 70% (for more detailed results, refer to Tables S11 and S12). Except for MAMF-GCN, all other tested methods exhibited an AUROC of less than 75%.

The t-distributed stochastic neighbor embedding (t-SNE) plots in Figure 3E visually depict the embedding outputs at each layer of MS-GCN. It is evident that as the number of layers increases, the embeddings of the same class become more clustered, while the distinction between two clusters of different classes becomes more pronounced. This observation suggests that Snowball GCN effectively mitigates the issue of oversmoothing, which often arises when the number of layers in GNN increases. We believe this is one of the key factors contributing to the superior performance of the proposed model compared to other GNN networks.

System calibration and its association with self-test depression scale score

In addition to generalization ability, we also assessed the calibration of the proposed system. The proposed LGMF-GNN exhibited good calibration because of its close agreement with the observed probabilities of MDD, as evaluated in the calibration curve (Figure 4D). A close examination of both the calibration curve and prediction results histogram revealed a marked distinction between the model trained under the two-stage mode and the end-to-end mode. The latter tends to provide a more decisive diagnostic outcome, as most predicted probabilities fall within the 0–0.1 and 0.9–1.0 range of bins. Furthermore, the two ends of the calibration curve of the end-to-end trained model are closer to the optimal calibration line, indicating that the disease risk is not significantly underestimated or overestimated. To further explore the relationship between the prediction results of the system and the severity of depression, we constructed boxplots between model prediction probability of illness, and participants' self-assessed BDI score (Figure 4E). According to the BDI-II criteria, a self-rating score of 0–13 is diagnosed as minimal, 14–19 as mild, 20–28 as moderate, and 29–63 as severe depression. Notably, the median self-test scores of patients in different predicted probability buckets fall in different partitions. This observation suggests that the system not only accomplishes the binary classification task between HCs and MDDs but it also possesses the capability to estimate the severity of depression.

System interpretation

This paper proposes a system that can assist physicians not only in diagnosing MDD but also offer references and guidance for exploring the underlying mechanisms and biomarkers of MDD. Diverging from the majority of methods that build a fixed graph structure based solely on the correlation between BOLD signals to focus only on either the individual or the population perspectives,[38,44] our proposed system incorporates learnability for the brain functional connectivity matrix, which can be optimized through local and global tasks. This feature provides a new entry point for interpreting the learning outcomes of the network—that is to say, we believe that the functional connectivity matrix learned by the network, through both the HC group and the MDD patients, can reflect the differences in brain function and structure between these two groups.

To measure this difference, for the functional modality, the average functional connectivity matrix of HCs obtained from network learning was subtracted from the average functional connectivity matrix of MDD patients to obtain the heatmap shown in Figure 5A, which we refer to as the differential functional connectivity matrix. The positive values in the matrix represent the enhanced functional connections of MDD patients compared with HCs, while the negative values imply diminished functional connections. The absolute value indicates the magnitude of the difference. We identified the top five enhanced and diminished functional connections based on their magnitudes. Figures 5B and 5C present the differential functional connections filtered using the system, along with their corresponding ROIs. The top five enhanced functional connections include left Rolandic operculum-right hippocampus, right precentral gyrus-left hippocampus, right precentral gyrus-right hippocampus, right Rolandic operculum-right hippocampus, and left Rolandic operculum-right Rolandic operculum. The top five diminished functional connections are left gyrus rectus-right cerebellum lobule VIIb, right cerebellum

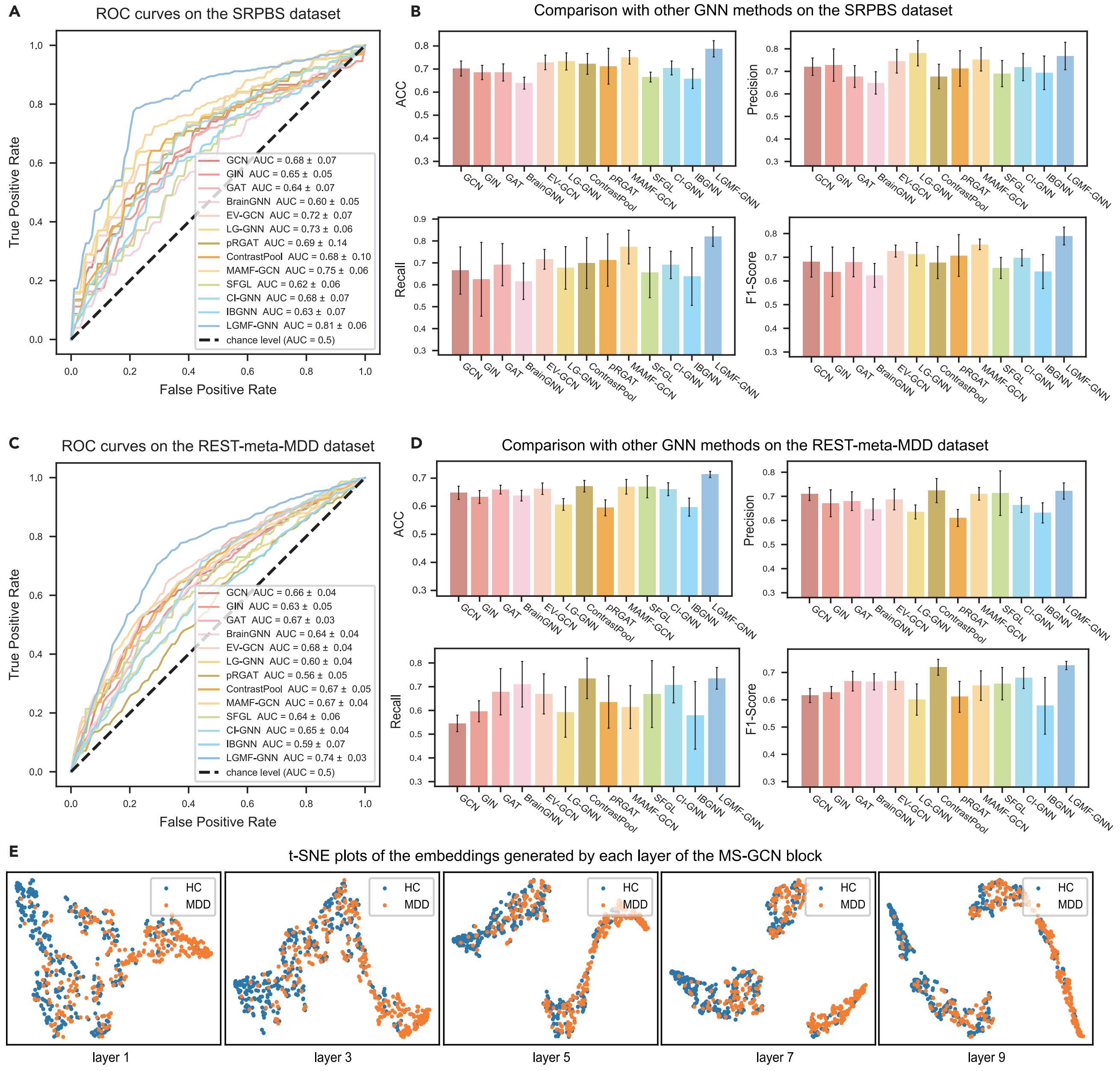

Figure 3. Comparison with other GNN methods

(A) ROCs comparison of different models on the SRPBS dataset.

(B) Histogram comparing the results of the proposed model and the SOTA models across various evaluation metrics in the SRPBS dataset. The histogram displays the mean performance metrics for each model, with error bars denoting the standard error of the mean.

(C) ROCs comparison of different models on the REST-meta-MDD dataset.

(D) Histogram comparing the results of the proposed model and the SOTA models across various evaluation metrics on the REST-meta-MDD dataset. The histogram displays the mean performance metrics for each model, with error bars denoting the standard error of the mean.

(E) t-SNE plots of the embeddings generated by each layer of the MS-GCN block.

lobule VIIb-right cerebellum lobule VIII, right cerebellum lobule VIIb-left cerebellum lobule X, right cerebellum lobule VIIb-right cerebellum lobule X, and right cerebellum crus II-right cerebellum lobule VIIb.

To gain insight into the brain regions that contribute the most to MDD diagnosis, we investigated the node degrees acquired from the absolute differential functional connectivity matrix. Based on statistical analysis from this matrix, we selected the top 10 brain regions with the largest degree to include as many potential markers as possible (Figure 5C). The selected brain regions are the right cerebellum lobule VIIb, right hippocampus, left Rolandic operculum, left gyrus rectus, left

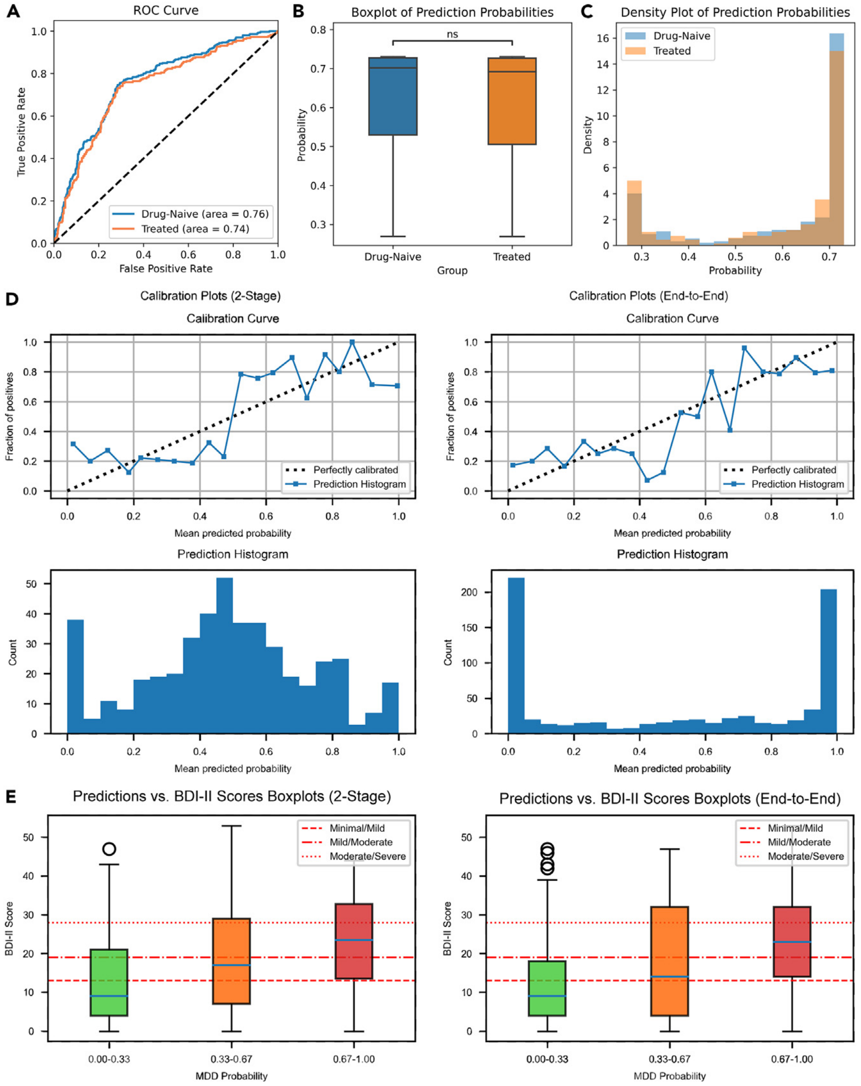

Figure 4. System calibration and association with self-test score of depression scale

(A) The ROC of drug-naive MDD vs. HC and treated MDD vs. HC on the REST-meta-MDD dataset.

(B) Boxplot of prediction probabilities output by LGMF-GNN for drug-naive and treated patients. The "ns" notation in the plot signifies that the p-value derived from the t-test is greater than or equal to 0.05, indicating no statistically significant difference of model prediction probabilities between the two patient groups.

(C) Density plots of prediction probabilities output by LGMF-GNN for drug-naive and treated patients.

(D) Calibration curve of two-stage and end-to-end trained LGMF-GNN.

(E) Boxplot of system prediction probability and participants' Beck Depression Inventory (BDI) self-rating scale scores.

cerebellum lobule X, right precentral gyrus, right gyrus rectus, left cerebellum lobule X, right cerebellum lobule VIII, right Rolandic operculum, and right cerebellum crus II, which include five cerebellar and five cerebral regions. Notably, these regions correspond to those involved in the differential functional connectivity analysis, which adds weight to our findings. We provide a detailed discussion of the functional and structural alterations that have been identified in these brain regions in MDD patients in Table S17.

For the sMRI analysis, we extracted 1,209 dimensional radiomic feature vectors from the GMV and white matter volume images, which encompass 93 radiomic characters. To determine the factors that contribute most to the diagnosis of MDD, we experimented by assessing the impact of specific radiomic characteristics on model performance. Specifically, we masked the 93 radiomic characters one by one during the test stage and identified the top five significant characters (e.g., GLCM_MaximumProbability, FirstOrder_Uniformity) that led to the greatest decrease in model performance. We asserted that these selected characters have the most significant impact on model performance and thus are highly effective in depression diagnosis. Detailed descriptions of these five characters and their implications for depression diagnosis are provided in Table S18.

The results obtained through the interpretation of our system demonstrate consistency with previous research findings, while also presenting statistically significant and reproducible discoveries that contribute additional knowledge to the field. Prior studies have indicated that depression pathogenesis entails dysfunctional neural circuits that regulate emotions, self-reflection, reward processing, and cognitive control. Abnormal functional connectivity in regions such as the precentral gyrus[20] and the hippocampus[57–59] have been found. However, structural magnetic resonance studies have revealed that during the acute phase of first-episode depression, a reduction in hippocampal, insular, prefrontal cortical, and orbitofrontal volume is common.[60] Notably, half of the abnormal brain regions in MDD patients specified by the model interpretations in this paper are located in the cerebellum. Historically underexplored beyond motor functions, the cerebellum is now recognized for its significant role in emotional and cognitive management. Liu et al.[61] found a correlation between abnormal cerebellar functional connectivity and depression in adults. Their research revealed that MDD patients exhibited markedly reduced cerebellar functional connectivity relative to both the default mode network (mainly consisting of the ventral medial prefrontal cortex and posterior cingulate/supraoccipital gyrus) and the executive control network (primarily involving the superior frontal cortex and orbitofrontal cortex). Xu et al.[62] found that emotional memory and the severity of depressive symptoms are associated with structural changes in both the posterior and anterior gray matter regions in the cerebellum in MDD sufferers.

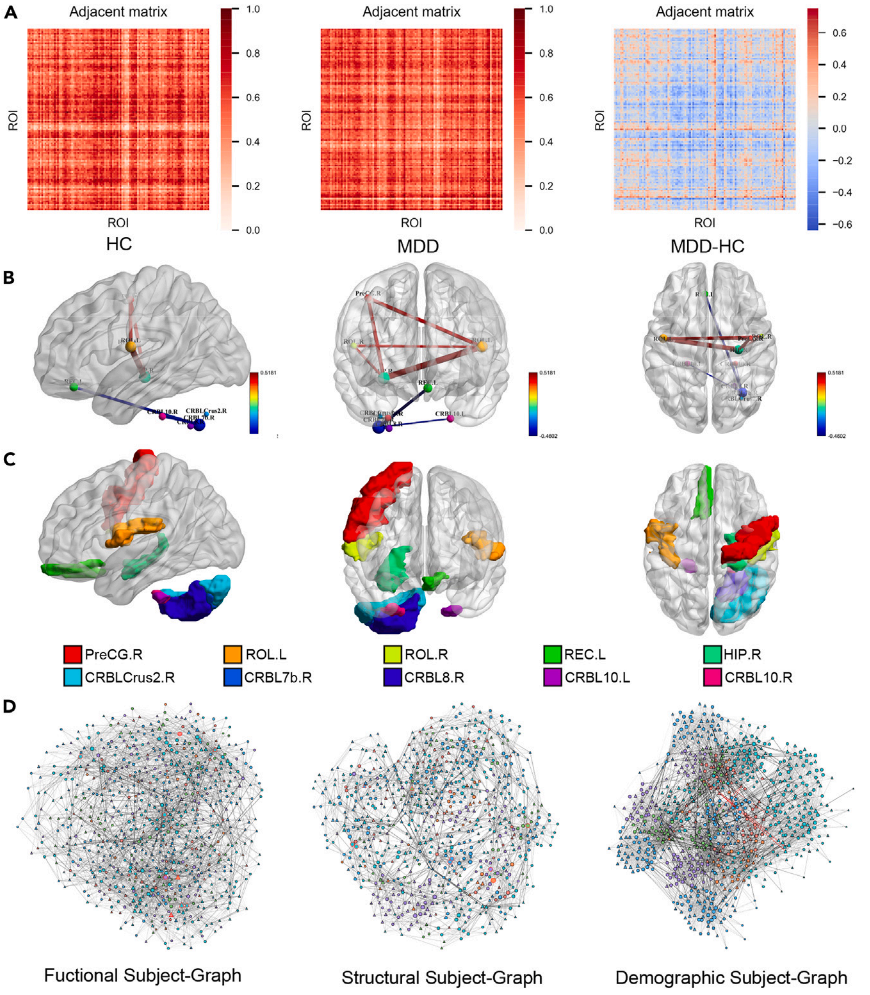

Figure 5. Abnormal functional connectivity in MDD patients obtained from the learnable functional connectivity matrix

(A) The average functional connectivity matrix of the MDD and HC groups learned by LGMF-GNN and the differential functional connectivity matrix (MDD-HC).

(B) The top five enhanced functional connections and the top five weakened functional connections shown in BrainNet Viewer.

(C) The top 10 abnormal brain regions shown in BrainNet Viewer.

(D) Subject graphs of functional, anatomical, and demographic modalities. "Δ" represents MDD, "○" represents HC, the different colors of nodes represent different sites, and the thickness of edges represents the strength of correlation between individuals.

DISCUSSION

In this study, we approached the depression diagnosis task from an individual-to-population perspective and facilitated an objective and quantitative diagnosis by effectively integrating multiple modalities, including functional, structural, and demographic information through a local-global multimodal fusion network. The interaction between individual brain regions and population graphs offers abundant referential information for disease diagnosis, improving the system classification performance and contributing to learning task-driven brain functional connectivity matrix. This, in turn, improves the interpretability of the system and provides innovative ways of analyzing disease mechanisms. Moreover, the GNN provides a powerful tool for high-quality fusion of multimodal and heterogeneous data, which offers multi-dimensional information for disease diagnosis. Data from various modalities offer different advantages, each contributing to a more holistic understanding of the disease.

We conducted our experiments on large multi-center datasets involving 2,442 participants from 24 sites. The results of 10-fold CV demonstrated that the system could effectively distinguish healthy individuals from MDD patients, achieving an ACC of 78.85% ± 5.50% and an AUROC of 80.64% ± 5.74% on the SRPBS dataset. Meanwhile, on the REST-meta-MDD dataset, it recorded an ACC of 71.34% ± 1.50% and an AUROC of 73.67% ± 2.67%. In the MDD subtype classification task, our system achieved similar performance in the HC vs. FEDN and HC vs. RECU classification tasks, with an ACC of 75.13% ± 3.16% and 74.05% ± 4.34%, respectively. This indicates the robustness of the proposed system. Furthermore, the system maintained a stable performance even in the challenging task of classifying FEDN and RECU subtypes, with an ACC of 76.92% ± 8.94%, demonstrating both expertise in diagnosing depression as a whole and a significant capability to classify subtypes within, which caters to actual clinical needs. The correlation between the predictive outcomes of the system and the patients' BDI self-assessment scores has shown that the diagnostic outputs of the system can accurately indicate the severity of depressive symptoms. The results of the LOSO CV demonstrated that the system effectively overcame the problems caused by site effects and exhibited strong generalization ability. The comparative experiments of LGMF-GNN with SOTA methods on various datasets also confirm this point.

The application of GNNs in diagnosing psychiatric disorders such as MDD and in pinpointing affected brain regions has undergone a surge in recent times. However, several challenges persist, including oversmoothing issues, a singular perspective, and limited interpretability.[63–67] Our approach aligns with and expands upon the current insights into MDD diagnostic models, emphasizing the advantages of both local and global perspective-based GNN depression diagnostic models. By developing a local-global network architecture, enhanced by sophisticated network modules and optimized training strategies, the proposed method not only synthesizes the benefits of existing methodologies but it also builds upon them. It offers a comprehensive global perspective through the population graph, while simultaneously preserving the detailed information of individual brain regions for the diagnosis

of MDD. Comparative experimental results have demonstrated that the LGMF-GNN proposed in this paper achieves superior performance compared to current advanced local, global, and local-global GNN methods. Moreover, in line with the contemporary shift toward multimodal integration in artificial intelligence (AI)-based depression diagnostic approaches,[68–70] the multimodal fusion strategy introduced in this study adeptly addresses the complexity of MDD by integrating various data sources. This approach significantly reduces the constraints inherent in single-modal data analysis found in current diagnostic models. Consequently, it offers a more nuanced and comprehensive insight into the diverse manifestations of the disease, enhancing our understanding of its heterogeneity and complexity.

Finally, through interpretation studies, we proposed a set of biomarkers, including abnormal brain functional connections and anatomical structures that can provide valuable insights for the MDD diagnosis from both functional and anatomical perspectives. These works make up for the deficiency of interpretability of MDD diagnostic models, providing alternative methods and perspectives for the interpretation of these models.

In conclusion, interpretable, end-to-end learning with a hierarchical graph structure can promote objective and quantitative diagnosis of MDD and has the potential to identify its underlying mechanism.

System generalization ability

Generalizing across multiple sites poses a significant challenge for a neuroimaging-based classifier due to different MRI scanners, acquisition parameters, and participant instructions. This knotty problem of site effects was also present in our study: three public multi-site datasets and one private dataset were used, involving 24 sites, 1,182 patients with MDD, and 1,260 HCs. The performance of the system in single-site and multi-site scenarios shown in Table S3 indicates that the site effects have a significant negative impact on model training and testing. Therefore, to mitigate this interference and improve the generalization performance of our system, we utilized the ComBat method to harmonize the data across sites (detailed in the "methods for suppressing site effects and data enhancement and ablation study" in the supplemental experimental procedures) during the data preprocessing stage. Additionally, our system design incorporates site information into demographic characteristics by learning and suppressing site differences through a demographic graph. Finally, the adversarial training method and domain migration loss were designed to encourage LGMF-GNN to disregard the interference factors introduced by site effects and focus on the features that are the most relevant to classification. The ablation results of the loss functions are shown in Table S15. Although the LOSO CV performance of the model is lower than that of a 10-fold CV when dealing with data from unfamiliar sites, it still demonstrates good classification performance and generalizability. The experimental results indicate that the methods employed in suppressing site effects are effective and outline a meaningful direction (Table S16). These approaches may serve as a reference for future multi-site research.

Multimodal fusion

Taking advantage of the high modeling flexibility of GNN, the proposed system successfully achieved a high-quality fusion of the three data modalities: functional, structural, and demographic. As shown in Figure 2B, the addition of the T1 modality provided essential anatomical insights, considerably enhancing the diagnostic accuracy for MDD. Concerning the features of functional modality, we experimented with three fusion methods, and our findings suggest that concatenating correlation features and local network embeddings resulted in the most optimal performance. This is because the concatenation form not only retains the original shallow features but it also incorporates more abstract high-order features extracted by the local ROI GNN network.

We visualized the subject graph constructed based on the three modalities in Figure 5D. In this visualization, "Δ" represents MDD, "○" represents HC, while the various node colors correspond to distinct imaging sites. Additionally, the thickness of the edges represents the strength of inter-individual correlations. Both the HC group and the MDD patient group have nodes with significantly larger degrees (marked in red border) in the functional and structural graphs, representing typical individuals of each group. The sMRI images of the patients corresponding to the eight MDD nodes with the highest degree in the structural subject graph are presented in Figure S7, providing an anatomical characterization of the "typical MDD patients." In Figure 5D, it can be seen from the graph that edges with larger weights often exist between individuals within the same category, helping to summarize the general characteristics related to a single category. In the demographic graph, nodes within the same site are more densely connected, promoting effective communication of information. In contrast, nodes from different sites are sparsely connected by edges among only a few individuals in each site. These edges (marked in red) build a pathway that facilitates site harmonization.

In conclusion, the graphs of the three modalities perform distinct functions within the network, each providing unique and vital information. The M-Attention block of the subject GNN learns the attention score of each modality during the fusion process. Based on the attention scores, the importance of the three modalities for depression diagnosis in descending order was functional (0.3148), demographic (0.1598), and structural (0.1367). It is worth noting that consistent with our system design, the MC embedding that includes information from all three modalities obtains the highest attention score (0.3887), underscoring its pivotal role.

Hierarchical graph learning

Based on the hierarchical network, the interaction between local and global perspectives enhances the diagnosis performance of LGMF-GNN. In Figures 2C and 2D, we compared the ROC curve of the 10-fold CV of the system obtained through two-stage training and end-to-end training. The system obtained through end-to-end training achieved a higher AUROC, supporting the effect of the hierarchical structure. Furthermore, upon comparison of the calibration curve and the histogram, we recognized that the end-to-end trained model tends to give a firmer diagnosis, and the predicted probability is better aligned with the prevalence rate. This also demonstrates the collaborative optimization of local and global networks.

The advantages of hierarchical network structure can be summarized into three primary aspects. First, the fine-grained brain

region information provided by the local network and the global population information provided by the global network can be collaboratively optimized in the network training stage, providing more comprehensive information for disease diagnosis. Second, the addition of the global network allows for more flexibility in the fusion of multimodal information, which is achieved by combining the three subject graphs of different modalities. Third, the local network enables the learnability of the functional connectivity matrix, thereby improving the interpretability of the system. Meanwhile, the local network also provides better feature vectors for nodes in the global graph. These enhancements hold promise for broader applications beyond MDD diagnosis, potentially extending to the study of other brain diseases or neurological conditions, such as ASD and AD.

Findings about the underlying mechanisms of depression obtained through interpretation

Through the interpretation of the system, from the perspective of functionality, we discovered abnormal brain functional connections and functional alterations of brain regions in MDD patients. From the perspective of structure, we identified anatomical structural alterations in brain gray matter and white matter as denoted by imaging radiomics characters. In Table S17, we present a detailed literature review that showcases the findings of existing studies on the brain regions identified through interpretable analyses in this paper.

Among the discovered abnormalities, functional abnormalities in the hippocampus, precentral gyrus, and the Rolandic operculum are confirmed by more existing research results. In depression analysis, the hippocampus has always been a key focus. Several studies have shown significant functional and structural abnormalities in patients with depression compared to HCs. Functionally, patients with MDD had increased functional connectivity between the right anterior hippocampus and lingual gyrus, while functional connectivity between the right posterior hippocampus and the right inferior frontal gyrus was diminished.[71] Moreover, reduced functional connectivity was observed between the right hippocampus and the bilateral medial superior frontal gyrus,[72] accompanied by a decrease in the node strength of the right hippocampus cornu ammonis 3/4, suggesting weakened brain-wide connectivity.[73] There are also studies showing that the MDD group exhibited significantly weaker connectivity of the right hippocampal subregional networks with the temporal cortex (extending to the insula) and basal ganglia but showed increased connectivity of the right subiculum to the bilateral lingual gyrus.[74] Additionally, with the aging of patients with depression, the functional connectivity between the right anterior hippocampus and the left postcentral gyrus tends to increase.[75] Structurally, hippocampal volume reduction is evident even in the early stages of MDD,[76–78] affecting both first-episode patients[79] and adolescents.[80] This reduction is also observed in patients with depression and bipolar disorder, affecting both GMV and white matter volume in the right hippocampus.[81]

The changes in the precentral gyrus in depression have also received attention. Compared to HCs, patients with depression have a higher regional cerebral blood flow and greater activation in the right precentral gyrus.[82,83] Non-anxious depression patients show reduced functional connectivity between the right precentral gyrus and the right centromedial/laterobasal nucleus.[84] In the somatic depression group, the regional homogeneity (ReHo) and amplitude of low-frequency fluctuations (ALFF) in the bilateral precentral gyrus are significantly lower than the pure depression group.[85]

The performance of the Rolandic operculum in depression is also noteworthy. In FEDN patients with depression, the functional connectivity of the left Rolandic operculum is enhanced.[86] In patients with late-onset RECU depression, the ReHO of the left Rolandic operculum gyrus is higher.[87] Alterations of ALFF in the right Rolandic operculum have also been identified.[88,89] In addition, structural alterations in the right Rolandic operculum have been observed to correlate with depressive symptoms in patients suffering from post-stroke depression.[90]

Some of our findings, however, such as the functional alterations in the cerebellum and abnormal functional connectivity between cerebral and cerebellar regions, represent fresh contributions from this study with a large sample size. The role of the cerebellum in depression is increasingly recognized in research. For instance, in terms of function, connectivity studies have revealed diminished cerebral and cerebellar coupling in lobules VI and VIIA/B with prefrontal, posterior parietal, and limbic regions in MDD patients.[91] Compared with HCs, patients with MDD exhibit significantly increased ReHo values in the right cerebellum crus II.[92] Structurally, studies have indicated increased gray matter density (GMD) in the right cerebellum VIII of MDD patients, alongside reduced gray GMVs in the right and left cerebellum VIII and X, which correlate with depression severity as measured by BDI scores.[62] Despite these advances, the exploration of functional connectivity between cerebral and cerebellar regions in depression remains underdeveloped. Our interpretable results underscore the imperative for more in-depth investigation in this direction.

This study's findings delineate both functional and anatomical abnormalities associated with the cerebellum and cerebral regions, offering valuable contributions to the advancing field of depression research. Our interpretations fortify current research on MDD-related alterations in regions such as the prefrontal cortex and stimulate inquiry into underexplored areas, particularly the role of the cerebellum in the etiology and progression of depression.

Clinical application and advantages

The LGMF-GNN model introduced in this paper is an end-to-end approach that automates the construction of ROI graphs and subject graphs, enabling an objective and quantitative diagnosis of depression. This innovation holds significant promise for its application in clinical settings. Hospitals can utilize medical records and imaging data from previous patients and healthy individuals to establish ROI graphs and subject graphs, thereby creating a comprehensive graph case library. Upon the arrival of a new patient or research subject, the hospital only needs to collect their MRI scans and basic demographic information, such as gender, age, and education level. The LGMF-GNN can then automatically construct the ROI graph and integrate the new subject into the population graph as a new node. During the model inference phase, the local ROI GNN module performs fine-grained feature extraction and fusion from an individual perspective. Meanwhile, the global subject GNN module facilitates automated message passing and comparison between

the new individual and the case nodes in the hospital's local case library within the latent space. This results in node-level classification, providing an objective and quantitative diagnosis of depression and outputting the probability of illness for the newly included subject. Additionally, the model can identify the most similar past cases from the case library based on the edge weights of the subject graph, offering interpretability.

Clinical practitioners can continue to use their customary diagnostic procedures, but they can now complement their decision-making process with the quantitative probability predictions provided by the LGMF-GNN, as well as the most similar cases selected from the case library. This integration enhances the objectivity and quantification of clinical diagnosis. It is noteworthy that the diagnostic framework based on LGMF-GNN is scalable and shareable. The population graph constructed via LGMF-GNN serves as an anonymized, lightweight case library. In this library, patient data such as fMRI, sMRI, and demographic information are stored, updated, and maintained as nodes with highly abstracted feature vectors. Leveraging federated learning, multiple hospitals across different regions can share and integrate their population graphs, thereby establishing a larger-scale case library. This deep feature-based sharing significantly reduces the risk of patient privacy breaches. With the collaboration of more hospitals and the accumulation of data over time, the population graph will continue to grow and strengthen, offering more precise diagnoses and more reliable insights into the underlying mechanism of MDD.

The lifetime prevalence rate of depression in the population is roughly 1 in 5.[17,93–95] Currently, studies are focusing on training and validating depression diagnostic models on unbalanced datasets and trying to reduce the interference of unbalanced data distribution on model training and generalization.[25,96] To assess the performance of LGMF-GNN on unbalanced datasets, we constructed a validation set with an MDD:HC ratio of 1:4 using the bootstrap method. LGMF-GNN was then validated using this set. The experimental setup and results are presented in Table S24 of the supplemental information. Despite the performance degradation in scenarios with unbalanced class distribution, LGMF-GNN still achieved performance comparable to that of the current methods.

Improving and future work

The local-global GNN architecture, along with the multimodal fusion strategy and the multi-site harmonization techniques, has demonstrated its effectiveness in enhancing diagnostic performance. The conceptual framework we have established is inherently versatile. The strategy of leveraging GNNs for local-global information integration and multimodal data fusion is a promising approach that warrants exploration in other diagnostic domains.

We identify three main limitations of LGMF-GNN and propose potential solutions for future work. First, an important distinction of our study from previous works is the local-global hierarchical graph learning strategy. However, this hierarchical structure also brings new limitations and challenges. In the subject graph, we include all individuals in one graph. With large datasets, the process requires excessive computing and storage resources, posing greater challenges to model training and generalization ability. Employing graph sampling methods like GraphSAGE[97] and FastGCN[98] could mitigate these resource demands and enhance the scalability and adaptability of the model to new data. Second, overfitting and site effect mitigation also remain concerns in multi-site data analysis. Although we have already proposed and utilized several methods, such as ComBat, adversarial training, and domain migration loss, to suppress site effects in this paper, the generalization of our system is still affected as the number of data sites increases. To address this issue, more neuroimaging data harmonization and domain migration methods need to be developed. Third, the network failed to provide sufficient interpretability of structural modality features extracted from T1 MRI, and this study did not delve deeply into the changes in the fine and exact anatomical structure of MDD patients compared with HCs. Further refinement of T1 MRI structural feature extraction methods could yield richer anatomical insights for MDD diagnosis.

We are currently collaborating with Beijing Anding Hospital and several other medical centers to clean and process the data of 9 million clinical records from more than 1 million individuals in the Beijing-Tianjin-Hebei mental health big data platform. We are contributing to the development of the China Brain Plan 2030 data platform for mental health disorders. These efforts are expected to facilitate a more comprehensive validation and long-term progress of the proposed system. We will also proactively release our large-scale multimodal datasets, pushing AI-assisted quantitative MDD diagnosis and treatment to a deeper level.

EXPERIMENTAL PROCEDURES

Data acquisition and preprocessing

Dataset Introduction

Anding dataset. The dataset acquired from Anding Hospital, China contains the raw fMRI and sMRI scanning results and clinical records of 196 MDD patients and 177 HCs (Table 1). Data collection was conducted using standardized scanners and parameters. The MDD patients include both those with their first episode of depression and those with recurrent depression. The enrollment process for this dataset was carefully controlled. Only MDD patients who had previously been medicated but had refrained from taking medication for at least 2 weeks before data collection were included. Functional images were acquired using the gradient-echo echo-planar imaging sequence, and 200 volumes were collected, with a total scan time of 6 min 40 s. The scan parameters of the functional images were as follows: repetition time (TR) = 2,000 ms; echo time (TE) = 30 ms; number of slices = 33, interlaced axial scanning; slice thickness/gap = 3.5/0.7 mm; flip angle (FA) = 90°; matrix = 64 × 64; field of view (FOV) = 200 × 200 mm^2; and voxel size = 3.13 × 3.13 × 4.2 mm^3. T1 structural images were acquired using the magnetization-prepared rapid acquisition gradient echo sequence, and the scan parameters were as follows: TR = 2,530 ms; TE = 1.85 ms; number of sagittal slices = 192; slice thickness = 1 mm; FA = 15°; matrix = 256 × 256; FOV = 256 × 256 mm^2; and voxel size = 1 × 1 × 1 mm^3. The diagnoses were established by senior psychiatrists based on the *DSM-IV* criteria for MDD, utilizing the Mini-International Neuropsychiatric Interview (MINI) diagnostic interview as the assessment standard.

SRPBS dataset. The SRPBS dataset[99] is a dataset collected and released by the SRPBS project. Neuroimaging data and demographic information from 457 MDD patients and HC participants were collected at six sites. Each participant underwent a single rs-fMRI session, a structural MRI session, and an optional field map session. Six scanners from three manufacturers (Siemens, Philips, and GE) were used to produce these neuroimaging data. A coherent protocol was designed and used for rs-fMRI. Detailed imaging parameters used at each site for rs-fMRI and T1 structural MRI are summarized in Table S19. Diagnosis of MDD was conducted site specifically. At sites 2, 3, 4, and 5, affiliated with Hiroshima University, diagnoses were made by expert clinicians based on the *DSM-IV-Text Revision* or *DSM-5* criteria, with confirmation via the MINI at the time of study participation. At Kyoto University's site 6, MDD diagnoses were determined using the Structured Clinical Interview

for *DSM* (SCID). Site 8, which is associated with the University of Tokyo, diagnosed psychiatric disorders using *DSM-IV* criteria. HCs were screened for psychiatric disorders using the MINI.

REST-meta-MDD dataset. In the REST-meta-MDD project,[19,100] 16 research groups from 16 hospitals in China agreed to share data from MDD patients and matched HCs from studies approved by local institutional review boards. The project contributed 1,570 subjects in total—814 MDDs and 756 HCs. The participating groups first preprocessed fMRI images with a standardized protocol at local hospitals and then shared the final fMRI indices and brain matters segmented from T1 sMRI along with demographic (age, sex, and education) and clinical information (e.g., FEDN/RECU, medication usage, illness severity). Detailed imaging parameters used at each site for rs-fMRI and T1 structural MRI are summarized in Table S20. The diagnostic labels for the dataset were derived from the study cohorts as provided by the contributing hospitals.

OpenNeuro dataset. The OpenNeuro dataset[101] involved 21 patients who suffer from DDs and 21 HCs from a single center in Russia, providing raw fMRI and sMRI images as well as demographic information for each subject. The fMRI study was carried out in the International Tomography Center, Novosibirsk, using a 3-T Ingenia scanner (Philips). Functional imaging scans were acquired using the following parameters: TR = 2,500 ms; TE = 35 ms; voxel size 2 × 2 × 5 mm^3; and fat suppression mode. The reference anatomical image was obtained by the T1w three-dimensional (3D) turbo field echo method with a voxel size of 1 × 1 × 1 mm^3. The instruction for participants was to lie still with eyes closed for 6 min. The diagnostic criteria for depression are based on the *ICD-10*,[102] which encompasses mild depression (F32.0), moderate depression (F32.1), and dysthymia (F34.1). Notably, the severity of all three depressive disorders in this dataset is lower than that of MDD (F33.0). The final diagnosis was made at the multidisciplinary clinic Pretor and the International Institute of Psychology and Psychotherapy. None of the patients received antidepressants. Additionally, both groups were equivalent in terms of gender, mean age, and intelligence level (as measured by Raven's Coloured Progressive Matrices).

Finally, a total of 2,442 participants (1,260 depressive disorder patients vs. 1,182 HCs) from 24 sites (6 sites from SRPBS, 16 sites from REST-meta-MDD, 1 site from Anding, and 1 site from OpenNeuro) were included in our analysis. This study was approved by the institutional review boards at the respective institutions and was conducted in accordance with the ethical standards of the Helsinki Declaration. Informed consent was waived for this retrospective study, as no protected health information was used. Detailed demographic and clinical characteristics of the study population are shown in Table 1. The category and site distribution of the SRPBS and REST-meta-MDD datasets are detailed in Tables S21 and S22.

Data Preprocessing

Rs-fMRI preprocessing. Each rs-fMRI scan was pre-processed using Data Processing & Analysis for (Resting-State) Brain Imaging (DPABI).[103] We first discarded the first five time points and corrected all volume slices for different signal acquisition times. Then, the time series of images for each subject were realigned. After realignment, individual structural images were co-registered to the mean functional image. To remove the nuisance signals, the Friston 24-parameter model and global signal regression[104] were referenced to regress out head motion effects from the realigned data. The DARTEL tool was used to transform the functional data from the individual native space to the MNI space. Further, spatial smoothing and temporal filtering were performed to reduce noises.

T1w MRI preprocessing. For the SRPBS, Anding and OpenNeuro datasets with raw whole brain T1 sMRI provided, the skull-stripped T1w MRI can be obtained after the rs-fMRI preprocessing with the DPABI tool. The T1 image was then affine registered to the Montreal Neurological Institute (MNI) atlas by FSL's Functional Magnetic Resonance Imaging of the Brain's Linear Image Registration Tool tool,[105] which has a voxel size of 1 × 1 × 1 mm^3. To remove the black boundaries, the dimension of the 3D brain volume is center cropped into a size of 140 × 170 × 140 mm^3. Additionally, GMV and white matter volume images were obtained for the three datasets through voxel-based morphometry (VBM) using the SPM tools in the DPABI toolbox. VBM provides an automated quantitative analysis of the distribution of gray and white matter to detect differences in brain tissue concentration for each voxel (e.g., GMD). To include voxel-wise volume changes, the GMD is then modulated by multiplication with the Jacobian determinant (JD). The JD is derived from the nonlinear deformation field needed to transform each subject brain to a given template brain. The modulated GMD is then multiplied with the voxel volumes and is interpreted as GMV.[106] GMV and white matter volume in SPM can provide insight into the changes and differences in brain anatomy and can be used to identify biomarkers of brain diseases.

Data exclusion and quality control. Stringent data exclusion and quality control measures were implemented to ensure the integrity of the datasets used in this study. Criteria for exclusion included age, image quality, head motion, spatial correlation, site-specific characteristics, and duplicate entries. The specific criteria and the number of subjects excluded based on these criteria for each dataset are detailed in the "data cleaning and quality control" section of the supplemental experimental procedures and Figures S1–S4). These measures were crucial for minimizing biases and ensuring the high quality of the analyzed data.

Feature Extraction

rs-fMRI feature extraction. Previous research has confirmed the correlation between blood flow levels in the brain and neural activity in the brain.[107] Therefore, the BOLD signal series reflects the changes in the activity levels of each ROI over time. It is one of the important indicators for studying brain function. We chose the widely used revised AAL[108] and CC200[109] brain atlases for ROI definition. The AAL brain atlas is a commonly used brain atlas that divides the human brain into 116 regions, including 90 regions in the brain and 26 regions in the cerebellum, and assigns a label to each region to help researchers identify and distinguish different brain regions. The CC200 brain atlas is a more complex brain atlas that divides the human brain into 200 regions. Both brain atlases are derived by analyzing and processing a large amount of brain imaging data and using MRI technology to measure the connectivity and organization of each region and have been used to study neurological and psychiatric disorders such as AD, Parkinson disease, and ASDs.

Based on the ROI defined by the AAL and CC200 brain atlases, the average BOLD signal within each ROI was extracted using the DPABI tool. Since the rs-fMRI data in the dataset were collected based on different scanners and protocols, the length of the extracted ROI BOLD signals was different. To align the data for subsequent extraction of temporal features, the extracted ROI BOLD signals were truncated using a sliding window with a certain window width and step size to obtain a time series of equal length. The Fisher Z-transform has been applied to standardize the time series of each ROI. We measured the functional connectivity between brain regions by the Pearson correlation of ROI BOLD signals to form an initial functional connectivity matrix.

T1-sMRI feature extraction. For the SRPBS dataset, we used a global-local transformer to extract anatomical features from the preprocessed image patches since it provided the whole raw T1-sMRI images. The optimization task is designed as brain age prediction, which correlates with anatomical structure and avoids information leakage for MDD diagnosis. The feature map $m_{GLT} \in \mathbb{R}^{n \times d}$ before the fully connected layer is extracted as each subject's anatomical feature vector, where n is the number of ROIs and d is the length of the feature vector for each ROI. However, the REST-meta-MDD dataset does not provide raw T1-sMRI data, but only contains structural image data of segmented gray matter, white matter, and cerebrospinal fluid volume images. Previous neuroimaging studies have shown that the GMV and white matter volume are correlated with depression.[110] Therefore, the radiomics features were extracted from the whole four datasets using PyRadiomics.[111] We extracted a 1,209-dimensional radiomics feature vector from each subject's GMV and white matter volume images respectively, yielding a 2,418-dimensional anatomical feature vector for each subject. Every 1,209-dimensional radiomics feature consists of 93 radiomics metrics extracted from the original images and 12 derived images (e.g., Laplacian of Gaussian filtering and wavelet transform). For details on feature extractor settings, see Figure S5.

Controlling for nuisance variables. Site effects[112] are important confounding factors that cannot be ignored in MRI-based imaging studies. The datasets used in this study are collected from multiple institutions using different scanners and protocols. The SRPBS dataset was collected from six different sites using different protocols, and the REST-meta-MDD dataset involves 16 cohorts from different hospitals, leading to series site effects. The nuisance variables caused by the heterogeneous scanner manufacturers, different acquisition protocols, and instructions to participants are difficult to remove even with a unified image preprocessing pipeline. To reduce the effects of unwanted nuisance variables, we applied the ComBat harmonization,[113] a multivariate

mixed linear regression model, on rs-fMRI-derived connectivity measures (see the "methods for suppressing site effects and data enhancement and ablation study" section in the supplemental experimental procedures for specific implementation details). According to Figure S6, this method can successfully regress out site effects while avoiding over-correction on important biological variance.

Model architecture and training

An overview of the proposed method is illustrated in Figure 1. The model mainly consists of a local ROI GNN and a global subject GNN. Each subject's ROI signals and initial functional connectivity matrix were fed into the ROI GNN to generate an embedding and a refined functional connection matrix. Then, subject graphs with different modalities were constructed based on the brain embedding generated by ROI GNN, with different atlases for each subject, T1 features, and the non-imaging data (e.g., age, sex, data acquisition site, years of education), respectively. The global subject GNN is performed on this graph for node classification to obtain the disease state prediction for each subject. The specific structure of the network and the method of graph construction are described in detail in the following sections.

Local ROI GNN

Network structure. As shown in Figure 1B, the local ROI GNN mainly consists of three components: (1) a GRU regional time series encoder, (2) a graph generator based on regional time series embedding, and (3) a GNN predictor to generate the graph level embedding and the local prediction.

GRU regional time series encoder. BOLD signal has been widely used in brain disease diagnosis and research. However, traditional BOLD signal encoding methods such as Pearson correlation or partial correlation failed to capture the temporal order. As a variant of the widely used time series signal encoder LSTM (long short-term memory), bidirectional GRU (bi-GRU) has a simpler structure and is able to obtain complete time series information by calculating and combining forward and backward signals. Therefore, we chose bi-GRU as the time series encoder to achieve lightweight frameworks and fit the low temporal resolution of the BOLD signal. Specifically, for an input BOLD time series $X \in \mathbb{R}^{n \times t}$ of a subject, where n is the number of ROI and t is the length of the time series, the GRU encoder generates a regional embedding for each ROI, $h_e = Encoder(x), h_e \in \mathbb{R}^{n \times d}$, where d is the output size of the GRU encoder.

Graph generator. The graph generator is designed to generate a flexible graph structure based on the encoded time series feature h_e. Since the graph structure is expressed by the adjacent matrix A, a learnable graph structure can be generated as $A = softmax(h_e h_e^T) \cdot n$. We enhanced the edge weight with the number of ROIs (nodes) to avoid too-small variance of the edge weights and ensure the sparsity of the graph. The learnable graph structure A can be regularized by the downstream prediction task.

GNN predictor. For the graph predictor, we adopted a three-layer GCN model, and the concatenation aggregation with attention mechanism was employed to transform and propagate node features and structure information on the constructed graph. The node feature F_p of node p was initialized as a vector of Pearson correlation coefficients to all ROIs. Specifically, the k-th GCN layer is defined referring to GCN proposed by Kipf and Welling[114] as:

$$H_l^k = \sigma\left(GCN\left(H_l^{k-1}, A\right)\right) = \sigma\left(D^{-\frac{1}{2}} A D^{-\frac{1}{2}} H_l^{k-1} W^k\right) \quad \text{(Equation 1)}$$

where D is the diagonal matrix, A is the adjacent matrix derived from the graph generator, and W^t is a trainable weight matrix of the k-th layer, which is a two-layer MLP in our implementation. $H_l^0 = F$. The final embedding of the whole graph is calculated through concatenating node embedding weighted by the attention score.

$$H_{lG} = concat\left(a_{ROI} H_l^k\right) \quad \text{(Equation 2)}$$

$$a_{ROI} = Attention(m) = softmax\left(\Sigma_{j=0}^{n} m_{ij}\right) \cdot n \quad \text{(Equation 3)}$$

Finally, another MLP layer is employed for class prediction $\hat{y}$

$$\hat{y} = MLP(BatchNorm1D(H_{lG})). \quad \text{(Equation 4)}$$

Global subject GNN

Graph construction. In subject graphs, an individual is modeled as a node in the graph, and the edges between nodes reflect the connections between individuals. Three types of subject graphs are constructed based on medical imaging and non-imaging data, as shown in Figure 1B. The first type is the functional subject graph, denoted as $G_f = (V_f, E_f, W_f)$. Similarities between graph embeddings generated by local GNN of two subjects are extracted to generate this k-nearest graph, which models the correlation of functional brain activity among different individuals. The second type is the anatomical subject graph, denoted as $G_s = (V_s, E_s, W_s)$. This is a k-nearest graph based on the similarities of the anatomical structure of the brain reflected by T1 sMRI features. The third type is the demographic subject graph, denoted as $G_d = (V_d, E_d, W_d)$. In this graph, the relationship of subjects is evaluated by non-image discrete data such as age and sex.

For G_f, the calculated functional feature embedding H_G is first taken as the feature of the corresponding node. Then, the edge weight W_f based on the cosine similarity of node features is calculated, and K pairs of nodes with top K edge weights are selected to construct a k-nearest neighbors (KNN) graph. G_s is constructed in a similar way, with the feature vector extracted from T1w MRI as the node feature. For the G_d, inspired by EV-GCN,[50] a PAE is used to determine the weights between subjects v_{si} and v_{sj} based on additional information provided by non-imaging data vector η. $W_d(i,j)$ is defined as $W_d(i,j) = \frac{cos(MLP(\eta_i), MLP(\eta_j)) + 1}{2}$, where cos denotes the cosine similarity between two input vectors.

Network structure. The global subject GNN is designed to be a multimodal fusion network with three MS-GCN blocks to extract the unique feature of each data modality, an MC-GCN block to fuse common information shared by all data modalities and a multimodal attention block (M-Attention) to achieve efficient information integration.

Snowball GCN. Li et al.[115] have clarified that GCN is essentially a type of Laplacian smoothing, which computes the new features of a vertex as the weighted average of itself and its neighbors. A k-layer GCN block will aggregate information from k-hop neighbors. However, stacking multiple layers of GCN will cause the oversmoothing problem, in which case, the features of vertices from different clusters are mixed and indistinguishable. Due to these limitations, most GCNs are no deeper than four layers,[116] which makes it hard to aggregate information comprehensively on the subject graph with more than 2,000 nodes. To mitigate the impact of this problem, the present study used the snowball GCN block proposed by Zhao et al.[117] as the basic GCN block. The main idea of this block is connecting multi-scale feature information in the hidden layer with a densely connected graph network to obtain richer representations of each node. The structure of snowball GCN is as follows:

$$H_g^0 = X, \quad \text{(Equation 5)}$$

$$H_{l+1} = Tanh\left(L\left[H_g^0, H_g^1, \ldots, H_g^l\right] W_l\right), l = 0, 1, 2, \ldots, n-1, \quad \text{(Equation 6)}$$

$$C = Tanh\left(\left[H_g^0, H_g^1, \ldots, H_g^n\right] W_n\right), \quad \text{(Equation 7)}$$

$$H_{gG} = normalize(L^p C W_c) \quad \text{(Equation 8)}$$

where n is the number of snowball GCN layers, W_l, W_n, W_c are trainable matrixes, $H_g^0, H_g^1, \ldots, H_g^l$ are extracted features, H_{gG} is the global subject-graph embedding of one specific modality, and $p \in \{0, 1\}$. When $p = 0, L^p = I$; when $p = 1, L^p = L = D^{-\frac{1}{2}} A D^{-\frac{1}{2}}$, which means that we project C back onto the Fourier basis. This is necessary when graph structure encodes a great deal of information.

MS-GCN block. The MS-GCN is applied to extract MS embeddings, which is defined as follows:

$$H_s^f = SnowballGCN\left(X^f, A^f\right) = SnowballGCN\left(H_{lG}, A^f\right), \quad \text{(Equation 9)}$$

$$H_s^s = SnowballGCN(X^s, A^s), \quad \text{(Equation 10)}$$

$$H_s^d = SnowballGCN\left(X^d, A^p\right), \quad \text{(Equation 11)}$$

where X^f, X^s, X^d are node features for rs-fMRI, sMRI, and demographic modalities, respectively, and H_s^f, H_s^s, H_s^d are the MS representations. The weights of the three snowball GCN networks are independent of one another, making it possible to extract unique features more effectively.

MC-GCN block. Although each modality and brain atlas has a specific data structure and semantic information, it is impossible to completely decouple the different types of data. For the same task, data from different modalities tend to contain some common information. Extracting this shared information not only helps to summarize high-quality features for solving the final problem but it also avoids redundancy in multimodal information fusion. To achieve this target, we add MC-GCN to the model, which shares the weight matrix between different modes when performing snowball GCN.

$$\begin{aligned} H_c^f &= SnowballGCN(X^f, A^f) \\ &= SnowballGCN(H_{IG}, A^f) \\ &= normalize\left(L^{fp} C^f W_{cs}\right), \end{aligned} \quad \text{(Equation 12)}$$

$$H_c^s = SnowballGCN(X^s, A^s) = normalize(L^{sp} C^s W_{cs}), \quad \text{(Equation 13)}$$

$$H_c^d = SnowballGCN(X^d, A^d) = normalize\left(L^{dp} C^d W_{cs}\right), \quad \text{(Equation 14)}$$

where H_c^f, H_c^s, H_c^d are the MC representations for rs-fMRI, sMRI and demographic modalities, respectively, and W_{cs} is the shared trainable matrix. By sharing weights in this way, MC features can be filtered out. In this experiment, we selected three modalities and two brain atlases for the experiment, and the resulting common embeddings are denoted as $H_c^{fAAL}, H_c^{fCC200}, H_c^s, and\ H_c^d$. The final common embedding is obtained by the weighted sum of the four $H_c = \alpha H_c^{fAAL} + \beta H_c^{fCC200} + \gamma H_c^s + \epsilon H_c^d$, where $\alpha, \beta, \gamma,$ and ϵ are hyperparameters measuring the importance of each MC embedding. In the implementation, we set $\alpha, \beta, \gamma,$ and ϵ to be equal and sum to 1 to pay equal attention to all modalities.

M-attention block. The contribution of different modes of information to the final diagnosis varies with the target disease. To apply more attention to the effective modes and make the less important modes only play an auxiliary role, we use the attention mechanism on four specific embeddings, $H_s^{fAAL}, H_s^{fCC200}, H_s^s, and\ H_s^d,$ and one common embedding, H_c:

$$a_{fAAL}, a_{fCC200}, a_s, a_p, a_c = Attention\left(H_s^{fAAL}, H_s^{fCC200}, H_s^s, H_s^d, H_c\right) \quad \text{(Equation 15)}$$

$$e_i = \mathrm{Tanh}\left(W_{ai} H_S^i + b_i\right) \quad \text{(Equation 16)}$$

$$a_i = \frac{\exp(e_i)}{\exp(e_{fAAL}) + \exp(e_{fCC200}) + \exp(e_s) + \exp(e_d) + \exp(e_c)},\ i \in \left\{f_{AAL}, f_{CC200}, s, d, c\right\} \quad \text{(Equation 17)}$$

After obtaining the attention score, the final embedding can be calculated by combining the representation and the weight as follows:

$$H = a_{fAAL} H_s^{fAAL} + a_{fCC200} H_s^{fCC200} + a_s H_s^s + a_p H_s^d + a_c H_C \quad \text{(Equation 18)}$$

Objective function

The overall objective function of LGMF-GNN contains three kinds of loss terms as follows:

$$L = L_{cls} + L_{specific} + L_{common} + L_{domain} \quad \text{(Equation 19)}$$

Classification loss. We chose cross-entropy loss as the classification loss function. It should be noted that the proposed model can make predictions in both local and global stages, so the classification loss is a weighted sum of the two parts:

$$L_{cls} = L_{cls_{global}} + \lambda L_{cls_{local}}, \quad \text{(Equation 20)}$$

where λ is the weight hyperparameter of the local classification loss. In our implementation, we set λ to 0.2 to ensure that the global classification loss plays a dominant role in the overall optimization process.

Modality independence loss. As mentioned above in the network structure section, we hope to extract features that are independent and shared between modalities separately. However, our extractor is based on the same structure. To facilitate the separation of MS and MC embeddings, we impose the modality-independence constraint on them. Specifically, the Hilbert-Schmidt independence criterion[118] is used to measure the dissimilarity of the two sets of embedding distributions:

$$HSIC(H_s, H_c) = (m-1)^{-2} tr(K_c R K_s R), \quad \text{(Equation 21)}$$

$$K\left(H^i, H^j\right) = <\phi\left(H^i\right), \phi\left(H^j\right)>, \quad \text{(Equation 22)}$$

$$R = I - \frac{1}{m} ee^T, \quad \text{(Equation 23)}$$

where I is the unit matrix, e is a column vector with all values of 1, ϕ is a kernel function that maps the input to reproducing kernel Hilbert space, and $<>$ denotes the inner product of the inputs. In this way, we obtain the loss function:

$$\begin{aligned} L_{specific} = &\ HSIC\left(H_s^{fAAL}, H_c\right) + HSIC\left(H_s^{fCC200}, H_c\right) \\ &+ HSIC\left(H_s^s, H_c\right) + HSIC\left(H_s^d, H_c\right). \end{aligned} \quad \text{(Equation 24)}$$

Modality similarity loss. To extract the modality-shared features, we designed the MC-GCN block, which is a snowball GCN with shared weights. Apart from the model structure design, the modality similarity constraint is proposed to further encourage the similarity of MC embeddings across modalities. Specifically, the L_2-norm is calculated between normalized embeddings of different modalities:

$$N_c = H_c \cdot H_c^T \quad \text{(Equation 25)}$$

$$L_{common} = \|N_c^f - N_c^p\|^2 + \|N_c^f - N_c^s\|^2 + \|N_c^d - N_c^s\|^2 \quad \text{(Equation 26)}$$

Domain migration loss. To alleviate site effects during the training stage, the domain adversarial training strategy was used, and a domain migration loss was designed to reduce the difference between the generated embeddings in the source and target domains. The adversarial training is achieved with a site classifier and a gradient inversion layer, and domain migration loss is designed based on the central moment discrepancy (CMD) distance. Thus, the total domain loss is defined as:

$$L_{domain} = L_{site} + L_{CMD}$$

where L_{site} is the cross-entropy loss between the site prediction result of the site classifier and the real site label. L_{CMD} is the CMD distance between the source and target domain. More detailed information can be found in the "domain adversarial training" and "domain migration loss" sections of the supplemental experimental procedures.

Implementation details

The proposed LGMF-GNN was implemented in Python 3.7 and PyTorch 1.12.1 on an NVIDIA TITAN RTX GPU. The Adam optimizer was used with a learning

CellPress
OPEN ACCESS

rate of 1e−2 and halved every 100 epochs. We allowed the model to run for at most 800 epochs for all experiments. Based on the experimental results and analysis presented in Table S23 and "the impact of brain atlases on model performance" section of the supplemental experimental procedures, we ultimately used the AAL brain atlas for brain ROI definition, model training, and interpretable research. The parameters α,β,γ, and ϵ, which govern the modality weights in the final common embedding H_c, were assigned equal values to sum to 1, ensuring balanced consideration across all modalities. The weight hyperparameter λ of the local classification loss was fixed at 0.2 to prioritize the global classification loss in the overall optimization. Grid searches were performed to determine the hyperparameter k, representing the number of neighbors in the KNN population graph construction, and n, indicating the number of layers in the snowball GCN. The optimal values for these parameters were found to be $k = 10$ and $n = 9$. A comprehensive discussion and detailed results concerning hyperparameter selection are provided in Figures S8 and S9 and the "hyperparameter selection" section of the supplemental experimental procedures.

Methods for interpretable research on the model

Interpretation of the functional modality

Interpretability experiments for the functional modality were conducted on the combined dataset from the SRPBS and REST-meta-MDD studies, which provided a sufficient sample size to ensure the statistical power of our findings. The local ROI GNN is designed to construct a learnable adjacency matrix by capturing the deep and dynamic temporal feature correlations of the ROI BOLD signals. This matrix was optimized for both local and global tasks. Consequently, the trained LGMF-GNN can predict an adjacency matrix based on the input fMRI ROI signals. This predicted matrix not only encompasses deeper temporal features but it also integrates demographic and anatomical constraints, surpassing traditional matrices that rely solely on Pearson correlation coefficients. By analyzing these learned adjacency matrices, or functional connectivity patterns, from the HC and MDD groups, we can elucidate the underlying mechanisms of the functional states of MDD.

During model training, we conducted 10-fold CV experiments on the SRPBS and REST-meta-MDD datasets, which means each sample in the dataset was added to the training set nine times. We used the model obtained from each fold to perform inference on the training set corresponding to that fold and saved the functional connectivity matrix obtained by local ROI GNN during inference. In this way, for each sample in the dataset, we obtained nine learned functional connectivity matrices. These functional connectivity matrices are subsequently averaged to produce a single functional connectivity matrix for each sample. Then, we utilized the diagnostic labels indicating whether each sample was diagnosed with MDD to split the dataset into two groups: HC and MDD. We separately averaged the learned functional connectivity matrices for all samples within each group. This resulted in the average functional connectivity matrices for the HC and MDD groups, as depicted in Figure 5A. The differential functional connectivity matrix (MDD-HC) is obtained by subtracting these two matrices.

The interpretability of the variation of functional connectivity between HC and MDD was based on the differential functional connectivity matrix. Specifically, we extracted the row and column coordinates corresponding to the five maximum and minimum values in the matrix. The functional connections between brain regions indexed by these coordinates represent the enhanced and weakened FCs in MDD compared to HC. The interpretability of the most contributive brain regions was based on the differential functional connectivity matrix after taking absolute values. By summing the rows of this matrix, we obtained the degree of each node, with a higher degree indicating a greater difference in functional connections associated with that brain region between the MDD and HC groups. Thus, we selected the 10 nodes with the highest degrees as the most diagnostically informative brain regions.

Interpretation of the anatomical modality

For anatomical modality, the analysis was conducted on the SRPBS dataset. Following the steps outlined in the data processing section, we obtained a 1,209-dimensional feature vector as input for the anatomical modality, encompassing 93 radiomic characters that characterize the anatomical properties of the brain tissue. To identify the key radiomic characters contributing to the diagnosis of MDD, we employed a feature-masking strategy to assess the impact of a specific radiomic character on model performance. Specifically, using the model trained with 10-fold CV for inference, we masked, or set to zero, the elements corresponding to the vector position of each radiomic feature during the inference process. This yielded the average performance across the 10-fold CVs after masking. The performance of the model was measured by the AUROC. By comparing the average inference AUROC of 10-fold CV results obtained from LGMF-GNN with unmasked inputs to those with masked inputs, we evaluated the performance loss of each radiomic character. We identified the top five radiomic characters with the greatest performance loss as the most contributive to diagnosis.

RESOURCE AVAILABILITY

Lead contact

Cheng Jin is the lead contact for this study and can be reached at chengjin520@sjtu.edu.cn.

Materials availability

This study did not generate new unique reagents.

Data and code availability

- All of the code associated with this study is publicly available online at https://github.com/ATP-BME/LGMF-GNN and has been archived at a Figshare repository[119] at https://doi.org/10.6084/m9.figshare.27107440.
- The SRPBS dataset is available at https://doi.org/10.7303/syn22317081.
- The REST-meta-MDD dataset is available at https://doi.org/10.57760/sciencedb.o00115.00013.
- Lists of members and their affiliations for the DIRECT Consortium appear in the supplemental information as Table S25.
- The OpenNeuro dataset is available at https://openneuro.org/datasets/ds002748.

ACKNOWLEDGMENTS

This study was funded by the Sci-Tech Innovation 2030 - Major Project of Brain Science and Brain-inspired Intelligence Technology (grant no. 2021ZD0200600), the National Key Research and Development Program of China (grant no. 2022YFB4702702) and the Shanghai Committee of Science and Technology, China (grant no. 21DZ1100301). We would also like to acknowledge the data curators and providers who meticulously organized and maintained the dataset. Their dedication in ensuring the accessibility and quality of the data has been paramount to our research.

AUTHOR CONTRIBUTIONS

C.J., G.W., L.Z., and Y.W. contributed to the conception of the study and offered method guidance. S.L. designed the algorithm, developed the structure, performed the experiment, and wrote the first and final draft of this manuscript. J.Z., X. Zhu, and X. Zhou performed the data preprocessing and offered clinical guidance. Y.Z. and S.Z. helped perform the analysis with constructive discussions. Z.Y. and X.C. contributed help in data preprocessing. Z.W. contributed significantly to manuscript preparation. R.W., Y.Y., and X.F. provided valuable suggestions for the experiments. All the authors reviewed and approved the final manuscript.

DECLARATION OF INTERESTS

The authors declare no competing interests.

SUPPLEMENTAL INFORMATION

Supplemental information can be found online at https://doi.org/10.1016/j.patter.2024.101081.

Received: May 6, 2024
Revised: September 9, 2024
Accepted: October 7, 2024
Published: November 4, 2024

REFERENCES

1. Joyce-Beaulieu, D., and Sulkowski, M.L. (2016). The Diagnostic and Statistical Manual of Mental Disorders: Fifth Edition (DSM-5) Model of Impairment. In Assessing Impairment: From Theory to Practice, S. Goldstein and J.A. Naglieri, eds. (Springer US), pp. 167–189. https://doi.org/10.1007/978-1-4899-7996-4_8.
2. Institute for Health Metrics and Evaluation (2019). Global Health Data Exchange (GHDx). https://vizhub.healthdata.org/gbd-results/?params=gbd-api-2019-permalink/d780dffbe8a381b25e1416884959e88b.
3. National Institute of Mental Health (2022). Depression. https://www.nimh.nih.gov/health/topics/depression.
4. Prendes-Alvarez, S., and Nemeroff, C.B. (2018). Personalized medicine: Prediction of disease vulnerability in mood disorders. Neurosci. Lett. *669*, 10–13. https://doi.org/10.1016/j.neulet.2016.09.049.
5. Association, A.P. (2013). Diagnostic and Statistical Manual of Mental, Disorders-5th Edition. https://doi.org/10.1176/appi.books.9780890425596.
6. Marx, W., Penninx, B.W.J.H., Solmi, M., Furukawa, T.A., Firth, J., Carvalho, A.F., and Berk, M. (2023). Major depressive disorder. Nat. Rev. Dis. Primers *9*, 44. https://doi.org/10.1038/s41572-023-00454-1.
7. Hamilton, M. (1960). A RATING SCALE FOR DEPRESSION. J. Neurol. Neurosurg. Psychiatry *23*, 56–62. https://doi.org/10.1136/jnnp.23.1.56.
8. HAMILTON, M. (1967). Development of a Rating Scale for Primary Depressive Illness. Br. J. Soc. Clin. Psychol. *6*, 278–296. https://doi.org/10.1111/j.2044-8260.1967.tb00530.x.
9. Beck, A.T., Ward, C.H., Mendelson, M., Mock, J., and Erbaugh, J. (1961). An Inventory for Measuring Depression. Arch. Gen. Psychiatry *4*, 561–571. https://doi.org/10.1001/archpsyc.1961.01710120031004.
10. El-Den, S., Chen, T.F., Gan, Y.-L., Wong, E., and O'Reilly, C.L. (2018). The psychometric properties of depression screening tools in primary healthcare settings: A systematic review. J. Affect. Disord. *225*, 503–522. https://doi.org/10.1016/j.jad.2017.08.060.
11. Qin, K., Lei, D., Pinaya, W.H.L., Pan, N., Li, W., Zhu, Z., Sweeney, J.A., Mechelli, A., and Gong, Q. (2022). Using graph convolutional network to characterize individuals with major depressive disorder across multiple imaging sites. EBioMedicine *78*, 103977. https://doi.org/10.1016/j.ebiom.2022.103977.
12. Schmaal, L., Hibar, D.P., Sämann, P.G., Hall, G.B., Baune, B.T., Jahanshad, N., Cheung, J.W., van Erp, T.G.M., Bos, D., Ikram, M.A., et al. (2017). Cortical abnormalities in adults and adolescents with major depression based on brain scans from 20 cohorts worldwide in the ENIGMA Major Depressive Disorder Working Group. Mol. Psychiatry *22*, 900–909. https://doi.org/10.1038/mp.2016.60.
13. van Velzen, L.S., Kelly, S., Isaev, D., Aleman, A., Aftanas, L.I., Bauer, J., Baune, B.T., Brak, I.V., Carballedo, A., Connolly, C.G., et al. (2020). White matter disturbances in major depressive disorder: a coordinated analysis across 20 international cohorts in the ENIGMA MDD working group. Mol. Psychiatry *25*, 1511–1525. https://doi.org/10.1038/s41380-019-0477-2.
14. Chen, F., Bertelsen, A.B., Holm, I.E., Nyengaard, J.R., Rosenberg, R., and Dorph-Petersen, K.-A. (2020). Hippocampal volume and cell number in depression, schizophrenia, and suicide subjects. Brain Res. *1727*, 146546. https://doi.org/10.1016/j.brainres.2019.146546.
15. Goldstein-Piekarski, A.N., Ball, T.M., Samara, Z., Staveland, B.R., Keller, A.S., Fleming, S.L., Grisanzio, K.A., Holt-Gosselin, B., Stetz, P., Ma, J., and Williams, L.M. (2022). Mapping Neural Circuit Biotypes to Symptoms and Behavioral Dimensions of Depression and Anxiety. Biol. Psychiatry *91*, 561–571. https://doi.org/10.1016/j.biopsych.2021.06.024.
16. Tang, S., Lu, L., Zhang, L., Hu, X., Bu, X., Li, H., Hu, X., Gao, Y., Zeng, Z., Gong, Q., and Huang, X. (2018). Abnormal amygdala resting-state functional connectivity in adults and adolescents with major depressive disorder: A comparative meta-analysis. EBioMedicine *36*, 436–445. https://doi.org/10.1016/j.ebiom.2018.09.010.
17. Herrman, H., Patel, V., Kieling, C., Berk, M., Buchweitz, C., Cuijpers, P., Furukawa, T.A., Kessler, R.C., Kohrt, B.A., Maj, M., et al. (2022). Time for united action on depression: a Lancet–World Psychiatric Association Commission. Lancet *399*, 957–1022. https://doi.org/10.1016/S0140-6736(21)02141-3.
18. Fries, G.R., Saldana, V.A., Finnstein, J., and Rein, T. (2023). Molecular pathways of major depressive disorder converge on the synapse. Mol. Psychiatry *28*, 284–297. https://doi.org/10.1038/s41380-022-01806-1.
19. Yan, C.-G., Chen, X., Li, L., Castellanos, F.X., Bai, T.-J., Bo, Q.-J., Cao, J., Chen, G.-M., Chen, N.-X., Chen, W., et al. (2019). Reduced default mode network functional connectivity in patients with recurrent major depressive disorder. Proc. Natl. Acad. Sci. USA *116*, 9078–9083. https://doi.org/10.1073/pnas.1900390116.
20. Kaiser, R.H., Andrews-Hanna, J.R., Wager, T.D., and Pizzagalli, D.A. (2015). Large-Scale Network Dysfunction in Major Depressive Disorder A Meta-analysis of Resting-State Functional Connectivity. JAMA Psychiatr. *72*, 603–611. https://doi.org/10.1001/jamapsychiatry.2015.0071.
21. McTeague, L.M., Rosenberg, B.M., Lopez, J.W., Carreon, D.M., Huemer, J., Jiang, Y., Chick, C.F., Eickhoff, S.B., and Etkin, A. (2020). Identification of Common Neural Circuit Disruptions in Emotional Processing Across Psychiatric Disorders. Am. J. Psychiatry *177*, 411–421. https://doi.org/10.1176/appi.ajp.2019.18111271.
22. Insel, T.R., and Cuthbert, B.N. (2015). Brain disorders? Precisely. Science *348*, 499–500. https://doi.org/10.1126/science.aab2358.
23. Williams, L.M. (2016). Precision psychiatry: a neural circuit taxonomy for depression and anxiety. Lancet Psychiatr. *3*, 472–480. https://doi.org/10.1016/S2215-0366(15)00579-9.
24. Lui, S., Zhou, X.J., Sweeney, J.A., and Gong, Q. (2016). Psychoradiology: the frontier of neuroimaging in psychiatry. Radiology *281*, 357–372.
25. Mousavian, M., Chen, J., Traylor, Z., and Greening, S. (2021). Depression detection from sMRI and rs-fMRI images using machine learning. J. Intell. Inf. Syst. *57*, 395–418. https://doi.org/10.1007/s10844-021-00653-w.
26. Hojjati, S.H., Ebrahimzadeh, A., Babajani-Feremi, A., and Alzheimers Dis Neuroimaging, I. (2019). Identification of the Early Stage of Alzheimer's Disease Using Structural MRI and Resting-State fMRI. Front. Neurol. *10*, 904. https://doi.org/10.3389/fneur.2019.00904.
27. Drysdale, A.T., Grosenick, L., Downar, J., Dunlop, K., Mansouri, F., Meng, Y., Fetcho, R.N., Zebley, B., Oathes, D.J., Etkin, A., et al. (2017). Resting-state connectivity biomarkers define neurophysiological subtypes of depression. Nat. Med. *23*, 28–38. https://doi.org/10.1038/nm.4246.
28. Li, M., Das, T., Deng, W., Wang, Q., Li, Y., Zhao, L., Ma, X., Wang, Y., Yu, H., Li, X., et al. (2017). Clinical utility of a short resting-state MRI scan in differentiating bipolar from unipolar depression. Acta Psychiatr. Scand. *136*, 288–299. https://doi.org/10.1111/acps.12752.
29. Christian, B., Ronny, R., Dominik, G., Susanne, M., and Dohm, K. (2017). Differential Abnormal Pattern of Anterior Cingulate Gyrus Activation in Unipolar and Bipolar Depression: an fMRI and Pattern Classification Approach. Neuropsychopharmacology *42*, 1399–1408.
30. Sato, J.R., Moll, J., Green, S., Deakin, J.F.W., Thomaz, C.E., and Zahn, R. (2015). Machine learning algorithm accurately detects fMRI signature of vulnerability to major depression. Psychiatry Res. *233*, 289–291.
31. Gao, S., Calhoun, V.D., and Sui, J. (2018). Machine learning in major depression: From classification to treatment outcome prediction. CNS Neurosci. Ther. *24*, 1037–1052. https://doi.org/10.1111/cns.13048.
32. Akhavan Aghdam, M., Sharifi, A., and Pedram, M.M. (2018). Combination of rs-fMRI and sMRI Data to Discriminate Autism Spectrum Disorders in Young Children Using Deep Belief Network. J. Digit. Imaging *31*, 895–903. https://doi.org/10.1007/s10278-018-0093-8.
33. Zhang, L., Wang, M., Liu, M., and Zhang, D. (2020). A survey on deep learning for neuroimaging-based brain disorder analysis. Front. Neurosci. *14*, 779.

CellPress
OPEN ACCESS

34. Qin, Z., Liu, Z., and Zhu, P. (2022). Aiding Alzheimer's Disease Diagnosis Using Graph Convolutional Networks Based on rs-fMRI Data. 2022 15th International Congress on Image and Signal Processing, BioMedical Engineering and Informatics (CISP-BMEI), Beijing, China, pp. 1–7. https://doi.org/10.1109/CISP-BMEI56279.2022.9980159.

35. Li, X., Zhou, Y., Dvornek, N.C., Zhang, M., Zhuang, J., Ventola, P., and Duncan, J.S. (2020). Pooling regularized graph neural network for fmri biomarker analysis. Medical Image Computing and Computer Assisted Intervention – MICCAI 2020. *Part VII 23*, 625–635. https://doi.org/10.1007/978-3-030-59728-3_61

36. Parisot, S., Ktena, S.I., Ferrante, E., Lee, M., Guerrero, R., Glocker, B., and Rueckert, D. (2018). Disease prediction using graph convolutional networks: application to autism spectrum disorder and Alzheimer's disease. Med. Image Anal. *48*, 117–130.

37. Ktena, S.I., Parisot, S., Ferrante, E., Rajchl, M., Lee, M., Glocker, B., and Rueckert, D. (2018). Metric learning with spectral graph convolutions on brain connectivity networks. Neuroimage *169*, 431–442. https://doi.org/10.1016/j.neuroimage.2017.12.052.

38. Li, X., Zhou, Y., Dvornek, N., Zhang, M., Gao, S., Zhuang, J., Scheinost, D., Staib, L.H., Ventola, P., and Duncan, J.S. (2021). BrainGNN: Interpretable Brain Graph Neural Network for fMRI Analysis. Med. Image Anal. *74*, 102233. https://doi.org/10.1016/j.media.2021.102233.

39. Pan, J., Lin, H., Dong, Y., Wang, Y., and Ji, Y. (2022). MAMF-GCN: Multi-scale adaptive multi-channel fusion deep graph convolutional network for predicting mental disorder. Comput. Biol. Med. *148*, 105823. https://doi.org/10.1016/j.compbiomed.2022.105823.

40. Chen, T., Hong, R., Guo, Y., Hao, S., and Hu, B. (2022). MS^2 -GNN: Exploring GNN-Based Multimodal Fusion Network for Depression Detection. IEEE Trans. Cybern. *53*, 7749–7759. https://doi.org/10.1109/TCYB.2022.3197127.

41. Liu, Y., He, L., Cao, B., Yu, P., Ragin, A., and Leow, A. (2018). Multi-view multi-graph embedding for brain network clustering analysis. Proceedings of the AAAI conference on artificial intelligence AAAI 2018 *32*, 117–124. https://doi.org/10.1609/aaai.v32i1.11288.

42. Xing, X., Li, Q., Wei, H., Zhang, M., Zhan, Y., Zhou, X.S., Xue, Z., and Shi, F. (2019). Dynamic Spectral Graph Convolution Networks with Assistant Task Training for Early MCI Diagnosis. Medical Image Computing and Computer Assisted Intervention – MICCAI *2019*, 639–646. https://doi.org/10.1007/978-3-030-32251-9_70.

43. Chen, Y., Yan, J., Jiang, M., Zhang, T., Zhao, Z., Zhao, W., Zheng, J., Yao, D., Zhang, R., Kendrick, K.M., and Jiang, X. (2024). Adversarial Learning Based Node-Edge Graph Attention Networks for Autism Spectrum Disorder Identification. IEEE Trans. Neural Netw. Learn. Syst. *35*, 7275–7286. https://doi.org/10.1109/tnnls.2022.3154755.

44. Pitsik, E.N., Maximenko, V.A., Kurkin, S.A., Sergeev, A.P., Stoyanov, D., Paunova, R., Kandilarova, S., Simeonova, D., and Hramov, A.E. (2023). The topology of fMRI-based networks defines the performance of a graph neural network for the classification of patients with major depressive disorder. Chaos, Solit. Fractals *167*, 113041. https://doi.org/10.1016/j.chaos.2022.113041.

45. Li, X., Dvornek, N.C., Zhou, Y., Zhuang, J., Ventola, P., and Duncan, J.S. (2019). Graph Neural Network for Interpreting Task-fMRI Biomarkers. Medical Image Computing and Computer Assisted Intervention – MICCAI 2019, 485–493. https://doi.org/10.1007/978-3-030-32254-0_54.

46. Yan, C.G., Craddock, R.C., Zuo, X.N., Zang, Y.F., and Milham, M.P. (2013). Standardizing the intrinsic brain: Towards robust measurement of inter-individual variation in 1000 functional connectomes. Neuroimage *80*, 246–262. https://doi.org/10.1016/j.neuroimage.2013.04.081.

47. Kipf, T.N., and Welling, M. (2016). Semi-supervised classification with graph convolutional networks. Preprint at arXiv. https://doi.org/10.48550/arXiv.1609.02907.

48. Xu, K., Hu, W., Leskovec, J., and Jegelka, S. (2019). How Powerful Are Graph Neural Networks? In International Conference on Learning Representations - ICLR 2019, pp. 1–7. https://openreview.net/forum?id=ryGs6iA5Kmdoi:.

49. Veličković, P., Cucurull, G., Casanova, A., Romero, A., Lio, P., and Bengio, Y. (2018). Graph attention networks. In International Conference on Learning Representations -ICLR *2018*, pp. 1–12. https://openreview.net/forum?id=rJXMpikCZ.

50. Huang, Y., and Chung, A.C.S. (2020). Edge-Variational Graph Convolutional Networks for Uncertainty-Aware Disease Prediction. Medical Image Computing and Computer Assisted Intervention – MICCAI 2020, 562–572. https://doi.org/10.1007/978-3-030-59728-3_55.

51. Zhang, H., Song, R., Wang, L., Zhang, L., Wang, D., Wang, C., and Zhang, W. (2023). Classification of Brain Disorders in rs-fMRI via Local-to-Global Graph Neural Networks. IEEE Trans. Med. Imaging *42*, 444–455. https://doi.org/10.1109/TMI.2022.3219260.

52. Xu, J., Bian, Q., Li, X., Zhang, A., Ke, Y., Qiao, M., Zhang, W., Khang Jeremy Sim, W., and Gulyás, B. (2024). Contrastive Graph Pooling for Explainable Classification of Brain Networks. IEEE Trans. Med. Imaging *43*, 3292–3305. https://doi.org/10.1109/TMI.2024.3392988.

53. Gu, X., Xie, L., Xia, Y., Cheng, Y., Liu, L., and Tang, L. (2023). Autism spectrum disorder diagnosis using the relational graph attention network. Biomed. Signal Process Control *85*, 105090. https://doi.org/10.1016/j.bspc.2023.105090.

54. Zhang, J., Wang, Q., Wang, X., Qiao, L., and Liu, M. (2024). Preserving specificity in federated graph learning for fMRI-based neurological disorder identification. Neural Netw. *169*, 584–596. https://doi.org/10.1016/j.neunet.2023.11.004.

55. Zheng, K., Yu, S., and Chen, B. (2024). CI-GNN: A Granger causality-inspired graph neural network for interpretable brain network-based psychiatric diagnosis. Neural Netw. *172*, 106147. https://doi.org/10.1016/j.neunet.2024.106147.

56. Cui, H., Dai, W., Zhu, Y., Li, X., He, L., and Yang, C. (2022). Interpretable Graph Neural Networks for Connectome-Based Brain Disorder Analysis. Medical Image Computing and Computer Assisted Intervention – MICCAI 2022, 375–385. https://doi.org/10.1007/978-3-031-16452-1_36.

57. Duman, R.S., and Aghajanian, G.K. (2012). Synaptic Dysfunction in Depression: Potential Therapeutic Targets. Science *338*, 68–72. https://doi.org/10.1126/science.1222939.

58. Hiser, J., and Koenigs, M. (2018). The Multifaceted Role of the Ventromedial Prefrontal Cortex in Emotion, Decision Making, Social Cognition, and Psychopathology. Biol. Psychiatry *83*, 638–647. https://doi.org/10.1016/j.biopsych.2017.10.030.

59. Zeng, L.L., Shen, H., Liu, L., Wang, L., Li, B., Fang, P., Zhou, Z., Li, Y., and Hu, D. (2012). Identifying major depression using whole-brain functional connectivity: a multivariate pattern analysis. Brain *135*, 1498–1507. https://doi.org/10.1093/brain/aws059.

60. Spellman, T., and Liston, C. (2020). Toward circuit mechanisms of pathophysiology in depression. Am. J. Psychiatry *177*, 381–390.

61. Liu, L., Zeng, L.L., Li, Y., Ma, Q., Li, B., Shen, H., and Hu, D. (2012). Altered cerebellar functional connectivity with intrinsic connectivity networks in adults with major depressive disorder. PLoS One *7*, e39516. https://doi.org/10.1371/journal.pone.0039516.

62. Xu, L.-Y., Xu, F.-C., Liu, C., Ji, Y.-F., Wu, J.-M., Wang, Y., Wang, H.-B., and Yu, Y.-Q. (2017). Relationship between cerebellar structure and emotional memory in depression. Brain Behav. *7*, e00738. https://doi.org/10.1002/brb3.738.

63. Yan, Y., Liu, G., Cai, H., Wu, E.Q., Cai, J., Cheok, A.D., Liu, N., Li, T., and Fan, Z. (2024). A review of graph theory-based diagnosis of neurological disorders based on EEG and MRI. Neurocomputing *599*, 128098. https://doi.org/10.1016/j.neucom.2024.128098.

64. Khemani, B., Patil, S., Kotecha, K., and Tanwar, S. (2024). A review of graph neural networks: concepts, architectures, techniques, challenges, datasets, applications, and future directions. J. Big Data *11*, 18. https://doi.org/10.1186/s40537-023-00876-4.

65. Bessadok, A., Mahjoub, M.A., and Rekik, I. (2023). Graph Neural Networks in Network Neuroscience. IEEE Trans. Pattern Anal. Mach. Intell. *45*, 5833–5848. https://doi.org/10.1109/TPAMI.2022.3209686.

66. Li, R., Yuan, X., Radfar, M., Marendy, P., Ni, W., O'Brien, T.J., and Casillas-Espinosa, P. (2023). Graph Signal Processing, Graph Neural Network and Graph Learning on Biological Data: A Systematic Review. IEEE Rev. Biomed. Eng. *16*, 109–135. https://doi.org/10.1109/RBME.2021.3122522.

67. Li, M.M., Huang, K., and Zitnik, M. (2022). Graph representation learning in biomedicine and healthcare. Nat. Biomed. Eng. *6*, 1353–1369. https://doi.org/10.1038/s41551-022-00942-x.

68. He, L., Niu, M., Tiwari, P., Marttinen, P., Su, R., Jiang, J., Guo, C., Wang, H., Ding, S., Wang, Z., et al. (2022). Deep learning for depression recognition with audiovisual cues: A review. Inf. Fusion *80*, 56–86. https://doi.org/10.1016/j.inffus.2021.10.012.

69. Zhuo, C., Li, G., Lin, X., Jiang, D., Xu, Y., Tian, H., Wang, W., and Song, X. (2019). The rise and fall of MRI studies in major depressive disorder. Transl. Psychiatry *9*, 335. https://doi.org/10.1038/s41398-019-0680-6.

70. Hasib, K.M., Islam, M.R., Sakib, S., Akbar, M.A., Razzak, I., and Alam, M.S. (2023). Depression Detection From Social Networks Data Based on Machine Learning and Deep Learning Techniques: An Interrogative Survey. IEEE Trans. Comput. Soc. Syst. *10*, 1568–1586. https://doi.org/10.1109/TCSS.2023.3263128.

71. Fateh, A.A., Long, Z., Duan, X., Cui, Q., Pang, Y., Farooq, M.U., Nan, X., Chen, Y., Sheng, W., Tang, Q., and Chen, H. (2019). Hippocampal functional connectivity-based discrimination between bipolar and major depressive disorders. Psychiatr. Res. Neuroimaging *284*, 53–60. https://doi.org/10.1016/j.pscychresns.2019.01.004.

72. Fan, J., Gao, F., Wang, X., Liu, Q., Xia, J., Han, Y., Yi, J., Tan, C., and Zhu, X. (2023). Right amygdala-right precuneus connectivity is associated with childhood trauma in major depression patients and healthy controls. Soc. Cogn. Affect. Neurosci. *18*, nsac064. https://doi.org/10.1093/scan/nsac064.

73. Jacob, Y., Morris, L.S., Verma, G., Rutter, S.B., Balchandani, P., and Murrough, J.W. (2022). Altered hippocampus and amygdala subregion connectome hierarchy in major depressive disorder. Transl. Psychiatry *12*, 209. https://doi.org/10.1038/s41398-022-01976-0.

74. Shengli, C., Yingli, Z., Zheng, G., Shiwei, L., Ziyun, X., Han, F., Yingwei, Q., and Gangqiang, H. (2022). An aberrant hippocampal subregional network, rather than structure, characterizes major depressive disorder. J. Affect. Disord. *302*, 123–130. https://doi.org/10.1016/j.jad.2022.01.087.

75. Hu, J., Liu, J., Liu, Y., Wu, X., Zhuang, K., Chen, Q., Yang, W., Xie, P., Qiu, J., and Wei, D. (2021). Dysfunction of the anterior and intermediate hippocampal functional network in major depressive disorders across the adult lifespan. Biol. Psychol. *165*, 108192. https://doi.org/10.1016/j.biopsycho.2021.108192.

76. Kaymak, S.U., Demir, B., Sentürk, S., Tatar, I., Aldur, M.M., and Uluğ, B. (2010). Hippocampus, glucocorticoids and neurocognitive functions in patients with first-episode major depressive disorders. Eur. Arch. Psychiatry Clin. Neurosci. *260*, 217–223. https://doi.org/10.1007/s00406-009-0045-x.

77. Cole, J., Toga, A.W., Hojatkashani, C., Thompson, P., Costafreda, S.G., Cleare, A.J., Williams, S.C.R., Bullmore, E.T., Scott, J.L., Mitterschiffthaler, M.T., et al. (2010). Subregional hippocampal deformations in major depressive disorder. J. Affect. Disord. *126*, 272–277. https://doi.org/10.1016/j.jad.2010.03.004.

78. Jun, X., Jun, C., Yicheng, Z., Jingfeng, Z., Bo, Y., Liming, X., and Chengyuan, W. (2004). Volumetric MRI analysis of the amygdala and hippocampus in subjects with major depression. Curr. Med. Sci. *24*, 500–502. https://doi.org/10.1007/BF02831120.

79. Cole, J., Costafreda, S.G., McGuffin, P., and Fu, C.H.Y. (2011). Hippocampal atrophy in first episode depression: A meta-analysis of magnetic resonance imaging studies. J. Affect. Disord. *134*, 483–487. https://doi.org/10.1016/j.jad.2011.05.057.

80. MacMaster, F.P., and Kusumakar, V. (2004). Hippocampal volume in early onset depression. BMC Med. *2*, 2. https://doi.org/10.1186/1741-7015-2-2.

81. Chen, L., Wang, Y., Niu, C., Zhong, S., Hu, H., Chen, P., Zhang, S., Chen, G., Deng, F., Lai, S., et al. (2018). Common and distinct abnormal frontal-limbic system structural and functional patterns in patients with major depression and bipolar disorder. Neuroimage. Clin. *20*, 42–50. https://doi.org/10.1016/j.nicl.2018.07.002.

82. Xiong, Y., Chen, R.-S., Wang, X.-Y., Li, X., Dai, L.-Q., and Yu, R.-Q. (2022). Cerebral blood flow in adolescents with drug-naive, first-episode major depressive disorder: An arterial spin labeling study based on voxel-level whole-brain analysis. Front. Neurosci. *16*, 966087. https://doi.org/10.3389/fnins.2022.966087.

83. Keller, M., Mendoza-Quiñones, R., Cabrera Muñoz, A., Iglesias-Fuster, J., Virués, A.V., Zvyagintsev, M., Edgar, J.C., Zweerings, J., and Mathiak, K. (2022). Transdiagnostic alterations in neural emotion regulation circuits – neural substrates of cognitive reappraisal in patients with depression and post-traumatic stress disorder. BMC Psychiatr. *22*, 173. https://doi.org/10.1186/s12888-022-03780-y.

84. Qiao, J., Tao, S., Wang, X., Shi, J., Chen, Y., Tian, S., Yao, Z., and Lu, Q. (2020). Brain functional abnormalities in the amygdala subregions is associated with anxious depression. J. Affect. Disord. *276*, 653–659. https://doi.org/10.1016/j.jad.2020.06.077.

85. Liu, P., Tu, H., Zhang, A., Yang, C., Liu, Z., Lei, L., Wu, P., Sun, N., and Zhang, K. (2021). Brain functional alterations in MDD patients with somatic symptoms: A resting-state fMRI study. J. Affect. Disord. *295*, 788–796. https://doi.org/10.1016/j.jad.2021.08.143.

86. Liu, Y., Chen, Y., Liang, X., Li, D., Zheng, Y., Zhang, H., Cui, Y., Chen, J., Liu, J., and Qiu, S. (2020). Altered Resting-State Functional Connectivity of Multiple Networks and Disrupted Correlation With Executive Function in Major Depressive Disorder. Front. Neurol. *11*, 272. https://doi.org/10.3389/fneur.2020.00272.

87. Sun, J.-f., Chen, L.-m., He, J.-k., Wang, Z., Guo, C.-l., Ma, Y., Luo, Y., Gao, D.-q., Hong, Y., Fang, J.-l., and Xu, F.-q. (2022). A Comparative Study of Regional Homogeneity of Resting-State fMRI Between the Early-Onset and Late-Onset Recurrent Depression in Adults. Front. Psychol. *13*, 849847. https://doi.org/10.3389/fpsyg.2022.849847.

88. Zhang, L., Wei, X., and Zhao, J. (2022). Amplitude of Low-Frequency Oscillations in First-Episode Drug-Naive Patients with Major Depressive Disorder: A Resting State Functional Magnetic Resonance Imaging Study. Neuropsychiatr. Dis. Treat. *18*, 555–561. https://doi.org/10.2147/ndt.s348683.

89. Wu, X., Wang, L., Jiang, H., Fu, Y., Wang, T., Ma, Z., Wu, X., Wang, Y., Fan, F., Song, Y., and Lv, Y. (2023). Frequency-dependent and time-variant alterations of neural activity in post-stroke depression: A resting-state fMRI study. Neuroimage. Clin. *38*, 103445. https://doi.org/10.1016/j.nicl.2023.103445.

90. Sutoko, S., Atsumori, H., Obata, A., Funane, T., Kandori, A., Shimonaga, K., Hama, S., Yamawaki, S., and Tsuji, T. (2020). Lesions in the right Rolandic operculum are associated with self-rating affective and apathetic depressive symptoms for post-stroke patients. Sci. Rep. *10*, 20264. https://doi.org/10.1038/s41598-020-77136-5.

91. Depping, M.S., Schmitgen, M.M., Kubera, K.M., and Wolf, R.C. (2018). Cerebellar Contributions to Major Depression. Front. Psychiatry *9*, 634. https://doi.org/10.3389/fpsyt.2018.00634.

92. Ni, S., Gao, S., Ling, C., Jiang, J., Wu, F., Peng, T., Sun, J., Zhang, N., and Xu, X. (2023). Altered brain regional homogeneity is associated with cognitive dysfunction in first-episode drug-naive major depressive disorder: A resting-state fMRI study. J. Affect. Disord. *343*, 102–108. https://doi.org/10.1016/j.jad.2023.10.003.

93. Saloni Dattani, L.R.-G., Ritchie, H., and Roser, M. (2023). Mental Health. https://ourworldindata.org/mental-health.

94. Patel, M.J., Khalaf, A., and Aizenstein, H.J. (2016). Studying depression using imaging and machine learning methods. Neuroimage. Clin. *10*, 115–123. https://doi.org/10.1016/j.nicl.2015.11.003.

95. American Psychiatric Association (2024). What is Depression. https://www.psychiatry.org/patients-families/depression/what-is-depression.
96. Mousavian, M., Chen, J., and Greening, S. (2018). In Feature Selection and Imbalanced Data Handling for Depression Detection. held in Cham, S. Wang, V. Yamamoto, J. Su, Y. Yang, E. Jones, L. Iasemidis, and T. Mitchell, eds. (Springer International Publishing), pp. 349–358.
97. Hamilton, W., Ying, Z., and Leskovec, J. (2017). Inductive representation learning on large graphs. In Conference and Workshop on Neural Information Processing Systems - NeurIPS, pp. 1025–1035. https://dl.acm.org/doi/10.5555/3294771.3294869.
98. Chen J., Ma T., Xiao C. (2018). FastGCN: fast learning with graph convolutional networks via importance sampling. Preprint at arXiv, 1801.10247.
99. Tanaka, S.C., Yamashita, A., Yahata, N., Itahashi, T., Lisi, G., Yamada, T., Ichikawa, N., Takamura, M., Yoshihara, Y., Kunimatsu, A., et al. (2021). A multi-site, multi-disorder resting-state magnetic resonance image database. Sci. Data *8*, 227. https://doi.org/10.1038/s41597-021-01004-8.
100. Chen, X., Lu, B., Li, H.-X., Li, X.-Y., Wang, Y.-W., Castellanos, F.X., Cao, L.-P., Chen, N.-X., Chen, W., Cheng, Y.-Q., et al. (2022). The DIRECT consortium and the REST-meta-MDD project: towards neuroimaging biomarkers of major depressive disorder. Psychoradiology *2*, 32–42. https://doi.org/10.1093/psyrad/kkac005.
101. Bezmaternykh, D.D., Mel'Nikov, M.E., Petrovskii, E.D., Kozlova, L.I., Shtark, M.B., Savelov, A.A., Shubina, O.S., and Natarova, K.A. (2018). Spontaneous Changes in Functional Connectivity of Independent Components of fMRI Signal in Healthy Volunteers at Rest and in Subjects with Mild Depression. Bull. Exp. Biol. Med. *165*, 325–330. https://doi.org/10.1007/s10517-018-4161-3.
102. Disantostefano, J. (2009). International Classification of Diseases 10th Revision (ICD-10). J. Nurse Pract. *5*, 56–57.
103. Yan, C.-G., Wang, X.-D., Zuo, X.-N., and Zang, Y.-F. (2016). DPABI: Data Processing & Analysis for (Resting-State) Brain Imaging. Neuroinformatics *14*, 339–351. https://doi.org/10.1007/s12021-016-9299-4.
104. Power, J.D., Mitra, A., Laumann, T.O., Snyder, A.Z., Schlaggar, B.L., and Petersen, S.E. (2014). Methods to detect, characterize, and remove motion artifact in resting state fMRI. Neuroimage *84*, 320–341. https://doi.org/10.1016/j.neuroimage.2013.08.048.
105. Jenkinson, M., Beckmann, C.F., Behrens, T.E.J., Woolrich, M.W., and Smith, S.M. (2012). FSL. Neuroimage *62*, 782–790. https://doi.org/10.1016/j.neuroimage.2011.09.015.
106. Asan, L., Falfán-Melgoza, C., Beretta, C.A., Sack, M., Zheng, L., Weber-Fahr, W., Kuner, T., and Knabbe, J. (2021). Cellular correlates of gray matter volume changes in magnetic resonance morphometry identified by two-photon microscopy. Sci. Rep. *11*, 4234. https://doi.org/10.1038/s41598-021-83491-8.
107. (2021). Relationship between Dynamic Blood-Oxygen-Level-Dependent Activity and Functional Network Connectivity: Characterization of Schizophrenia Subgroups. Brain Connect. *11*, 430–446. https://doi.org/10.1089/brain.2020.0815.
108. Rolls, E.T., Joliot, M., and Tzourio-Mazoyer, N. (2015). Implementation of a new parcellation of the orbitofrontal cortex in the automated anatomical labeling atlas. Neuroimage *122*, 1–5. https://doi.org/10.1016/j.neuroimage.2015.07.075.
109. Craddock, R.C., James, G.A., Holtzheimer, P.E., 3rd, Hu, X.P., and Mayberg, H.S. (2012). A whole brain fMRI atlas generated via spatially constrained spectral clustering. Hum. Brain Mapp. *33*, 1914–1928. https://doi.org/10.1002/hbm.21333.
110. Faulkner, P., Paioni, S.L., Kozhuharova, P., Orlov, N., Lythgoe, D.J., Daniju, Y., Morgenroth, E., Barker, H., and Allen, P. (2021). Relationship between depression, prefrontal creatine and grey matter volume. J. Psychopharmacol. *35*, 1464–1472. https://doi.org/10.1177/02698811211050550.
111. van Griethuysen, J.J.M., Fedorov, A., Parmar, C., Hosny, A., Aucoin, N., Narayan, V., Beets-Tan, R.G.H., Fillion-Robin, J.-C., Pieper, S., and Aerts, H.J.W.L. (2017). Computational Radiomics System to Decode the Radiographic Phenotype. Cancer Res. *77*, e104–e107. https://doi.org/10.1158/0008-5472.Can-17-0339.
112. Yan, C.-G., Craddock, R.C., Zuo, X.-N., Zang, Y.-F., and Milham, M.P. (2013). Standardizing the intrinsic brain: Towards robust measurement of inter-individual variation in 1000 functional connectomes. Neuroimage *80*, 246–262. https://doi.org/10.1016/j.neuroimage.2013.04.081.
113. Fortin, J.-P., Cullen, N., Sheline, Y.I., Taylor, W.D., Aselcioglu, I., Cook, P.A., Adams, P., Cooper, C., Fava, M., McGrath, P.J., et al. (2018). Harmonization of cortical thickness measurements across scanners and sites. Neuroimage *167*, 104–120. https://doi.org/10.1016/j.neuroimage.2017.11.024.
114. Kipf, T.N., and Welling, M. (2017). Semi-supervised Classification with Graph Convolutional Networks. International Conference on Learning Representations 2017 (ICLR), 1–14.
115. Li, Q., Han, Z., and Wu, X.-M. (2018). Deeper Insights into Graph Convolutional Networks for Semi-Supervised Learning. In Proceedings of the AAAI conference on artificial intelligence, *32*, pp. 3538–3545. https://doi.org/10.1609/aaai.v32i1.11604.
116. Zhou, J., Cui, G., Hu, S., Zhang, Z., Yang, C., Liu, Z., Wang, L., Li, C., and Sun, M. (2020). Graph neural networks: A review of methods and applications. AI Open *1*, 57–81. https://doi.org/10.1016/j.aiopen.2021.01.001.
117. Luan, S.T., Zhao, M.D., Chang, X.W., and Precup, D. (2019). Break the Ceiling: Stronger Multi-scale Deep Graph Convolutional Networks. 33rd Conference on Neural Information Processing Systems (NeurIPS), 10943–10953. https://arxiv.org/abs/1906.02174.
118. Gretton, A., Bousquet, O., Smola, A., and Schölkopf, B. (2005). Measuring Statistical Dependence with Hilbert-Schmidt Norms. In International conference on algorithmic learning theory *3734*, pp. 63–77. https://doi.org/10.1007/11564089_7.
119. Liu, S. (2024). LGMF-GNN. figshare. Software. https://doi.org/10.6084/m9.figshare.27107440.

Patterns, Volume *5*

Supplemental information

An objective quantitative diagnosis of depression using a local-to-global multimodal fusion graph neural network

Shuyu Liu, Jingjing Zhou, Xuequan Zhu, Ya Zhang, Xinzhu Zhou, Shaoting Zhang, Zhi Yang, Ziji Wang, Ruoxi Wang, Yizhe Yuan, Xin Fang, Xiongying Chen, DIRECT Consortium, Yanfeng Wang, Ling Zhang, Gang Wang, and Cheng Jin

Supplemental Items

Table S1. Cross validation result of LGMF-GNN on SRPBS dataset

		ACC	AUC	Precision	Recall	F1-Score
10-Fold CV	2-Stage	78.22±2.38	78.85±5.50	79.37±9.19	74.05±8.28	75.91±4.27
	End-to-End	78.75±4.55	80.64±5.74	76.80±7.93	82.00±5.79	78.96±4.88
LOSO CV	2-Stage	71.43±5.68	71.72±5.17	72.08±8.12	73.00±5.67	72.30±5.59
	End-to-End	72.64±4.77	73.71±4.12	72.45±5.72	74.35±6.49	73.33±5.70

Table S2. Cross validation result of LGMF-GNN on REST-meta-MDD dataset

	ACC	AUC	Precision	Recall	F1-score
10-Fold CV	71.34±1.50	73.67±2.67	72.20±4.61	73.52±6.26	72.56±2.06
LOSO CV	74.53±9.70	73.46±10.89	74.71±9.98	77.21±11.90	75.57±9.45

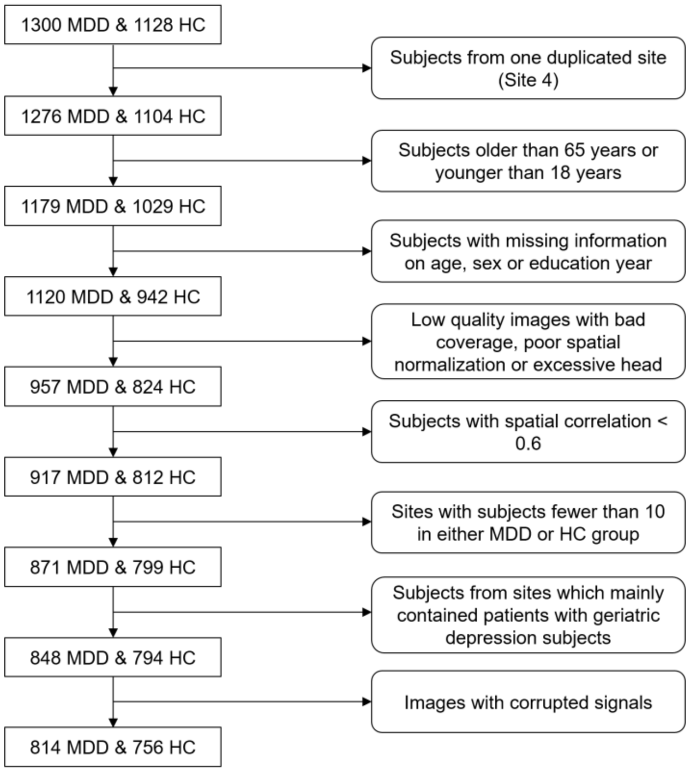

Figure S1. The flowchart of data selection on REST-meta-MDD dataset

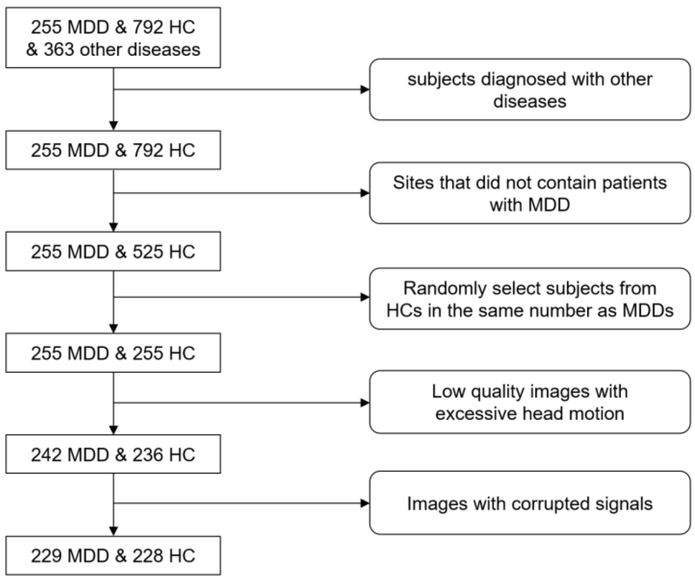

Figure S2. The flowchart of data selection on SRPBS dataset

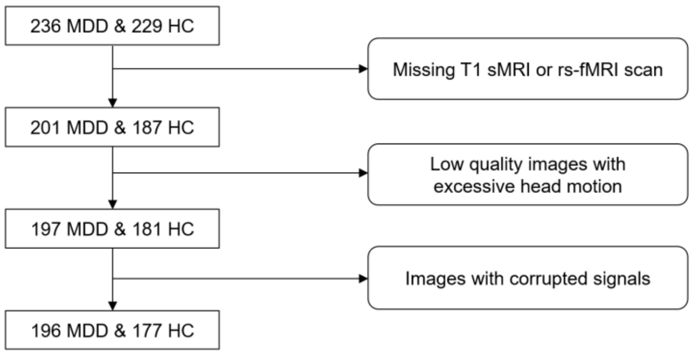

Figure S3. The flowchart of data selection on Anding dataset

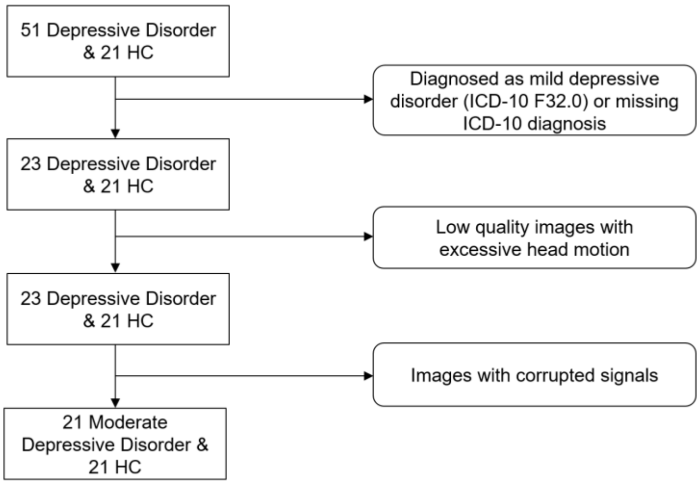

Figure S4. The flowchart of data selection on Openneuro dataset

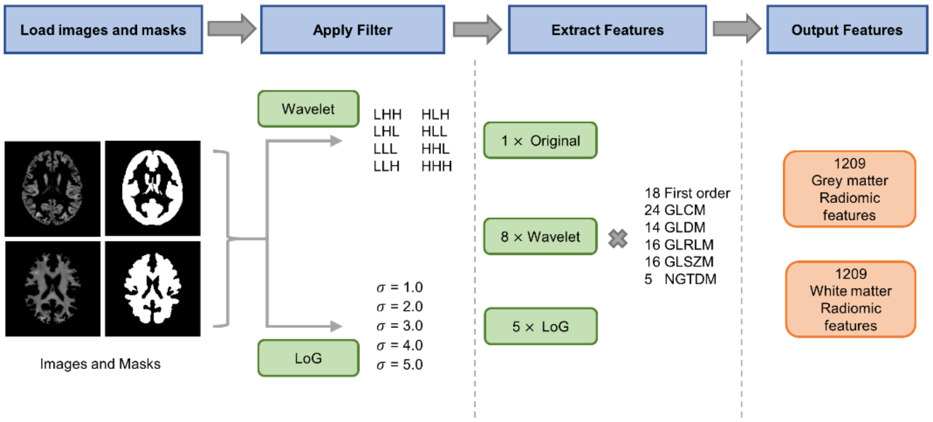

Figure S5. Overview of the feature extraction process using PyRadiomics. Grey matter or white matter volumes and segmentation masks are loaded into PyRadiomics first. Next, Laplacian of Gaussian filters (Sigma=2.0,3.0,4.0,5.0) and Wavelet filters were applied to the original image, resulting in 12 derived images. Subsequently, radiomic features were extracted from the 13 images, forming two 1209-dimensional feature vectors to represent the anatomical features in grey and white matter.

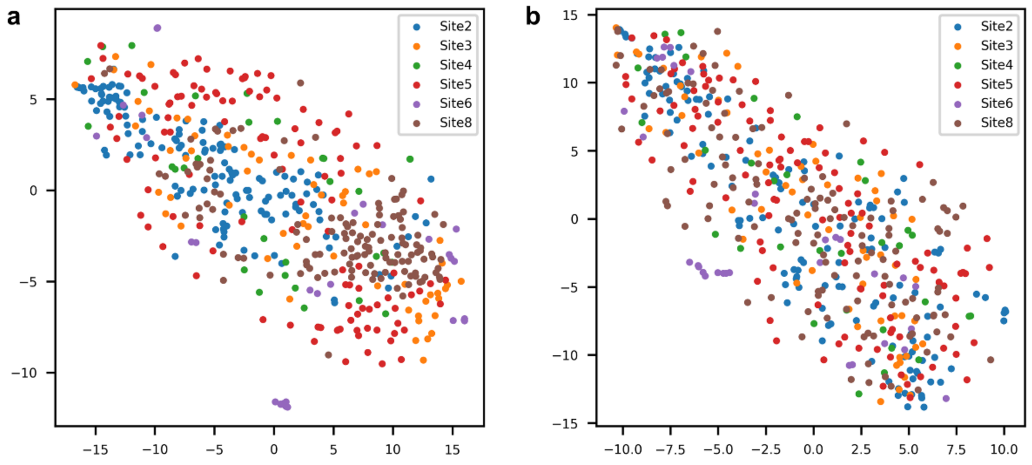

Figure S6. t-SNE plots of SRPBS dataset before and after Combat harmonization

Table S3. Comparison of 10-fold CV results on single-site and multi-site scenarios

	ACC	AUC	Precision	Recall	F1-Score
S20 (1-site)	91.92±4.67	93.41±4.56	89.32±4.56	92.47±7.84	90.62±4.54
SRPBS (6-site)	69.02±3.57	66.33±4.76	72.88±4.55	62.93±2.84	65.94±4.53

Table S4. Category distribution of the FEDN vs HC subset of REST-meta-MDD dataset by site

Site	FEDN	HC	Total
8	28	28	56
9	22	22	44
14	61	32	93
20	97	126	223
23	11	11	22
Total	219	219	438

Table S5. Category distribution of the RECU vs HC subset of REST-meta-MDD dataset by site

Site	RECU	HC	Total
7	11	11	22
9	26	26	52
11	18	17	35
19	12	12	24
20	46	58	104
21	76	65	141
Total	189	189	378

Table S6. Category distribution of the FEDN vs RECU subset of REST-meta-MDD dataset by site

Site	FEDN	RECU	Total
9	22	26	48
20	97	46	143
Total	119	72	191

Table S7. Subtype classification on the REST-meta-MDD dataset

	ACC	AUC	Precision	Recall	F1-Score
FEDN vs. HC	75.13±3.16	76.73±4.57	76.65±8.80	73.12±6.50	74.31±4.08
RECU vs. HC	74.05±4.34	72.28±7.49	73.24±8.44	71.91±6.36	71.98±3.45
FEDN vs. RECU	76.92±8.94	68.89±15.63	78.98±14.81	85.95±11.68	81.35±10.20

Table S8. Comparison with other GNN methods on the Anding dataset

	ACC	AUC	Precision	Recall	F1-Score
**GCN**	54.69	57.75	67.53	26.53	38.10
**GIN**	52.54	53.65	57.60	36.74	44.86
**GAT**	56.84	56.93	64.00	40.82	49.84
**BrainGNN**	55.23	48.89	54.33	**92.86**	68.55
**EV-GCN**	57.11	57.74	60.98	51.02	55.56
**LG-GNN**	57.91	59.92	60.54	57.14	58.79
**ContrastPool**	54.17	54.49	53.06	85.25	65.41
**pRGAT**	54.42	54.14	56.25	59.69	57.92
**MAMF-GCN**	56.84	54.88	59.89	54.08	56.84
**SFGL**	51.21	48.34	53.27	58.16	55.61
**CI-GNN**	57.50	58.84	58.79	59.69	59.24
**IBGNN**	52.01	48.64	52.51	90.82	66.54
**LGMF-GNN**	**69.97**	**72.91**	**71.21**	71.94	**71.57**

Table S9. Comparison with other GNN methods on the Openneuro dataset

	ACC	AUC	Precision	Recall	F1-Score
**GCN**	59.52	55.33	59.09	61.91	60.47
**GIN**	61.91	51.47	66.67	47.62	55.56
**GAT**	66.67	60.32	**68.42**	61.91	65.00
**BrainGNN**	57.14	48.75	63.64	33.33	43.75
**EV-GCN**	66.67	65.08	65.22	71.43	68.18
**LG-GNN**	**69.05**	69.84	68.18	71.43	69.77
**ContrastPool**	65.00	62.66	65.22	71.43	68.18
**pRGAT**	54.76	54.76	60.00	28.57	38.71
**MAMF-GCN**	66.67	64.17	**68.42**	61.91	65.00
**SFGL**	54.76	46.49	54.17	61.91	57.78
**CI-GNN**	71.67	61.68	70.00	66.67	68.29
**IBGNN**	57.14	55.67	55.17	76.19	64.00
**LGMF-GNN**	**69.05**	**70.30**	66.67	**76.19**	**71.11**

Table S10.1. Hyperparameter setting of GCN

Hyperparameter name	Value	Description
num_layer	3	Number of GNN message passing layers
emb_dim	64	Dimensionality of hidden units in GNNs
lr	1e-4	Learning rate of optimizer
batch_size	32	Input batch size for training
drop_ratio	0.6	Dropout ratio
stepsize	50	Scheduler step size
gamma	0.6	Scheduler shrinking rate

Table S10.2. Hyperparameter setting of GIN

Hyperparameter name	Value	Description
num_layer	3	Number of GNN message passing layers
emb_dim	64	Dimensionality of hidden units in GNNs
lr	1e-4	Learning rate of optimizer
batch_size	32	Input batch size for training
drop_ratio	0.6	Dropout ratio
stepsize	50	Scheduler step size
gamma	0.6	Scheduler shrinking rate

Table S10.3. Hyperparameter setting of GAT

Hyperparameter name	Value	Description
num_layer	5	Number of GNN message passing layers
emb_dim	32	Dimensionality of hidden units in GNNs
lr	1e-3	Learning rate of optimizer
batch_size	16	Input batch size for training
drop_ratio	0.5	Dropout ratio
stepsize	50	Scheduler step size
gamma	0.6	Scheduler shrinking rate

Table S10.4. Hyperparameter setting of BrainGNN

Hyperparameter name	Value	Description
num_layer	2	Number of GNN layers
indim	200	Dimensionality of node feature
lr	1e-2	Learning rate of optimizer
batch_size	64	Input batch size for training
ratio	0.5	Pooling ratio
stepsize	20	Scheduler step size
gamma	0.6	Scheduler shrinking rate
lamb0	1	Classification loss weight
lamb1	0	S1 unit regularization
lamb2	0	S2 unit regularization
lamb3	0.1	S1 entropy regularization
lamb4	0.1	S2 entropy regularization
lamb5	0.1	S1 consistence regularization

Table S10.5. Hyperparameter setting of EV-GNN

Hyperparameter name	Value	Description
hgc	16	Hidden units of gconv layer
lg	4	Number of gconv layers
lr	1e-2	Learning rate of optimizer
edropout	0.3	Edge dropout rate

Table S10.6. Hyperparameter setting of LG-GNN

Hyperparameter name	Value	Description
hgc	64	Hidden units of gconv layer
lg	4	Number of gconv layers
lr	1e-2	Learning rate of optimizer
edropout	0.3	Edge dropout rate
topk_ratio	0.5	The ratio of ROIs differ significantly from those of the unselected nodes to be selected
dropout	0.6	Ratio of dropout

Table S10.7. Hyperparameter setting of ContrastPool

Hyperparameter name	Value	Description
batch_size	20	Input batch size for training
Init_lr	1e-2	The initial learning rate
lr_reduce_factor	0.5	Scheduler shrinking rate
lr_schedule_patience	25	Scheduler step size
min_lr	1e-6	Minimum learning rate
Lamda1	1	The weight factor of the entropy loss of the assignment matrix
Lamda2	1e-3	The weight factor of the entropy loss to the adjacency matrix of the contrast graph
dropout	0	The ratio of dropout
L	2	Number of GNN layers
pooling ratio	0.5	The ratio of nodes in the output graph of each ContrastPool layer

Table S10.8. Hyperparameter setting of pRGAT

Hyperparameter name	Value	Description
num_features	2000	Number of features to keep for the feature selection step
connectivity	correlation	Type of connectivity used for network construction
lr	1e-4	Learning rate of optimizer
hid_c	12	Size of hidden layer

Table S10.9. Hyperparameter setting of MAMF-GCN

Hyperparameter name	Value	Description
pheno_edge_threshold	3.1	Graph sparsification edge threshold of the phenotypic grpah
hgc	16	Hidden units of gconv layer
lg	4	Number of gconv layers
lr	0.01	Learning rate of optimizer
node_feature_dim	900	Dimension of node feature
edropout	0.2	Edge dropout rate
dropout	0.2	Ratio of dropout
snowball_layer_num	6	Num of snowball layer
alpha	5e-5	Weight of modal consistency loss
Beta	1e-6	Weight of modal disparsity loss
K	9	Number of the top k neighbors in the k-nearest graph
Stepsize	100	Scheduler step size
gamma	0.6	Scheduler shrinking rate

Table S10.10. Hyperparameter setting of SFGL

Hyperparameter name	Value	Description
minibatch_size	32	Input batch size
window_size	30	The length of the sliding window
window_stride	2	The stride of the sliding window
lr	1e-3	Learning rate of optimizer
reg_lambda	1e-5	Value of lambda
gamma	0.8	Balancing coefficient of the shared branch and the subject-specific branch
num_epochs	8	Local epochs of federal learning
num_iters	20	Communication rounds of federal learning
num_heads	4	The head number of Transformer
num_layers	2	The number of GIN layer
hidden_dim	64	Output dimension of Transfomer
ph	16	Hidden dimension of personalized branch
sparsity	30	Degree of sparsity
dropout	0.5	Ratio of dropout

Table S10.11. Hyperparameter setting of CI-GNN

Hyperparameter name	Value	Description
batch_size	16	Input batch size for training
latent_dim	[128, 128, 128]	Classifier hidden dims
dropout	0.5	Ratio of final layer dropout
lr	1e-3	Learning rate of optimizer
mlp_hidden	[128,128]	Mlp hidden dims
GVAE_hidden_dim	64	Hidden dims of graph VAE
Nalpha	56	Dimension of causal factor alpha
Nbeta	8	Dimension of non-causal factor beta

Table S10.12. Hyperparameter setting of IBGNN

Hyperparameter name	Value	Description
Batch_size	16	Input batch size for training
n_GNN_layers	2	Number of IBGConv layers
n_MLP_layers	1	Number of MLP layers
lr	1e-3	Learning rate of optimizer
initial_epochs	100	Number of epochs for initial training
explainer_epochs	100	Number of epochs to train the explainer
tuning_epochs	100	Number of epochs to fine-tune the whole model

Table S11. Comparison with other GNN methods on the SRPBS dataset

	ACC	AUC	Precision	Recall	F1-Score
**GCN**	70.25±4.44	67.65±7.00	72.06±5.29	66.49±14.76	68.11±8.78
**GIN**	68.51±4.22	65.14±5.05	72.79±9.85	62.51±23.09	63.86±14.32
**GAT**	68.52±4.83	63.90±6.82	67.72±6.29	69.19±12.52	67.94±8.03
**BrainGNN**	63.88±3.55	59.49±5.48	64.87±6.81	61.59±11.36	62.37±6.89
**EV-GCN**	72.87±4.11	71.58±7.46	74.49±6.84	71.62±5.88	72.61±3.29
**LG-GNN**	73.29±5.08	73.04±6.28	**78.03±7.65**	67.69±13.30	71.32±6.73
**ContrastPool**	72.25±6.17	68.30±10.39	67.71±7.42	69.90±15.93	67.76±9.30
**pRGAT**	71.20±10.65	68.95±13.89	71.28±10.82	71.28±16.39	70.73±12.07
**MAMF-GCN**	75.10±4.03	75.25±5.99	75.33±7.11	77.21±10.55	75.43±3.07
**SFGL**	66.52±2.90	62.49±6.30	69.00±7.95	65.57±15.71	65.50±6.07
**CI-GNN**	70.48±4.13	68.34±7.34	71.88±8.27	69.07±8.58	69.78±4.68
**IBGNN**	65.84±5.84	62.82±6.98	69.35±10.22	63.77±18.06	63.98±9.86
**LGMF-GNN**	**78.75±4.55**	**80.64±5.74**	76.80±7.93	**82.00±5.79**	**78.96±4.88**

Table S12. Comparison with other GNN methods on the REST-meta-MDD dataset

	ACC	AUC	Precision	Recall	F1-Score
**GCN**	64.78±3.21	65.73±4.31	71.00±3.68	54.53±4.76	61.55±3.53
**GIN**	63.31±3.15	63.45±4.63	67.14±7.66	59.64±6.07	62.67±2.99
**GAT**	65.92±2.15	66.95±2.97	68.00±5.32	67.87±13.37	66.83±4.95
**BrainGNN**	63.76±2.59	64.24±3.75	64.59±6.00	71.05±13.16	66.58±4.07
**EV-GCN**	66.24±2.80	68.49±4.41	68.70±5.91	66.98±11.58	66.93±4.39
**LG-GNN**	60.64±2.86	59.86±3.52	63.52±3.99	59.33±14.54	60.08±7.85
**ContrastPool**	67.14±2.80	66.80±5.44	**72.37±6.86**	73.49±11.69	71.91±3.99
**pRGAT**	59.43±3.88	56.36±4.94	61.03±4.81	63.57±15.14	61.08±7.78
**MAMF-GCN**	66.94±3.58	66.76±3.52	71.05±3.59	61.43±12.39	65.18±7.43
**SFGL**	66.95±5.40	64.09±5.98	71.30±12.70	66.90±19.29	65.91±8.14
**CI-GNN**	66.05±3.14	64.91±3.52	66.39±4.28	70.77±10.41	68.00±5.30
**IBGNN**	59.68±4.40	58.90±6.53	63.13±5.73	57.93±19.56	57.78±14.25
**LGMF-GNN**	**71.34±1.50**	**73.67±2.67**	72.20±4.61	**73.52±6.26**	**72.56±2.06**

Table S13. Ablation study performance of local ROI-GNN module

	ACC	AUC	Precision	Recall	F1-Score
LGMF-GNN	78.75±4.55	80.64±5.74	76.80±7.93	82.00±5.79	78.96±4.88
-local ROI-GNN	75.10±4.62	75.96±5.41	76.39±7.21	72.77±8.67	74.31±3.94

Table S14. Ablation study performance of different data modalities

	ACC	AUC	Precision	Recall	F1-Score
LGMF-GNN	78.75±4.55	80.64±5.74	76.80±7.93	82.00±5.79	78.96±4.88
-Demographic	68.66±7.74	73.81±5.74	68.42±12.41	76.20±14.95	69.89±7.91
-Anatomical	73.72±5.05	74.11±7.14	73.83±8.57	73.64±5.89	73.31±4.79

Table S15. Ablation study performance of loss functions

	ACC	AUC	Precision	Recall	F1-Score
LGMF-GNN	78.75±4.55	80.64±5.74	76.80±7.93	82.00±5.79	78.96±4.88
-$L_{specific}$	76.85±4.78	70.08±6.55	76.31±5.68	76.64±5.93	76.40±5.34
-L_{common}	76.89±3.53	75.90±7.29	77.40±7.30	74.86±8.49	75.70±5.78

Table S16. Effect of site effects suppression methods on model generalization ability

	ACC	AUC	Precision	Recall	F1-Score
LGMF-GNN	69.02±3.57	66.33±4.76	72.88±4.55	62.93±2.84	65.94±4.53
+ Combat	72.67±4.51	71.91±3.32	74.23±4.01	67.84±2.46	70.58±3.74
+ Mixup	76.22±4.53	75.76±5.21	74.03±3.87	76.92±3.05	75.04±3.24
+ DropEdge	76.89±3.98	75.94±4.54	74.93±5.67	78.18±6.03	75.98±5.23
+Adversarial	78.22±2.38	78.85±5.50	79.37±9.19	74.05±8.28	75.91±4.27
+End-to-End	78.75±4.55	80.64±5.74	76.80±7.93	82.00±5.79	78.96±4.88

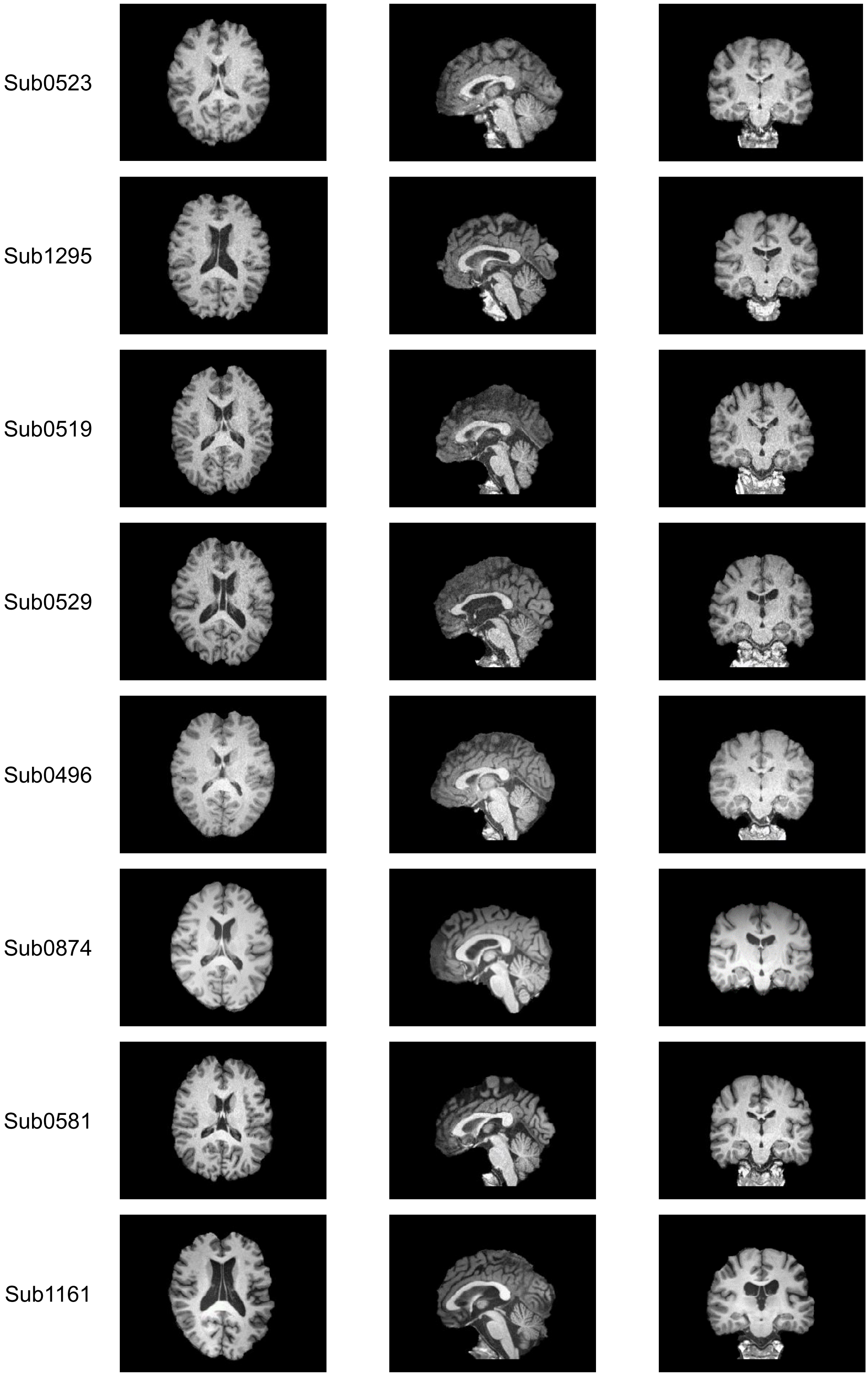

Figure S7. Eight cases corresponding to the MDD nodes with the highest degree in the structural subject graph.

Table S17. Abnormal brain regions and existing research findings

Name in AAL atlas	Brain region name	Existing findings related to functional alteration	Existing findings related to structural alteration
CRBL7b.R, CRBL10.L, CRBL10.R, CRBL8.R	Right Cerebellum lobules VIIB; Left Cerebellum lobules X; Right Cerebellum lobules X; Right Cerebellum lobules VIII;	connectivity analyses have shown reduced cerebro-cerebellar coupling of lobules VI and VIIA/B with prefrontal, posterior parietal, and limbic regions in patients with MDD[18]	The GM density in the right Cerebellum VIII was increased in the MDD patients ($p < 0.05$), and the relative GM densities in the right Cerebellum VIII were significantly correlated with the BDI scores in the MDD patients[19].<br>The GM volumes in right Cerebellum VIII, left Cerebellum X and right Cerebellum X were significantly smaller in the patients with MDD than in the HC subjects ($p < 0.05$), and the GM volumes in right Cerebellum VIII and left Cerebellum X were significantly correlated with the BDI scores in the MDD patients ($p < 0.05$)[19].
HIP.R	Right Hippocampus	In comparison with HCs, patients with BD and MDD had an increased FC between the right anterior hippocampus and lingual gyrus and a decreased FC between the right posterior hippocampus and right IFG ($p<0.00005$)[20];<br>MDD patients demonstrated decreased right hippocampus–bilateral medial superior frontal gyrus FC relative to HC ($p<0.001$)[21];<br>Compared to HC, MDD patients showed decreased node strength of the right	MDD patients have smaller hippocampus volumes even in the earlier phase of their illness ($p<0.01$)[25-27];<br>The cumulative analysis revealed hippocampus volume loss in patients with first-episode depression relative to controls in both the left ($p=0.0321$) and right ($p=0.0173$) hippocampus, and the average volume reduction was −4.0% in the left and −4.5% in the right hippocampus[28].

		hippocampus cornu ammonis 3/4 ($p<0.001$), indicating decreased connectivity to the rest of the brain[22]; The functional connectivity between the right anterior hippocampus and left postcentral gyrus increased with aging in MDD patients compared with HCs ($p<0.05$)[23]; The MDD group exhibited significantly weaker connectivity of the right hippocampal subregional networks with the temporal cortex (extending to the insula) and basal ganglia but showed increased connectivity of the right subiculum to the bilateral lingual gyrus ($p<0.05$)[24]	Adolescents with MDD had reduced hippocampal volume compared with healthy adolescents ($p=0.006$) [29]; Both the MDD and BD patients showed decreased gray matter volume in the right hippocampus compared with the HCs ($p<0.001$)[30]; Hippocampal white matter was significantly smaller in MDDs than in HCs ($p=0.01$)[31].
ROL.L	Left Rolandic operculum	The fronto-limbic system has altered in the first-episode and drug-naïve MDD patients， and increased FC of the left rolandic operculum was found ($p<0.05$)[32]. Compared with old HCs, the late-onset recurrent depression patients had higher ReHo in the left rolandic operculum gyrus/left superior temporal gyrus ($p<0.05$) [33].	-
REC.L	Left gyrus rectus	Compared with HCs, the connectivity between the left rectus gyrus and the left orbital part of the superior frontal gyrus is enhanced in patients with treatment-naive depression ($p=0.0054$)[34]	For structural MRI, the greatest difference between healthy individuals and those with depression could be observed in a voxel in the left gyrus rectus[35]
PreCG.R	Right Precentral gyrus	Compared with HCs, MDD patients had a higher regional cerebral blood flow in the right Precental gyrus (PreCG) ($p<0.001$)[36]; The non-anxious depression patients showed decreased FC	-

		between the right precentral gyrus and the right centromedial /laterobasal compared to the HC group (p<0.001) [37]; There was a significant reduction in ReHo and ALFF in the bilateral precentral gyrus in the somatic MDD group as compared to the pure depression group (p<0.01)[38]; HCs had greater activation compared to MDD+PTSD patients in the right precentral gyrus (p<0.001)[39].	
ROL.R	Right Rolandic operculum	Compared with HCs, patients with MDD had significantly higher ALFF in the left cerebellum and significantly lower ALFF in the right rolandic opercular cortex (p<0.001)[40]; Increased ALFF in the contralesional rolandic operculum was found in the slow-5 frequency band, and this post-stroke depression-specific alteration could predict depression severity [41].	Lesions in the right Rolandic operculum are associated with self-rating affective and apathetic depressive symptoms for post-stroke patients[42].
CRBLCrus2.R	Right Cerebellum Crus II	Compared with HCs, patients with MDD showed significantly increased ReHo values in the right cerebellum Crus2 (p<0.001). Additionally, partial correlation analysis showed that the right cerebellum crus2 and right angular gyrus were positively associated with working memory (r=0.277, p=0.038)[43].	-

Table S18. Key indicators of sMRI imaging radiomics for the diagnosis of depression

**Name**	**Description**	**Implication for Depression Diagnosis**
GLCM_MaximumProbability	The maximum probability of co-occurring pixels within a specified distance and angle, suggesting the homogeneity in the image texture	Alterations in the microstructural organization of gray and white matter; Disruptions in neural connectivity and organization
FirstOrder_Uniformity	The similarity of the intensity distribution in a given region, indicating the uniformity of an image	Disruptions in the typical distribution of grey and white matter intensities; Irregularities in tissue composition which indicate neurostructural changes associated with depression
FirstOrder_Range	The difference between the maximum and minimum voxel intensity values of an image	Variations in tissue density; The magnitude and range of anatomical changes related to depression
GLRLM_RunLengthNonUniformity	The deviation of the run lengths from the average run length value, suggesting the heterogeneity in the texture	Disruptions in the regularity of structural patterns of grey and white matter; Changes in tissue composition, connectivity, or other microstructural features related to depression
GLSZM_GrayLevelNonUniformityNormalized	The degree of variability of discrete voxel intensity values in an image, indicating the heterogeneity of voxel intensities	Irregularities in tissue structure and composition of grey and white matter; Grey and white matter morphological abnormalities associated with depression

Table S19. Imaging parameters used for SRPBS dataset

rs-fMRI						
Protocol #	2	3	4	5	6	8
Site	Hiroshima University Hospital	Hiroshima University Hospital	Hiroshima Kajikawa Hospital	Center of Innovation in Hiroshima University	Kyoto university	University of Tokyo
MRI Scanner	GE Signa HDxt	GE Signa HDxt	SIEMENS Spectra	SIEMENS MAGNETOM Verio.Dot	SIEMENS TimTrio	GE Discovery MR750w
Magnitic field strength	3T	3T	3T	3T	3T	3T
Number of channels per coil	8HR-BRAIN	8HR-BRAIN	head-12ch	head-12ch	32-channel phased array head coil	Head 24
TR (s)	2	2	2.7	2.5	2,500	2.5
TE (ms)	27	27	31	30	30	30
Flip angel (deg)	90	90	90	80	80	80
Phse encoding	PA	AP	AP	AP	PA	PA
Matrix	64 x 64	64 x 64	64 x 64	64 x 64	64 x 64	64 x 64
Field of view (mm)	256	256	192	212	212 x 212	212
In-plane resolution (mm)	4.0 x 4.0	4.0 x 4.0	3.0 x 3.0	3.3 x 3.3	3.3125 X 3.3125	3.3
Slice thickness (mm)	4	4	3	3.2	3.2	3.2

Slice gap (mm)	0	0	0	0.8	0.8	0.8
Number of slices	32	32	38	40	40	40
Slice acquisition order	Ascending (Interleaved)	Ascending (Interleaved)	Ascending	Ascending	Ascending	Ascending
Number of volumes	143 + 7 (dummy)	143 + 7 (dummy)	107 + 5 (dummy)	240 + 4 (dummy)	240 + 4 (dummy)	240 + 4 (dummy)
Total scan time	4 min. 46s. + 14s (dummy)	4 min. 46s. + 14s (dummy)	4 min. 49s. + 14s (dummy)	10 min + 10s (dummy)	10 min + 10s (dummy)	10 min + 10s (dummy)
Eye closed/fixate	Fixate	Fixate	Fixate	Fixate	Fixate	Fixate
**Structural (T1w)**						
Protocol #	2	3	4	5	6	8
Site	Hiroshima University Hospital	Hiroshima University Hospital	Hiroshima Kajikawa Hospital	Center of Innovation in Hiroshima University	Kyoto university	University of Tokyo
Scanner	GE Signa HDxt	GE Signa HDxt	SIEMENS Spectra	SIEMENS MAGNETOM Verio.Dot	SIEMENS TimTrio	GE Discovery MR750w
Voxel size (mm^3)	1 x 1 x 1	1 x 1 x 1	1 x 1 x 1	1 x 1 x 1	0.9375 x 0.9375 x 1.0	1 x 1 x 1.2
TR (ms)	6812	6812	1900	2300	2000	7.7
TE (ms)	1896	1896	2.38	2.98	3.4	3.1
TI (ms)	450	450	900	900	990	400
Flip angel (deg)	20	20	10	9	8	11
FOV	256	256	256	256	225 x 240	240
Matrix	256 x 256	256 x 256	256 x 256	256 x 256	240 x 256	256 x 256

Table S20. Imaging parameters used for REST-meta-MDD dataset

Cohort	Site	MRI Scanner	Magnitic field strength	Receive (coil channel)	TR (s)	TE (ms)	Flip angel (deg)	Field of view (mm)	Voxel size	Slice thickness (mm)	Number of slices	Number of volumes
1	National Clinical Research Center for Mental Disorders (Peking University Sixth Hospital) & Key Laboratory of Mental Health, Ministry of Health (Peking University)	Siemens Tim Trio 3T	3T	32	2000	30	90	210×210	3.28×3.28 ×4.80	4.0/0.8	30	210
2	Department of Clinical Psychology, Suzhou Suzhou Psychiatric Hospital, The Affiliated Guangji Hospital of Soochow University	Philips Achieva 3T	3T	8	2000	30	90	240×240	1.67×1.67 ×4.00	4.0/0	37	200
7	Sir Run Run Shaw Hospital, Zhejiang University School of Medicine	GE discovery MR750	3T	8	2000	30	90	220×220	2.29×2.29 ×3.20	3.2/0	37	184
8	Department of Psychiatry, First Affiliated Hospital, China Medical University	GE Signa 3T	3T	8	2000	30	90	240×240	3.75×3.75 ×3.00	3.0/0	35	200

9	The First Affiliated Hospital of Jinan University	GE Discovery MR750 3.0T	3T	8	2000	25	90	240×240	3.75×3.75 ×4.00	3.0/1.0	35	200
10	First Hospital of Shanxi Medical University	Siemens Tim Trio 3T	3T	32	2000	30	90	240×240	3.75×3.75 ×4.52	3.0/1.52	32	212
11	Department of Psychiatry, The First Affiliated Hospital of Chongqing Medical University	GE Signa 3T	3T	8	2000	30	90	240×240	3.75×3.75 ×5.00	5	33	200
13	The First Affiliated Hospital of Xi'an Jiaotong University, Xi'an Central Hospital	GE Excite 1.5T	1.5T	16	2500	35	90	256×256	4.00×4.00 ×4.00	4/0	36	150
14	The Second Xiangya Hospital of Central South University	Siemens Tim Trio 3T	3T	32	2500	25	90	240×240	3.75×3.75 ×3.50	3.5/0	39	200
15	Department of Psychosomatics and Psychiatry, Zhongda Hospital, School of Medicine, Southeast University	Siemens Verio 3.0T MRI	3T	12	2000	25	90	240×240	3.75×3.75 ×4.00	4/0	36	240
17	Department of Psychiatry, The First Affiliated Hospital of Chongqing Medical	GE Signa 3T	3T	8	2000	40	90	240×240	3.75×3.75 ×4.00	4.0/0	33	240

	University											
19	Anhui Medical University	GE Signa 3T	3T	8	2000	22.5	30	220×220	3.44×3.44 ×4.60	4.0/0.6	33	240
20	Faculty of Psychology, Southwest University	Siemens Tim Trio 3T	3T	12	2000	30	90	220×220	3.44×3.44 ×4.00	3.0/1.0	32	242
21	Beijing Anding Hospital, Capital Medical University	Siemens Tim Trio 3T	3T	32	2000	30	90	200×200	3.12×3.12 ×4.20	3.5/0.7	33	240
22	The Institute of Mental Health, Second Xiangya Hospital of Central South University	Philips Gyroscan Achieva 3.0T	3T	32	2000	30	90	240×240	1.67×1.67 ×4.00	4.0/0	36	250
23	Mental Health Center, West China Hospital, Sichuan University	Philips Achieva 3.0T TX	3T	8	2000	30	90	240×240	3.75×3.75 ×4.00	4.0/0	38	240

Table S21. Category distribution of the included SRPBS dataset participants by site

Site	MDD	HC	Total
2	49	49	98
3	16	14	30
4	32	25	57
5	65	65	130
6	16	15	31
8	51	60	111
Total	229	228	457

Table S22. Category distribution of the included REST-meta-MDD dataset participants by site

Site	MDD	HC	Total
1	73	73	146
2	16	14	30
7	35	37	72
8	39	48	87
9	48	48	96
10	45	26	71
11	20	17	37
13	20	16	36
14	61	32	93
15	30	37	67
17	41	41	80
19	18	31	49
20	249	228	477
21	79	65	144
22	18	20	38
23	22	23	45
Total	814	756	1570

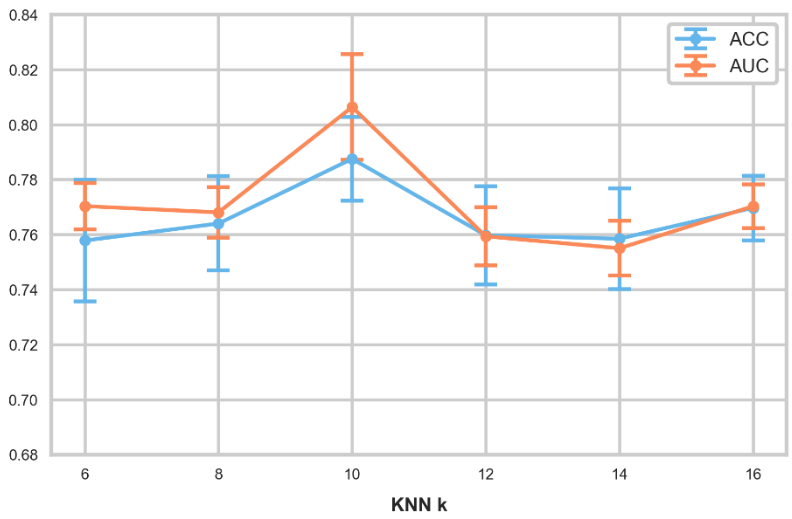

Figure S8. Experimental results for the optimal number of nearest neighbors during KNN graph construction.

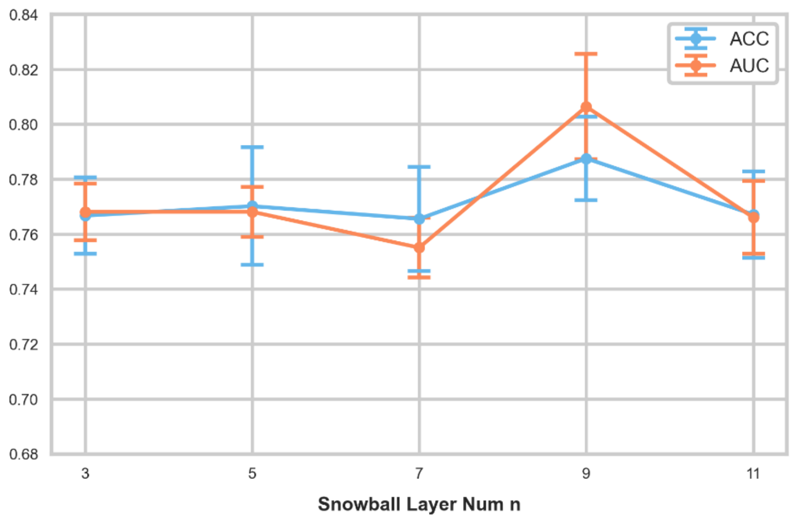

Figure S9. Experimental results for the optimal number of Snowball Layer.

Table S23. The impact of brain atlases on model performance.

	ACC	AUC	Precision	Recall	F1-Score
2atlas	74.57±4.16	74.52±5.53	75.48±9.76	72.04±3.36	73.42±5.45
AAL	**78.75±4.55**	**80.64±5.74**	**76.80±7.93**	**82.00±5.79**	**78.96±4.88**
CC200	76.63±4.97	76.72±8.18	76.44±9.67	75.46±10.77	75.49±8.36

Table S24. Performance of LGMF-GNN on the unbalanced SRPBS validation dataset.

	ACC	AUC	Precision	Recall	F1-Score
LGMF-GNN	86.50±8.08	82.97±11.19	68.40±20.31	87.50±16.77	73.50±12.76

Table S25. The full list of DIRECT consortium members

Chao-Gan Yan[1,2,3,4,5,6], Xiao Chen[1,2,3,4], Li-Ping Cao[9], Wei Chen[11], Yu-Qi Cheng[12], YiRu Fang[13], Qi-Yong Gong[14,15], Wen-Bin Guo[16], Li Kuang[17], Bao-Juan Li[18], Tao Li[20,21], Yan-Song Liu[22], Zhe-Ning Liu[16], Jian-Ping Lu[23], Qing-Hua Luo[17], Hua-Qing Meng[17], Dai-Hui Peng[13], Jiang Qiu[24], Yue-Di Shen[25], Tian-Mei Si[26], Yan-Qing Tang[27], Chuan-Yue Wang[28], Fei Wang[27,29], Hua-Ning Wang[18], Kai Wang[30], Xiang Wang[16], Ying Wang[31], Xiao-Ping Wu[32], Chun-Ming Xie[33], Guang-Rong Xie[16], Peng Xie[34,35,36], Xiu-Feng Xu[12], Hong Yang[37], Jian Yang[35], Shu-Qiao Yao[16], Yong-Qiang Yu[38], Yong-Gui Yuan[39], Ke-Rang Zhang[40], Wei Zhang[41], Zhi-Jun Zhang[33], Jun-Juan Zhu[42], Xi-Nian Zuo[43,44], Jing-Ping Zhao[16], Yu-Feng Zang[45,46]

[1]CAS Key Laboratory of Behavioral Science, Institute of Psychology, Chinese Academy of Sciences, Beijing 100101, China
[2]International Big-Data Center for Depression Research, Chinese Academy of Sciences, Beijing 100101, China
[3]Magnetic Resonance Imaging Research Center, Institute of Psychology, Chinese Academy of Sciences, Beijing 100101, China
[4]Department of Psychology, University of Chinese Academy of Sciences, Beijing 100049, China
[5]Sino-Danish College, University of Chinese Academy of Sciences, Beijing 101408, China
[6]Sino-Danish Center for Education and Research, Graduate University of Chinese Academy of Sciences, Beijing 101408, China
[9]Affiliated Brain Hospital of Guangzhou Medical University, Guangzhou 510370, China
[11]Department of Psychiatry, Sir Run Run Shaw Hospital, Zhejiang University School of Medicine, Hangzhou 310020, Zhejiang, China
[12]Department of Psychiatry, First Affiliated Hospital of Kunming Medical University, Kunming, Yunnan 650032, China
[13]Shanghai Mental Health Center, Shanghai Jiao Tong University School of Medicine, Shanghai 200030, China
[14]Huaxi MR Research Center (HMRRC), Department of Radiology, West China Hospital of Sichuan University, Chengdu, Sichuan 610044, China
[15]Research Unit of Psychoradiology, Chinese Academy of Medical Sciences, Chengdu, Sichuan 610052, China
[16]Department of Psychiatry, and National Clinical Research Center for Mental Disorders, The Second Xiangya Hospital of Central South University, Changsha 410011, Hunan, China
[17]Department of Psychiatry, The First Affiliated Hospital of Chongqing Medical University, Chongqing 400042, China
[18]Xijing Hospital of Air Force Military Medical University, Xi'an, Shaanxi 710032, China
[20]Affiliated Mental Health Center & Hangzhou Seventh People's Hospital, Zhejiang University School
of Medicine, Hangzhou, Zhejiang 310063, China
[21]Mental Health Center and Psychiatric Laboratory, West China Hospital of Sichuan University, Chengdu, Sichuan 610044, China
[22]Department of Clinical Psychology, Suzhou Psychiatric Hospital, The Affiliated Guangji Hospital of Soochow University, Suzhou, Jiangsu 215003, China
[23]Shenzhen Kangning Hospital, Shenzhen, Guangzhou 518020, China
[24]Faculty of Psychology, Southwest University, Chongqing 400715, China
[25]Department of Diagnostics, Affiliated Hospital, Hangzhou Normal University Medical School, Hangzhou, Zhejiang 311121, China
[26]National Clinical Research Center for Mental Disorders (Peking University Sixth Hospital) & Key Laboratory of Mental Health, Ministry of Health (Peking University), Beijing 100191, China
[27]Department of Psychiatry, First Affiliated Hospital, China Medical University, Shenyang, Liaoning 110122, China
[28]Beijing Anding Hospital, Capital Medical University, Beijing 100120, China
[29]Early Intervention Unit, Department of Psychiatry, Affiliated Nanjing Brain Hospital, Nanjing Medical University, Nanjing 210024, China
[30]Department of Neurology, The First Affiliated Hospital of Anhui Medical University, Hefei,

Anhui 230022, China
[31]The First Affiliated Hospital of Jinan University, Guangzhou, Guangdong 250024, China
[32]Xi'an Central Hospital, Xi'an, Shaanxi 710004, China
[33]Department of Neurology, Affiliated ZhongDa Hospital of Southeast University, Nanjing, Jiangsu 210009, China
[34]Institute of Neuroscience, Chongqing Medical University, Chongqing 400016, China
[35]Chongqing Key Laboratory of Neurobiology, Chongqing 400000, China
[36]Department of Neurology, The First Affiliated Hospital of Chongqing Medical University, Chongqing 400042, China
[37]Department of Radiology, The First Affiliated Hospital, College of Medicine, Zhejiang University, Hangzhou, Zhejiang 310058, China
[38]The First Affiliated Hospital of Anhui Medical University, Hefei, Anhui 230032, China
[39]Department of Psychosomatics and Psychiatry, Zhongda Hospital, School of Medicine, Southeast University, Nanjing, Jiangsu 210009, China
[40]First Hospital of Shanxi Medical University, Taiyuan, Shanxi 030001, China
[41]West China Hospital of Sichuan University, Chengdu, Sichuan 610044, China
[42]Department of Psychiatry, Shanghai Jiao Tong University School of Medicine, Shanghai 200025, China
[43]Developmental Population Neuroscience Research Center, IDG/McGovern Institute for Brain Research, Beijing Normal University, Beijing 100091, China
[44]National Basic Science Data Center, Beijing 100038, China
[45]Center for Cognition and Brain Disorders, The Affiliated Hospital of Hangzhou Normal University, Hangzhou, Zhejiang 310018, China
[46]Zhejiang Key Laboratory for Research in Assessment of Cognitive Impairments, Hangzhou, Zhejiang 310000, China

Supplemental Experimental Procedures

Supplemental Experimental Procedures. Data cleaning and quality control

REST-meta-MDD:
As shown in **Figure. S1**. To control the data quality and avoid introduction of bias, subjects were selected from a total of 2428 subjects as follows: (1) subjects older than 65 years or younger than 18 years were excluded; (2) subjects with missing information on age, sex or education year were excluded; (3) low quality images with bad coverage (< 90% group mask), poor spatial normalization (visual inspection) or excessive head motion (mean framewise displacement (FD) > 0.2) were discarded; (4) to further exclude subjects with distortions that were not detected by visual inspection, we excluded subjects with spatial correlation < 0.6 (a threshold defined by mean - 2SD) between each subject's regional homogeneity (ReHo) map and the group mean ReHo map; (5) sites with subjects fewer than 10 in either MDD or HC group was removed; (6) sites mainly contained patients with geriatric depression subjects were excluded; (7) one duplicated site (detected after consortium data sharing) was discarded; (8) images with zero signals detected in targeted atlas were discarded.

SRPBS:
As shown in **Figure. S2**. To control the data quality and avoid introduction of bias, subjects were selected from a total of 1410 subjects as follows: (1) subjects diagnosed with other disease were excluded; (2) subjects from sites that did not contain MDD patients were excluded; (3) to make the data more balanced, subjects with the same number of MDDs were randomly selected from HCs; (4) low quality images with excessive head motion (mean framewise displacement (FD) > 0.4) were discarded; (5) images with zero signals detected in targeted atlas were discarded.

Anding:
As shown in **Figure. S3**. To control the data quality and avoid introduction of bias, subjects were selected from a total of 465 subjects as follows: (1) subjects missing T1 sMRI or rs-fMRI scan were excluded; (2) low quality images with excessive head motion (mean framewise displacement (FD) > 0.2) were discarded; (3) images with zero signals detected in targeted atlas were discarded.

Openneuro:
As shown in **Figure. S4**. To control the data quality and avoid introduction of bias, subjects were selected from a total of 72 subjects as follows: (1) subjects diagnosed as mild depressive disorder (ICD-10 F32.0) or missing ICD-10 diagnosis were excluded; (2) low quality images with excessive head motion (mean framewise displacement (FD) > 0.2) were discarded; (3) images with zero signals detected in targeted atlas were discarded.

Supplemental Experimental Procedures. The evaluation of site-effect

The datasets utilized in this study exhibit high heterogeneity. Specifically, the SRPBS dataset was collected from six different sites with varying acquisition protocols, and the participants in the REST-meta-MDD dataset included in this study involves 16 cohorts from different hospitals. To make reasonable and adequate use of these experimental data, multicenter correction is a necessary and important step. Multicenter correction involves harmonizing fMRI-derived data from multiple acquisition sites, study cohorts or devices. The purpose of this correction is to eliminate site effects that arise from differences in image scanners, scanning parameters, and image processing flows. Strong site effects can impact the reliability, reproducibility, and generalization ability of the data, which, in turn, can affect the interpretation of the results.

To evaluate the extent of site effects, we initially conducted experiments from two perspectives: 1) The data distribution, specifically the brain functional connectivity matrix calculated based on the BOLD signal, and 2) The interference with the model's generalization ability.

1) The data distribution For the brain functional connectivity matrix calculated from fMRI, the upper triangle of the functional connectivity matrix based on the AAL atlas was flattened to a one-dimensional vector and then normalized by the Z-Score standardization method in this paper. To visualize the site effects present in the functional connectivity matrix data, dimensional reduction using t-SNE is performed, followed by creating a scatter plot to illustrate the data distribution. **Figure S6** reveals that before correction, the data from each site has a tendency to aggregate and there are more outliers, indicating that the original functional connectivity data derived from fMRI has strong site effects. Directly inputting this data into the model would lead to confusion and confounding.

2) The interference with the generalization ability To examine the impact of site effects on model's performance, we conducted a pre-experiment using single-site data from site 20 of the REST-meta-MDD dataset and data from six sites of the SRPBS dataset. We employed the global Subject-GNN network for ten-fold cross-validation, and the results are shown in **Table S3**. It is evident that the model performs significantly better on the single-site dataset than on the multi-site dataset, with the cross-validation results for the multi-site dataset showing a decrease of nearly 20% in comparison to the single site. This indicates that site effects have a significant impact on both the training and generalization performance of the model. Therefore, the development of an effective multicenter correction method is essential to mitigate the influence of site effects.

Supplemental Experimental Procedures. System performance for MDD subtype classification

We conducted an experiment where we divided the MDD patients in the REST-meta-MDD dataset into two subsets: “first-episode drug-naïve” (FEDN) and “recurrent” (RECU).

The subset partition according to FEDN and RECU references the previous work[1] published in PNAS by the REST-meta-MDD dataset providers, aiming to explore the model’s ability to distinguish different disease subtypes. The medication status was further considered for the first-episode group primarily because this group has detailed and reliable medication records in the dataset. Moreover, medication use may affect the physiological and psychological states of patients, thereby influencing diagnostic accuracy. Excluding the influence of medication for first-episode patients helps to more clearly identify the initial state and characteristics of depression. The medication status was not specifically considered for the recurrent group because these patients may have already received pharmacological treatment in previous episodes, and the dataset lacks detailed records of their medication use. Additionally, recurrence itself indicates the uniqueness and complexity of the disease. In this case, the research focus may shift more towards understanding the recurrence mechanisms and seeking more effective treatments.

For the FEDN vs HC subset and the RECU vs HC subset, we first filtered all FEDN and RECU subject samples from the REST-meta-MDD dataset. We then randomly sampled an equal number of HC samples from the sites where the FEDN and RECU subjects originated. In cases where the number of HC at a particular site was fewer than MDD, we supplemented HC samples from site 20, which had the largest data volume, to achieve a 1:1 ratio of HC to MDD in the subset. For the FEDN vs RECU subset, we only selected samples from the overlapping acquisition sites (i.e., site 9 and site 20) for the two subtypes. The category distribution across sites after filtering is shown in T**able S4-S6**. And the performance of the proposed system for MDD subtype classification is shown in **Table S7**.

Supplemental Experimental Procedures. Comparison experiments

To further validate the proposed system, LGMF-GNN is compared to state-of-the-art (SOTA) graph neural network models for brain disease diagnosis. Specifically, we assessed the performance of LGMF-GNN against traditional GCN[2], GIN[3], and GAT[4] networks and popular brain graph neural networks, namely BrainGNN[5], EV-GCN[6], LG-GNN[7], ContrastPool[8], pRGAT[9], MAMF-GCN[10], SFGL[11], CI-GNN[12] and IBGNN[13]. Among these, BrainGNN, SFGL, CI-GNN and IBGNN are local-view methods based on graph-level classification of ROI graphs, EV-GNN, pRGAT and MAMF-GCN are global-view methods based on node-level classification on population graphs, and LG-GNN and ContrastPool are local-to-global methods

Here, we briefly introduce the experimental design and the setting of hyperparameters of the comparison method.

All the comparison methods outlined below utilize the same loss functions and optimizers as described in the original paper. For methods such as GCN, GIN, and GAT, which are not specifically designed for brain graphs, we have provided a detailed description of how the graph and node features are constructed. For methods like BrainGNN, which are tailored for brain graphs, we have strived to maintain consistency with the original paper in terms of network architecture and hyperparameter selection in our implementation. In cases where the hyperparameters are not applicable due to changes in atlas or usage scenarios, such as different disease types, we have conducted random searches within the parameter space defined in the original paper to determine the optimal hyperparameter settings for the comparative experiments. The hyperparameter settings for each method are detailed in **Table S10**.

1) GCN

GCN is one of the earliest and most influential approaches in the GNN domain. They extend the concept of convolution from Euclidean data to non-Euclidean graph data. The authors motivate the choice of the convolutional architecture via a localized first-order approximation of spectral graph convolutions

In the implementation of GCN, we modeled depression diagnosis as a graph-level classification task, each subject was constructed as a graph, and the ROIs defined by AAL atlas were used as nodes. The node features were the row vectors corresponding to the functional connectivity matrix calculated by Fisher-Z transformation according to the Pearson correlation coefficient of ROI BOLD signal. The absolute value of the functional connectivity matrix was used as the graph adjacency matrix.

2) GIN

GIN aims to improve on the aggregation mechanism of GCNs by ensuring that the representation of a node is sufficiently influenced by its neighbors, even in the presence of isomorphic subgraphs. The key operation in GINs is a "set-aggregation" function that sums the features of a node and its neighbors, followed by a transformation that includes a learnable function of the node's own feature. This design ensures that GINs can distinguish between different nodes, even if they have the same number of neighbors and similar neighbor features, thus enhancing the expressive power of the model.

In our implementation of GIN, the graph and node features were constructed in the same way as GCN.

3) GAT

GAT uses an Attention mechanism that allows different weights to be assigned to different nodes and relies on pairs of neighboring nodes for training, rather than on a specific network structure, and can be used for inductive tasks.

In our implementation of GAT, the graph and node features were constructed in the same way as GCN.

4) BrainGNN

BrainGNN is a graph neural network (GNN) framework to analyze functional magnetic resonance images (fMRI) and discover neurological biomarkers. Considering the special property of brain graphs, the authors design novel ROI-aware graph convolutional (Ra-GConv)

layers that leverage the topological and functional information of fMRI. Motivated by the need for transparency in medical image analysis, BrainGNN contains ROI-selection pooling layers (R-pool) that highlight salient ROIs (nodes in the graph), so that it can infer which ROIs are important for prediction. Furthermore, regularization terms including unit loss, topK pooling (TPK) loss and group-level consistency (GLC) loss were proposed on pooling results to encourage reasonable ROI-selection and provide flexibility to encourage either fully individual-patterns or patterns that agree with group-level data.

In our implementation, brain ROI was defined according to the AAL atlas, and the methods of node feature extraction and dimension reduction and graph construction were consistent with the original paper.

5) EV-GCN

In this paper, the authors present a generalizable framework designed to automatically integrate imaging data with non-imaging data in populations for uncertainty-aware disease prediction. The framework's centerpiece is a learnable adaptive population graph equipped with variational edges. The authors mathematically demonstrate that this graph can be optimized in conjunction with graph convolutional neural networks. To quantify the predictive uncertainty associated with the graph topology, they introduce the novel concept of Monte-Carlo edge dropout. Across four different databases, experimental results indicate that the authors' method can consistently and significantly enhance diagnostic accuracy for Autism spectrum disorder, Alzheimer’s disease, and ocular diseases.

In our implementation, brain ROI was defined according to the AAL atlas, and demographic information included age, sex, and site. The methods of node feature extraction and dimension reduction and graph construction were consistent with the original paper.

6) LG-GNN

The LG-GNN model is a local-to-global graph neural network structured to include a local ROI-GNN component, which is tasked with learning feature embeddings of local brain regions and pinpointing biomarkers. Subsequently, a global Subject-GNN is constructed to discern the relationships between subjects by utilizing the embeddings produced by the local ROI-GNN in conjunction with non-imaging data. The local ROI-GNN is equipped with a self-attention based pooling module that is designed to retain the most crucial embeddings for the classification task. On the other hand, the global Subject-GNN incorporates an adaptive weight aggregation block that generates multi-scale feature embeddings specific to each subject. The LG-GNN model has been rigorously tested using two public datasets for the classification of ASD and AD. The experimental findings revealed that this approach not only achieves state-of-the-art performance but also excels across a range of evaluation metrics.

In our implementation, brain ROI was defined according to the AAL atlas, and demographic information included age, sex, and site. The methods of node feature extraction and dimension reduction and graph construction were consistent with the original paper.

7) ContrastPool

In their paper, the authors propose a contrastive dual-attention block, which adaptively assigns a weight to each ROI of each subject, performs adaptive aggregation over subjects in each group, and makes contrast across different groups to obtain a contrast graph. Guided by the contrast graph, the authors introduce a differentiable graph pooling method called ContrastPool to generate brain network representations that are effective to the task of disease classification. They have applied their method to five resting-state fMRI brain network datasets across three diseases and have shown its superiority compared to state-of-the-art baselines. The case study conducted by the authors confirms that the patterns extracted by their method correspond with the domain knowledge found in neuroscience literature and reveal direct and intriguing insights. Their contributions highlight the potential of ContrastPool in enhancing the understanding of brain networks and neurodegenerative conditions.

In our implementation, brain ROI was defined according to the AAL atlas. The methods of node feature extraction and dimension reduction and graph construction were consistent with the original paper.

8) pRGAT

In an effort to enhance the valid information in ASD prediction, this paper has investigated various methods to construct the population graph, including Phenotype-Edge (P-Edge), fMRI-

Edge (F-Edge), and a combination of phenotype and fMRI-Edge (PF-Edge). Additionally, they have introduced Graph Attention Networks (GAT) to capture the correlation between subjects on the graph's node-features, which was previously overlooked by GCN-based methods. However, the original GAT architecture does not account for edge-features. To leverage the structural information encoded in edge-features, the researchers further introduced relation-aware attention through the Relational Graph Attention Network (RGAT), which is based on GAT. Using the three graph structures and RGAT, they proposed three ASD prediction models: RGAT with P-Edge (p-RGAT), RGAT with F-Edge (f-RGAT), and RGAT with PF-Edge (pf-RGAT). Experiment results have shown that the integration of relation-aware attention through RGAT, based on GAT, has enhanced the ASD prediction model's ability to learn more diverse information, thereby improving the model's generalization ability.

As a comparative method for the proposed model, we utilized the pf-RGAT model structure that exhibited the best performance in the original paper. This model integrates phenotype data and fMRI data to determine the edges between nodes in the population graph. Moreover, it employs the Relational Graph Attention Convolutional layer (RGATConv), which takes into account not only the correlation of node features but also the relevance of edge features, allowing RGAT to more comprehensively capture the complex relationships between individuals. In our implementation, brain ROI was defined according to the AAL atlas, and demographic information included age, sex, and site. The methods of node feature extraction and dimension reduction and graph construction were consistent with the original paper.

9) MAMF-GCN

In this paper, the researchers propose a multi-scale adaptive multi-channel fusion deep graph convolutional network with an attention mechanism (MAMF-GCN) to better integrate features of various modalities and atlases by exploiting multi-channel correlations. An encoder automatically merges one channel with non-imaging data to produce similarity weights between subjects using a similarity perception mechanism. Additional channels generate multi-scale imaging features of fMRI data after processing across different atlases. An adaptive convolution module fuses multi-modal information, applying a deep graph convolutional network (GCN) to extract information from more profound hidden layers. The researchers assessed the performance of the proposed method on the Autism Brain Imaging Data Exchange (ABIDE) dataset and the Major Depressive Disorder (MDD) dataset. Experimental results indicate that the proposed method surpasses many state-of-the-art methods in node classification performance.

In the original paper, the AAL and HO (Harvard-Oxford) atlases were employed to build the fMRI functional k-nearest neighbor (knn) population graph. To maintain alignment with our research, we utilized the AAL and CC200 atlases for constructing the fMRI functional knn population graph in our implementation. Demographic data, including age, sex, and site, were incorporated to create the phenotypic graph. The techniques for node feature extraction, dimensionality reduction, and graph construction were adopted in line with the methods described in the original paper.

10) SFGL

In this paper, the research team has proposed a specificity-aware federated graph learning (SFGL) framework for rs-fMRI analysis and automated brain disorder identification. This framework consists of a server and multiple clients or sites for federated model aggregation and prediction. At each client, the model is divided into a shared branch and a personalized branch. Parameters of the shared branch are sent to the server, while those of the personalized branch stay local, promoting knowledge sharing among sites while maintaining site specificity. In the shared branch, the team employs a spatio-temporal attention graph isomorphism network to learn dynamic fMRI representations. In the personalized branch, they integrate vectorized demographic information (including age, gender, and education years) and functional connectivity networks to retain site-specific characteristics. The representations generated by both branches are then fused for classification purposes. The experimental results on two fMRI datasets, totaling 1218 subjects, indicate that the SFGL framework outperforms several state-of-the-art approaches.

In our implementation, brain ROI was defined according to the AAL atlas, and demographic information included age, sex, and site. The methods of dynamic graph sequence construction and dynamic graph representation learning are consistent with the original paper.

11) CI-GNN

This study presents the CI-GNN, a novel Granger causality-inspired model that intrinsically identifies influential subgraphs within brain networks that are causally tied to diagnostic outcomes, such as differentiating between patients with major depressive disorder and healthy individuals. Unlike conventional approaches, CI-GNN does not necessitate additional interpretive models post-training. It employs a graph variational autoencoder framework to learn subgraph representations that capture both causal and non-causal elements, regulated by a conditional mutual information (CMI) constraint to ensure the fidelity of the causal relationships. Theoretical validation supports the CMI constraint's effectiveness in capturing causality. Empirical assessments against other GNNs and explainers on synthetic and real-world brain disease datasets demonstrate CI-GNN's superior performance and its ability to offer clinically relevant, concise, and reliable explanations.

In our implementation, brain ROI was defined according to the AAL atlas, and the methods of functional graph construction and two-stage training strategy were consistent with the original paper.

12) IBGNN

In this work, an interpretable framework has been proposed to analyze disorder-specific ROIs and key connections. This framework is composed of two main components: a brain-network-oriented backbone model designed for disease prediction, and a globally shared explanation generator that emphasizes disorder-specific biomarkers, including salient ROIs and significant connections. Experiments have been conducted using three real-world datasets of brain disorders, and the results have demonstrated that the framework not only achieves exceptional performance but also successfully identifies meaningful biomarkers.

In our implementation, brain ROI was defined according to the AAL atlas, and the methods of functional graph construction were consistent with the original paper. According to the training method of the original article, we first trained the backbone IBGNN model and then applied the learned explanation mask to fine-tune the whole model to obtain the IBGNN+ model.

Supplemental Experimental Procedures. Ablation study

The ablation study of local ROI-GNN module.

In our proposed LGMF-GNN model, the local ROI-GNN module is designed to capture the BOLD signal time-series features of multiple brain ROI and the functional connectivity patterns between them at a refined scale. It aggregates these features to form graph-level embedding for each individual, which are subsequently utilized as feature vectors for the functional graph nodes in the global Subject-GNN. In the ablation experiment concerning this module, we removed the local ROI-GNN from the LGMF-GNN and instead used functional connectivity as the node features for the subject-GNN functional subject graph. Specifically, we computed the Pearson correlation coefficients between the ROI BOLD signals for each individual to obtain a functional connectivity matrix, which was then flattened into a vector using its upper triangle for node features. The results of this ablation are presented in **Table S13**. It is evident that the absence of the local ROI-GNN module resulted in a marked degradation in model performance across all metrics. This indicates that the fine-grained functional connectivity features and BOLD signal time-series information provided by the local ROI-GNN are instrumental in the diagnostic process for depression. Additionally, it is noteworthy that the local ROI-GNN plays a pivotal role in enhancing the interpretability of the proposed model by offering a more granular and flexible understanding. Analyzing the functional connectivity matrix learned by the local ROI-GNN allows us to identify the brain regions and changes in functional connectivity strength that the model focuses on for diagnosis.

The ablation study of different modalities

In our manuscript, we introduce a method that leverages three distinct data modalities: functional, anatomical, and demographic. We achieve multi-modal fusion within the global GNN by integrating three subject graphs, each representing one of the modalities. For the ablation experiments, we omitted the anatomical and demographic modalities by removing their respective subject graphs from the global subject GNN, thus focusing on the fusion of the remaining two modalities. The results of these ablation studies are presented in **Table S14**. It can be seen that without demographic or anatomical modality, the performance of the model can be significantly reduced, which proves the significance of integrating multi-modal information in the diagnosis of depression and the effectiveness of LGMF-GNN multi-modal fusion method.

The ablation study of loss functions

In order to achieve efficient multi-modal fusion, we have designed Modality Independent Loss and Modality Common Loss in our paper:

- Modality Independent Loss ($L_{specific}$): This loss term is designed to ensure that the model can extract features that are specific to each modality. By enforcing independence between the modality-specific embeddings and the modality-common embeddings, the model is encouraged to learn unique characteristics of each modality. This is crucial for scenarios where distinct modalities provide complementary information.
- Modality Common Loss (L_{common}): This loss term is responsible for promoting the similarity between the modality-common embeddings across different modalities. By doing so, it helps the model to identify and extract features that are shared among the modalities. This is particularly important for tasks that require integrating information from multiple sources, as it allows the model to capture and leverage the commonalities that exist across different types of data.

To assess the impact of these loss functions on the performance of our LGMF-GNN model, we conducted ablation study and the result are shown in **Table S15**. It can be seen that the performance of the model decreases after the removal of $L_{specific}$ and L_{common}, which proves the help of the two multimodal fusion loss for model training.

Supplemental Experimental Procedures. Methods for suppressing site effects and data enhancement and ablation study

- **Combat harmonization**

The results above indicate that the functional connectivity matrix derived from fMRI images exhibits notable site effects. To mitigate the site effects during data preprocessing, we employed the Combat harmonization method to promote multi-site coordination. The Combat method is essentially a model based on multiple mixed linear regression, which was originally proposed to suppress the batch effect in genomic studies. Due to the effectiveness of this method in genomic studies, this method was later extended to the study of neuroimaging data to remove site-related bias from neuroimaging data acquired from multiple sites and their derived values[14]. Combat uses empirical Bayes to improve parameter estimation of biological effects and site images to avoid overcorrection of important biological variability during the correction process. In this paper, we set the site index or acquisition protocol index as the batch number, and the subject's sex, age, and disease status as covariates to preserve as much site-independent variance as possible during the correction process. To avoid information leakage, a machine learning form of Combat (https://github.com/sahahn/neuroCombat)[14] was used for harmonization. The Combat model underwent a 10-fold cross-validation process to obtain the coordinated data. The t-SNE plot of the functional connectivity matrix after harmonization is shown in Figure S2b, illustrating a more uniform data distribution across all sites, successful elimination of outliers, and effective reduction of site effects on the data.

- **Data Enhancement**

Site effects are primarily caused by the differences in data distribution among sites, which are due to the use of different instruments and parameters for data collection at each site. As a consequence, the training data fails to meet the assumption of an independent and homogeneous distribution necessary for network training and generalization. Furthermore, data-driven methods like graph neural networks necessitate a significant amount of training data to attain satisfactory generalization performance, thereby demanding effective data augmentation techniques to increase the size of the training data. In this paper, DropEdge[15] and a mixup method on graphs were used for data augmentation. DropEdge, which is commonly used in graph neural networks, perturbs the graph structure by randomly removing a portion of the edges in the adjacency matrix during the training process to achieve the effect of data enhancement, thereby alleviating the overfitting phenomenon and enhancing the model generalization performance. The mixup method proposed in this paper extends the Mixup[16] method commonly used in computer vision to graph neural networks, and perturbation of the graph is achieved by linear interpolation of node features and graph structure. Linear interpolation makes the distribution of training data more uniform, which helps suppress the site effects. From the perspective of graph theory, the enhanced data can be viewed as sampling from different graphons, thus achieving data augmentation and improved generalization ability of the model.

Specifically, the Mixup method used in this paper is implemented according to the following equation:

$$\lambda \sim Bern(a,b), y \in \{0,1\} \tag{1}$$

$$\tilde{X}[y] = \lambda X[y] + (1-\lambda)\bar{X}[y] \tag{2}$$

$$\tilde{A}[y] = \lambda X[y] + (1-\lambda)\bar{A}[y] \tag{3}$$

$$\tilde{Y}[y] = \lambda Y[y] + (1-\lambda)\bar{Y}[y] \tag{4}$$

Where $Bern(a,b)$ represents the Bernoulli distribution with parameters a,b. $X,A,Y,$ and $\tilde{X},\tilde{A},\tilde{Y}$ represents the node features, adjacent matrix and classification labels, respectively. $[\cdot]$ represents indexing according to the original label and $\bar{\cdot}$ indicates random permutation by row.

- **Domain adversarial training**

Based on the pre-experimental results, site effects have a substantial impact on both the training and generalization of the model. To reduce site-specific variation in the local embeddings produced by the local ROI-GNN, a domain classifier and a gradient inversion layer were added to the local ROI-GNN in this paper. Specifically, the domain classifier (a 2-layer MLP) identifies the origin site for each local whole-brain embedding. In addition, during the process of gradient backpropagation, a gradient inversion layer is used to reverse the gradients. Through the minimization of the domain classification loss, the domain classifier can be optimized, and the layers preceding it can be encouraged to generate embeddings that confuse the domain classifier. During the model training phase, the layers before the local ROI-GNN domain classifier and the domain classifier engage in an adversarial process and eventually

mitigate the site variance of the local whole-brain embeddings generated by the model.

- **Domain migration loss**

The training and generalization of the model rely on the assumption that the labeled training nodes are randomly sampled and that the test nodes and training nodes follow the independent and identical distribution (iid). This allows the patterns and information learned from the training set to be successfully applied to the test set. However, in a multi-site scenario, particularly when the training set and test set come from different sites, satisfying the assumption of iid becomes challenging. As a result, site effects can significantly hinder the model's ability to generalize. In such cases, the model is more likely to fit redundant rules from the training data, leading to a loss in generalization performance. Therefore, inspired by the work of Qi Zhu et al[17], this paper used a domain migration loss to migrate GNNs from partial site-biased data to complete graph data containing all sites. Specifically, the distribution bias of GNN is defined as follows: assume that the n node embeddings are $H = \{h_1, h_2, \dots, h_n\}$, from which M nodes are labeled $\{(x_i, y_i)\}$ and represented as $H_{train} = \{h_1, \dots, h_m\}$. Assuming that H and H_{train} originate from two different probability distributions p, q, the distribution bias in GNN can be quantified by the distance metric $d(H, H_{train})$. In the training phase, this paper randomly sampled the same number of embeddings from H_{train} and H_{test} and calculated the Central Moment Discrepancy (CMD) distance between them as the domain migration loss:

$$L_{CMD}(H_{train}, H_{test}) = \frac{1}{b-a} \|E(H_{train}) - E(H_{test})\| + \Sigma_{k=2}^{\infty} \frac{1}{|b-a|^k} \|c_k(H_{train}) - c_k(H_{test})\| \quad (5)$$

During the experimental phase, we systematically integrated these methods and evaluated their efficiency in enhancing the model’s generalization performance using 10-fold cross-validation on the SRPBS dataset. The results are summarized in **Table S16**, which demonstrates that the Combat harmonization, Mixup data augmentation, and adversarial training achieved a significant improvement in model generalization ability.

Supplemental Experimental Procedures. Hyperparameter Selection

To further investigate the impact of the number of neighbors (KNN k) for each node in constructing the K-nearest neighbor graph and the number of network layers for Snowball GCN (Snowball Layer Num) on the performance of the model, we explored the changes in model performance under different hyperparameter and model structure settings using grid search on the SRPBS dataset, as shown in **Figure S8** and **Figure S9**. It can be observed that the model's performance remains relatively stable with the variation of hyperparameters. The model achieves the best performance when the number of neighbors in the K-nearest graph is 10 and the number of network layers in Snowball GCN is 9. These two parameter settings can be considered as influencing the receptive field of LGMF-GNN. When the number of neighboring nodes is small or the network depth is shallow, the network aggregates and updates node representations without accessing enough information from neighboring nodes, leading to insufficient information in the node representations. In contrast, when the number of neighboring nodes is large or the network depth is deep, the network introduces excessive noise information into the node representations, resulting in a decrease in model performance. When the number of neighboring nodes and network layers are appropriately balanced and at a moderate level, the network can effectively aggregate and update information within the desired range, thus avoiding excessive noise and contributing to improved performance.

Supplemental Experimental Procedures. The impact of brain atlases on model performance

In this study we have tried 2 brain atlases, the AAL atlas and the CC200 atlas, to define the region of interest (ROI) in participants' brains. In order to investigate the impact of ROI partitioning on the diagnosis of depression, we examined the changes in the 10-fold cross-validation results of the end-to-end LGMF-GNN model on the SRPBS dataset when using different brain atlases. The experimental results showed that the model exhibited robust performance across different ROI partitioning methods (**Table S23**). Additionally, we found that the model performed better when the ROI partitioning was more generalized. This may be because overly fine-grained ROI partitioning introduces more noise, thereby increasing the risk of model confusion.

Supplemental Experimental Procedures. Model validation on unbalanced dataset

In practice, the incidence rate of depression in the population is roughly 1/5. To thoroughly investigate the model’s generalization capability on unbalanced datasets, we employed the Bootstrap method to sample the validation sets from the 10-fold cross-validation on the SRPBS dataset, creating validations set with a HC to MDD ratio of 4:1 to mimic the real-world distribution. For each fold, we performed 10 Bootstrap samplings, collected the model’s prediction results for performance metric calculation, and then averaged the Bootstrap results across the 10 folds. The outcomes of the Bootstrap experiments are detailed in **Table S24**.

We acknowledge that the experimental results on the imbalanced dataset do not meet the performance standards we had achieved on the balanced dataset. The performance metrics indicate that the model’s ability to accurately identify MDD cases is slightly compromised when the data distribution is skewed towards the HC group. Despite the performance degradation in scenarios with unbalanced class distribution, LGMF-GNN still achieved similar performance to current methods[44,45].

Supplemental References

1. Yan, C.G., Chen, X., Li, L., Castellanos, F.X., Bai, T.J., Bo, Q.J., Cao, J., Chen, G.M., Chen, N.X., Chen, W., et al. (2019). Reduced default mode network functional connectivity in patients with recurrent major depressive disorder. Proc Natl Acad Sci U S A *116*, 9078-9083. 10.1073/pnas.1900390116.
2. Kipf, T.N., and Welling, M. (2016). Semi-supervised classification with graph convolutional networks. arXiv preprint arXiv:1609.02907.
3. Xu, K., Hu, W., Leskovec, J., and Jegelka, S. (2019). How powerful are graph neural networks? International Conference on Learning Representations 2019. ICLR.
4. Veličković, P., Cucurull, G., Casanova, A., Romero, A., Lio, P., and Bengio, Y. (2018). Graph attention networks. International Conference on Learning Representations 2018. ICLR.
5. Li, X., Zhou, Y., Dvornek, N., Zhang, M., Gao, S., Zhuang, J., Scheinost, D., Staib, L.H., Ventola, P., and Duncan, J.S. (2021). BrainGNN: Interpretable Brain Graph Neural Network for fMRI Analysis. Medical Image Analysis *74*, 102233. 10.1016/j.media.2021.102233.
6. Huang, Y., and Chung, A.C.S. (2020). Edge-Variational Graph Convolutional Networks for Uncertainty-Aware Disease Prediction. held in Cham, 2020//. A.L. Martel, P. Abolmaesumi, D. Stoyanov, D. Mateus, M.A. Zuluaga, S.K. Zhou, D. Racoceanu, and L. Joskowicz, eds. (Springer International Publishing), pp. 562-572.
7. Zhang, H., Song, R., Wang, L., Zhang, L., Wang, D., Wang, C., and Zhang, W. (2022). Classification of Brain Disorders in rs-fMRI via Local-to-Global Graph Neural Networks. IEEE Transactions on Medical Imaging, 1-1. 10.1109/TMI.2022.3219260.
8. Xu, J., Bian, Q., Li, X., Zhang, A., Ke, Y., Qiao, M., Zhang, W., Sim, W.K.J., and Gulyás, B. (2024). Contrastive Graph Pooling for Explainable Classification of Brain Networks. IEEE Transactions on Medical Imaging, 1-1. 10.1109/TMI.2024.3392988.
9. Gu, X., Xie, L., Xia, Y., Cheng, Y., Liu, L., and Tang, L. (2023). Autism spectrum disorder diagnosis using the relational graph attention network. Biomedical Signal Processing and Control *85*, 105090. https://doi.org/10.1016/j.bspc.2023.105090.
10. Pan, J., Lin, H., Dong, Y., Wang, Y., and Ji, Y. (2022). MAMF-GCN: Multi-scale adaptive multi-channel fusion deep graph convolutional network for predicting mental disorder. Computers in Biology and Medicine *148*, 105823. https://doi.org/10.1016/j.compbiomed.2022.105823.
11. Zhang, J., Wang, Q., Wang, X., Qiao, L., and Liu, M. (2024). Preserving specificity in federated graph learning for fMRI-based neurological disorder identification. Neural Networks *169*, 584-596. https://doi.org/10.1016/j.neunet.2023.11.004.
12. Zheng, K., Yu, S., and Chen, B. (2024). CI-GNN: A Granger causality-inspired graph neural network for interpretable brain network-based psychiatric diagnosis. Neural Networks *172*, 106147. https://doi.org/10.1016/j.neunet.2024.106147.
13. Cui, H., Dai, W., Zhu, Y., Li, X., He, L., and Yang, C. (2022). Interpretable Graph Neural Networks for Connectome-Based Brain Disorder Analysis. held in Cham, 2022//. L. Wang, Q. Dou, P.T. Fletcher, S. Speidel, and S. Li, eds. (Springer Nature Switzerland), pp. 375-385.
14. Fortin, J.-P., Cullen, N., Sheline, Y.I., Taylor, W.D., Aselcioglu, I., Cook, P.A., Adams, P., Cooper, C., Fava, M., McGrath, P.J., et al. (2018). Harmonization of cortical thickness measurements across scanners and sites. NeuroImage *167*, 104-120. https://doi.org/10.1016/j.neuroimage.2017.11.024.
15. Rong, Y., Huang, W.-b., Xu, T., and Huang, J. (2019). DropEdge: Towards Deep Graph Convolutional Networks on Node Classification. held in New Orleans, Louisiana, United States, (OpenReview.net).
16. Zhang, H., Cisse, M., Dauphin, Y.N., and Lopez-Paz, D. (2017). mixup: Beyond Empirical Risk Minimization. arXiv:1710.09412. 10.48550/arXiv.1710.09412.
17. Zhu, Q., Ponomareva, N., Han, J., and Perozzi, B. (2021). Shift-Robust GNNs: Overcoming the Limitations of Localized Graph Training Data. arXiv:2108.01099. DOI:10.48550/arXiv.2108.01099.
18. Depping, M.S., Schmitgen, M.M., Kubera, K.M., and Wolf, R.C. (2018). Cerebellar Contributions to Major Depression. Frontiers in Psychiatry *9*. 10.3389/fpsyt.2018.00634.
19. Xu, L.-Y., Xu, F.-C., Liu, C., Ji, Y.-F., Wu, J.-M., Wang, Y., Wang, H.-B., and Yu, Y.-Q.

(2017). Relationship between cerebellar structure and emotional memory in depression. Brain and Behavior *7*, e00738. https://doi.org/10.1002/brb3.738.
20. Fateh, A.A., Long, Z., Duan, X., Cui, Q., Pang, Y., Farooq, M.U., Nan, X., Chen, Y., Sheng, W., Tang, Q., and Chen, H. (2019). Hippocampal functional connectivity-based discrimination between bipolar and major depressive disorders. Psychiatry Research: Neuroimaging *284*, 53-60. https://doi.org/10.1016/j.pscychresns.2019.01.004.
21. Fan, J., Gao, F., Wang, X., Liu, Q., Xia, J., Han, Y., Yi, J., Tan, C., and Zhu, X. (2023). Right amygdala-right precuneus connectivity is associated with childhood trauma in major depression patients and healthy controls. Soc Cogn Affect Neurosci *18*. 10.1093/scan/nsac064.
22. Jacob, Y., Morris, L.S., Verma, G., Rutter, S.B., Balchandani, P., and Murrough, J.W. (2022). Altered hippocampus and amygdala subregion connectome hierarchy in major depressive disorder. Translational Psychiatry *12*, 209. 10.1038/s41398-022-01976-0.
23. Hu, J., Liu, J., Liu, Y., Wu, X., Zhuang, K., Chen, Q., Yang, W., Xie, P., Qiu, J., and Wei, D. (2021). Dysfunction of the anterior and intermediate hippocampal functional network in major depressive disorders across the adult lifespan. Biological Psychology *165*, 108192. https://doi.org/10.1016/j.biopsycho.2021.108192.
24. Shengli, C., Yingli, Z., Zheng, G., Shiwei, L., Ziyun, X., Han, F., Yingwei, Q., and Gangqiang, H. (2022). An aberrant hippocampal subregional network, rather than structure, characterizes major depressive disorder. Journal of Affective Disorders *302*, 123-130. https://doi.org/10.1016/j.jad.2022.01.087.
25. Kaymak, S.U., Demir, B., Şentürk, S., Tatar, I., Aldur, M.M., and Uluğ, B. (2010). Hippocampus, glucocorticoids and neurocognitive functions in patients with first-episode major depressive disorders. European Archives of Psychiatry and Clinical Neuroscience *260*, 217-223. 10.1007/s00406-009-0045-x.
26. Cole, J., Toga, A.W., Hojatkashani, C., Thompson, P., Costafreda, S.G., Cleare, A.J., Williams, S.C., Bullmore, E.T., Scott, J.L., Mitterschiffthaler, M.T., et al. (2010). Subregional hippocampal deformations in major depressive disorder. J Affect Disord *126*, 272-277. 10.1016/j.jad.2010.03.004.
27. Jun, X., Jun, C., Yicheng, Z., Jingfeng, Z., Bo, Y., Liming, X., and Chengyuan, W. (2004). Volumetric MRI analysis of the amygdala and hippocampus in subjects with major depression. Journal of Huazhong University of Science and Technology [Medical Sciences] *24*, 500-502. 10.1007/BF02831120.
28. Cole, J., Costafreda, S.G., McGuffin, P., and Fu, C.H.Y. (2011). Hippocampal atrophy in first episode depression: A meta-analysis of magnetic resonance imaging studies. Journal of Affective Disorders *134*, 483-487. 10.1016/j.jad.2011.05.057.
29. MacMaster, F.P., and Kusumakar, V. (2004). Hippocampal volume in early onset depression. BMC Medicine *2*, 2. 10.1186/1741-7015-2-2.
30. Chen, L., Wang, Y., Niu, C., Zhong, S., Hu, H., Chen, P., Zhang, S., Chen, G., Deng, F., Lai, S., et al. (2018). Common and distinct abnormal frontal-limbic system structural and functional patterns in patients with major depression and bipolar disorder. NeuroImage: Clinical *20*, 42-50. https://doi.org/10.1016/j.nicl.2018.07.002.
31. Frodl, T., Reinhold, E., Koutsouleris, N., Reiser, M., and Meisenzahl, E.M. (2010). Interaction of childhood stress with hippocampus and prefrontal cortex volume reduction in major depression. Journal of Psychiatric Research *44*, 799-807. https://doi.org/10.1016/j.jpsychires.2010.01.006.
32. Liu, Y., Chen, Y., Liang, X., Li, D., Zheng, Y., Zhang, H., Cui, Y., Chen, J., Liu, J., and Qiu, S. (2020). Altered Resting-State Functional Connectivity of Multiple Networks and Disrupted Correlation With Executive Function in Major Depressive Disorder. Frontiers in Neurology *11*. 10.3389/fneur.2020.00272.
33. Sun, J.-f., Chen, L.-m., He, J.-k., Wang, Z., Guo, C.-l., Ma, Y., Luo, Y., Gao, D.-q., Hong, Y., Fang, J.-l., and Xu, F.-q. (2022). A Comparative Study of Regional Homogeneity of Resting-State fMRI Between the Early-Onset and Late-Onset Recurrent Depression in Adults. Frontiers in Psychology *13*. 10.3389/fpsyg.2022.849847.
34. Long, Z., Duan, X., Wang, Y., Liu, F., Zeng, L., Zhao, J.-p., and Chen, H. (2015). Disrupted structural connectivity network in treatment-naive depression. Progress in Neuro-Psychopharmacology and Biological Psychiatry *56*, 18-26. https://doi.org/10.1016/j.pnpbp.2014.07.007.
35. Winter, N.R., Leenings, R., Ernsting, J., Sarink, K., Fisch, L., Emden, D., Blanke, J., Goltermann, J., Opel, N., Barkhau, C., et al. (2022). Quantifying Deviations of Brain

Structure and Function in Major Depressive Disorder Across Neuroimaging Modalities. JAMA Psychiatry *79*, 879-888. 10.1001/jamapsychiatry.2022.1780.
36. Xiong, Y., Chen, R.-S., Wang, X.-Y., Li, X., Dai, L.-Q., and Yu, R.-Q. (2022). Cerebral blood flow in adolescents with drug-naive, first-episode major depressive disorder: An arterial spin labeling study based on voxel-level whole-brain analysis. Frontiers in Neuroscience *16*. 10.3389/fnins.2022.966087.
37. Qiao, J., Tao, S., Wang, X., Shi, J., Chen, Y., Tian, S., Yao, Z., and Lu, Q. (2020). Brain functional abnormalities in the amygdala subregions is associated with anxious depression. Journal of Affective Disorders *276*, 653-659. https://doi.org/10.1016/j.jad.2020.06.077.
38. Liu, P., Tu, H., Zhang, A., Yang, C., Liu, Z., Lei, L., Wu, P., Sun, N., and Zhang, K. (2021). Brain functional alterations in MDD patients with somatic symptoms: A resting-state fMRI study. Journal of Affective Disorders *295*, 788-796. https://doi.org/10.1016/j.jad.2021.08.143.
39. Keller, M., Mendoza-Quiñones, R., Cabrera Muñoz, A., Iglesias-Fuster, J., Virués, A.V., Zvyagintsev, M., Edgar, J.C., Zweerings, J., and Mathiak, K. (2022). Transdiagnostic alterations in neural emotion regulation circuits – neural substrates of cognitive reappraisal in patients with depression and post-traumatic stress disorder. BMC Psychiatry *22*, 173. 10.1186/s12888-022-03780-y.
40. Zhang, L., Wei, X., and Zhao, J. (2022). Amplitude of Low-Frequency Oscillations in First-Episode Drug-Naive Patients with Major Depressive Disorder: A Resting State Functional Magnetic Resonance Imaging Study. Neuropsychiatric Disease and Treatment *Volume 18*, 555-561. 10.2147/ndt.s348683.
41. Wu, X., Wang, L., Jiang, H., Fu, Y., Wang, T., Ma, Z., Wu, X., Wang, Y., Fan, F., Song, Y., and Lv, Y. (2023). Frequency-dependent and time-variant alterations of neural activity in post-stroke depression: A resting-state fMRI study. NeuroImage: Clinical *38*, 103445. https://doi.org/10.1016/j.nicl.2023.103445.
42. Sutoko, S., Atsumori, H., Obata, A., Funane, T., Kandori, A., Shimonaga, K., Hama, S., Yamawaki, S., and Tsuji, T. (2020). Lesions in the right Rolandic operculum are associated with self-rating affective and apathetic depressive symptoms for post-stroke patients. Scientific Reports *10*, 20264. 10.1038/s41598-020-77136-5.
43. Ni, S., Gao, S., Ling, C., Jiang, J., Wu, F., Peng, T., Sun, J., Zhang, N., and Xu, X. (2023). Altered brain regional homogeneity is associated with cognitive dysfunction in first-episode drug-naive major depressive disorder: A resting-state fMRI study. Journal of Affective Disorders *343*, 102-108. https://doi.org/10.1016/j.jad.2023.10.003.
44. Mousavian, M., Chen, J., and Greening, S. (2018). Feature Selection and Imbalanced Data Handling for Depression Detection. held in Cham, 2018//. S. Wang, V. Yamamoto, J. Su, Y. Yang, E. Jones, L. Iasemidis, and T. Mitchell, eds. (Springer International Publishing), pp. 349-358.
45. Mousavian, M., Chen, J., Traylor, Z., and Greening, S. (2021). Depression detection from sMRI and rs-fMRI images using machine learning. Journal of Intelligent Information Systems *57*, 395-418. 10.1007/s10844-021-00653-w.
